# Palladium-catalysed C–H arylation of benzophospholes with aryl halides†

**DOI:** 10.1039/d2sc04311d

**Published:** 2022-08-30

**Authors:** Shibo Xu, Kazutoshi Nishimura, Kosuke Saito, Koji Hirano, Masahiro Miura

**Affiliations:** Innovative Catalysis Science Division, Institute for Open and Transdisciplinary Research Initiatives (ICS-OTRI), Osaka University Suita Osaka 565-0871 Japan k_hirano@chem.eng.osaka-u.ac.jp miura@chem.eng.osaka-u.ac.jp; Department of Applied Chemistry, Graduate School of Engineering, Osaka University Suita Osaka 565-0871 Japan

## Abstract

A palladium-catalysed C–H arylation of benzophospholes with aryl halides has been developed. The reaction with aryl iodides and bromides proceeds well even under phosphine ligand-free Pd(OAc)_2_ catalysis whereas the Pd(PCy_3_)_2_ is effective for the coupling with less reactive aryl chlorides. The optimal conditions are also applicable to the double arylations with organic dihalides and annulation reaction with *ortho*-dihalogenated benzenes, making the corresponding benzophosphole-based acceptor–donor–acceptor-type molecules and highly condensed heteroacene-type molecules of potent interest in materials chemistry. Although there are many reports of catalytic C–H functionalisations of related benzoheteroles such as indoles, benzothiophenes, and benzofurans, this is the first successful example of the catalytic direct C–H transformation of benzophospholes, to the best of our knowledge. The preliminary optoelectronic properties of some newly synthesized benzophosphole derivatives are also investigated.

## Abstract 2

A palladium-catalysed C–H arylation of benzophospholes with aryl halides has been developed to form the corresponding acceptor–donor–acceptor-type molecules and highly condensed heteroacene-type molecules of potent interest in material chemistry.

## Graphical abstract

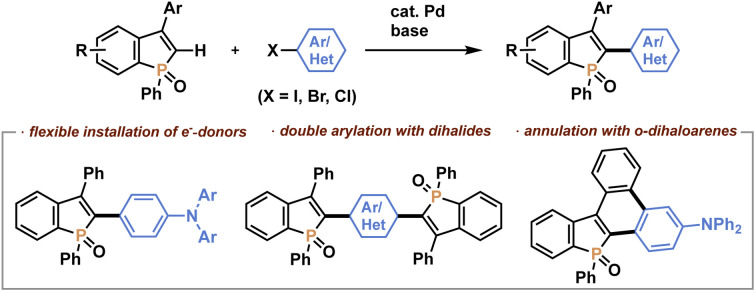

## Introduction

**Fig. 1:**
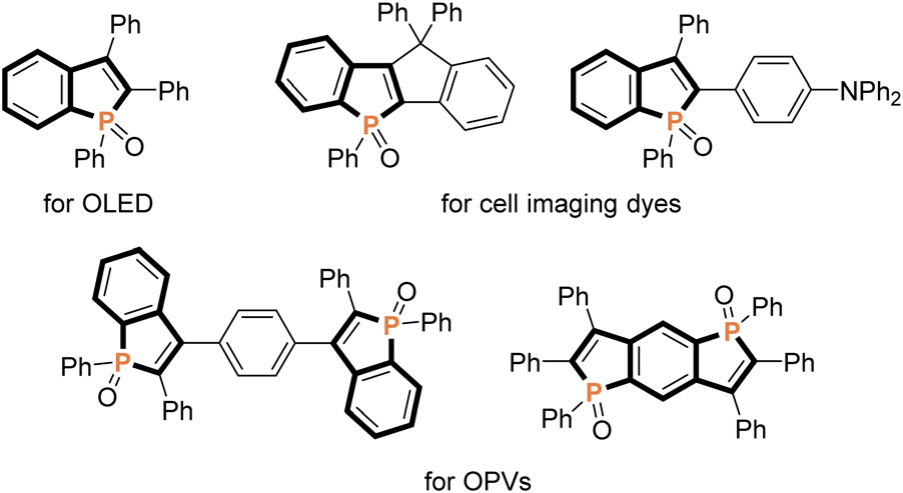
Representative examples of benzophosphole-containing functional molecules.

**Scheme 1:**
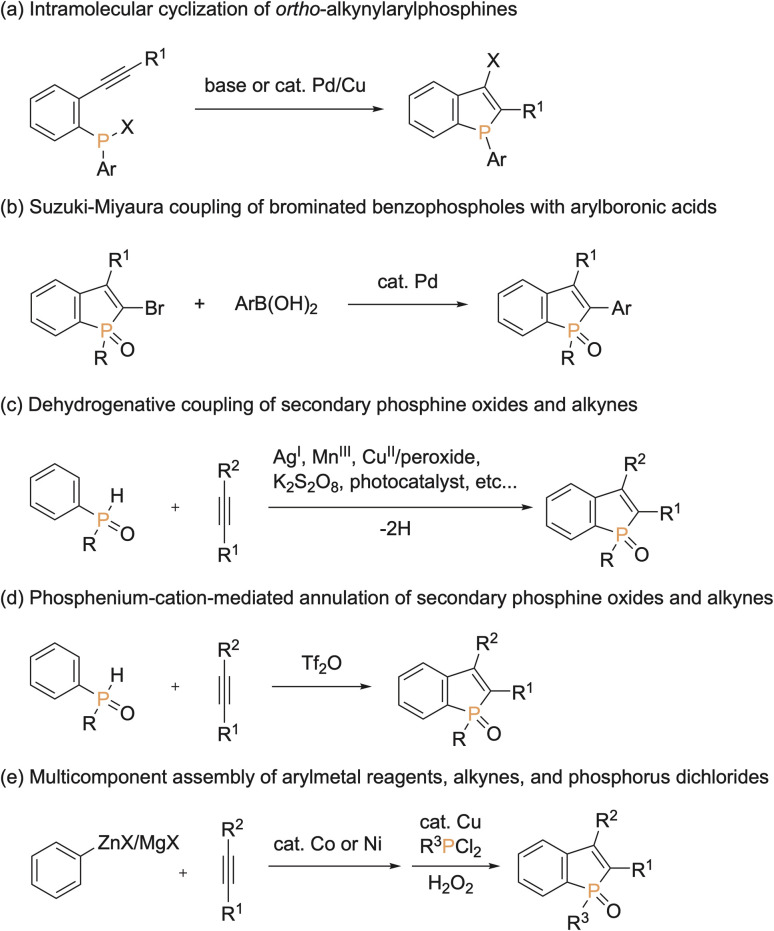
Synthetic approaches to substituted benzophospholes.

Because of unique optical, electronical, and physical properties, benzophosphole derivatives have attracted attention in the field of organic functional materials. Among well-known examples are organic light-emitting diodes (OLEDs),^1^ photovoltaics,^2^ and cell imaging dyes^3^ (Fig. 1). Accordingly, the development of synthetic strategies for the preparation of benzophospholes, particularly, multiply substituted ones, has been one of the long-standing research subjects in the synthetic community.^4^ The most reliable protocols are the cyclization–functionalisation sequence from the *ortho*-alkynylarylphosphines (Scheme 1a) and the related Suzuki–Miyaura cross-coupling of brominated benzophospholes with arylboronic acids (Scheme 1b), which can control the substituent position on the phosphole nucleus.^5^ However, the starting substrates are prepared in multiple steps, often from unstable and sensitive substrates/reagents, and thus the functional group compatibility is still problematic. Other protocols are the radical- and cation-mediated annulation reactions of secondary phosphine oxides with alkynes (Schemes 1c and d).^6,7^ The direct use of stable and readily available starting materials is the significant advantage, but the regioselectivity is difficult to control when unsymmetrical internal diaryl alkynes are employed. Recently, Yoshikai reported the modular approach from internal alkynes, chlorophosphines, and arylzincs or -magnesiums through the cobalt- or nickel-catalysed carbometalation reaction (Scheme 1e).^8^ However, also in this case, the reaction with unsymmetrical diaryl alkynes generally encounters difficulty in controlling of the regioselectivity. Thus, the rapid, concise, and selective synthesis of substituted benzophospholes, particularly, that bear two different aryl groups at the C2 and C3 positions is strongly appealing.

**Scheme 2:**
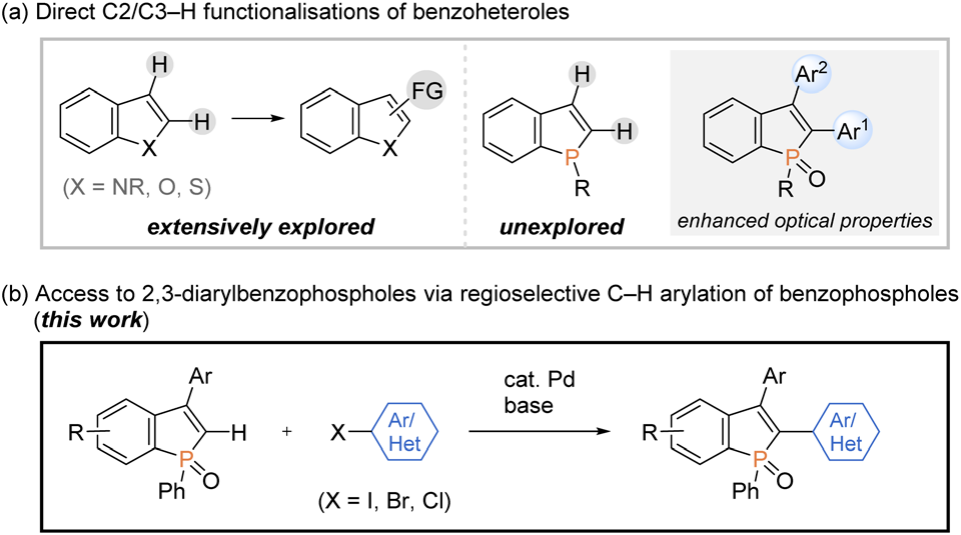
Direct C–H functionalisations of benzoheteroles.

On the other hand, transition-metal-promoted C–H functionalisation has been proven to be one of the most powerful strategies in the conversion of simple starting materials to the diverse and value-added molecules.^9^ Among them, the direct C–H arylation of benzoheteroles such as indoles, benzothiophenes, and benzofurans, to construct functional aryl-heteroaryl linkages has received tremendous attention and has made remarkable progress (Scheme 2a, left).^10^ However, the direct catalytic C–H transformation of phosphorus analogues has not been successful so far, and only a formal C–H arylation of *P*-aryl phospholes was recently reported under Cu catalysis.^11^ Given the significant optical performance of C2- and C3-diarylated benzophospholes,^1–3^ the development of C–H arylation strategy can provide a potentially more practical alternative for the rapid construction of benzophosphole-based π-electron materials (Scheme 2a, right).

Meanwhile, our research group has been interested in the development of efficient methodologies for the synthesis of benzophosphole derivatives^7,12^ and recently disclosed profitable access to the highly π-conjugated dibenzophospholes from simple biaryls *via* phosphenium dication strategy in one operation.^13^ Notably, this protocol was also productive in the reaction with 1,1-diphenylethylene to give the corresponding benzophosphole bearing a free C2–H bond, which provides accessible space for the further transformation based on C–H activation. During our continuing interest in this chemistry, we herein report a concise and general process for the synthesis of structurally useful C2,C3-diarylated benzophospholes *via* the Pd-catalysed regioselective C2–H arylation with aryl halides (Scheme 2b). Owing to the broad scope of aryl halides, the C–H arylation reaction flexibly introduces various aryl groups at the C2 position, which is complementary to reported strategies in Scheme 1 for the synthesis of C2,C3-diarylated benzophosphole derivatives. It is important to note that the identity of C2-aryl-substituent is known to largely affect the optical properties.^3^ More attractively, the double arylation with aromatic dihalides and annulation reaction with *ortho*-dihalogenated benzenes are also applicable to afford the corresponding benzophosphole-based acceptor–donor–acceptor-type molecules and highly condensed heteroacene-type molecules of potent interest in material chemistry. Additionally, we evaluated the cardinal optoelectronic properties of several new compounds. Our preliminary mechanistic studies revealed that the C–H cleavage occurred by the base-promoted deprotonation.

## Results and discussion

**Scheme 3:**
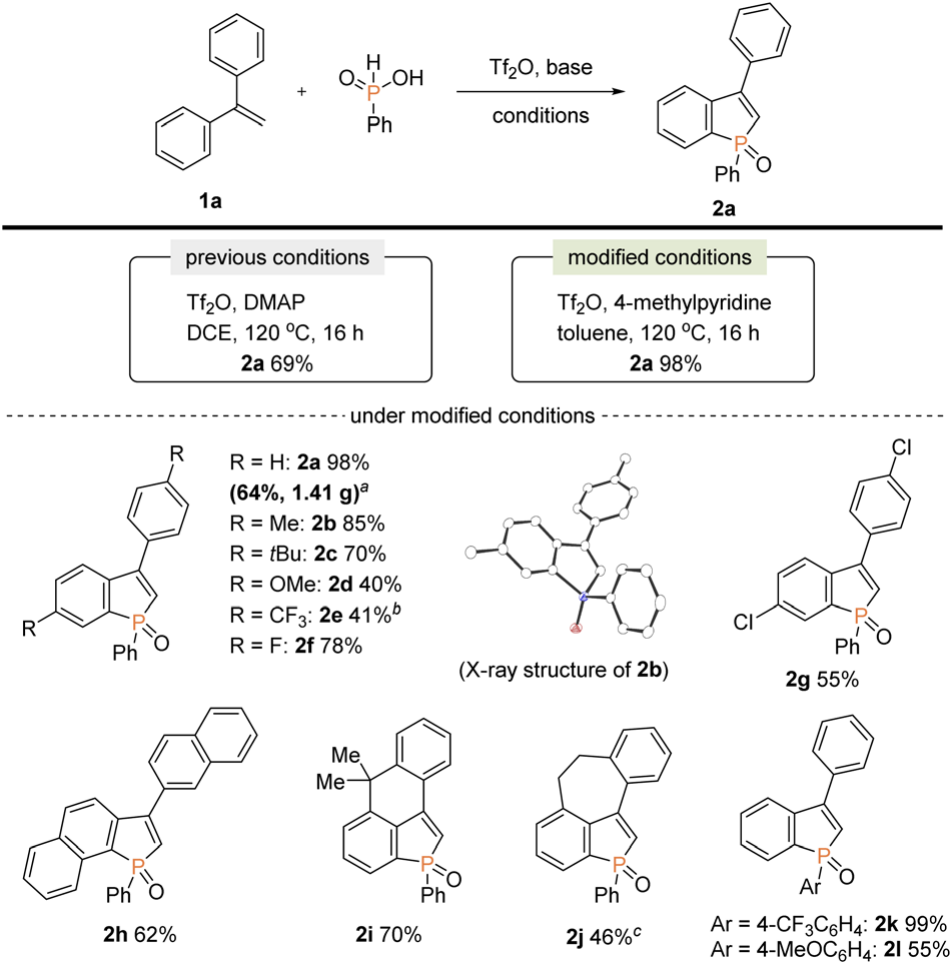
Synthesis of starting benzophospholes 2 from 1,1-diarylethylenes 1 and phenylphosphinic acids. Conditions: 1 (0.10 mmol), phenylphosphinic acid (0.20 mmol), Tf_2_O (0.48 mmol), 4-methylpyridine (0.48 mmol), toluene (1.5 mL), N_2_. Isolated yields are shown. ^*a*^On a 7.30 mmol scale for 22 h. ^*b*^Pyridine was used instead of 4-methylpyridine for 48 h. ^*c*^On a 6.0 mmol scale for 22 h.

Our attention was initially focused on preparation of the starting C2–H benzophospholes 2 from 1,1-diarylethylenes 1*via* the phosphenium dication-mediated cyclization reaction (Scheme 3). Although the 1,1-diphenylethylene 1a directly furnished the corresponding benzophosphole 2a, previous *N*,*N*-dimethyl-4-aminopyridine (DMAP)-involved conditions still suffer from a moderate yield, poor scalability, and electronic sensitivity to substrates, which hamper applications in the scalable synthesis.^13^ To address this problem, 1a was selected as model substrate, and we started re-investigation of reaction parameters including base, solvent, and reaction temperature (see the ESI for more details†). To our delight, the reaction efficiency was dramatically improved when 4-methylpyridine was used instead of DMAP in toluene at 120 °C to deliver the desired product 2a in 98% yield. The gram-scale reaction also proceeded well affording 2a in 64% yield. Under the modified conditions, several 1,1-diarylethylenes bearing electron-donating and -withdrawing groups successfully furnished the corresponding C2–H benzophospholes 2b–g in moderate to good yields. The structure of 2b was unambiguously confirmed by X-ray crystallographic analysis (CCDC 2174057†). The C–P bond formation exclusively occurred at the more electron-rich α position in the case of naphthyl substituent, and the desired 2h was obtained in 62% yield. Additionally, the structurally novel benzophosphole skeletons 2i and 2j that contain the six- and seven-membered systems, respectively, were readily accessible. Unfortunately, the heterocyclic system such as thiophene gave a complicated mixture or inseparable regiomixtures (see the ESI for details†). The substitution effect on phosphinic acid was also investigated. A reactivity trend similar to our previous work^13,14^ was observed: the electron-deficient CF_3_ group furnished 2k in a quantitative yield, whereas the electron-rich MeO-substituted one gave the corresponding 2l with decreased efficiency.

We next selected 3-phenylbenzophosphole 2a and 4-iodotoluene 3a as model substrates and commenced optimization studies on C–H arylation under Pd(OAc)_2_ catalysis (Table 1). Pleasingly, the C–H arylation occurred to form the coupling product 4aa in 66% yield even in the absence of any supporting ligands when NaO*t*Bu was used as base (entry 1). The addition of phosphine ligands gave negative or negligible results (entries 2–5, see the ESI for more details†). The choice of base is critical to the reaction: KO*t*Bu showed a comparable reactivity, while LiO*t*Bu resulted in a low conversion, and even no reaction was observed in the case of Cs_2_CO_3_ (entries 6–8). The amount of base is also important, and the conversion significantly dropped when NaO*t*Bu was reduced to 1.5 equiv. from 2 equiv. (entry 9). The additive TBAB has been demonstrated to improve the reaction efficiency in the Pd-catalysed C–H arylation under ligand-free conditions,^15^ but in our case, the conversion largely dropped (entry 10). The reaction period was greatly shortened with the assistance of microwave irradiation, and the yield was increased up to 72% (entry 11). The temperature effect was obvious, and the reaction showed a dramatically reduced efficiency at 80 °C (entry 12). The yield could be furtherly improved under more concentrated conditions (entry 13). Additionally notable is the high C2–H regioselectivity in spite of the possibility of phosphole P=O-directed C–H functionalisation of the phenyl group on phosphorus.^16^ The P=O-directed second arylation of the C2–H arylation product was actually detected in *ca.* 10% yield in the prolonged reaction periods with the conventional oil bath heating (data not shown), but the formation of such a diarylation byproduct could be avoided under the microwave irradiation. The palladium loading could be reduced to 5 mol% on a 0.20 mmol scale, and the arylation product was isolated in 79% yield (entry 14).

**Table 1:** Optimization studies for palladium-catalysed C–H arylation of benzophosphole 2a with 4-iodotoluene 3a^a^

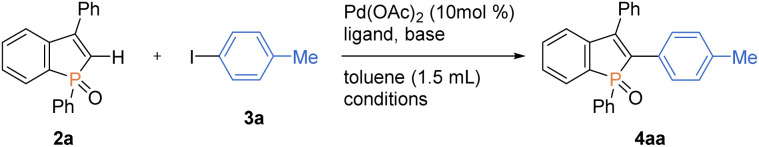				
Entry	Ligand	Base (equiv.)	Conditions	Yield of 4aa^b^ (%)
1		NaO*t*Bu (2.0)	90 °C, 16 h	66
2	SPhos	NaO*t*Bu (2.0)	90 °C, 16 h	21
3	XPhos	NaO*t*Bu (2.0)	90 °C, 16 h	19
4	PPh_2_Cy	NaO*t*Bu (2.0)	90 °C, 16 h	56
5	PPh_3_	NaO*t*Bu (2.0)	90 °C, 16 h	39
6		KO*t*Bu (2.0)	90 °C, 16 h	68
7		LiO*t*Bu (2.0)	110 °C, 16 h	21
8		Cs_2_CO_3_ (2.0)	110 °C, 16 h	0
9		NaO*t*Bu (1.5)	90 °C, 16 h	31
10		NaO*t*Bu (2.0)/TBAB (1.0)	90 °C, 16 h	22
11		NaO*t*Bu (2.0)	μW, 90 °C, 1 h	72
12		NaO*t*Bu (2.0)	μW, 80 °C, 2 h	35
13^c^		NaO*t*Bu (2.0)	μW, 90 °C, 1 h	83
14^d^		NaO*t*Bu (2.0)	μW, 90 °C, 1 h	(79)

a Conditions: 2a (0.10 mmol), 3a (0.15 mmol), Pd(OAc)_2_ (0.010 mmol), ligand (0.020 mmol), base (0.20 mmol), toluene (1.5 mL), under the indicated conditions.

b Determined by ^1^H NMR using triethylphosphate as internal standard. Isolated yield is in parentheses.

c In 1.0 mL of toluene.

d On a 0.20 mmol scale with Pd(OAc)_2_ (0.010 mmol, 5 mol%) in 2.0 mL of toluene. TBAB = tetrabutylammonium bromide.

**Scheme 4:**
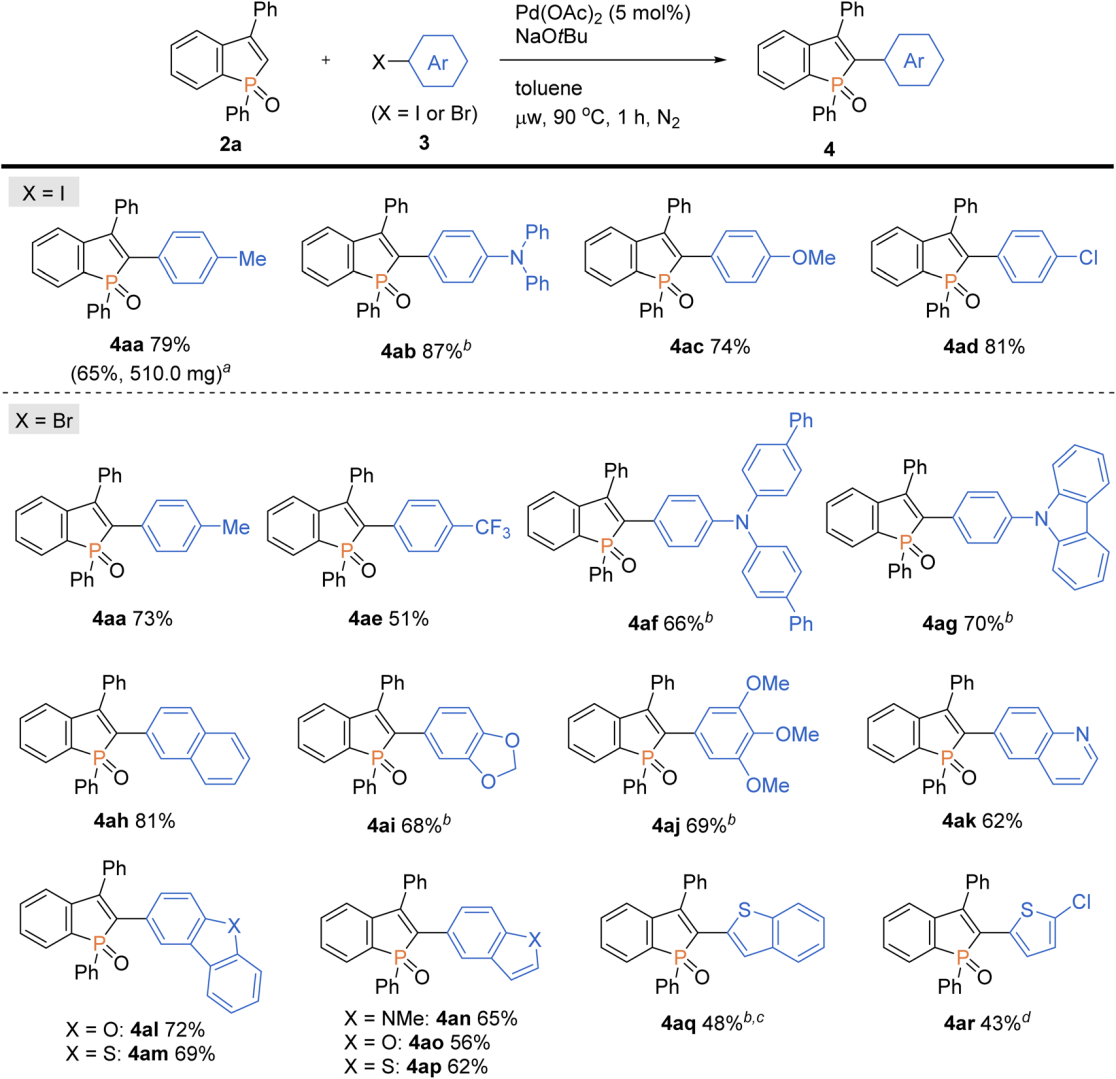
Palladium-catalysed regioselective C–H arylation of benzophosphole 2a with aryl halides 3. Conditions: 2a (0.20 mmol), 3 (0.30 mmol), Pd(OAc)_2_ (0.010 mmol), NaO*t*Bu (0.40 mmol), toluene (2.0 mL), microwave irradiation (90 °C), 1 h, N_2_. Isolated yields are shown. ^*a*^On a 2.0 mmol scale. ^*b*^At 100 °C. ^*c*^For 1.5 h. ^*d*^At 110 °C.

**Scheme 5:**
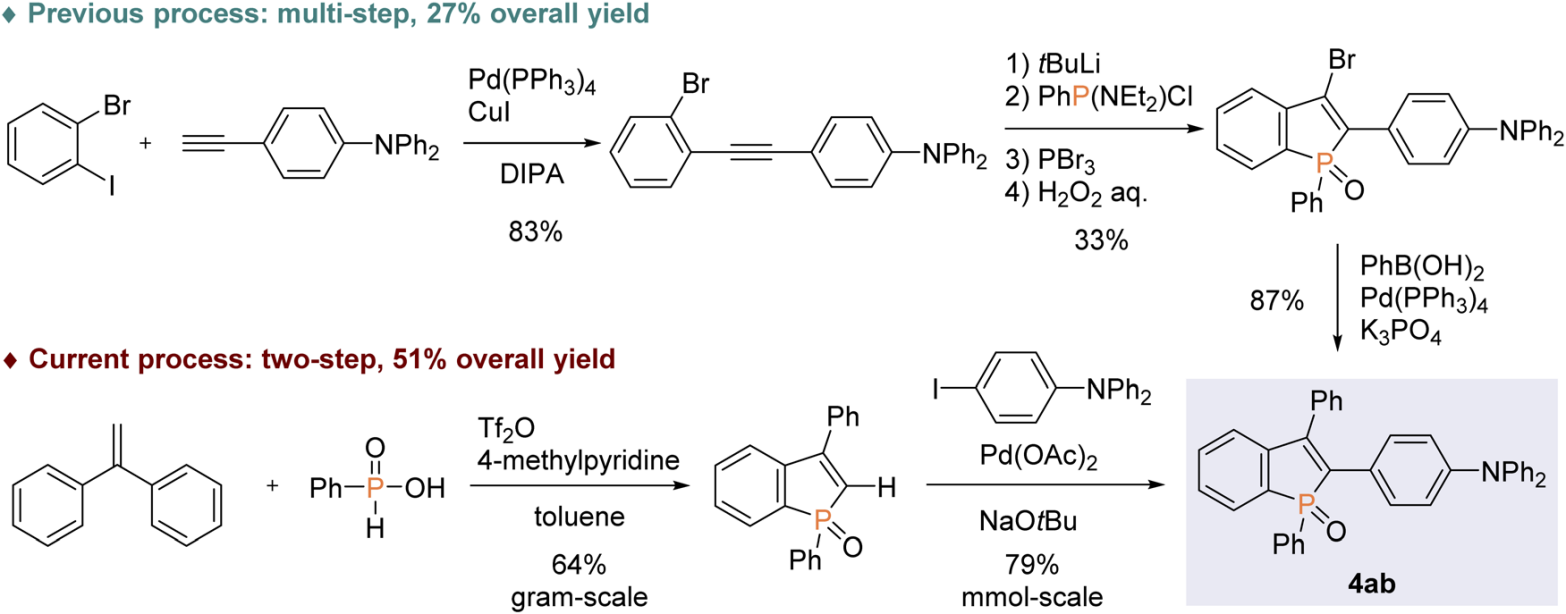
Comparison of two possible approaches to the fluorescence probe molecule 4ab.

With the optimal conditions in hand, we investigated the practicality and generality of the palladium-catalysed C–H arylation reaction (Scheme 4). The model reaction could be easily performed on a 10-fold larger scale to afford 4aa in 65% yield. The 4-iodoarenes 3 bearing both electron-donating and -withdrawing groups were good coupling partners. 4-Iodotriphenylamine 3b participated in the C–H transformation to produce the arylation product 4ab in a high yield; this molecule is particularly useful and has been used for fluorescent probe.^3*a*^ As illustrated in Scheme 5, the synthesis of such a valuable molecule 4ab was available through the Suzuki–Miyaura coupling reaction, however, the C3-brominated benzophosphole intermediate should be prepared in several steps including the Sonogashira coupling and intramolecular cyclization with sensitive reagents. In contrast, our current strategy features the short step synthesis, operational simplicity, scalability, and synthetically useful yield. As shown in Scheme 4, the MeO- and Cl-substituted iodobenzenes were also tolerated to give the coupling products 4ac and 4ad. Of note, under the standard ligand-free conditions, the reaction of 2a with 4-bromotoluene also occurred smoothly to provide the desired 4aa in a good yield. Considering better availability of aryl bromides than aryl iodides, our attempts were then moved to investigate the scope of aryl bromides in detail. The electron-withdrawing CF_3_ substituent was tolerated to deliver the arylating benzophosphole 4ae in a synthetically useful yield. Notably, at 100 °C, the aryl bromides bearing strongly electron-donating diarylamino and carbazolyl groups successfully furnished the corresponding products 4af and 4ag, which are of great potential for applications in functional materials.^17^ Furthermore, 2-bromonaphthalene 3h, 5-bromobenzodioxole 3i, and 5-bromotrimethoxybenzene 3j were also viable coupling partners to produce the donor–acceptor-type molecules 4ah–aj in good yields. Moreover, a variety of heterocyclic bromides could also be employed in the C–H arylation. For example, 6-bromoquinoline, 4-bromodibenzofuran, and 4-bromodibenzothiophene underwent the C–C coupling to give the highly π-extended frameworks (4ak–am) without any difficulties. Particularly notable is the successful application of 5-bromoindole, 5-bromobenzofuran, and 5-bromobenzothiophene that bear potentially reactive C2/C3–H bonds under the C–H activation conditions.^10^ The phosphole C2–H showed higher reactivity, and the corresponding arylated benzophospholes (4an–ap) were dominantly generated under the standard conditions. Additionally, our direct C–H arylation protocol was applicable in the reaction with 2-bromothiophenes to form the phosphole–thiophene linkages (4aq and 4ar), albeit in moderate yields.

**Scheme 6:**
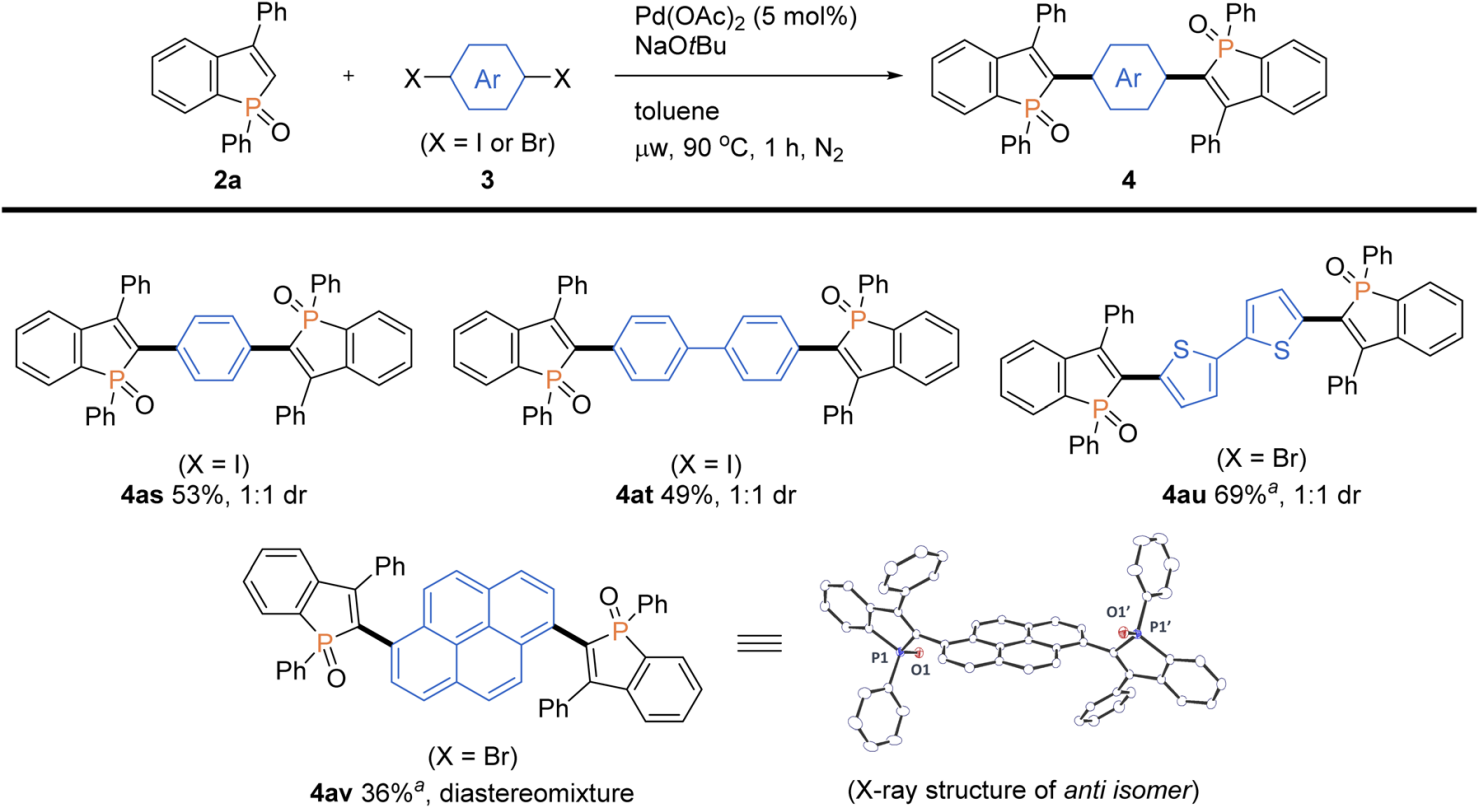
Palladium-catalysed regioselective double arylations of benzophosphole 2a with dihalides 3. Conditions: 2a (0.20 mmol), 3s–v (0.080 mmol), Pd(OAc)_2_ (0.010 mmol, 5 mol% based on 2a), NaO*t*Bu (0.40 mmol), toluene (2.0 mL), microwave irradiation (90 °C), 1 h, N_2_. Isolated yields are shown. ^*a*^At 100 °C.

More intriguingly, the optimal conditions are also applicable to the double arylations with aromatic dihalides, enabling the rapid construction of benzophosphole-based acceptor–donor–acceptor-type molecules. As shown in Scheme 6, 1,4-diiodobenzene and 4,4′-diiodobiphenyl underwent the double C–C bond formation to give the expected molecules 4as and 4at, respectively. This type of structure is of great interest for applications in OLEDs and thin-film photovoltaics.^2*a*^ As a promising electron-donor, 5,5′-dibromo-2,2′-bithiophene was also effective in the double C–C coupling to provide the highly conjugated molecules 4au in a good yield. Furthermore, as an outstanding chromophore, 1,6-dibromopyrene was coupled with two benzophosphole molecules to furnish the largely π-extended 4av, and the structure of its *anti* isomer was unambiguously confirmed by X-ray analysis (CCDC 2166424†).

**Scheme 7:**
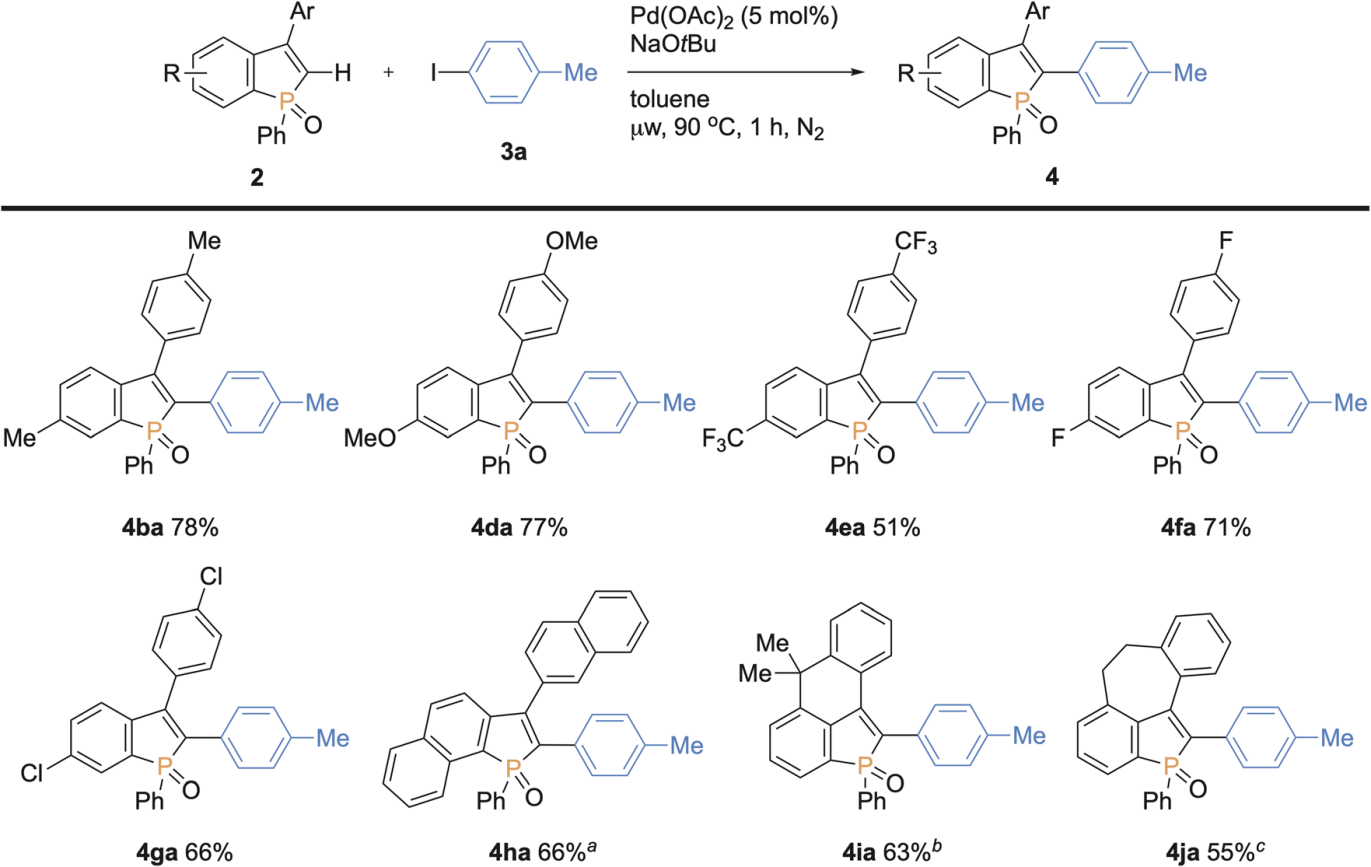
Palladium-catalysed regioselective C–H arylation of various benzophospholes 2 with 4-iodotoluene 3a. Conditions: 2 (0.20 mmol), 3a (0.30 mmol), Pd(OAc)_2_ (0.010 mmol), NaO*t*Bu (0.40 mmol), toluene (2.0 mL), microwave irradiation (90 °C), 1 h, N_2_. Isolated yields are shown. ^*a*^KO*t*Bu was used instead of NaO*t*Bu at 110 °C. ^*b*^At 110 °C for 1.5 h. ^*c*^KO*t*Bu was used instead of NaO*t*Bu at 100 °C.

We next examined the scope of benzophospholes with 4-iodotoluene 3a as the coupling partner (Scheme 7). Various C2–H free benzophospholes were smoothly arylated under our standard conditions *via* the C–H bond cleavage, thus easily accessing the structurally useful C2,C3-diarylated benzophospholes. The electron-donating (Me and OMe) and electron-withdrawing (CF_3_ and F) substituents were well tolerated to deliver the functionalized benzophospholes 4ba–fa in acceptable to good yields. The chloro-substituted benzophosphole 2g also furnished the coupling product with the Ar–Cl moiety left intact. Additionally, the more condensed naphthophosphole 2h could also be functionalized under the modified conditions using KO*t*Bu in place of NaO*t*Bu. It should be noted that the C2-arylated naphthophospholes also show unique optical properties, and our synthetic method provides a rapid access to such interesting skeletons.^5*f*,18^ Moreover, the benzophospholes bearing six- and seven-membered cyclic systems underwent the C–C coupling to deliver the multi-ring fused products 4ia–ja in acceptable yields. Thus, additional salient feature of our synthetic platform is the controllable introduction of two different aryl substituents at the C2 and C3 positions of benzophospholes.

**Scheme 8:**
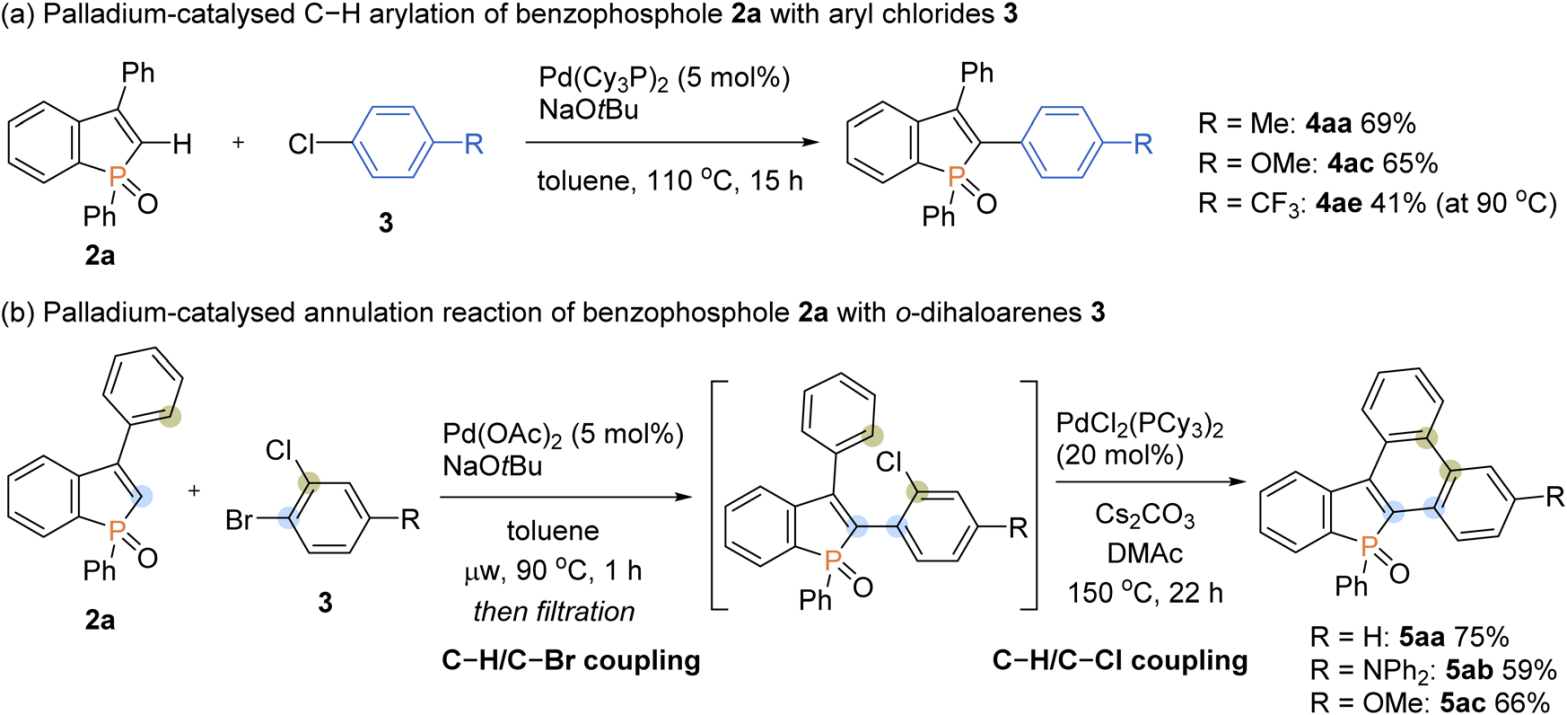
Palladium-catalysed regioselective C–H arylation of benzophosphole 2a with aryl chlorides 3 and application to annulation reactions with *o*-dihaloarenes 3.

To further expand the generality of our protocol, we then focused on the less reactive aryl chlorides for the C–H arylation reaction (Scheme 8a). However, under standard ligand-free conditions, no target product was detected. After extensive screening of palladium catalysts and ligands (see the ESI for details†), we were pleased to find that the Pd(Cy_3_P)_2_ complex was optimal, and the arylating product 4aa was formed in a good yield. The Pd(Cy_3_P)_2_ catalyst was effective for both the electron-rich and -deficient aryl chlorides to afford the corresponding products in synthetically useful yields (4ac and 4ae).

Taking advantage of distinct reactivity of Ar–Br and Ar–Cl moieties, we attempted the annulation reaction with bromochloroarenes *via* sequential C–H/C–X coupling (Scheme 8b). The 2-bromochloroarene 3 and benzophosphole 2a were subjected to the ligand-free Pd(OAc)_2_-catalysed C–H arylation conditions, which was followed by PdCl_2_(PCy_3_)_2_-catalysed intramolecular C–H arylation of phenyl ring with the C–Cl moiety to furnish the highly condensed framework 5aa in a good overall yield. The donor–acceptor systems 5ab and 5ac were also readily prepared with acceptable efficiencies.

**Scheme 9:**
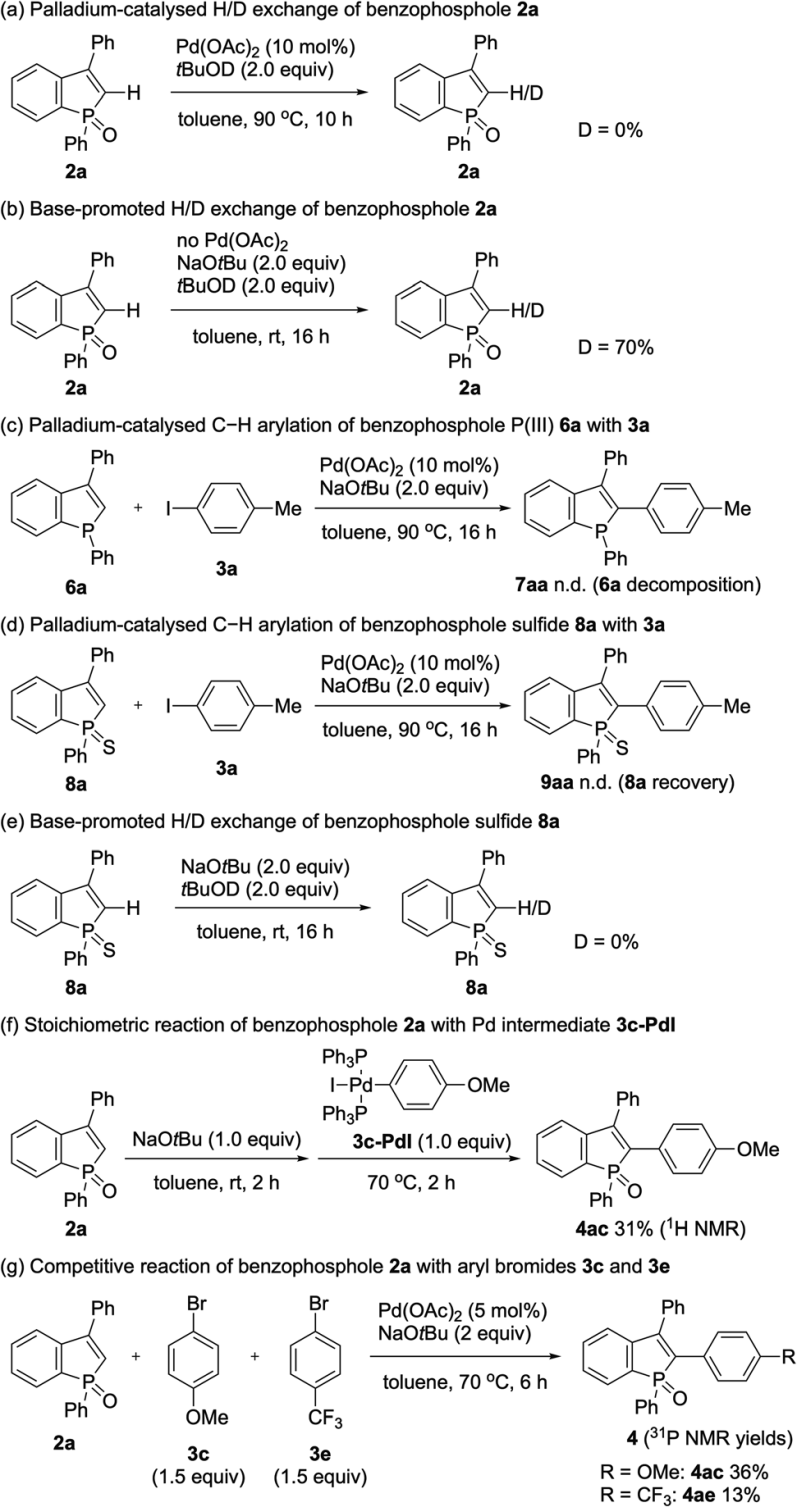
Mechanistic studies.

**Fig. 2:**
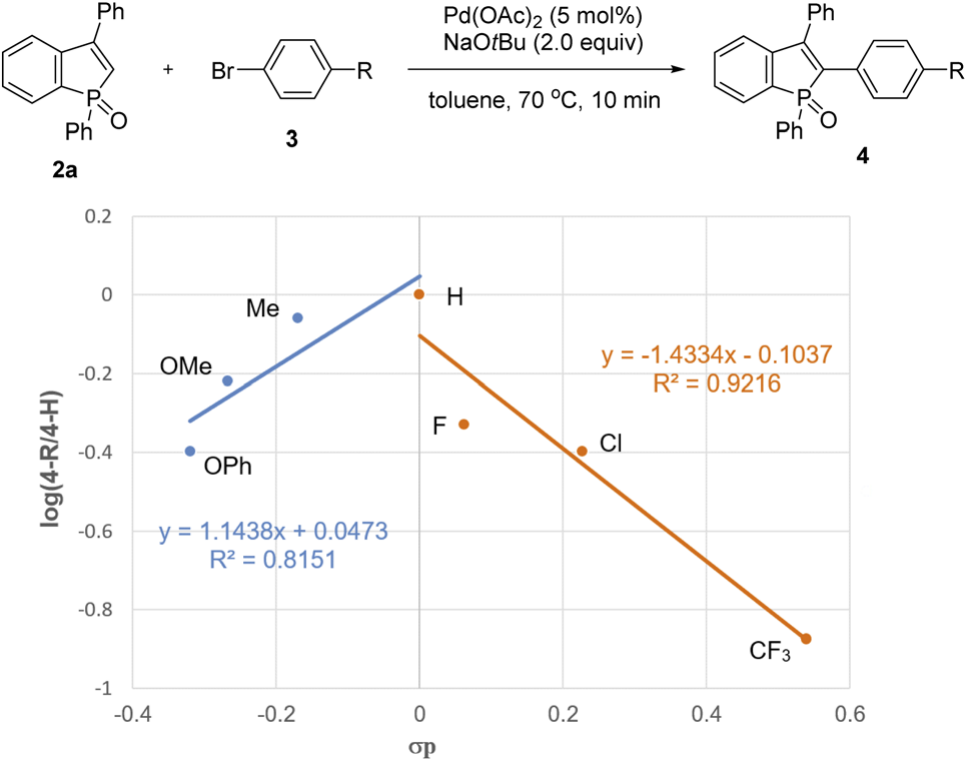
Hammett plot with *para*-substituted aryl bromides 3.

To gain insight into the reaction pathway, we performed several control experiments (Scheme 9). The C–H bond cleavage step was first investigated by H/D scrambling experiments with benzophosphole 2a and *t*BuOD in the absence of the aryl halide coupling partner. The H/D exchange of 2a was not observed at all in the presence of Pd(OAc)_2_ alone (Scheme 9a). In contrast, 70% deuterium incorporation at the C2 position was detected when 2a was treated with 2 equiv. of NaO*t*Bu at room temperature even in the absence of Pd(OAc)_2_ (Scheme 9b). These results apparently indicate that the C–H bond cleavage of benzophosphole can occur by deprotonation with basic NaO*t*Bu.^19^ To investigate the effect of phosphorus moiety in the reaction, we tested the corresponding P(iii) benzophosphole 6a and benzophosphole sulfide 8a with 4-iodotoluene (3a) under the standard conditions (Schemes 9c and d, respectively). There was no detectable arylation product in both cases; 6a underwent decomposition because of its stability issue, while no reaction occurred with 8a probably due to the lower acidity of the C2–H bond.^20^ We actually did not observe any deuterium incorporation when 8a was treated with NaO*t*Bu and *t*BuOD (Scheme 9e). Although we could not completely preclude the directing effect of P=O group, the acidity of C2–H bond seems to be critical in the regioselective arylation. Additionally, when we independently prepared the palladium complex 3c-PdI^21^ and subjected it to a mixture of 2a and NaO*t*Bu, the arylation product 4ac was indeed formed in 31% ^1^H NMR yield, indicating that the reaction proceeds *via* a Pd(0)/Pd(ii) catalytic cycle (Scheme 9f). We also observed an inverted V-shaped Hammett plot by the reaction of 1a with several *para*-substituted aryl bromides 3 (Fig. 2): a positive slope of *ρ* = 1.14 was obtained from the electron-donating groups, whereas the electron-withdrawing groups resulted in a negatively sharper slope of *ρ* = −1.43 (see the ESI for more details†). Thus, the rate-limiting step would change, dependent on the electronic nature of the substituent on the aryl bromide. Further competitive experiment of substrate 2a with aryl bromides 3c and 3e revealed that the more electron-rich 3c showed higher reactivity than the electron-deficient 3e (Scheme 9g).

**Scheme 10:**
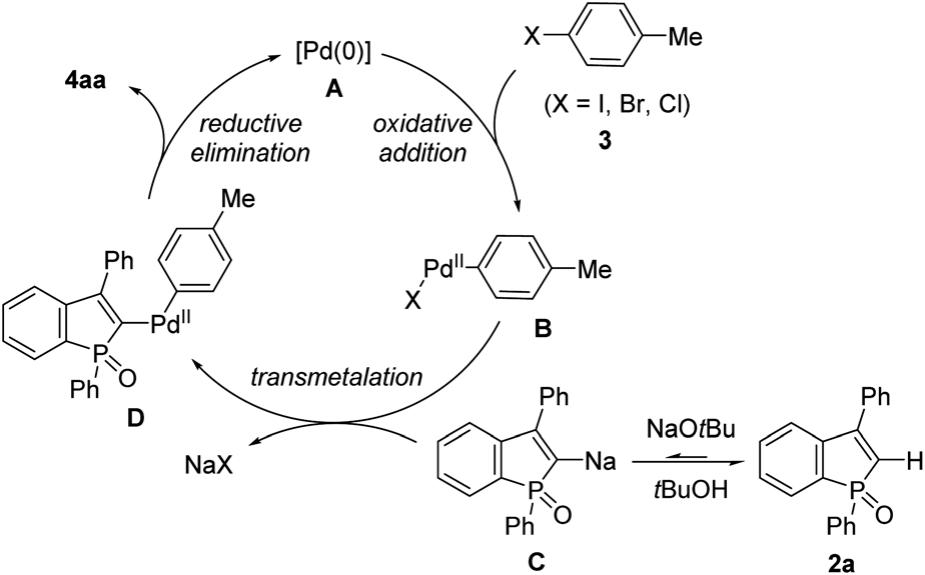
A plausible catalytic cycle.

On the basis of the aforementioned outcomes, our proposed reaction mechanism of 2a with 3 is illustrated in Scheme 10. Oxidative addition of Pd(0) A to the aryl electrophile 3 results in the formation of Ar–Pd(ii)–X complex B. A dynamic deprotonation/metalation of benzophosphole with NaO*t*Bu (2a ⇋ C) is followed by transmetalation to Pd, giving the Ar–Pd(ii)–phosphole intermediate D.^22^ Subsequent reductive elimination forms the arylated benzophosphole 4aa with regeneration of the starting Pd(0) species A to complete the catalytic cycle. The result in Scheme 9g suggests that the oxidative addition step is somewhat influential, but the reductive elimination is a more predominant step in the product formation.

**Fig. 3:**
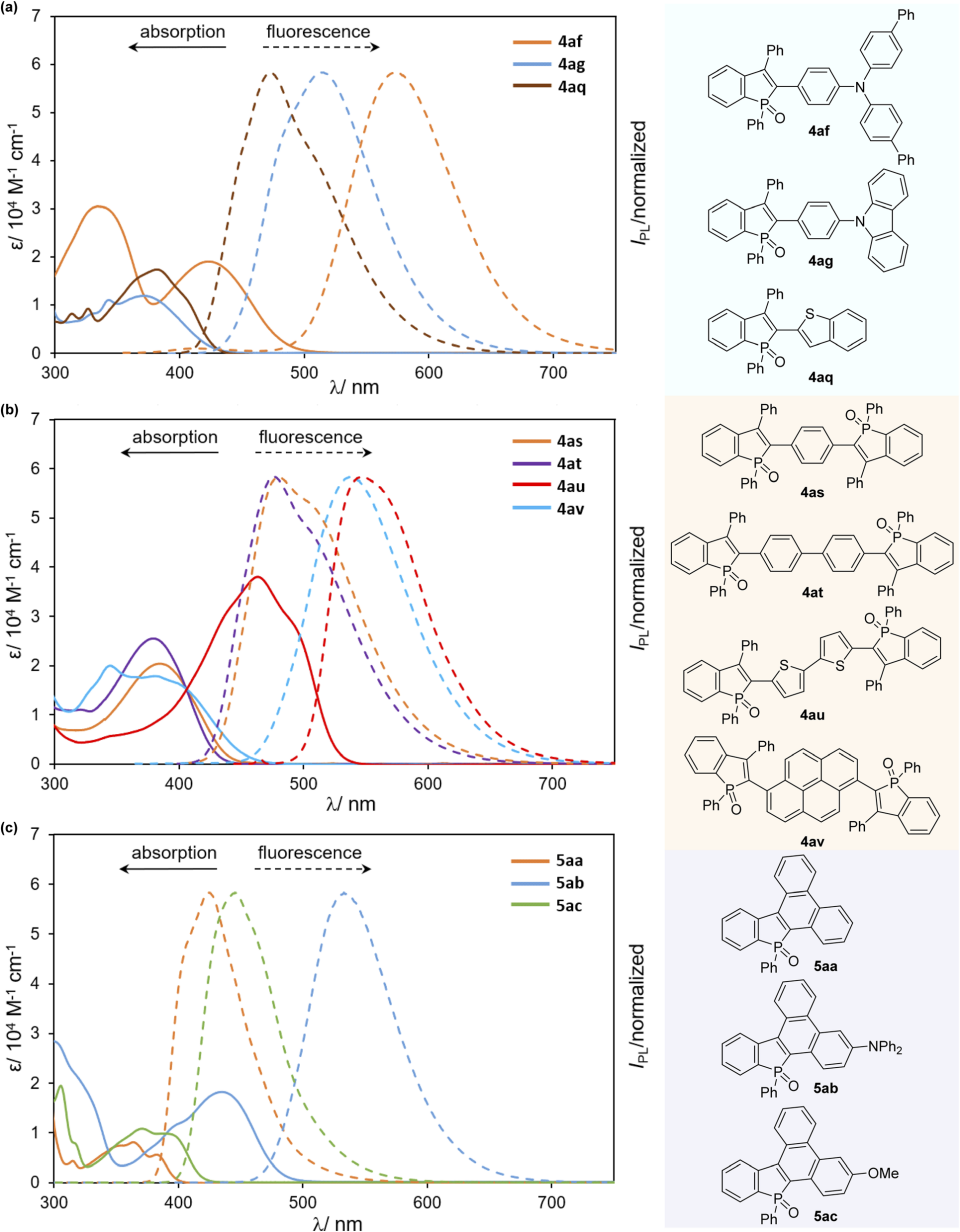
UV-Vis absorption (solid line) and fluorescence spectra (dashed line) of (a) 4af–ag, and 4aq, (b) 4as–av, and (c) 5aa–ac in CH_2_Cl_2_ (*c* = 1.0 × 10^−5^ M).

**Fig. 4:**
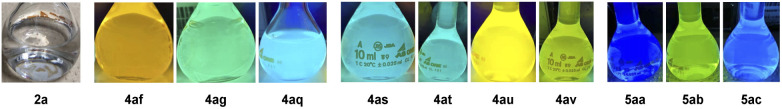
Fluorescence images of some compounds in CH_2_Cl_2_ (*c* = 1.0 × 10^−5^ M) under UV irradiation (365 nm).

We finally examined the optical properties of several newly synthesized compounds in CH_2_Cl_2_ solution. UV/Vis absorption and fluorescence spectra of selected compounds 4 and 5 are shown in Fig. 3, and the absorption/emission properties (*λ*_abs_/*λ*_em_) and fluorescence quantum yields (*Φ*_F_) are summarized in Table 2. Compared with the starting compound 2a, all arylated benzophosphole derivatives were fluorescent in solution (Fig. 4, 2a*vs.* selected compounds). Most compounds gave a relatively narrow range of their longest wavelength absorption maxima (370–395 nm), whereas the electron-donating diarylamino-substituted 4af and 5ab, and bithiophenyl-derived 4au showed large bathochromic shifts of their *λ*_abs_ values (533–572 nm) with higher molar extinction coefficients (*ε*) and fluorescence in the green to yellow colour regions. Additionally, 4ag and 4av also exhibited intense fluorescence with broadened emission bands in the visible region. Although the quantum yield was moderate, 4af showed absorption and emission maxima at the even longer wavelength regions with a larger molar extinction coefficient relative to a similar framework 4ab. It is also noteworthy that, in comparison with a series of biaryl-type compounds 4, the more condensed 5 exhibited distinctly smaller Stokes shifts with good to excellent quantum yields, probably because of their highly rigid structures.

**Table 2:** Optical properties of selected compounds 4 and 5 in CH_2_Cl_2_

4/5	*λ*_abs_ (nm) (*ε* (10^4^ M^−1^ cm^−1^))	*λ*_em_ a (nm)	*Φ*_F_ b (%)	Δ*ν*^c^ (cm^−1^)
4ab^d^	415 (1.73)	565	90	6397
4af	334 (3.05), 422 (1.90)^e^	572	48	6214
4ag	294 (1.87), 348 (1.04), 370 (1.19)^e^	512	83	7496
4aq	380 (1.73)	471	20	5085
4as	388 (2.03)	481, 505	23	4983
4at	380 (2.55)	479, 499	39	5439
4au	438 (3.08), 465 (3.78), 490 (2.89)^e^	548	25	2160
4av	344 (2.0), 385 (1.77)^e^	540	32	7455
5aa	297 (1.97), 364 (0.81), 382 (0.56)^e^	425	56	2649
5ab	278 (3.19), 304 (2.78), 438 (1.81)^e^	533	87	4069
5ac	306 (0.19), 370 (0.11), 395 (0.10)^e^	446	95	2894

a Excited at *λ*_abs_.

b Absolute fluorescence quantum yields.

c Stokes shifts.

d The optical data of 4ab in CH_2_Cl_2_ was reported by Yamaguchi *et al.* in ref. 3*a*.

e Absorption maxima at the longest wavelength.

We also investigated the electrochemical properties of the aforementioned compounds 4 and 5 by cyclic voltammetry (CV) and differential pulse voltammetry (DPV) in MeCN with tetrabutylammonium hexafluorophosphate (Bu_4_NPF_6_) as an electrolyte *versus* ferrocene/ferrocenium ion (Fc/Fc^+^) (Fig. S2–S12†), and their HOMO and LUMO levels were estimated according to the first oxidation potentials and the optical band gaps (*E*^opt^_g_). The data is summarized in Table 3. The CV of 4ab, 4af, 4au, and 5ab showed reversible first oxidation waves, and their *E*^1/2^_ox_ values are significantly shifted in a negative direction. Notably, compared with 4ab, identical HOMO levels and even lower LUMO levels were estimated for the 4af and 5ab, which may suggest their larger intramolecular charge transfer abilities. Density functional theory (DFT) calculations at the PBE0/6-31+G(d) level of theory were performed for 4ab,^3*a*^4at, and 4av, and their HOMO levels were estimated as −5.35 eV, −5.91 eV, and −5.48 eV, respectively (Fig. S14 and S15†). These values are in good agreement with those obtained from the CV and DPV experiments.

**Table 3:** Absorption wavelengths, HOMO–LUMO energy gaps, and cyclic (differential pulse) voltammogram data of selected compounds 4 and 5

4/5	*λ* ^abs^_onset_ a (nm)	*E* ^opt^_g_ b (eV)	*E* ^1/2^_ox_ c (V)	*E*_HOMO_ d (eV)	*E*_LUMO_ e (eV)
4ab	441^f^	2.81	0.535	−5.34	−2.53
4af	485	2.56	0.509	−5.31	−2.75
4ag	424	2.92	0.87	−5.67	−2.75
4aq	428	2.90	1.06	−5.86	−2.96
4as	440	2.82	1.18	−5.98	−3.16
4at	429	2.89	1.16	−5.96	−3.07
4au	525	2.36	0.714	−5.51	−3.15
4av	455	2.73	0.89	−5.69	−2.96
5aa	397	3.12	1.32	−6.12	−3.0
5ab	481	2.58	0.553	−5.35	−2.77
5ac	418	2.96	1.03	−5.83	−2.87

a Measured in CH_2_Cl_2_.

b Determined from the onset of the absorption spectra.

c Performed in MeCN in the presence of Bu_4_NPF_6_. *ν* = 0.1 V s^−1^ (4ag), 0.05 V s^−1^ (4af, 4aq, 4as–av, and 5aa), 0.03 V s^−1^ (4at, 5ab–ac). Values determined by CV (4ab, 4af, 4au, and 5ab) or DPV (4ag, 4aq, 4as, 4at, 4av, 5aa, and 5ac), *versus* Fc/Fc^+^.

d The approximation for Fc/Fc^+^ level is −4.8 eV *versus* vacuum: *E*_HOMO_ = −4.8 − *E*^1/2^_ox_.

e Estimated from *E*_HOMO_ and *E*^opt^_g_: *E*_LUMO_ = *E*_HOMO_ + *E*^opt^_g_.

f The value was cited from ref. 3*a*.

## Conclusions

In conclusion, we have developed the first example of palladium-catalysed regioselective C2–H arylation of benzophospholes with aryl halides. The reaction proceeds well with various aryl iodides, bromides, and even more challenging aryl chlorides, thus providing a modular synthetic platform for the construction of C2,C3-diarylated benzophospholes. Moreover, the double arylations with aromatic dihalides and annulation with *ortho*-dihalogenated arenes are also achieved to give the corresponding π-extended molecules of potent interest in material chemistry. Additionally, preliminary investigations of cardinal optoelectronic properties of some newly synthesized benzophospholes are also conducted. We anticipate that this strategy will find wide applications in the design and synthesis of benzophosphole-based materials and provide an entry point to other C–H functionalisations of benzophosphole derivatives.

## Data availability

All experimental procedures and spectroscopic data can be found in the ESI.†

## Author contributions

S. X. and K. N. performed optimization studies and scope investigations of C–H arylation. K. S. investigated the synthesis of starting benzophospholes. S. X. and K. H. prepared the manuscript. The project was supervised by K. H. and M. M. All the authors discussed the results and commented on the manuscript.

## Conflicts of interest

There are no conflicts to declare.

## Supplementary Material

SC-013-D2SC04311D-s001

SC-013-D2SC04311D-s002
