# Supplementary material for: Palladium-catalysed C–H arylation of benzophospholes with aryl halides
Source: Chem Sci. 2022 Aug 30;13(36):10950–60. doi: 10.1039/d2sc04311d (PMC9491221; doi:10.1039/d2sc04311d)
Supplement: SC-013-D2SC04311D-s002 [file SC-013-D2SC04311D-s002.pdf]

Electronic Supplementary Material (ESI) for Chemical Science.
This journal is © The Royal Society of Chemistry 2022

# Supplementary Information

**Palladium-catalysed C–H arylation of benzophospholes with aryl halides**

Shibo Xu,† Kazutoshi Nishimura,‡ Kosuke Saito,‡ Koji Hirano*,†,‡ and Masahiro Miura*,†

†*Innovative Catalysis Science Division, Institute for Open and Transdisciplinary Research Initiatives (ICS-OTRI), Osaka University, Suita, Osaka 565-0871, Japan*

‡*Department of Applied Chemistry, Graduate School of Engineering, Osaka University, Suita, Osaka 565-0871, Japan*

*k_hirano@chem.eng.osaka-u.ac.jp; miura@chem.eng.osaka-u.ac.jp*

## Contents


| | |
|---|---|
| **Instrumentation and Chemicals** | S2–S3 |
| **Experimental Procedures** | S4–S6 |
| **Detailed Optimization Studies** | S7–S11 |
| **Hammett Studies** | S12 |
| **Cyclic Voltammetry and Differential Pulse Voltammetry** | S13–S18 |
| **Absorption and Fluorescence Spectra** | S19 |
| **Theoretical Calculations** | S20–S21 |
| **X-Ray Analysis** | S22–S23 |
| **Characterization Data for Products** | S24–S141 |
| **References** | S142 |


## Instrumentation and Chemicals

$^1H$, $^{13}C\{^1H\}$, $^{19}F\{^1H\}$, and $^{31}P\{^1H\}$ NMR spectra were recorded at 400 MHz, 100 MHz, 376 MHz, and 162 MHz, respectively, for $CDCl_3$ solutions. HRMS data were obtained by APCI using TOF. GC analysis was carried out using a silicon OV-17 column (i. d. 2.6 mm x 1.5 m) or CBP capillary column (i. d. 0.5 mm x 25 m). TLC analyses were performed on commercial glass plates bearing 0.25-mm layer of Merck Silica gel $60F_{254}$. Silica gel (Wakosil C-200, Wako Pure Chemical Co.) was used for column chromatography. Flash silica gel column chromatography was performed by Isolera One (Biotage). Gel permeation chromatography (GPC) was performed by LC-20AR (pump, SHIMADZU, 7.5 mL/min $CHCl_3$ or EtOAc) and SPD-20A (UV detector, SHIMADZU, 254 nm) with two in-line YMC-GPC T2000 (20 x 600 mm, particle size: 10 μm) (preparative columns, YMC). UV-vis spectra were acquired with JASCO V-750 spectrometer. Photoluminescence spectra and quantum yield measurements were conducted with JASCO FP-8500 spectrometer equipped with an integration sphere system. Cyclic voltammograms and differential pulse voltammograms were recorded on ALS Electrochemical Analyzer Model 600E equipped with SVC-3 Voltammetry cell. Counter and working electrodes were made of Pt, and the reference electrode was $Ag/Ag^+$. The measurements were conducted in MeCN solvent containing tetrabutylammonium hexafluorophosphate as a supporting electrolyte at an indicated scan rate. All the potentials were calibrated with the standard ferrocene/ferrocenium ($Fc/Fc^+$) redox couple measured in identical conditions. Microwave irradiation

was conducted with Initiator+ (Biotage), and the reaction temperature was measured by an internal probe. The crystal measurement was performed with XtaLAB Synergy-S/Cu (Rigaku).

Unless otherwise noted, materials obtained from commercial suppliers were used without further purification. Toluene was dried on a Glass Contour Solvent dispensing system (Nikko Hansen & Co., Ltd.) prior to use. The 1,1-diarylethylene (**1a**) was commercially available from TCI, and others **1b-d** and **1f-j** were prepared from the commercially available corresponding benzophenones by the Wittig reaction with methyltriphenylphosphonium bromide. The **1e** was prepared according to the literature method.[S1] 4-Bromo-3-chloro-*N,N*-diphenylaniline was synthesized from 1-bromo-2-chloro-4-iodobenzene and diphenylamine by the Buchwald-Hartwig amination reaction.[S2] Unless otherwise noted, all reaction were performed under nitrogen atmosphere.

## Experimental Procedures

### 1. Procedures for the synthesis of benzophosphole 2 (Scheme 3)

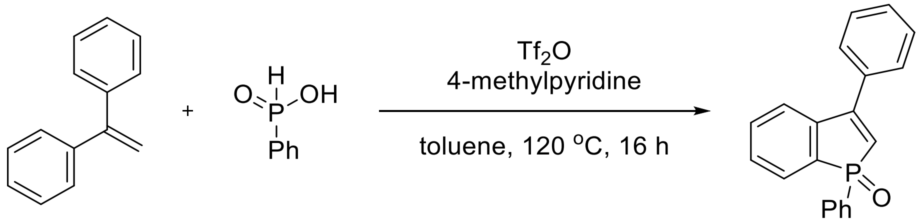


**1a** **2a**

<u>A 0.1 mmol scale reaction of **1a**:</u> In a Schlenk tube with pressure resistance, phenylphosphinic acid (0.20 mmol, 28.4 mg) was dissolved in toluene (1.5 mL), and 1,1-diphenylethylene (**1a**, 0.10 mmol, 18.0 mg) and 4-methylpyridine (0.48 mmol, 44.7 mg) were subsequently added under $N_2$. $Tf_2O$ (0.48 mmol, 80 $\mu$L) was then added, and the mixture was heated at 120 ˚C in an oil bath for 16 h. After cooling, the resulting mixture was quenched with aqueous HCl (1 M, 1 mL) and extracted with $CHCl_3$ (3 x 10 mL). The combined organic layer was dried over $Na_2SO_4$ and concentrated in vacuo. The residue was purified by flash silica gel column chromatography with hexane/EtOAc (1:2, v/v) to give the desired product **2a** (98%, 29.6 mg).

<u>A 7.3 mmol scale reaction of **1a**:</u> In a Schlenk tube with pressure resistance, phenylphosphinic acid (14.6 mmol, 2.073 g) was dissolved in toluene (15 mL), and 1,1-diphenylethylene (**1a**, 7.3 mmol, 1.314 g) and 4-methylpyridine (35.04 mmol, 3.263 g) were subsequently added under $N_2$. The tube was cooled to 0 °C with an ice bath, and $Tf_2O$ (35.04 mmol, 5.8 mL) was added. The mixture was heated at 120 ˚C in an oil bath for 22 h. After cooling, the resulting mixture was quenched with aqueous HCl (1 M, 10 mL) and extracted with $CHCl_3$ (3 x 25 mL). The combined organic layer was dried over $Na_2SO_4$ and concentrated in vacuo. The residue was purified by silica gel column chromatography with hexane/EtOAc (1:2, v/v) and GPC ($CHCl_3$) subsequently to give the desired product **2a** (64%, 1.41 g).

### 2. Procedures for palladium-catalysed C–H arylation of benzophospholes

### Procedure for the reaction with aryl iodides or bromides (Scheme 4)

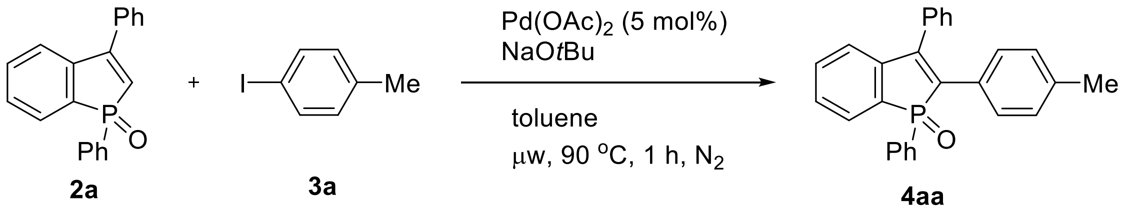


<u>A 0.2 mmol scale reaction of **2a**:</u> To a 10 mL microwave vessel were added **2a** (0.20 mmol, 60.4 mg), 4-iodotoluene (**3a**, 0.30 mmol, 65.4 mg), and $Pd(OAc)_2$ (0.010 mmol, 2.3 mg). The vessel was moved to a glovebox filled with $N_2$, and NaO*t*Bu (0.40 mmol, 38.4 mg) was added. The vessel was sealed and taken out from the glovebox. Toluene (2.0 mL) was then added by syringe, and the reaction mixture was irradiated under microwave reactor conditions at 90 °C for 1 hour. The reaction was allowed to cool to room temperature, and the mixture was filtered through a short plug of activated alumina (EtOAc as eluent) and concentrated in vacuo. The residue was purified by silica gel column chromatography with hexane/ethyl acetate (1:2, v/v) as eluent to give the desired product **4aa** (79%, 61.9 mg).

<u>A 2.0 mmol scale reaction of **2a**:</u> To a 30 mL microwave vessel were added **2a** (2.0 mmol, 604.0 mg), 4-iodotoluene (**3a**, 3.0 mmol, 654.0 mg), and $Pd(OAc)_2$ (0.10 mmol, 22.5 mg). The vessel was moved to a glovebox filled with $N_2$, and NaO*t*Bu (4.0 mmol, 384.0 mg) was added. The vessel was sealed and taken out from the glovebox. Toluene (15 mL) was then added by syringe, and the reaction mixture was irradiated under microwave reactor conditions at 90 °C for 1 hour. The reaction was allowed to cool to room temperature, and the mixture was filtered through a short plug of activated alumina (EtOAc as eluent) and concentrated in vacuo. The residue was purified by silica gel column chromatography with hexane/ethyl acetate (1:2, v/v) as eluent to give the desired product **4aa** (65%, 510.0 mg).

**Procedure for the reaction with aryl chlorides (Scheme 8a)**

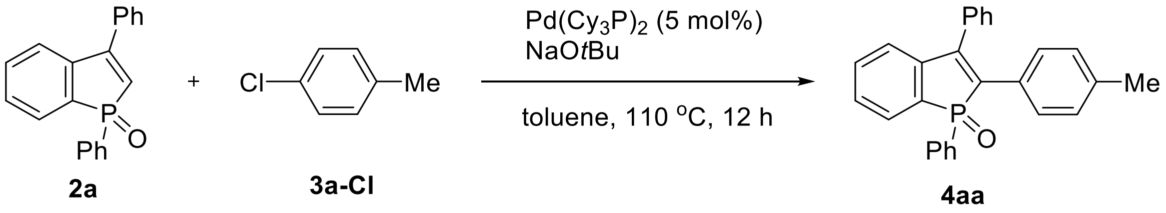


<u>A 0.1 mmol scale reaction of **2a**:</u> To a 10 mL microwave vessel were added **2a** (0.10 mmol, 30.2 mg) and 4-chlorotoluene (**3a-Cl**, 0.30 mmol, 38.0 mg). The vessel was moved to a glovebox filled with $N_2$, and $Pd(Cy_3P)_2$ (0.0050 mmol, 3.3 mg) and NaO*t*Bu (0.20 mmol, 19.2 mg) were added. The vessel was sealed and taken out from the glovebox. Toluene (1.0 mL) was then added by

syringe, and the reaction mixture was stirred at 110 °C (oil bath) for 12 hour. The reaction was allowed to cool to room temperature, and the mixture was filtered through a short plug of activated alumina (EtOAc as eluent) and concentrated in vacuo. The residue was purified by silica gel column chromatography with hexane/ethyl acetate (1:2, v/v) as eluent to give the desired product **4aa** (69%, 27.1 mg).

**Procedure for the synthesis of 5 (Scheme 8b)**

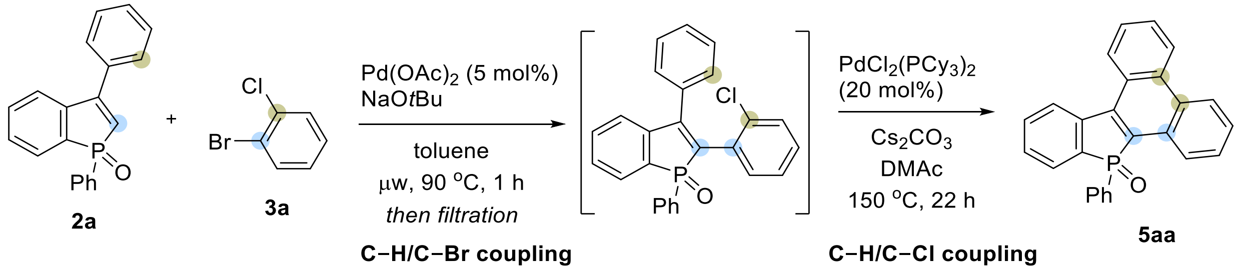


Synthesis of **5aa**: To a 10 mL microwave vessel were added **2a** (0.20 mmol, 60.4 mg), 1-bromo-2-chlorobenzene (**3**, 0.30 mmol, 57.4 mg), and $Pd(OAc)_2$ (0.010 mmol, 2.3 mg). The vessel was moved to a glovebox filled with $N_2$, and NaO*t*Bu (0.40 mmol, 38.4 mg) was added. The vessel was sealed and taken out from the glovebox. Toluene (2.0 mL) was then added by syringe, and the reaction mixture was irradiated under microwave reactor conditions at 90 °C for 1 hour. The reaction was allowed to cool to room temperature, and the mixture was filtered through a short plug of activated alumina (EtOAc as eluent) and concentrated in vacuo. The residue was transferred to a 20 mL two-neck round-bottom flask, and $PdCl_2(PCy_3)_2$ (0.040 mmol, 29.6 mg), $Cs_2CO_3$ (0.4 mmol, 130.4 mg), and DMAc (1.0 mL) were added. The mixture was stirred at 150 °C (oil bath) for 22 hour under $N_2$. The resulting mixture was then quenched with brine and extracted with ethyl acetate (3 x 10 mL). The combined organic layer was dried over sodium sulfate. Concentration in vacuo followed by silica gel column purification with hexane/ethyl acetate (1:1, v/v) gave the desired product **5aa** (75%, 56.3 mg).

## Detailed Optimization Studies

**Table S1.** Optimization Studies for the Synthesis of Starting Benzophosphole **2a** from 1,1-Diphenylethylene **1a** and Phenylphosphinic Acid[a]

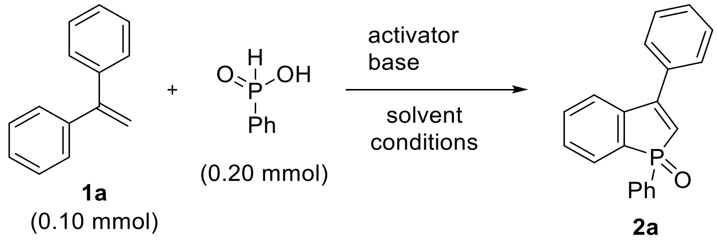


| Entry | Activator (mmol) | Base (mmol) | Solvent | Conditions | Yield of 2a (%)[b] |
|---|---|---|---|---|---|
| 1 | $Tf_2O$ (0.48) | DMAP (0.48) | DCE | 120 °C, 16 h | (69) |
| 2 | $Tf_2O$ (0.48) | DMAP (0.48) | DCE | 90 °C, 16 h | trace |
| 3 | $PhNTf_2$ (0.48) | DMAP (0.48) | DCE | 120 °C, 16 h | n.d. |
| 4 | TfOH (0.96) | none | DCE | 120 °C, 16 h | n.d. |
| 5 | $Tf_2O$ (0.48) | 2,6-lutidine (0.48) | DCE | 120 °C, 16 h | 1 |
| 6 | $Tf_2O$ (0.48) | 4-methylpyridine (0.48) | DCE | 120 °C, 16 h | 76 |
| 7 | $Tf_2O$ (0.48) | pyridine (0.48) | DCE | 120 °C, 16 h | 60 |
| 8 | $Tf_2O$ (0.48) | $Et_3N$ (0.48) | DCE | 120 °C, 16 h | 1 |
| 9 | $Tf_2O$ (0.48) | $K_2CO_3$ (0.48) | DCE | 120 °C, 16 h | 21 |
| 10 | $Tf_2O$ (0.48) | none | DCE | 120 °C, 16 h | n.d. |
| **11** | **$Tf_2O$ (0.48)** | **4-methylpyridine (0.48)** | **touene** | **120 °C, 16 h** | **92(98)** |
| 12 | $Tf_2O$ (0.48) | 4-methylpyridine (0.48) | toluene | 110 °C, 16 h | 81 |
| 13 | $Tf_2O$ (0.48) | 4-methylpyridine (0.48) | $PhCF_3$ | 120 °C, 16 h | 78 |
| 14 | $Tf_2O$ (0.48) | 4-methylpyridine (0.48) | dioxane | 120 °C, 16 h | n.d. |
| 15 | $Tf_2O$ (0.48) | 4-methylpyridine (0.48) | HFIP | 120 °C, 16 h | 1 |

[a]Reaction conditions: **1a** (0.10 mmol), phenylphosphinic acid (0.20 mmol), activator (0.48 mmol), base (0.48 mmol), solvent (1.5 mL), $N_2$. [b]Determined by $^{31}P$ NMR using triethylphosphate as an internal standard. Isolated yield is in parentheses.

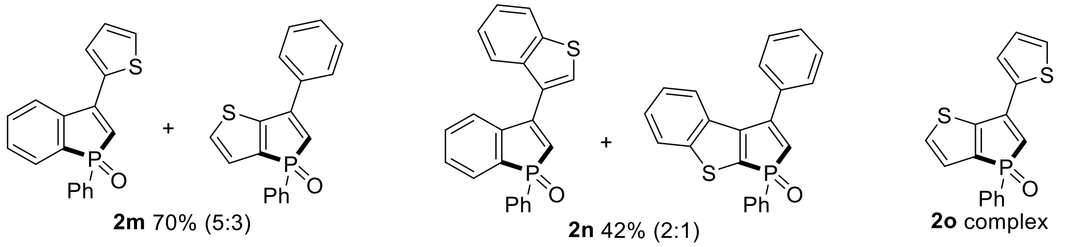


**Scheme S1** Unsuccessful heterocyclic systems in starting material preparation.

**Table S2.** Optimization Studies for Palladium-Catalysed C–H Arylation of Benzophosphole **2a** with 4-Iodotoluene **3a**[a]

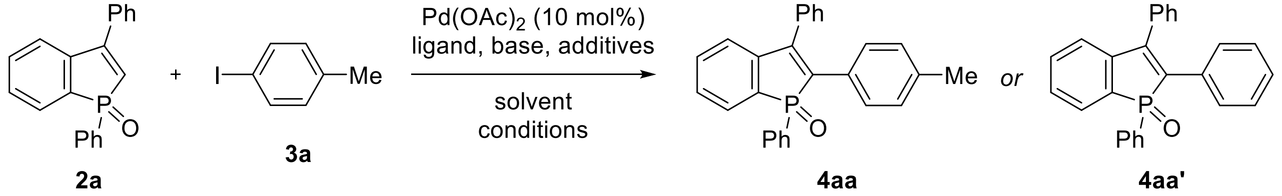


| Entry | Ligand (mol%) | Base (equiv) | Additives (equiv) | Solvent (mL) | Conditions | Yield of 4aa or 4aa' (%)[b] |
|---|---|---|---|---|---|---|
| 1 | | NaO*t*Bu (2.0) | | toluene (1.5) | 90 °C, 16 h | 66 |
| 2 | | NaO*t*Bu (2.0) | | toluene (1.5) | 110 °C, 16 h | 32 |
| 3[c] | | NaO*t*Bu (2.0) | | toluene (1.5) | 90 °C, 22 h | 60(66) |
| 4[c] | | NaO*t*Bu (2.0) | | toluene (1.5) | 90 °C, 11 h | 42 |
| 5[c] | | NaO*t*Bu (2.0) | | toluene (1.5) | 80 °C, 22 h | 55 |
| 6[d] | | NaO*t*Bu (2.0) | | toluene (1.5) | 90 °C, 16 h | n.d. |
| 7 | SPhos (20) | NaO*t*Bu (2.0) | | toluene (1.5) | 90 °C, 16 h | 21 |
| 8 | XPhos (20) | NaO*t*Bu (2.0) | | toluene (1.5) | 90 °C, 16 h | 19 |
| 9 | $PPh_2Cy$ (20) | NaO*t*Bu (2.0) | | toluene (1.5) | 90 °C, 16 h | 56 |
| 10 | $PPh_3$ (20) | NaO*t*Bu (2.0) | | toluene (1.5) | 90 °C, 16 h | 39 |
| 11[e] | | NaO*t*Bu (2.0) | | toluene (1.5) | 90 °C, 16 h | 49 |
| 12[f] | | NaO*t*Bu (2.0) | | toluene (1.5) | 110 °C, 16 h | 0 |
| 13[g] | | NaO*t*Bu (2.0) | | toluene (1.5) | 90 °C, 16 h | trace |
| 14 | | KO*t*Bu (2.0) | | toluene (1.5) | 90 °C, 16 h | 68 |
| 15 | | LiO*t*Bu (2.0) | | toluene (1.5) | 110 °C, 16 h | 21 |
| 16 | | LiO*t*Bu (2.0) | | DMF (1.5) | 90 °C, 18 h | 13 |
| 17 | | NaO*t*Amyl (2.0) | | toluene (1.5) | 90 °C, 16 h | 28 |
| 18 | | $Cs_2CO_3$ (2.0) | | toluene (1.5) | 110 °C, 16 h | 0 |
| 19 | | NaO*t*Bu (1.5) | | toluene (1.5) | 90 °C, 16 h | 39 |
| 20 | | NaO*t*Bu (1.0) | | toluene (1.5) | 110 °C, 16 h | 23 |
| 21 | | NaO*t*Bu (1.5) | DIPEA (1.0) | toluene (1.5) | 90 °C, 16 h | 43 |
| 22 | | NaO*t*Bu (1.5) | DIPEA (1.0) | toluene (1.5) | 100 °C, 11 h | 46 |
| 23 | | NaO*t*Bu (2.0) | | mesitylene (1.5) | 90 °C, 16 h | 44 |
| 24 | | NaO*t*Bu (2.0) | | *o*-xylene (1.5) | 90 °C, 16 h | 61 |
| 25 | | NaO*t*Bu (2.0) | | benzene (1.5) | 90 °C, 16 h | 58 |
| 26[c] | | NaO*t*Bu (2.0) | | $PhCF_3$ (1.5) | 90 °C, 18 h | 61 |
| 27[c] | | NaO*t*Bu (2.0) | | MeCN (1.5) | 90 °C, 16 h | complex |
| 28[c] | | NaO*t*Bu (2.0) | | octane (1.5) | 90 °C, 16 h | 9 |

| | | | | | |
|---|---|---|---|---|---|
| 29 | NaO*t*Bu (2.0) | LiCl (1.0) | toluene (1.5) | 90 °C, 16 h | 67 |
| 30 | NaO*t*Bu (2.0) | TBAI (1.0) | toluene (1.5) | 90 °C, 16 h | 59 |
| 31 | NaO*t*Bu (2.0) | TBAB (1.0) | toluene (1.5) | 90 °C, 16 h | 22 |
| 32 | NaO*t*Bu (2.0) | $Ag_2CO_3$ (0.5) | toluene (1.5) | 90 °C, 12 h | 42 |
| 33 | NaO*t*Bu (1.5) | $Ag_2CO_3$ (0.5) | toluene (1.5) | 90 °C, 18 h | 32 |
| 34 | NaO*t*Bu (2.0) | AgOAc (1.0) | toluene (1.5) | 90 °C, 12 h | 54 |
| 35 | NaO*t*Bu (2.0) | AgTFA (1.0) | toluene (1.5) | 90 °C, 16 h | 46 |
| 36 | NaO*t*Bu (2.0) | $Cu(OAc)_2$ (2.0) | toluene (1.5) | 90 °C, 16 h | 43 |
| 37 | NaO*t*Bu (2.0) | CuOAc (2.0) | toluene (1.5) | 90 °C, 16 h | 51 |
| 38 | NaO*t*Bu (2.0) | | toluene (2.0) | 90 °C, 16 h | 73(76) |
| 39 | NaO*t*Bu (2.0) | | toluene (2.0) | 100 °C, 8 h | 70 |
| 40 | NaO*t*Bu (2.0) | | toluene (3.0) | 90 °C, 16 h | 43 |
| 41 | NaO*t*Bu (2.0) | | toluene (2.0) | μw, 100 °C, 2 h | 71 |
| 42 | NaO*t*Bu (2.0) | | toluene (1.5) | μw, 100 °C, 1 h | 78 |
| 43 | NaO*t*Bu (2.0) | | toluene (1.5) | μw, 90 °C, 2 h | 78 |
| 44 | NaO*t*Bu (2.0) | | toluene (1.5) | μw, 80 °C, 2 h | 35 |
| 45 | NaO*t*Bu (2.0) | | toluene (1.5) | μw, 90 °C, 1 h | 72 |
| 46 | NaO*t*Bu (2.0) | | toluene (1.0) | μw, 90 °C, 1 h | 83 |
| 47 | NaO*t*Bu (2.0) | | toluene (0.5) | μw, 90 °C, 1 h | 76 |
| **48**[h] | **NaO*t*Bu (2.0)** | | **toluene (2.0)** | **μw, 90 °C, 1 h** | **81(79)** |
| 49[e] | NaO*t*Bu (2.0) | | toluene (1.0) | μw, 90 °C, 1 h | 74 |
| 50[i] | NaO*t*Bu (2.0) | | toluene (1.0) | μw, 90 °C, 1 h | n.d. |
| 51[j] | NaO*t*Bu (2.0) | | toluene (1.0) | μw, 90 °C, 1 h | trace |

[a]Reaction conditions: $Pd(OAc)_2$ (0.010 mmol), **2a** (0.10 mmol), **3a** (0.15 mmol), solvent (1.5 mL), $N_2$. [b]Determined by $^1$H NMR using triethylphosphate as an internal standard. Isolated yields are in parentheses. [c]With 0.20 mmol of **2a**. [d]Without $Pd(OAc)_2$. [e]4-Bromotoluene was used instead of **3a**. [f]PhOTf was used instead of **3a**. The targeted product was **4aa'**. [g]$Ph_2IOTf$ was used instead of **3a**. The targeted product was **4aa'**. [h]On a 0.20 mmol scale with $Pd(OAc)_2$ (0.010 mmol, 5 mol %). [i]PhOTs was used instead of **3a**. The targeted product was **4aa'**. [j]4-Chlorotoluene was used instead of **3a**.

**Table S3.** Optimization Studies for Palladium-Catalysed C–H Arylation of Benzophosphole **2a** with 4-Chlorotoluene **3a-Cl**[a]

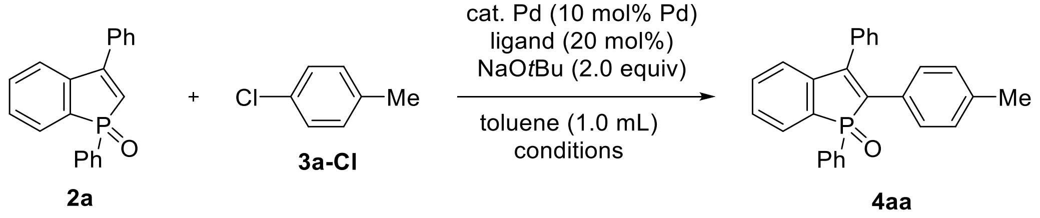


| Entry | Cat. Pd | Ligand (20 mol%) | Conditions | Yield of 4aa (%)[b] |
|---|---|---|---|---|
| 1 | $Pd_2(dba)_3$ | XPhos | μw, 100 °C, 1 h | 15 |
| 2 | $Pd_2(dba)_3$ | XPhos | μw, 140 °C, 1 h | 11 |
| 3 | $Pd_2(dba)_3$ | | μw, 100 °C, 1 h | trace |
| 4[c] | $PdCl_2(Cy_3P)_2$ | | μw, 110 °C, 1 h | 11 |
| **5** | **$Pd_2(dba)_3$** | **XPhos** | **110 °C, 12 h** | **45** |
| 6[d] | $Pd_2(dba)_3$ | XPhos | 90 °C, 20 h | 42 |
| 7 | $Pd_2(dba)_3$ | XPhos | 70 °C, 16 h | 33 |
| 8 | $Pd_2(dba)_3$ | $PCy_3H{\cdot}BF_4$ | 90 °C, 16 h | complex |
| 9 | $Pd_2(dba)_3$ | $PCy_3$ | 100 °C, 16 h | 32 |
| 10 | $Pd(OAc)_2$ | XPhos | 110 °C, 12 h | 6 |
| 11 | $Pd(OAc)_2$ | SPhos | 110 °C, 12 h | complex |
| 12[c] | XPhos Pd G4 | | 110 °C, 6 h | 16 |
| 13[c] | XPhos Pd G4 | | 110 °C, 18 h | 14 |
| 14[c, e] | XPhos Pd G4 | | 110 °C, 10 h | 24 |
| 15[c] | RuPhos Pd G3 | | 110 °C, 10 h | trace |
| 16 | CpPd(π-allyl) | XPhos | 110 °C, 17 h | n.d. |
| 17 | $(cod)Pd(CH_2TMS)_2$ | XPhos | 110 °C, 17 h | n.d. |
| 18 | $Pd_2(dba)_3{\cdot}CHCl_3$ | XPhos | 110 °C, 12 h | n.d. |
| 19[c,d] | $Pd(tBu_3P)_2$ | | 100 °C, 15 h | 51 |
| 20[c,d] | $Pd(Cy_3P)_2$ | | 100 °C, 15 h | 62 |
| 21[c,d] | $Pd(Ph_3P)_4$ | | 100 °C, 15 h | n.d. |
| 22[c,d] | $Pd(Cy_3P)_2$ | | μw, 100 °C, 1 h | 33 |
| **23[c,d]** | **$Pd(Cy_3P)_2$** | | **110 °C, 12 h** | **69(69)** |
| 24[f] | $Pd(Cy_3P)_2$ | | 110 °C, 12 h | 40 |
| 25[g] | $Pd(Cy_3P)_2$ | | 110 °C, 12 h | 11 |

[a]Reaction conditions: Pd catalyst (0.010 mmol, based on Pd), **2a** (0.10 mmol), **3a-Cl** (0.15 mmol), solvent (1.0 mL), $N_2$. [b]Determined by $^1$H NMR using triethylphosphate as an internal standard. Isolated yield is in parentheses. [c]With 5 mol % Pd loading. [d]With 0.30 mmol of **3a-Cl**. [e]Dioxane

(1.0 mL) was used instead of toluene. [f]With 0.30 mmol of 4-iodotoluene. [g]With 0.30 mmol of 4-bromotoluene.

## Hammett Studies

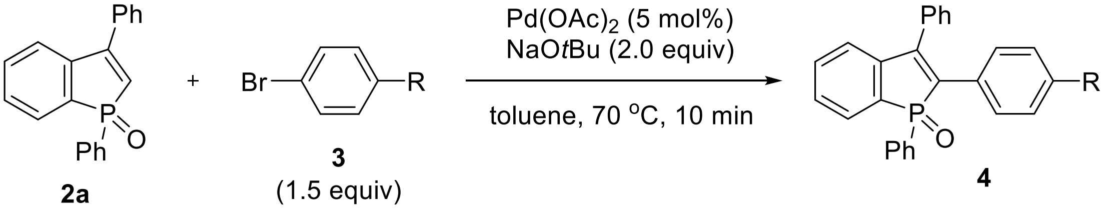


Yields of **4** (NMR)
R = OPh: 6% R = OMe: 9% R = Me: 13% R = H: 15%
R = F: 7% R = Cl: 6% R = $CF_3$: 2%

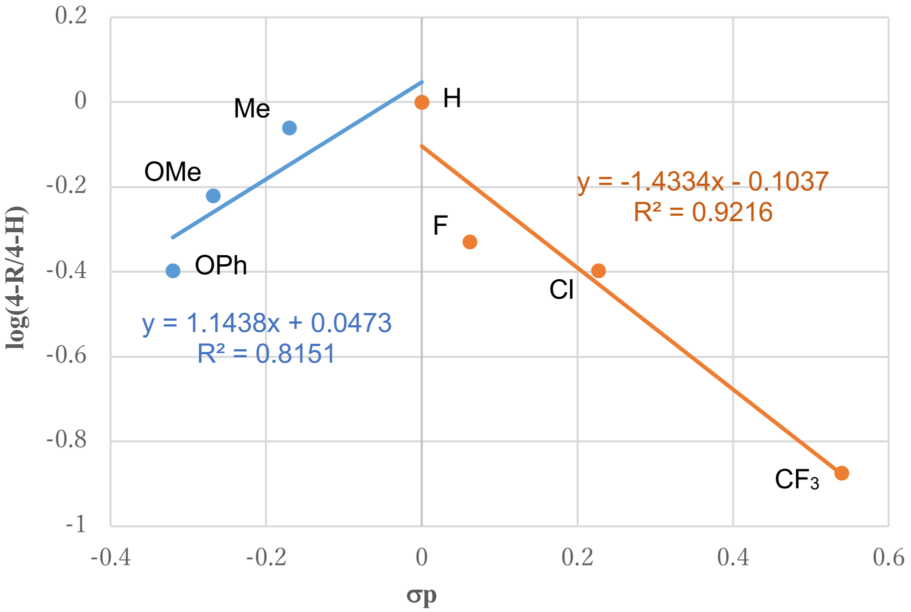


**Figure S1.** Hammett plot with *para*-substituted aryl bromides **3**.

## Cyclic Voltammetry and Differential Pulse Voltammetry

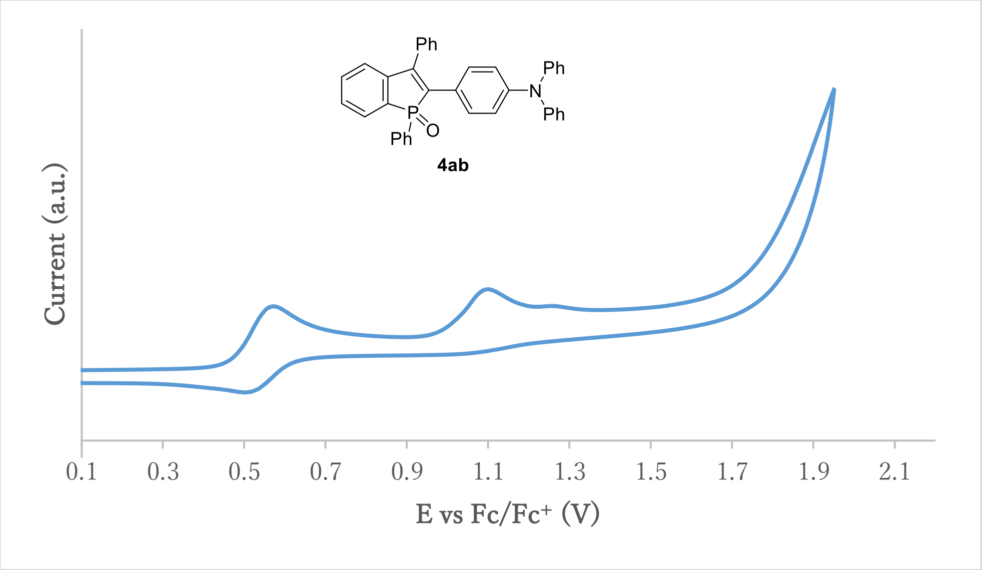


**Figure S2**. Cyclic voltammograms of **4ab** in MeCN containing 0.1 M n-$Bu_4NPF_6$ at a scan rate of 0.05 V/s.

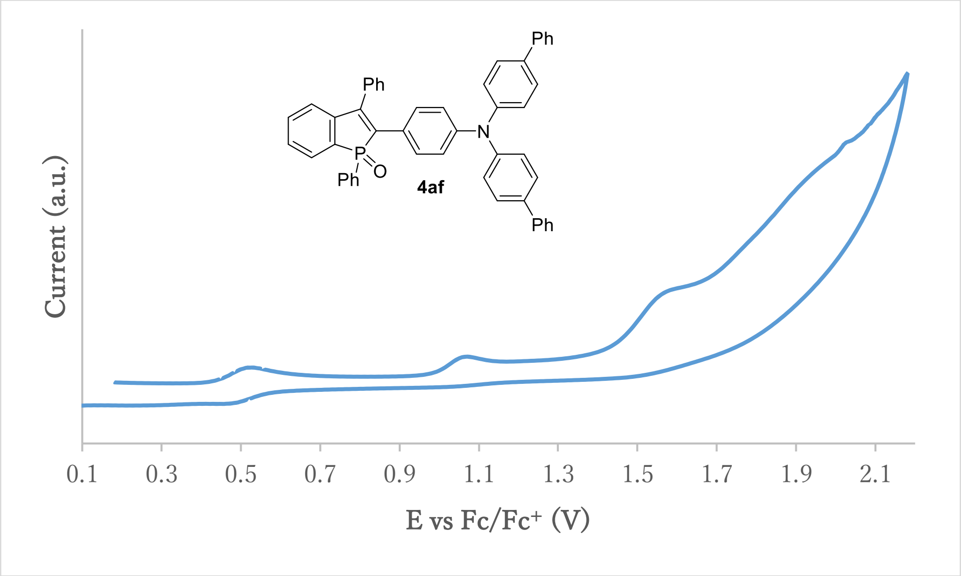


**Figure S3**. Cyclic voltammograms of **4af** in MeCN containing 0.1 M n-$Bu_4NPF_6$ at a scan rate of 0.05 V/s.

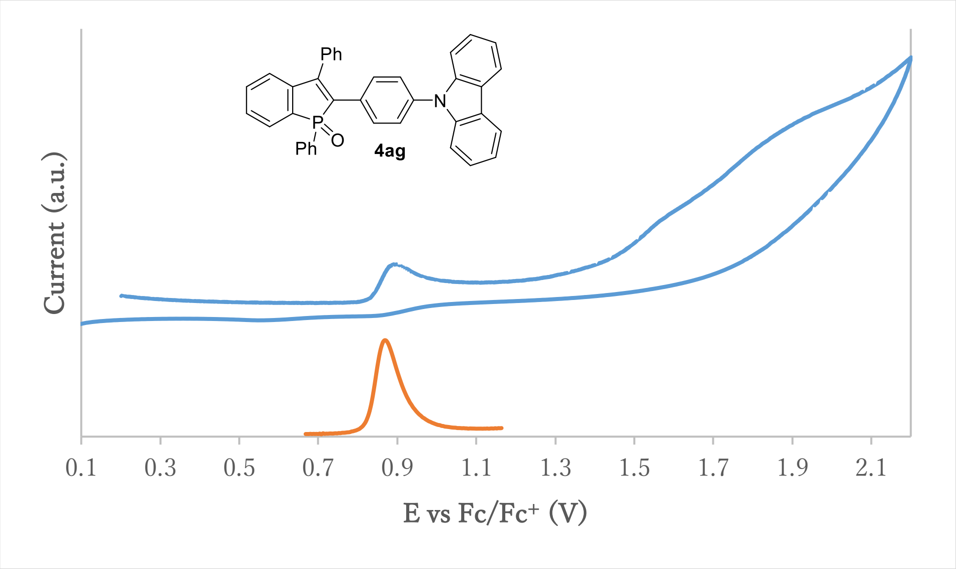


**Figure S4**. Cyclic voltammograms (blue line) and differential pulse voltammograms (orange line) of **4ag** in MeCN containing 0.1 M n-$Bu_4NPF_6$ at a scan rate of 0.1 V/s.

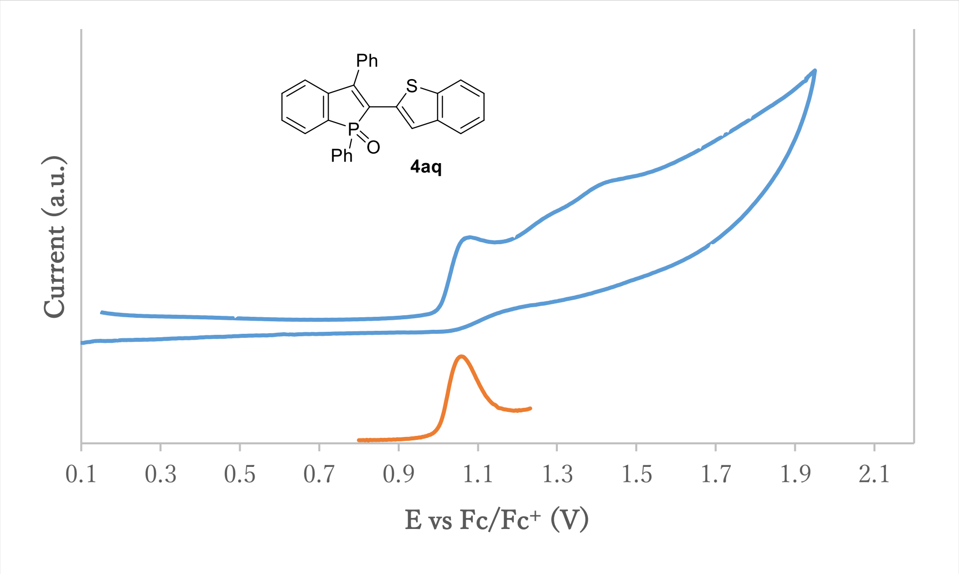


**Figure S5**. Cyclic voltammograms (blue line) and differential pulse voltammograms (orange line) of **4aq** in MeCN containing 0.1 M n-$Bu_4NPF_6$ at a scan rate of 0.05 V/s.

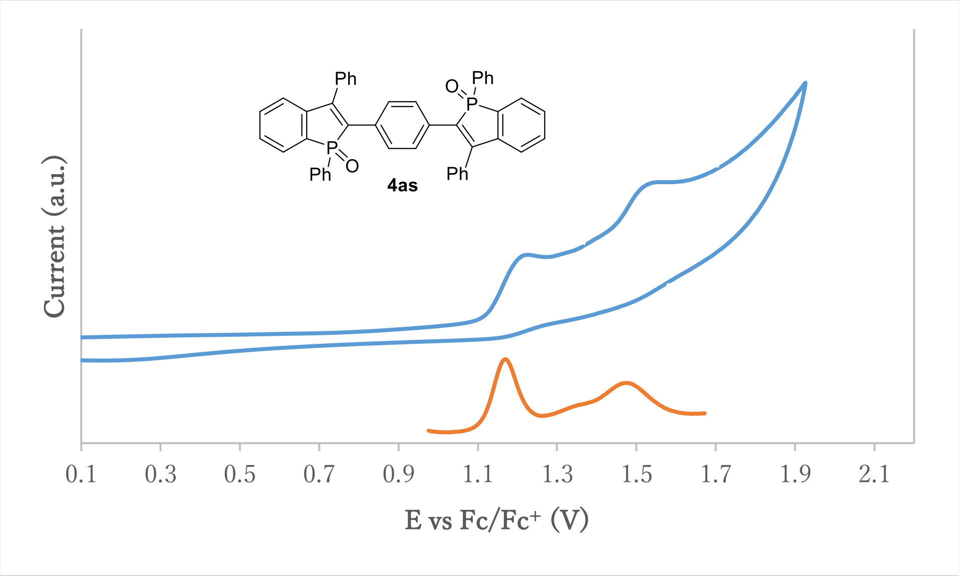


**Figure S6**. Cyclic voltammograms (blue line) and differential pulse voltammograms (orange line) of **4as** in MeCN containing 0.1 M n-$Bu_4NPF_6$ at a scan rate of 0.05 V/s.

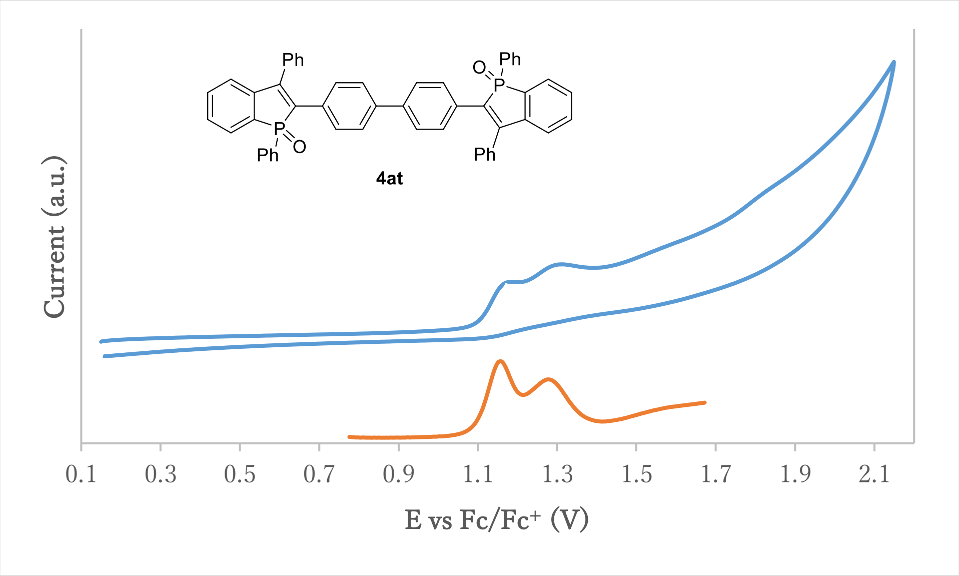


**Figure S7**. Cyclic voltammograms (blue line) and differential pulse voltammograms (orange line) of **4at** in MeCN containing 0.1 M n-$Bu_4NPF_6$ at a scan rate of 0.03 V/s.

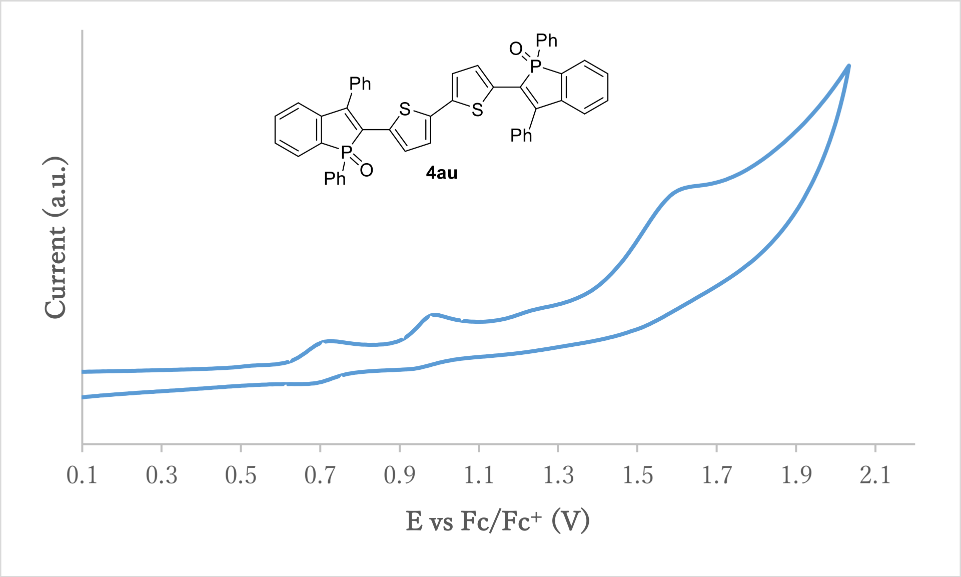


**Figure S8**. Cyclic voltammograms of **4au** in MeCN containing 0.1 M n-$Bu_4NPF_6$ at a scan rate of 0.05 V/s.

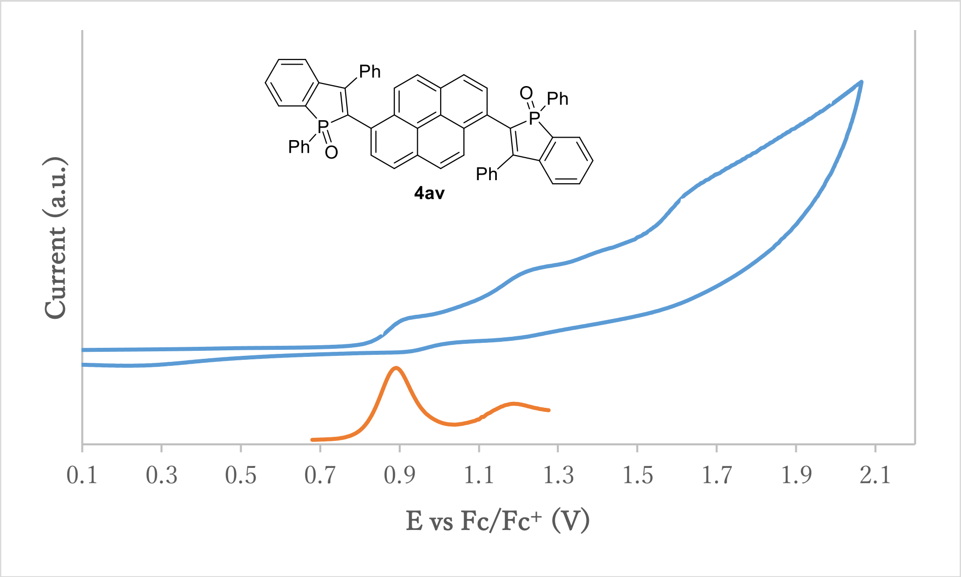


**Figure S9**. Cyclic voltammograms (blue line) and differential pulse voltammograms (orange line) of **4av** in MeCN containing 0.1 M n-$Bu_4NPF_6$ at a scan rate of 0.05 V/s.

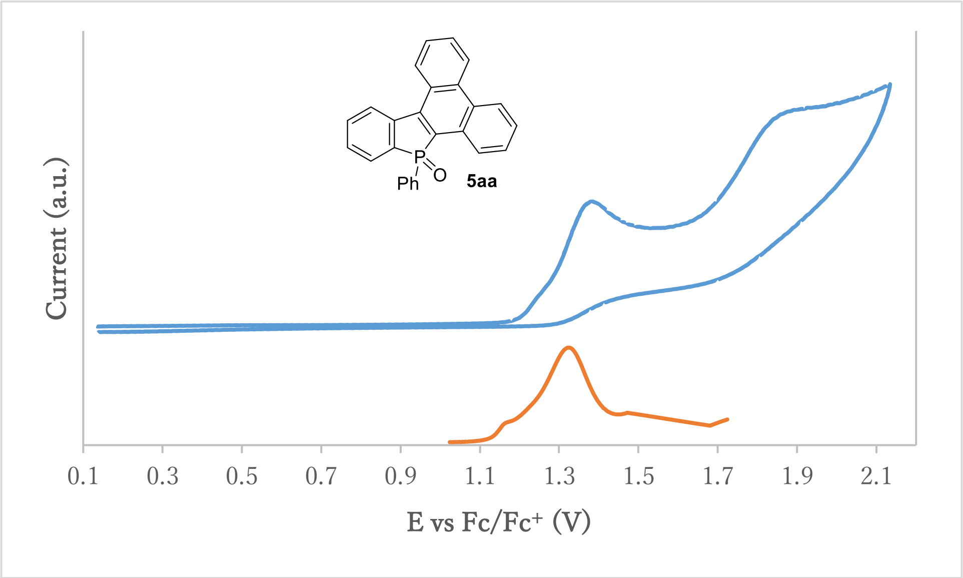


**Figure S10**. Cyclic voltammograms (blue line) and differential pulse voltammograms (orange line) of **5aa** in MeCN containing 0.1 M n-$Bu_4NPF_6$ at a scan rate of 0.05 V/s.

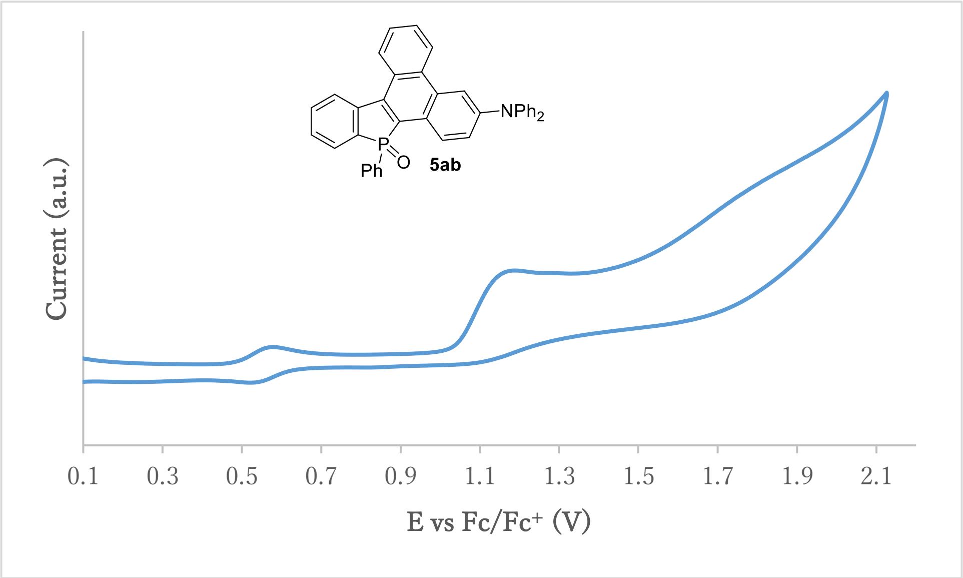


**Figure S11**. Cyclic voltammograms of **5ab** in MeCN containing 0.1 M n-$Bu_4NPF_6$ at a scan rate of 0.03 V/s.

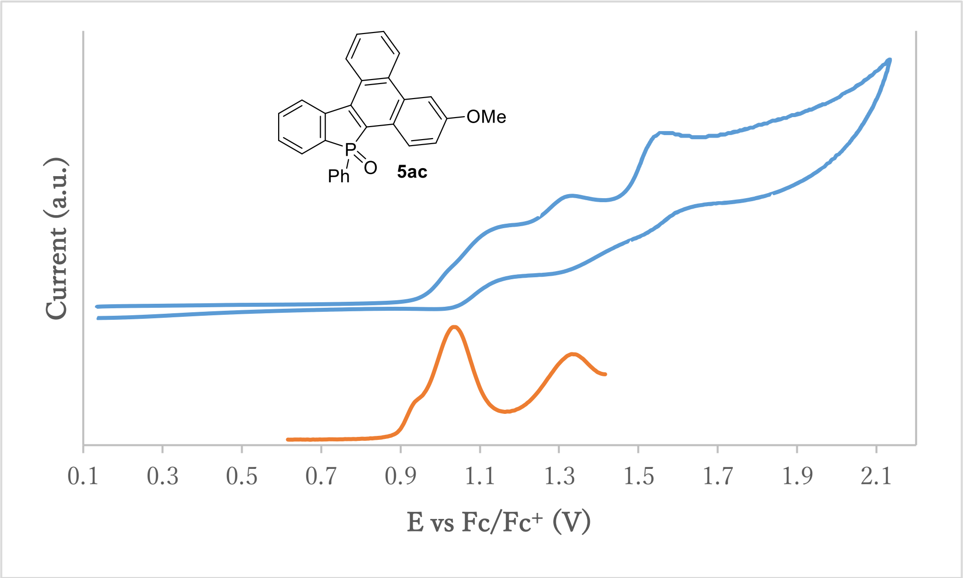


**Figure S12**. Cyclic voltammograms (blue line) and differential pulse voltammograms (orange line) of **5ac** in MeCN containing 0.1 M n-$Bu_4NPF_6$ at a scan rate of 0.03 V/s.

## Absorption and Fluorescence Spectra

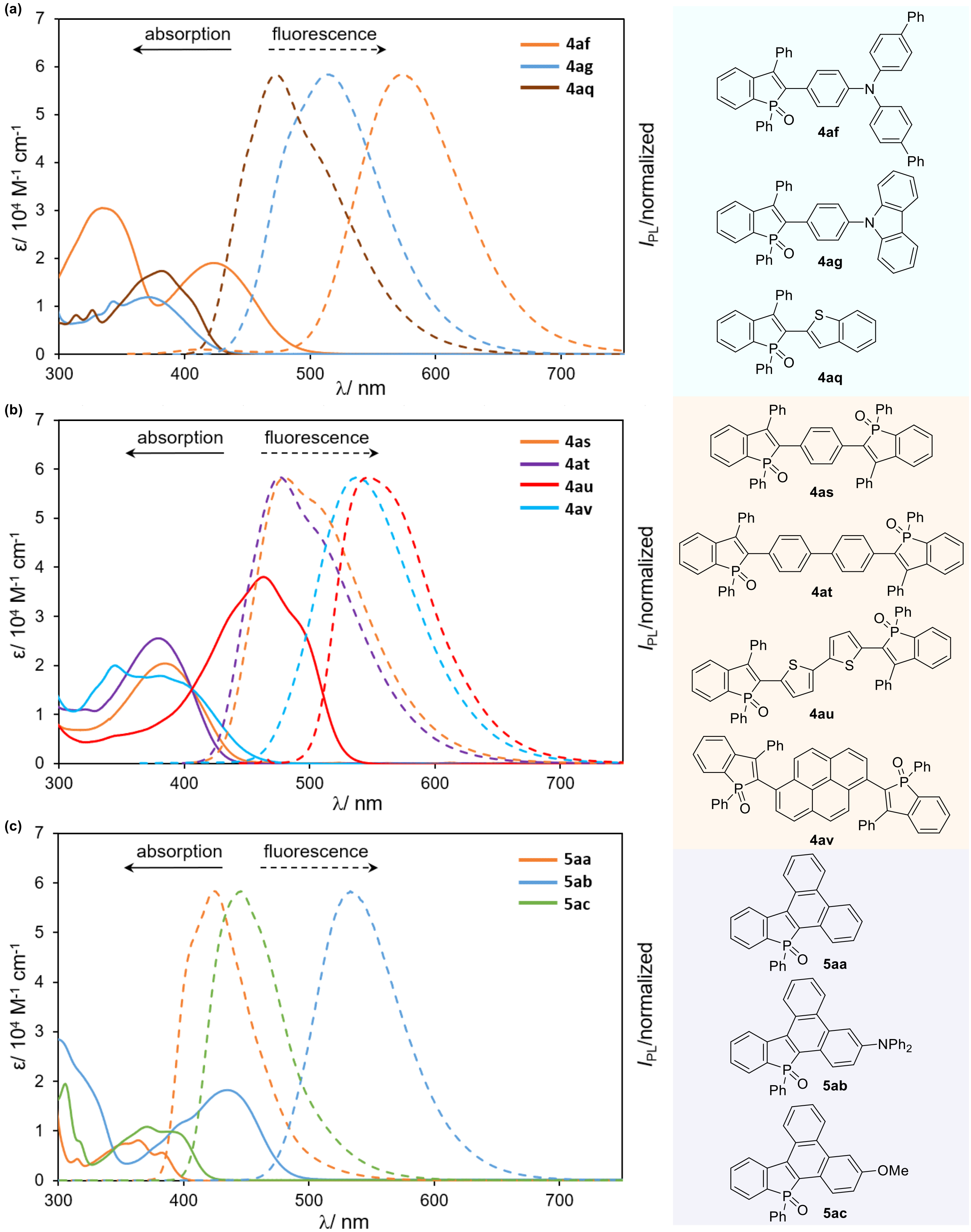


**Figure S13.** UV-vis absorption (solid line) and fluorescence spectra (dashed line; excited at the absorption maxima) of (a) **4af-ag**, and **4aq**, (b) **4as-av**, and **5aa-ac** in $CH_2Cl_2$ ($c = 10^{-5}$ M).

## Theoretical Calculations

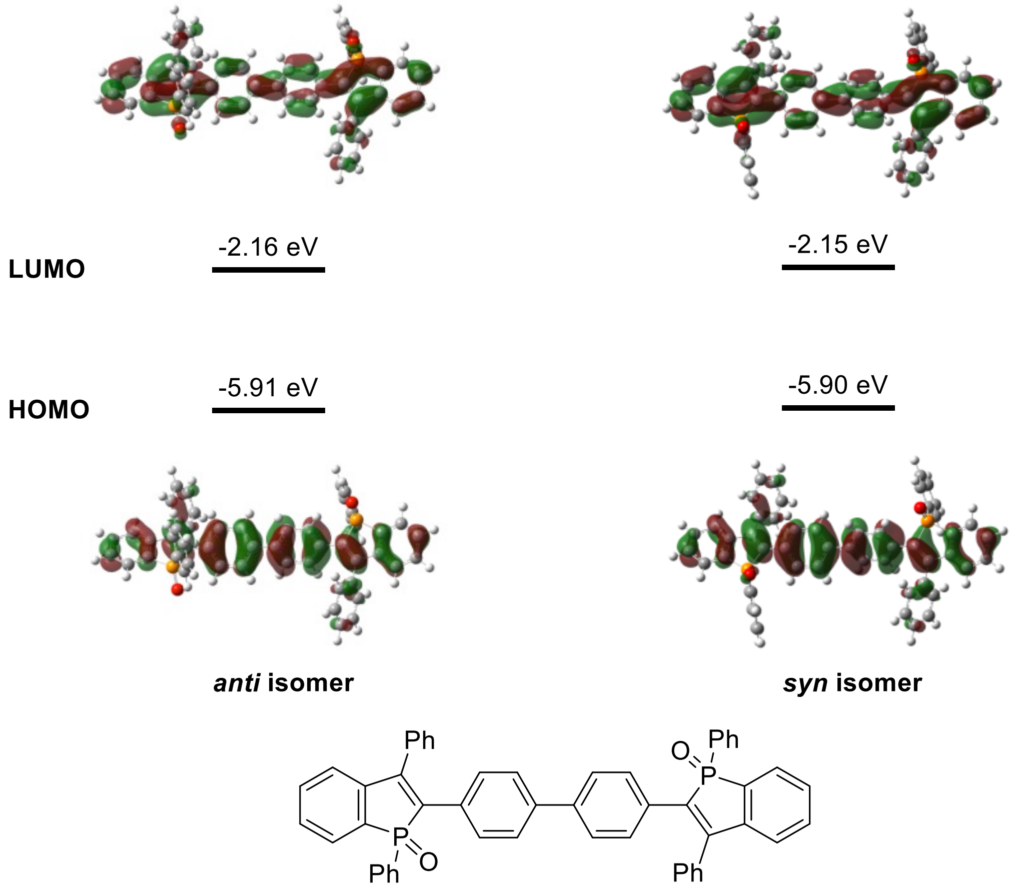


**4at**

**Figure S14.** Kohn-Sham molecular orbitals of **4at** calculated at the PBE0/6-31 +G(d) level of theory, implemented in the Gaussian 16 program.

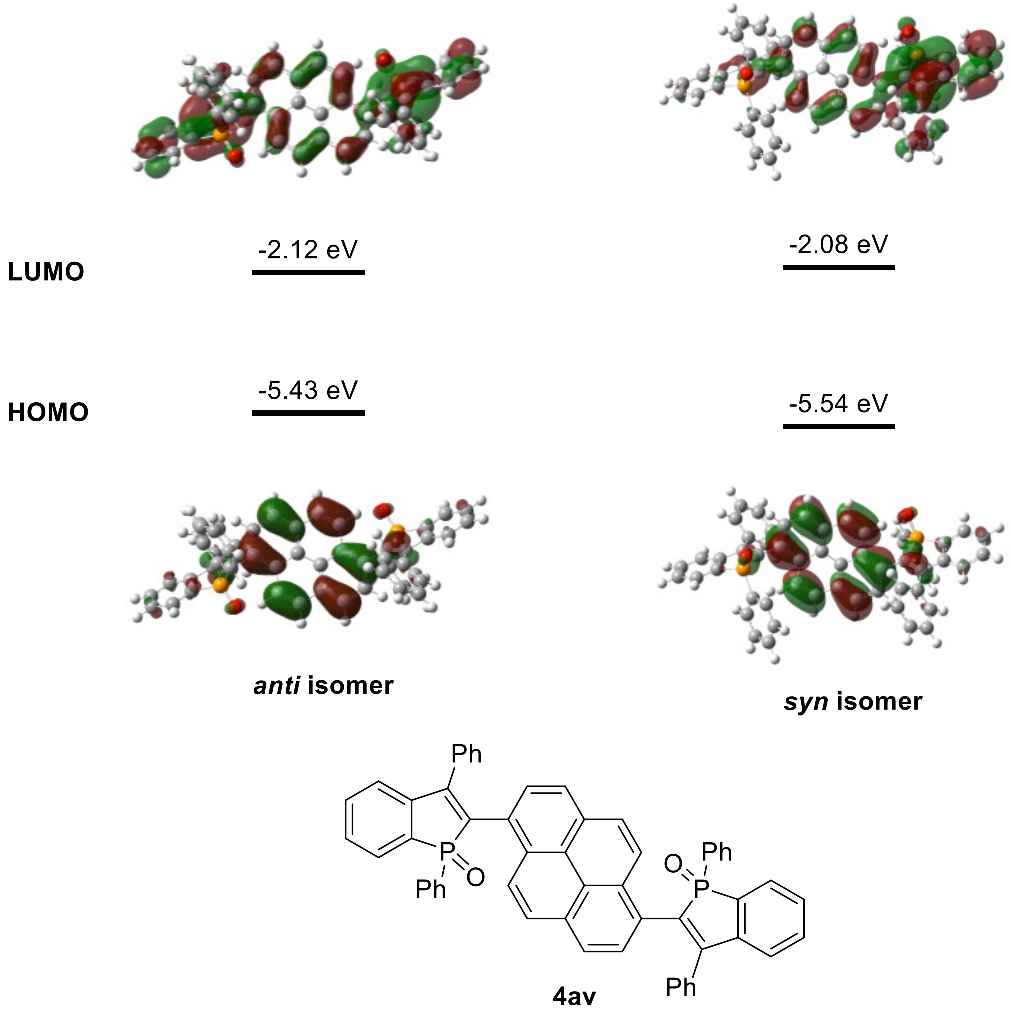


**Figure S15.** Kohn-Sham molecular orbitals of **4av** calculated at the PBE0/6-31 +G(d) level of theory, implemented in the Gaussian 16 program.

## X-Ray Analysis

The single crystals of **2b** suitable for X-ray analysis were grown from $CHCl_3$/hexane. The structure was refined by full-matrix least-squares method using SHELXL-2017/1.

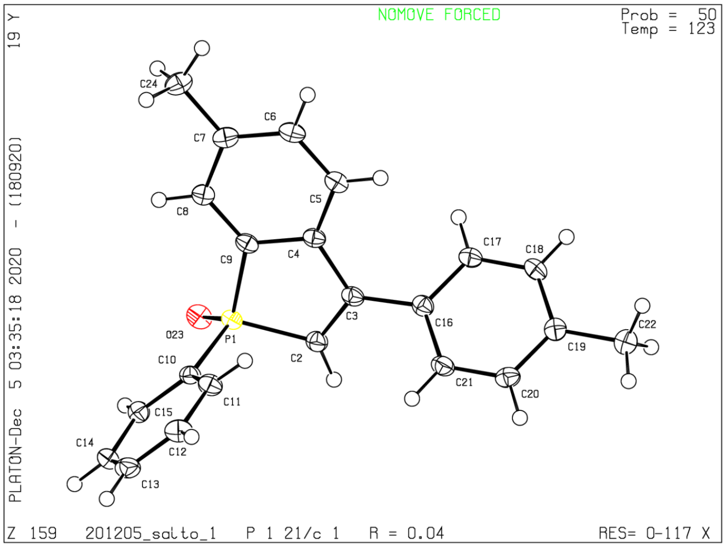


**Figure S16.** ORTEP drawing of **2b** (CCDC 2174057, 50% thermal probability).

**Table S4**. Crystal Data for **2b**

| | |
|---|---|
| Crystal system | monoclinic |
| Space group IT number | 14 |
| Space group name H-M alt | P 1 21/c 1 |
| Space group name Hall | -P 2ybc |
| Cell length a | 12.5743(3) |
| Cell length b | 11.3680(2) |
| Cell length c | 11.7601(2) |
| Cell angle alpha | 90 |
| Cell angle beta | 93.369(2) |
| Cell angle gamma | 90 |
| Cell volume | 1678.14(6) |
| Cell formula units Z | 4 |
| Refine ls R factor all | 0.0435 |
| Refine ls R factor gt | 0.0403 |
| Refine ls wR factor gt | 0.1064 |
| Refine ls wR factor ref | 0.1094 |
| Refine ls goodness of fit ref | 1.064 |

The single crystals of **4av** suitable for X-ray analysis were grown from $CHCl_3$/EtOAc/hexane. The structure was refined by full-matrix least-squares method using SHELXL-2017/1.

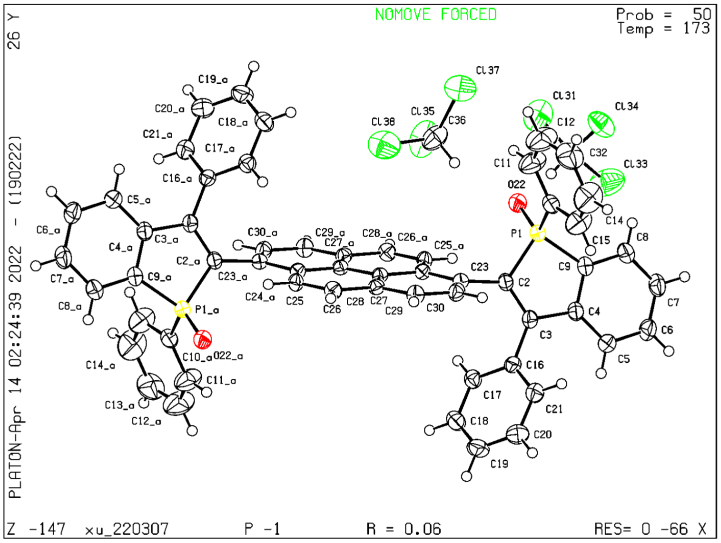


**Figure S17.** ORTEP drawing of **4av** (CCDC 2166424, 50% thermal probability).

**Table S5**. Crystal Data for **4av**

| | |
|---|---|
| Crystal system | triclinic |
| Space group IT number | 2 |
| Space group name H-M alt | P -1 |
| Space group name Hall | -P 1 |
| Cell length a | 10.0941(3) |
| Cell length b | 10.4249(3) |
| Cell length c | 15.3161(3) |
| Cell angle alpha | 73.955(2) |
| Cell angle beta | 87.469(2) |
| Cell angle gamma | 73.633(3) |
| Cell volume | 1485.17(7) |
| Cell formula units Z | 2 |
| Refine ls R factor all | 0.0877 |
| Refine ls R factor gt | 0.0633 |
| Refine ls wR factor gt | 0.1680 |
| Refine ls wR factor ref | 0.1847 |
| Refine ls goodness of fit ref | 1.113 |

**Characterization Data for Substrates and Products**

The characterization data for **2a** is consistent with that reported in our previous work.[S3]

$^{1}H$, $^{13}C\{^{1}H\}$, $^{19}F\{^{1}H\}$, and $^{31}P\{^{1}H\}$ NMR spectra for all compounds are attached in the last part.

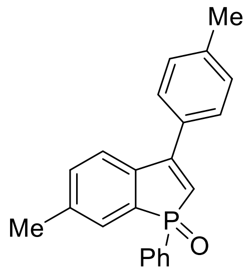

**6-Methyl-1-phenyl-3-(*p*-tolyl)phosphindole 1-oxide (2b)** : Purified by flash chromatography with hexane/ethyl acetate (1/2, v/v): 28 mg (85%, 0.10 mmol scale); pale yellow solid; m.p. 157.6 – 158.7 °C; $^{1}H$ NMR ($CDCl_3$, 400 MHz): $\delta$ 7.82-7.76 (m, 2H), 7.55-7.42 (m, 6H), 7.37 (dd, $J$ = 3.0, 7.8 Hz, 1H), 7.30 (d, $J$ = 7.9 Hz, 2H), 7.27 (d, $J$ = 10.4 Hz, 1H), 6.26 (d, $J$ = 24.2 Hz, 1H), 2.43 (s, 3H), 2.36 (s, 3H); $^{13}C\{^{1}H\}$ NMR ($CDCl_3$, 100 MHz): $\delta$ 158.3 (d, $^{2}J_{CP}$ = 15.4 Hz, 1C), 140.0 (d, $^{3}J_{CP}$ = 10.5 Hz, 1C), 139.8 (1C), 139.7 (d, $^{2}J_{CP}$ = 27.0 Hz, 1C), 134.4 (d, $^{1}J_{CP}$ = 104.1 Hz, 1C), 133.0 (1C), 132.5 (d, $^{3}J_{CP}$ = 16.4 Hz, 1C), 132.3 (d, $^{4}J_{CP}$ = 2.8 Hz, 1C), 131.1 (d, $^{2}J_{CP}$ = 10.8 Hz, 2C), 130.2 (d, $^{2}J_{CP}$ = 9.8 Hz, 1C), 130.1 (d, $^{1}J_{CP}$ = 101.4 Hz, 1C), 129.6 (2C), 128.9 (d, $^{3}J_{CP}$ = 12.4 Hz, 2C), 127.9 (2C), 124.0 (d, $^{3}J_{CP}$ = 11.9 Hz, 1C), 121.3 (d, $^{1}J_{CP}$ = 99.9 Hz, 1C), 21.6 (1C), 21.4 (1C); $^{31}P\{^{1}H\}$ NMR ($CDCl_3$, 162 MHz): $\delta$ 36.92; HRMS (APCI) m/z $(M+H)^+$ calcd for $C_{22}H_{20}OP$: 331.1246, found: 331.1224.

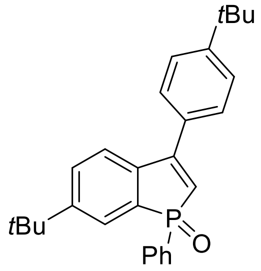

**6-(*tert*-Butyl)-3-(4-(*tert*-butyl)phenyl)-1-phenylphosphindole 1-oxide (2c)** : Purified by flash chromatography with hexane/ethyl acetate (1/2, v/v): 29 mg (70%, 0.10 mmol scale); pale yellow solid; m.p. 180.4 – 181.6 °C; $^{1}H$ NMR ($CDCl_3$, 400 MHz): $\delta$ 7.82-7.72 (m, 3H), 7.52-7.49 (m, 6H), 7.47-7.42 (m, 3H), 6.29 (d, $J$ = 24.2 Hz, 1H), 1.38 (s, 9H), 1.30 (s, 9H); $^{13}C\{^{1}H\}$ NMR ($CDCl_3$, 100 MHz); $\delta$ 157.9 (d, $^{2}J_{CP}$ = 15.3 Hz, 1C), 153.3 (d, $^{3}J_{CP}$ = 9.7 Hz, 1C), 153.0 (1C), 139.7 (d, $^{2}J_{CP}$ = 27.6 Hz, 1C), 134.1 (d, $^{1}J_{CP}$ = 104.3 Hz, 1C), 132.4 (d, $^{3}J_{CP}$ = 16.1 Hz, 1C), 132.2 (d, $^{4}J_{CP}$ = 2.6 Hz, 1C), 131.2 (d, $^{2}J_{CP}$ = 10.8 Hz, 2C), 130.2 (d, $^{1}J_{CP}$ = 101.9 Hz, 1C), 129.5 (1C), 128.9 (d, $^{3}J_{CP}$ = 12.3 Hz, 2C), 127.8 (2C), 126.6 (d, $^{2}J_{CP}$ = 10.1 Hz, 1C), 125.8 (2C), 124.0 (d, $^{3}J_{CP}$ = 11.9 Hz, 1C), 121.9 (d, $^{1}J_{CP}$ = 100.7 Hz, 1C), 35.2 (1C), 35.0 (1C), 31.4-31.3 (overlapped, 6C); $^{31}P\{^{1}H\}$ NMR ($CDCl_3$, 162 MHz): $\delta$ 37.31; HRMS (APCI) m/z $(M+H)^+$ calcd for $C_{28}H_{32}OP$: 415.2185, found: 415.2205.

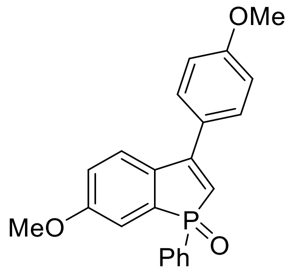

**6-Methoxy-3-(4-methoxyphenyl)-1-phenylphosphindole 1-oxide (2d)** : Purified by flash chromatography with hexane/ethyl acetate (1/2, v/v): 14 mg (40%, 0.10 mmol scale); pale yellow solid; m.p. 61.6 – 62.7 °C; $^{1}$H NMR ($CDCl_3$, 400 MHz): $\delta$ 7.81-7.76 (m, 2H), 7.54-7.51 (m, 3H), 7.47-7.41 (m, 3H), 7.22 (dd, $J$ = 2.5, 11.0 Hz, 1H), 7.02-7.00 (m, 2H), 6.95 (dd, $J$ = 2.5, 8.5 Hz, 1H), 6.16 (d, $J$ = 24.5 Hz, 1H), 3.88 (s, 3H), 3.81 (s, 3H); $^{13}$C{$^{1}$H} NMR ($CDCl_3$, 100 MHz): $\delta$ 161.1 (d, $^{3}J_{CP}$ = 13.6 Hz, 1C), 160.8 (1C), 158.0 (d, $^{2}J_{CP}$ = 15.2 Hz, 1C), 136.5 (d, $^{1}J_{CP}$ = 103.0 Hz, 1C), 134.7 (d, $^{2}J_{CP}$ = 26.9 Hz, 1C), 132.3 (d, $^{4}J_{CP}$ = 2.8 Hz, 1C), 131.1 (d, $^{2}J_{CP}$ = 10.8 Hz, 2C), 130.1 (d, $^{1}J_{CP}$ = 102.2 Hz, 1C), 129.4 (2C), 129.0 (d, $^{3}J_{CP}$ = 12.4 Hz, 2C), 127.8 (d, $^{3}J_{CP}$ = 17.0 Hz, 1C), 125.4 (d, $^{3}J_{CP}$ = 13.2 Hz, 1C), 119.2 (d, $^{1}J_{CP}$ = 101.6 Hz, 1C), 117.4 (d, $^{4}J_{CP}$ = 1.6 Hz, 1C), 115.2 (d, $^{2}J_{CP}$ = 10.9 Hz, 1C), 114.3 (2C), 55.8 (1C), 55.6 (1C); $^{31}$P{$^{1}$H} NMR ($CDCl_3$, 162 MHz): $\delta$ 36.59; HRMS (APCI) m/z $(M+H)^+$ calcd for $C_{22}H_{20}O_3P$: 363.1145, found:363.1115.

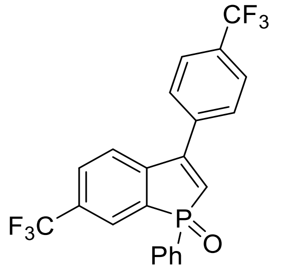

**1-Phenyl-6-(trifluoromethyl)-3-(4-(trifluoromethyl)phenyl)phosphindole 1-oxide (2e):** colorless solid (0.1 mmol scale, 17.9 mg, 41% yield; 1.0 mmol scale, 113.9 mg, 26% yield); m.p. 142.8-143.7 °C (from hexane); TLC $R_f$ 0.25 (hexane/EtOAc, 1:3). $^{1}$H NMR ($CDCl_3$, 400 MHz) δ 7.95 (dd, $J$ = 9.5, 0.5 Hz, 1H), 7.84-7.78 (m, 5H), 7.69 (d, $J$ = 8.0 Hz, 2H), 7.64-7.59 (m, 1H), 7.56-7.49 (m, 3H), 6.61 (d, $J$ = 23.5 Hz, 1H). $^{13}$C{$^{1}$H} NMR ($CDCl_3$, 100 MHz) δ 155.5 (d, $^{2}J_{CP}$ = 14.3 Hz, 1C), 142.1, 144.6 (d, $^{2}J_{CP}$ = 27.1 Hz, 1C), 137.8 (d, $^{3}J_{CP}$ = 16.0 Hz, 1C), 135.6, 134.5, 133.0 (d, $^{4}J_{CP}$ = 2.8 Hz, 1C), 132.5, 132.4, 132.2, 132.1, 131.8, 131.7, 131.5, 131.4, 130.9 (d, $^{2}J_{CP}$ = 11.0 Hz, 2C), 130.19, 130.18, 130.16, 129.2 (d, $^{3}J_{CP}$ = 12.6 Hz, 2C), 128.2, 128.1, 127.8, 127.5, 127.1, 126.5, 126.34, 126.31, 126.27, 126.23, 126.19, 126.17, 126.13, 126.10, 126.0, 125.1, 124.8, 124.79, 123.9 (d, $^{3}J_{CP}$ = 11.0 Hz, 1C), 122.3, 122.09, 122.07, 119.7, 119.4 (All observed signals are just shown because of complexity associated with C–F and C–P couplings.); $^{19}$F{$^{1}$H} NMR ($CDCl_3$, 376 MHz) δ -62.7, -62.8. $^{31}$P{$^{1}$H}

NMR ($CDCl_3$, 162 MHz) δ 35.8; HRMS (APCI) m/z ($[M+H]^+$) Calcd for $C_{22}H_{14}F_6OP$: 439.0681, Found: 439.0667.

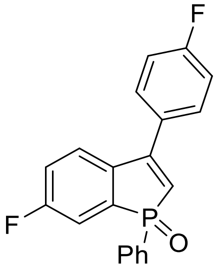

**6-Fluoro-3-(4-fluorophenyl)-1-phenylphosphindole 1-oxide (2f)** : Purified by flash chromatography with hexane/ethyl acetate (1/2, v/v): 26 mg (78%, 0.10 mmol scale); pale yellow solid; m.p. 51.6 – 53.0 °C; $^1$H NMR ($CDCl_3$, 400 MHz): $\delta$ 7.80-7.74 (m, 2H), 7.59-7.50 (m, 3H), 7.49-7.44 (m, 2H), 7.42-7.35 (m, 2H), 7.23-7.14 (m, 3H), 6.33 (d, $J$ = 24.1 Hz, 1H); $^{13}$C{$^1$H} NMR ($CDCl_3$, 100 MHz): $\delta$ 163.9 (dd, $^3J_{CP}/^1J_{CF}$ = 15.2, 252.3 Hz, 1C), 163.7 (d, $^1J_{CF}$ = 248.7 Hz, 1C), 156.6 (d, $^2J_{CP}$ = 14.5 Hz, 1C), 137.8 (dd, $^4J_{CF}/^2J_{CP}$ = 3.0, 26.4 Hz, 1C), 137.1 (dd, $^3J_{CF}/^1J_{CP}$ = 7.0, 103.1 Hz, 1C), 132.8 (d, $^4J_{CP}$ = 2.8 Hz, 1C), 131.0 (d, $^2J_{CP}$ = 10.9 Hz, 2C), 130.9 (dd, $^4J_{CF}/^3J_{CP}$ = 3.5, 16.8 Hz, 1C), 129.8 (d, $^3J_{CF}$ = 8.6 Hz, 2C), 129.2 (d, $^3J_{CP}$ = 12.5 Hz, 2C), 128.9 (d, $^1J_{CP}$ = 103.6 Hz, 1C), 125.6 (dd, $^3J_{CF}/^3J_{CP}$ = 7.9, 13.1 Hz, 1C), 122.8 (dd, $^5J_{CF}/^1J_{CP}$ = 3.7, 100.0 Hz, 1C), 119.2 (dd, $^4J_{CP}/^2J_{CF}$ = 1.2, 22.5 Hz, 1C), 117.3 (dd, $^2J_{CP}/^2J_{CF}$ = 10.7, 23.7 Hz, 1C), 116.2 (d, $^2J_{CF}$ = 21.7 Hz, 2C); $^{19}$F{$^1$H} NMR (376 MHz, $CDCl_3$) δ -110.3 (d, $J$ = 5.4 Hz), -110.5; $^{31}$P{$^1$H} NMR ($CDCl_3$, 162 MHz): $\delta$ 35.44 (d, $J$ = 4.6 Hz); HRMS (APCI) m/z $(M+H)^+$ calcd for $C_{20}H_{14}F_2OP$: 339.0745, found: 339.0763.

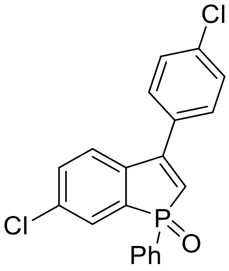

**6-Chloro-3-(4-chlorophenyl)-1-phenylphosphindole 1-oxide (2g)** : Purified by flash chromatography with hexane/ethyl acetate (1/2, v/v): 21 mg (55%, 0.10 mmol scale); pale yellow solid; m.p. 75.6 – 76.7 °C; $^1$H NMR ($CDCl_3$, 400 MHz): $\delta$ 7.79-7.74 (m, 2H), 7.63 (dd, $J$ = 1.7, 9.9 Hz, 1H), 7.60-7.55 (m, 1H), 7.50-7.45 (m, 7H), 7.34 (dd, $J$ = 3.2, 8.2 Hz, 1H), 6.37 (d, $J$ = 23.9 Hz, 1H); $^{13}$C{$^1$H} NMR ($CDCl_3$, 100 MHz): $\delta$ 156.4 (d, $^2J_{CP}$ = 14.6 Hz, 1C), 140.1 (d, $^2J_{CP}$ = 26.7 Hz, 1C), 136.5 (d, $^3J_{CP}$ = 13.9 Hz, 1C), 136.3 (d, $^1J_{CP}$ = 102.3 Hz, 1C), 136.1 (1C), 133.1 (d, $^3J_{CP}$ = 16.5 Hz, 1C), 132.9 (d, $^4J_{CP}$ = 2.8 Hz, 1C), 132.7 (d, $^4J_{CP}$ = 1.4 Hz, 1C), 131.0 (d, $^2J_{CP}$ = 10.8 Hz, 2C), 129.8 (d, $^2J_{CP}$ = 10.5 Hz, 1C), 129.4 (2C), 129.23 (d, $^3J_{CP}$ = 12.5 Hz, 2C), 129.23 (2C), 128.6 (d, $^1J_{CP}$ = 103.9 Hz, 1C), 125.0 (d, $^3J_{CP}$ = 12.2 Hz, 1C), 123.6 (d, $^1J_{CP}$ = 98.9 Hz, 1C); $^{31}$P{$^1$H} NMR ($CDCl_3$,

162 MHz): $\delta$ 35.84; HRMS (APCI) m/z $(M+H)^+$ calcd for $C_{20}H_{14}Cl_2OP$: 371.0154, found: 371.0142.

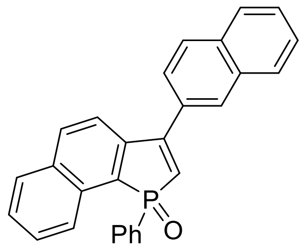

**3-(Naphthalen-2-yl)-1-phenylbenzo[*g*]phosphindole 1-oxide (2h)** : Purified by flash chromatography with hexane/ethyl acetate (1/2, v/v): 25 mg (62%, 0.10 mmol scale); pale yellow solid; m.p. 125.7 – 126.7 °C; $^1$H NMR ($CDCl_3$, 400 MHz): $\delta$ 8.11-8.09 (m, 2H), 8.05-7.99 (dd, $J$ = 8.5, 14.2 Hz, 2H), 7.95-7.85 (m, 5H), 7.69 (dd, $J$ = 2.0, 8.5 Hz, 2H), 7.60-7.57 (m, 2H), 7.53-7.43 (m, 5H), 6.55 (d, $J$ = 25.3 Hz, 1H); $^{13}$C{$^1$H} NMR ($CDCl_3$, 100 MHz): $\delta$ 157.8 (d, $^2J_{CP}$ = 16.6 Hz, 1C), 141.6 (d, $^2J_{CP}$ = 26.1 Hz, 1C), 133.9 (d, $^3J_{CP}$ = 8.6 Hz, 1C), 133.8 (1C), 133.7 (1C), 133.3 (1C), 132.7 (d, $^3J_{CP}$ = 16.7 Hz, 1C), 132.4 (d, $^4J_{CP}$ = 2.8 Hz, 1C), 132.2 (d, $^2J_{CP}$ = 9.2 Hz, 1C), 131.1 (d, $^2J_{CP}$ = 10.9 Hz, 2C), 129.77 (d, $^1J_{CP}$ = 103.2 Hz, 1C), 129.76 (d, $^1J_{CP}$ = 101.0 Hz, 1C), 129.2 (d, $^3J_{CP}$ = 12.4 Hz, 2C), 128.84 (d, $^4J_{CP}$ = 2.0 Hz, 1C), 128.79 (1C), 128.6 (1C), 128.5 (1C), 128.0 (1C), 127.5 (1C), 127.2 (2C), 127.0 (1C), 126.0 (d, $^3J_{CP}$ = 5.0 Hz, 1C), 125.6 (1C), 124.0 (d, $^1J_{CP}$ = 99.1 Hz, 1C), 121.5 (d, $^3J_{CP}$ = 12.8 Hz, 1C); $^{31}$P{$^1$H} NMR ($CDCl_3$, 162 MHz): $\delta$ 38.09; HRMS (APCI) m/z $(M+H)^+$ calcd for $C_{28}H_{20}OP$: 403.1246, found: 403.1250.

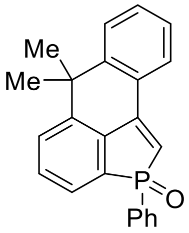

**6,6-Dimethyl-2-phenyl-6*H*-naphtho[1,2,3-*cd*]phosphindole 2-oxide (2i):** pale yellow solid (0.1 mmol scale, 23.9 mg, 70% yield; 3.04 mmol scale, 488.8 mg, 47% yield); m.p. 194.8-195.8 °C (from hexane); TLC $R_f$ 0.20 (hexane/EtOAc, 1:3). $^1$H NMR ($CDCl_3$, 400 MHz) δ 7.91 (dd, $J$ = 7.9, 1.3 Hz, 1H), 7.80-7.75 (m, 2H), 7.69-7.66 (m, 2H), 7.59-7.34 (m, 7H), 6.60 (d, $J$ = 23.2 Hz, 1H), 1.75 (s, 3H), 1.70 (s, 3H). $^{13}$C{$^1$H} NMR ($CDCl_3$, 100 MHz) δ 148.2 (d, $^2J_{CP}$ = 17.0 Hz, 1C), 146.3 (1C), 141.7 (d, $^3J_{CP}$ = 11.3 Hz, 1C), 135.6 (d, $^2J_{CP}$ = 29.5 Hz, 1C), 132.4 (d, $^1J_{CP}$ = 105.8 Hz, 1C), 132.1 (d, $^4J_{CP}$ = 2.5 Hz, 1C), 131.4 (1C), 131.0 (d, $^2J_{CP}$ = 10.8 Hz, 2C), 130.6 (d, $^1J_{CP}$ = 102.9 Hz, 1C), 130.4 (1C), 130.3 (d, $^4J_{CP}$ = 1.9 Hz, 1C), 128.7 (d, $^3J_{CP}$ = 12.2 Hz, 1C), 127.8 (1C), 127.1 (d, $^2J_{CP}$ = 9.6 Hz, 1C), 126.8 (1C), 126.6 (d, $^1J_{CP}$ = 96.8 Hz, 1C), 126.0 (1C), 112.0 (d, $^3J_{CP}$ = 9.7 Hz, 1C), 110.9 (d, $^3J_{CP}$ = 9.1 Hz, 1C), 38.6 (d, $^5J_{CP}$ = 1.0 Hz, 1C), 33.3 (d, $^4J_{CP}$ = 3.4 Hz, 1C), 32.5 (d, $^5J_{CP}$ = 1.9 Hz, 1C). $^{31}$P{$^1$H} NMR ($CDCl_3$, 162 MHz) δ 40.6; HRMS (APCI) m/z ($[M+H]^+$) Calcd for $C_{23}H_{20}OP$:

343.1247, Found: 343.1234.

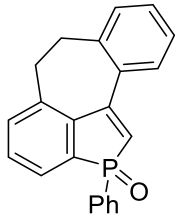


**6-Phenyl-10,11-dihydrobenzo[6,7]cyclohepta[1,2,3-*cd*]phosphindole 6-oxide (2j):** pale yellow solid (6.0 mmol scale, 905.6 mg, 46% yield); m.p. 157.8-158.8 °C (from hexane); TLC $R_f$ 0.20 (hexane/EtOAc, 1:3). $^1$H NMR ($CDCl_3$, 400 MHz) δ 7.78 (dd, $J$ = 12.6, 7.4 Hz, 2H), 7.68 (bs, 1H), 7.54-7.50 (m, 2H), 7.43 (td, $J$ = 7.7, 2.4 Hz, 2H), 7.37 (t, $J$ = 7.4 Hz, 1H), 7.30-7.23 (m, 4H), 6.57 (d, $J$ = 21.6 Hz, 1H), 3.12 (bs, 4H). $^{13}$C{$^1$H} NMR ($CDCl_3$, 100 MHz) δ 158.4 (d, $^2J_{CP}$ = 11.8 Hz, 1C), 142.1 (1C), 140.5 (d, $^3J_{CP}$ = 10.6 Hz, 1C), 139.2 (d, $^2J_{CP}$ = 26.9 Hz, 1C), 135.0 (d, $^4J_{CP}$ = 1.8 Hz, 1C), 134.9 (d, $^3J_{CP}$ = 16.1 Hz, 1C), 132.1 (d, $^4J_{CP}$ = 2.7 Hz, 1C), 131.1 (d, $^2J_{CP}$ = 10.7 Hz, 2C), 130.6 (1C), 129.9 (1C), 129.8 (d, $^1J_{CP}$ = 104.1 Hz, 1C), 129.4 (d, $^1J_{CP}$ = 90.0 Hz, 1C), 129.1 (d, $^3J_{CP}$ = 11.5 Hz, 1C), 129.0 (1C), 128.8 (d, $^3J_{CP}$ = 12.5 Hz, 2C), 127.2 (d, $^2J_{CP}$ = 9.8 Hz, 1C), 126.9 (1C), 122.9 (d, $^1J_{CP}$ = 98.2 Hz, 1C), 35.4 (1C), 34.9 (d, $^4J_{CP}$ = 2.1 Hz, 1C). $^{31}$P{$^1$H} NMR ($CDCl_3$, 162 MHz) δ 34.07; HRMS (APCI) m/z ($[M+H]^+$) Calcd for $C_{22}H_{18}OP$: 329.1090, Found: 329.1073.

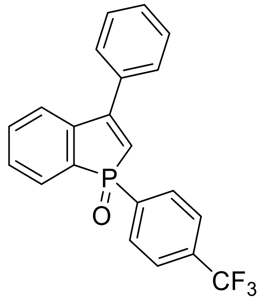


**3-Phenyl-1-(4-(trifluoromethyl)phenyl)phosphindole 1-oxide (2k)** : Purified by flash chromatography with hexane/ethyl acetate (1/2, v/v): 37 mg (99%, 0.10 mmol scale); pale yellow solid; m.p. 43.2 – 44.3 °C; $^1$H NMR ($CDCl_3$, 400 MHz): $\delta$ 7.93-7.88 (m, 2H), 7.71-7.66 (m, 3H), 7.57-7.49 (m, 7H), 7.46-7.43 (m, 1H), 6.36 (d, $J$ = 24.3 Hz, 1H); $^{13}$C{$^1$H} NMR ($CDCl_3$, 100 MHz): $\delta$ 159.1 (d, $^2J_{CP}$ = 15.8 Hz, 1C), 142.1 (d, $^2J_{CP}$ = 27.7 Hz, 1C), 134.8 (d, $^3J_{CP}$ = 16.6 Hz, 1C), 134.6 (d, $^1J_{CP}$ = 99.5 Hz, 1C), 134.2 (qd, $^4J_{CP}$/$^2J_{CF}$ = 3.0, 32.9 Hz, 1C), 133.3 (d, $^1J_{CP}$ = 105.6 Hz, 1C), 133.2 (d, $^4J_{CP}$ = 1.9 Hz, 1C), 131.7 (d, $^2J_{CP}$ = 11.1 Hz, 2C), 129.98 (1C), 129.97 (d, $^2J_{CP}$ = 10.6 Hz, 1C), 129.5 (d, $^3J_{CP}$ = 9.6 Hz, 1C), 129.0 (2C), 127.9 (2C), 125.8 (qd, $^3J_{CF}$/$^3J_{CP}$ = 3.7, 12.4 Hz, 2C), 124.5 (d, $^3J_{CP}$ = 11.3 Hz, 1C), 123.7 (q, $^1J_{CF}$ = 273.2 Hz, 1C), 122.1 (d, $^1J_{CP}$ = 100.0 Hz, 1C); $^{19}$F{$^1$H} NMR (376 MHz, $CDCl_3$) δ -63.2; $^{31}$P{$^1$H} NMR ($CDCl_3$, 162 MHz): $\delta$ 35.38; HRMS (APCI) m/z $(M+H)^+$ calcd for $C_{21}H_{15}F_3OP$: 371.0807, found: 371.0783.

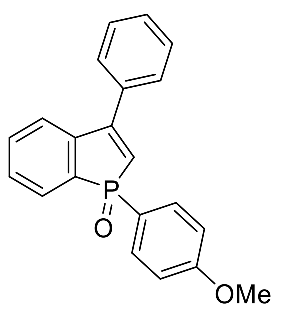

**1-(4-Methoxyphenyl)-3-phenylphosphindole 1-oxide (2l)** : Purified by flash chromatography with hexane/ethyl acetate (1/2, v/v): 18 mg (55%, 0.10 mmol scale); pale yellow oil; $^{1}$H NMR ($CDCl_3$, 400 MHz): $\delta$ 7.74-7.65 (m, 3H), 7.55-7.52 (m, 2H), 7.51-7.39 (m, 6H), 6.97-6.94 (m, 2H), 6.34 (d, $J$ = 23.9 Hz, 1H), 3.83 (s, 3H); $^{13}$C{$^{1}$H} NMR ($CDCl_3$, 100 MHz): $\delta$ 163.1 (d, $^{4}J_{CP}$ = 2.9 Hz, 1C), 157.7 (d, $^{2}J_{CP}$ = 15.3 Hz, 1C), 142.1 (d, $^{2}J_{CP}$ = 27.0 Hz, 1C), 135.3 (d, $^{3}J_{CP}$ = 16.1 Hz, 1C), 134.4 (d, $^{1}J_{CP}$ = 104.6 Hz, 1C), 133.0 (d, $^{2}J_{CP}$ = 12.0 Hz, 2C), 132.60 (d, $^{4}J_{CP}$ = 1.7 Hz, 1C), 129.63 (1C), 129.5 (d, $^{2}J_{CP}$ = 9.9 Hz, 1C), 129.3 (d, $^{3}J_{CP}$ = 9.7 Hz, 1C), 128.9 (2C), 128.0 (2C), 124.1 (d, $^{3}J_{CP}$ = 11.3 Hz, 1C), 123.5 (d, $^{1}J_{CP}$ = 99.3 Hz, 1C), 120.2 (d, $^{1}J_{CP}$ = 108.7 Hz, 1C), 114.7 (d, $^{3}J_{CP}$ = 13.6 Hz, 2C), 55.5 (1C); $^{31}$P{$^{1}$H} NMR ($CDCl_3$, 162 MHz): $\delta$ 37.19; HRMS (APCI) m/z $(M+H)^+$ calcd for $C_{21}H_{18}O_2P$: 333.1039, found: 333.1022.

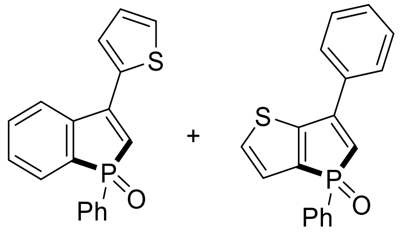

**1-Phenyl-3-(thiophen-2-yl)phosphindole 1-oxide + 4,6-Diphenylphospholo[3,2-*b*]thiophene 4-oxide (2m + 2m', 5:3 mixture)** : Purified by flash chromatography with hexane/ethyl acetate (1/2, v/v): 22 mg (70%, 0.10 mmol scale); pale yellow oil; $^{1}$H NMR ($CDCl_3$, 400 MHz): $\delta$ 7.81-7.63 (m, 5.2H), 7.57-7.52 (1.6H), 7.49-7.40 (m, 4.2H), 7.35 (dd, $J$ = 1.3, 5.0 Hz, 1H), 6.40 (d, $J$ = 23.6 Hz, 0.6H), 6.29 (dd, $J$ = 1.0, 26.2 Hz, 0.4H); $^{13}$C{$^{1}$H} NMR ($CDCl_3$, 100 MHz); $\delta$ 152.6, 142.2, 135.9, 135.7, 134.3, 133.3, 132.9, 132.5, 131.2, 131.1, 130.2, 129.9, 129.8, 129.6, 129.5, 129.2, 129.1, 129.02, 128.96, 127.6, 127.5, 126.8, 125.4, 124.1, 124.0, 122.2, 121.2 (All observed signals are just shown because of complexity associated with C–P couplings and structural isomers.); $^{31}$P{$^{1}$H} NMR ($CDCl_3$, 162 MHz) for mixture: $\delta$ 37.49, 28.99; HRMS (APCI) m/z $(M+H)^+$ calcd for $C_{18}H_{14}OPS$:309.0497, found: 309.0475.

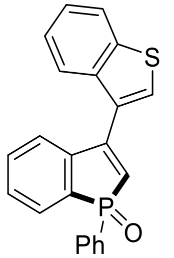


**3-(Benzo[*b*]thiophen-3-yl)-1-phenylphosphindole 1-oxide (2n)** : Purified by flash chromatography with hexane/ethyl acetate (1/2, v/v): 10 mg (28%, 0.10 mmol scale); pale yellow solid; m.p. 67.9 – 69.0 °C; $^{1}$H NMR ($CDCl_3$, 400 MHz): $\delta$ 7.97-7.94 (m, 1H), 7.87-7.81 (m, 3H), 7.75-7.71 (m, 1H), 7.69 (s, 1H), 7.58-7.54 (m, 1H), 7.75-7.39 (m, 7H), 6.56 (d, $J$ = 24.1 Hz, 1H); $^{13}$C{$^{1}$H} NMR ($CDCl_3$, 100 MHz): $\delta$ 151.7 (d, $^{2}J_{CP}$ = 15.9 Hz, 1C), 142.4 (d, $^{2}J_{CP}$ = 27.0 Hz, 1C), 140.5 (1C), 137.7 (1C), 133.6 (d, $^{1}J_{CP}$ = 104.4 Hz, 1C), 132.9 (d, $^{4}J_{CP}$ = 1.6 Hz, 1C), 132.5 (d, $^{4}J_{CP}$ = 2.9 Hz, 1C), 131.1 (d, $^{2}J_{CP}$ = 10.8 Hz, 2C), 130.8 (d, $^{3}J_{CP}$ = 17.3 Hz, 1C), 130.0 (d, $^{2}J_{CP}$ = 10.6 Hz, 1C), 129.6 (d, $^{1}J_{CP}$ = 102.7 Hz, 1C), 129.4 (d, $^{3}J_{CP}$ = 9.6 Hz, 1C), 129.1 (d, $^{3}J_{CP}$ = 12.4 Hz, 2C), 126.7 (1C), 125.3 (1C), 124.9 (1C), 124.3 (d, $^{1}J_{CP}$ = 97.9 Hz, 1C), 124.2 (d, $^{3}J_{CP}$ = 11.0 Hz, 1C), 123.3 (1C), 123.1 (1C); $^{31}$P{$^{1}$H} NMR ($CDCl_3$, 162 MHz): $\delta$ 37.42; HRMS (APCI) m/z $(M+H)^{+}$ calcd for $C_{22}H_{16}OPS$: 359.0654, found: 359.0631.

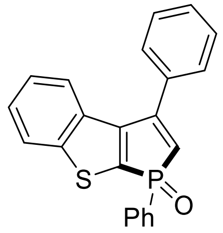


**1,3-Diphenylbenzo[*b*]phospholo[3,2-*d*]thiophene 1-oxide (2n')** : Purified by flash chromatography with hexane/ethyl acetate (1/2, v/v): 5 mg (14%, 0.10 mmol scale); pale yellow solid; m.p. 115.3 – 116.5 °C; $^{1}$H NMR ($CDCl_3$, 400 MHz): $\delta$ 7.91-7.89 (m, 1H), 7.85-7.79 (m, 2H), 7.59-7.52 (m, 6H), 7.49-7.44 (m, 2H), 7.40-7.36 (m, 2H), 7.26 (dd, $J$ = 1.1, 15.3 Hz, 1H), 6.24 (d, $J$ = 26.8 Hz, 1H); $^{13}$C{$^{1}$H} NMR ($CDCl_3$, 100 MHz): The clear spectra was not obtained because of a small amount of the sample.; $^{31}$P{$^{1}$H} NMR ($CDCl_3$, 162 MHz): $\delta$ 29.76; HRMS (APCI) m/z $(M+H)^{+}$ calcd for $C_{22}H_{16}OPS$: 359.0654, found: 359.0658.

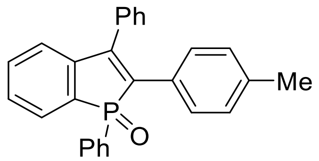


**1,3-Diphenyl-2-(*p*-tolyl)phosphindole 1-oxide (4aa):** pale yellow solid (0.2 mmol scale, 61.9 mg, 79% yield; 2.0 mmol scale, 510.0 mg, 65% yield); m.p. 159.7-161.0 °C (from hexane); TLC $R_f$ 0.25 (hexane/EtOAc, 1:2). $^{1}$H NMR ($CDCl_3$, 400 MHz) δ 7.80-7.75 (m, 2H), 7.71-7.67 (m, 1H), 7.48-7.32

(m, 10H), 7.18 (dd, $J$ = 7.6, 2.9 Hz, 1H), 7.14 (d, $J$ = 7.6 Hz, 2H), 6.89 (d, $J$ = 8.2 Hz, 2H), 2.18 (s, 3H). $^{13}C\{^1H\}$ NMR ($CDCl_3$, 100 MHz) δ 149.3 (d, $^2J_{CP}$ = 21.8 Hz, 1C), 143.9 (d, $^2J_{CP}$ = 27.2 Hz, 1C), 137.8 (1C), 134.5 (d, $^3J_{CP}$ = 15.4 Hz, 1C), 134.1 (d, $^1J_{CP}$ = 95.7 Hz, 1C), 132.9 (d, $^4J_{CP}$ = 1.8 Hz, 1C), 132.1 (d, $^4J_{CP}$ = 2.5 Hz, 1C), 132.1 (d, $^1J_{CP}$ = 105.6 Hz, 1C), 131.0 (d, $^2J_{CP}$ = 10.7 Hz, 2C), 130.1 (d, $^1J_{CP}$ = 99.6 Hz, 1C), 129.7 (d, $^2J_{CP}$ = 10.1 Hz, 1C), 129.1 (4C), 129.0 (2C), 128.9 (2C), 128.89 (2C), 128.85 (d, $^3J_{CP}$ = 12.2 Hz, 2C), 128.6 (1C), 123.9 (d, $^2J_{CP}$ = 10.9 Hz, 1C), 21.2 (1C). $^{31}P\{^1H\}$ NMR ($CDCl_3$, 162 MHz) δ 39.2; HRMS (APCI) m/z ($[M+H]^+$) Calcd for $C_{27}H_{22}OP$: 393.1408, Found: 393.1407.

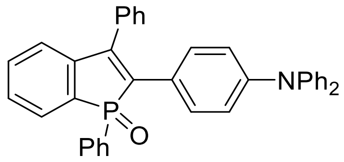

**2-(4-(Diphenylamino)phenyl)-1,3-diphenylphosphindole 1-oxide (4ab):** yellow solid (0.2 mmol scale, 94.8 mg, 87% yield; 1.0 mmol scale, 430.7 mg, 79% yield); TLC $R_f$ 0.20 (hexane/EtOAc, 1:2). $^1H$ NMR ($CDCl_3$, 400 MHz) δ 7.85-7.80 (m, 2H), 7.68-7.64 (m, 1H), 7.52-7.29 (m, 10H), 7.21-7.17 (m, 4H), 7.10 (dd, $J$ = 7.5, 2.8 Hz, 1H), 7.07-6.97 (m, 8H), 6.72 (d, $J$ = 8.8 Hz, 2H). $^{13}C\{^1H\}$ NMR ($CDCl_3$, 100 MHz) δ 147.8 (d, $^2J_{CP}$ = 21.8 Hz, 1C), 147.4 (1C), 147.1 (2C), 144.3 (d, $^2J_{CP}$ = 27.2 Hz, 1C), 135.0 (d, $^3J_{CP}$ = 15.4 Hz, 1C), 133.5 (d, $^1J_{CP}$ = 95.7 Hz, 1C), 132.9 (d, $^4J_{CP}$ = 1.8 Hz, 1C), 132.1 (d, $^4J_{CP}$ = 2.7 Hz, 1C), 131.8 (d, $^1J_{CP}$ = 106.1 Hz, 1C), 131.0 (d, $^2J_{CP}$ = 10.5 Hz, 2C), 130.7 (d, $^1J_{CP}$ = 98.6 Hz, 1C), 129.9 (d, $^3J_{CP}$ = 6.4 Hz, 2C), 129.3 (4C), 129.1 (2C), 128.95 (2C), 128.93 (d, $^3J_{CP}$ = 12.0 Hz, 2C), 128.91 (1C), 128.8 (d, $^3J_{CP}$ = 10.5 Hz, 1C), 128.6 (1C), 125.7 (d, $^2J_{CP}$ = 10.3 Hz, 1C), 125.1 (4C), 123.6 (d, $^2J_{CP}$ = 10.9 Hz, 1C), 123.4 (2C), 121.6 (2C). $^{31}P\{^1H\}$ NMR ($CDCl_3$, 162 MHz) δ 39.3; HRMS (APCI) m/z ($[M+H]^+$) Calcd for $C_{38}H_{29}NOP$: 546.1982, Found: 546.2011.

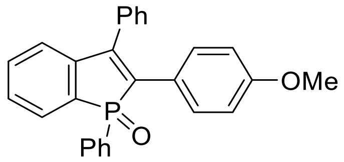

**2-(4-Methoxyphenyl)-1,3-diphenylphosphindole 1-oxide (4ac):** pale yellow solid (0.2 mmol, 60.6 mg, 74% yield); TLC $R_f$ 0.24 (hexane/EtOAc, 1:2). $^1H$ NMR ($CDCl_3$, 400 MHz) δ 7.80-7.75 (m, 2H), 7.70-7.66 (m, 1H), 7.48-7.30 (m, 10H), 7.21-7.19 (m, 2H), 7.15 (dd, $J$ = 7.6, 2.9 Hz, 1H), 6.62 (d, $J$ = 8.8 Hz, 2H), 3.67 (s, 3H). $^{13}C\{^1H\}$ NMR ($CDCl_3$, 100 MHz) δ 159.2 (1C), 148.3 (d, $^2J_{CP}$ = 21.9 Hz, 1C), 144.1 (d, $^2J_{CP}$ = 27.2 Hz, 1C), 134.7 (d, $^3J_{CP}$ = 15.4 Hz, 1C), 133.6 (d, $^1J_{CP}$ = 96.0 Hz, 1C), 132.9 (d, $^4J_{CP}$ = 1.5 Hz, 1C), 132.1 (d, $^4J_{CP}$ = 2.7 Hz, 1C), 131.8 (d, $^1J_{CP}$ = 105.6 Hz, 1C), 130.9 (d, $^2J_{CP}$ = 10.5 Hz, 2C), 130.4 (d, $^3J_{CP}$ = 6.0 Hz, 2C), 130.3 (d, $^1J_{CP}$ = 99.2 Hz, 1C), 129.09 (2C), 129.06 (2C), 128.9 (d, $^3J_{CP}$ = 9.8 Hz, 1C), 128.8 (d, $^3J_{CP}$ = 12.0 Hz, 2C), 128.7 (d, $^3J_{CP}$ = 10.4 Hz, 1C), 128.6 (1C),

125.1 (d, $^2J_{CP}$ = 10.3 Hz, 1C), 123.7 (d, $^2J_{CP}$ = 10.9 Hz, 1C), 113.8 (2C), 55.1 (1C). $^{31}P\{^1H\}$ NMR ($CDCl_3$, 162 MHz) δ 39.2; HRMS (APCI) m/z ($[M+H]^+$) Calcd for $C_{27}H_{22}O_2P$: 409.1352, Found: 409.1367.

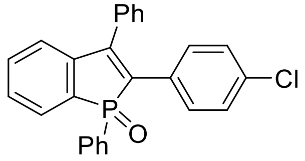


**2-(4-Chlorophenyl)-1,3-diphenylphosphindole 1-oxide (4ad):** colorless solid (0.2 mmol, 66.7 mg, 81% yield); m.p. 104.6-105.9 °C (from hexane); TLC $R_f$ 0.25 (hexane/EtOAc, 1:2). $^1H$ NMR ($CDCl_3$, 400 MHz) δ 7.77-7.69 (m, 3H), 7.51-7.36 (m, 8H), 7.32-7.30 (m, 2H), 7.21 (dd, $J$ = 7.5, 2.9 Hz, 1H), 7.19-7.16 (m, 2H), 7.07-7.05 (m, 2H). $^{13}C\{^1H\}$ NMR ($CDCl_3$, 100 MHz) δ 150.5 (d, $^2J_{CP}$ = 21.1 Hz, 1C), 143.6 (d, $^2J_{CP}$ = 26.7 Hz, 1C), 133.9 (d, $^3J_{CP}$ = 14.7 Hz, 1C), 133.8 (1C), 133.1 (d, $^1J_{CP}$ = 95.7 Hz, 1C), 133.0 (d, $^4J_{CP}$ = 1.9 Hz, 1C), 132.4 (1C), 132.3 (d, $^4J_{CP}$ = 2.9 Hz, 1C), 131.9 (d, $^1J_{CP}$ = 105.3 Hz, 1C), 131.3 (1C), 130.9 (d, $^2J_{CP}$ = 10.8 Hz, 2C), 130.6 (d, $^1J_{CP}$ = 112.6 Hz, 1C), 130.3 (d, $^3J_{CP}$ = 5.7 Hz, 2C), 129.3 (d, $^2J_{CP}$ = 10.7 Hz, 1C), 129.15 (d, $^3J_{CP}$ = 9.7 Hz, 1C), 129.14 (2C), 128.96 (d, $^3J_{CP}$ = 12.2 Hz, 2C), 128.93 (2C), 128.6 (2C), 124.2 (d, $^2J_{CP}$ = 10.7 Hz, 1C). $^{31}P\{^1H\}$ NMR ($CDCl_3$, 162 MHz) δ 39.0; HRMS (APCI) m/z ($[M+H]^+$) Calcd for $C_{26}H_{19}ClOP$: 413.0857, Found: 413.0878.

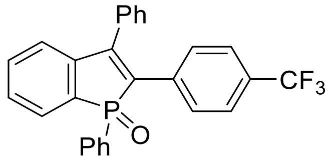


**1,3-Diphenyl-2-(4-(trifluoromethyl)phenyl)phosphindole 1-oxide (4ae):** colorless solid (0.2 mmol, 45.5 mg, 51% yield); m.p. 94.3-95.6 °C (from hexane); TLC $R_f$ 0.23 (hexane/EtOAc, 1:2). $^1H$ NMR ($CDCl_3$, 400 MHz) δ 7.78-7.71 (m, 3H), 7.53-7.39 (m, 8H), 7.34-7.31 (m, 6H), 7.25 (dd, $J$ = 6.5, 1.8 Hz, 1H). $^{13}C\{^1H\}$ NMR ($CDCl_3$, 100 MHz) δ 151.8 (d, $^2J_{CP}$ = 20.9 Hz, 1C), 143.3 (d, $^2J_{CP}$ = 26.4 Hz, 1C), 136.5 (d, $^2J_{CP}$ = 10.2 Hz, 1C), 133.6 (d, $^3J_{CP}$ = 14.5 Hz, 1C), 133.1 (d, $^4J_{CP}$ = 1.8 Hz, 1C), 133.0 (d, $^1J_{CP}$ = 95.8 Hz, 1C), 132.5 (d, $^4J_{CP}$ = 2.9 Hz, 1C), 132.0 (d, $^1J_{CP}$ = 106.3 Hz, 1C), 130.9 (d, $^2J_{CP}$ = 10.7 Hz, 2C), 129.6 (d, $^3J_{CP}$ = 10.8 Hz, 1C), 129.5 (q, $^2J_{CF}$ = 32.2 Hz, 1C), 129.4 (1C), 129.3 (d, $^1J_{CP}$ = 99.9 Hz, 1C), 129.2 (2C), 129.18 (2C), 129.10 (2C), 128.93 (d, $^3J_{CP}$ = 12.4 Hz, 2C), 128.90 (1C), 125.2 (q, $^3J_{CF}$ = 3.8 Hz, 2C), 124.5 (d, $^2J_{CP}$ = 10.9 Hz, 1C), 124.0 (q, $^1J_{CF}$ = 272.1 Hz, 1C). $^{19}F\{^1H\}$ NMR ($CDCl_3$, 376 MHz) δ -62.8. $^{31}P\{^1H\}$ NMR ($CDCl_3$, 162 MHz) δ 39.0; HRMS (APCI) m/z ($[M+H]^+$) Calcd for $C_{27}H_{19}F_3OP$: 447.1121, Found: 447.1131.

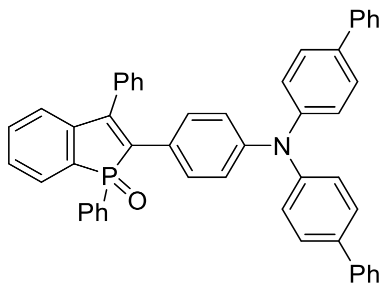

**2-(4-(Di([1,1'-biphenyl]-4-yl)amino)phenyl)-1,3-diphenylphosphindole 1-oxide (4af):** orange solid (0.2 mmol, 92.1 mg, 66% yield); m.p. 151.7-153.0 °C (from hexane); TLC $R_f$ 0.20 (hexane/EtOAc, 1:2). $^1$H NMR ($CDCl_3$, 400 MHz) δ 7.87-7.82 (m, 2H), 7.66 (dd, $J$ = 9.2, 7.3 Hz, 1H), 7.54-7.52 (m, 4H), 7.50-7.37 (m, 17H), 7.33-7.27 (m, 3H), 7.13-7.08 (m, 7H), 6.83 (d, $J$ = 8.8 Hz, 2H). $^{13}$C{$^1$H} NMR ($CDCl_3$, 100 MHz) δ 148.1 (d, $^2J_{CP}$ = 22.0 Hz, 1C), 147.4 (1C), 146.2 (2C), 144.3 (d, $^2J_{CP}$ = 27.1 Hz, 1C), 140.5 (2C), 136.2 (2C), 135.0 (d, $^3J_{CP}$ = 15.2 Hz, 1C), 133.4 (d, $^1J_{CP}$ = 95.7 Hz, 1C), 133.0 (d, $^4J_{CP}$ = 1.6 Hz, 1C), 132.2 (d, $^4J_{CP}$ = 2.5 Hz, 1C), 131.9 (d, $^1J_{CP}$ = 106.2 Hz, 1C), 131.0 (d, $^2J_{CP}$ = 10.7 Hz, 2C), 130.7 (d, $^1J_{CP}$ = 98.6 Hz, 1C), 130.1 (d, $^3J_{CP}$ = 6.3 Hz, 2C), 129.2 (2C), 129.0 (d, $^3J_{CP}$ = 12.2 Hz, 2C), 128.97 (2C), 128.92 (d, $^3J_{CP}$ = 10.3 Hz, 1C), 128.79 (4C), 128.78 (1C), 128.73 (d, $^3J_{CP}$ = 9.5 Hz, 1C), 127.9 (4C), 127.0 (2C), 126.8 (4C), 126.3 (d, $^2J_{CP}$ = 10.0 Hz, 1C), 125.1 (4C), 123.7 (d, $^2J_{CP}$ = 10.7 Hz, 1C), 122.2 (2C). $^{31}$P{$^1$H} NMR ($CDCl_3$, 162 MHz) δ 39.3; HRMS (APCI) m/z ($[M+H]^+$) Calcd for $C_{50}H_{37}NOP$: 698.2608, Found: 698.2635.

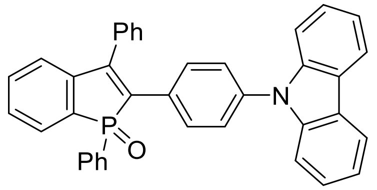

**2-(4-(9*H*-Carbazol-9-yl)phenyl)-1,3-diphenylphosphindole 1-oxide (4ag):** yellow solid (0.2 mmol, 76.0 mg, 70% yield); m.p. 135.7-137.0 °C (from hexane); TLC $R_f$ 0.22 (hexane/EtOAc, 1:2). $^1$H NMR ($CDCl_3$, 400 MHz) δ 8.07 (d, $J$ = 7.7 Hz, 2H), 7.90-7.84 (m, 2H), 7.74 (dd, $J$ = 9.4, 7.2 Hz, 1H), 7.56-7.38 (m, 12H), 7.37-7.30 (m, 6H), 7.25-7.21 (m, 3H). $^{13}$C{$^1$H} NMR ($CDCl_3$, 100 MHz) δ 150.6 (d, $^2J_{CP}$ = 21.2 Hz, 1C), 143.7 (d, $^2J_{CP}$ = 26.7 Hz, 1C), 140.4 (2C), 137.2 (1C), 134.2 (d, $^3J_{CP}$ = 14.8 Hz, 1C), 133.4 (d, $^1J_{CP}$ = 96.2 Hz, 1C), 133.1 (d, $^4J_{CP}$ = 1.7 Hz, 1C), 132.4 (d, $^4J_{CP}$ = 2.8 Hz, 1C), 132.1 (d, $^1J_{CP}$ = 106.1 Hz, 1C), 131.6 (d, $^2J_{CP}$ = 10.0 Hz, 1C), 131.0 (d, $^2J_{CP}$ = 10.8 Hz, 2C), 130.5 (d, $^3J_{CP}$ = 6.0 Hz, 2C), 130.0 (d, $^1J_{CP}$ = 98.5 Hz, 1C), 129.4 (d, $^3J_{CP}$ = 10.8 Hz, 1C), 129.2 (2C), 129.1 (d, $^3J_{CP}$ = 12.7 Hz, 2C), 129.09 (1C), 129.03 (3C), 126.5 (2C), 125.9 (2C), 124.3 (d, $^2J_{CP}$ = 10.8 Hz, 1C), 123.5 (2C), 120.3 (2C), 120.1 (2C), 109.9 (2C). $^{31}$P{$^1$H} NMR ($CDCl_3$, 162 MHz) δ 39.1; HRMS (APCI) m/z ($[M+H]^+$) Calcd for $C_{38}H_{27}NOP$: 544.1825, Found: 544.1850.

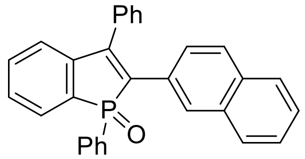

**2-(Naphthalen-2-yl)-1,3-diphenylphosphindole 1-oxide (4ah):** colorless solid (0.2 mmol, 69.3 mg, 81% yield); m.p. 92.7-94.3 °C (from hexane); TLC $R_f$ 0.25 (hexane/EtOAc, 1:2). $^1$H NMR ($CDCl_3$, 400 MHz) δ 7.97 (bs, 1H), 7.83-7.72 (m, 3H), 7.66-7.63 (m, 2H), 7.50-7.34 (m, 13H), 7.26 (dd, $J$ = 7.6, 2.9 Hz, 1H), 7.14 (dd, $J$ = 8.6, 0.9 Hz, 1H). $^{13}$C{$^1$H} NMR ($CDCl_3$, 100 MHz) δ 150.3 (d, $^2J_{CP}$ = 21.1 Hz, 1C), 143.7 (d, $^2J_{CP}$ = 26.8 Hz, 1C), 134.3 (d, $^3J_{CP}$ = 14.8 Hz, 1C), 134.1 (d, $^1J_{CP}$ = 96.2 Hz, 1C), 133.1 (1C), 133.0 (d, $^4J_{CP}$ = 1.9 Hz, 1C), 132.6 (1C), 132.2 (d, $^4J_{CP}$ = 2.8 Hz, 1C), 132.1 (d, $^1J_{CP}$ = 106.0 Hz, 1C), 130.9 (d, $^2J_{CP}$ = 10.3 Hz, 2C), 130.4 (1C), 129.9 (d, $^1J_{CP}$ = 90.1 Hz, 1C), 129.19 (2C), 129.18 (d, $^2J_{CP}$ = 10.5 Hz, 1C), 129.10 (1C), 129.05 (2C), 128.9 (d, $^3J_{CP}$ = 12.5 Hz, 2C), 128.8 (1C), 128.7 (d, $^3J_{CP}$ = 5.4 Hz, 1C), 128.5 (1C), 127.7 (1C), 127.4 (1C), 126.6 (d, $^3J_{CP}$ = 6.2 Hz, 1C), 126.4 (1C), 126.0 (1C), 124.1 (d, $^2J_{CP}$ = 11.0 Hz, 1C). $^{31}$P{$^1$H} NMR ($CDCl_3$, 162 MHz) δ 39.2; HRMS (APCI) m/z ([M+H]$^+$) Calcd for $C_{30}H_{22}OP$: 429.1403, Found: 429.1419.

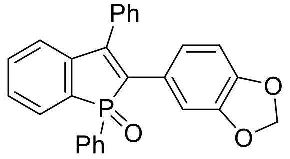

**2-(Benzo[*d*][1,3]dioxol-5-yl)-1,3-diphenylphosphindole 1-oxide (4ai):** pale yellow solid (0.2 mmol, 57.4 mg, 68% yield); m.p. 209.1-210.5 °C (from hexane); TLC $R_f$ 0.22 (hexane/EtOAc, 1:2). $^1$H NMR ($CDCl_3$, 400 MHz) δ 7.79-7.74 (m, 2H), 7.70-7-66 (m, 1H), 7.51-7.38 (m, 7H), 7.35-7.32 (m, 3H), 7.16 (dd, $J$ = 7.5, 2.9 Hz, 1H), 6.91 (dt, $J$ = 8.2, 1.6 Hz, 1H), 6.61 (dd, $J$ = 1.3, 1.0 Hz, 1H), 6.57 (d, $J$ = 8.2 Hz, 1H), 5.81 (d, $J$ = 1.5 Hz, 1H), 5.80 (d, $J$ = 1.5 Hz, 1H). $^{13}$C{$^1$H} NMR ($CDCl_3$, 100 MHz) δ 148.9 (d, $^2J_{CP}$ = 21.6 Hz, 1C), 147.4 (1C), 147.3 (1C), 143.9 (d, $^2J_{CP}$ = 26.7 Hz, 1C), 134.4 (d, $^3J_{CP}$ = 14.9 Hz, 1C), 133.8 (d, $^1J_{CP}$ = 96.4 Hz, 1C), 132.9 (d, $^4J_{CP}$ = 1.8 Hz, 1C), 132.2 (d, $^4J_{CP}$ = 2.7 Hz, 1C), 131.8 (d, $^1J_{CP}$ = 106.0 Hz, 1C), 130.9 (d, $^2J_{CP}$ = 10.8 Hz, 2C), 130.0 (d, $^1J_{CP}$ = 99.7 Hz, 1C), 129.1 (1C), 129.05 (1C), 129.0 (2C), 128.95 (2C), 128.90 (1C), 128.8 (d, $^3J_{CP}$ = 10.1 Hz, 2C), 126.5 (d, $^2J_{CP}$ = 10.4 Hz, 1C), 123.8 (d, $^2J_{CP}$ = 11.0 Hz, 1C), 123.5 (d, $^3J_{CP}$ = 5.8 Hz, 1C), 109.2 (d, $^3J_{CP}$ = 6.3 Hz, 1C), 108.3 (1C), 100.9 (1C) . $^{31}$P{$^1$H} NMR ($CDCl_3$, 162 MHz) δ 39.1; HRMS (APCI) m/z ([M+H]$^+$) Calcd for $C_{27}H_{20}O_3P$: 423.1145, Found: 423.1155.

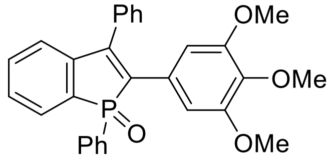

**1,3-Diphenyl-2-(3,4,5-trimethoxyphenyl)phosphindole 1-oxide (4aj):** pale yellow solid (0.2 mmol, 64.6 mg, 69% yield); m.p. 197.1-198.4 °C (from hexane); TLC $R_f$ 0.20 (hexane/EtOAc, 1:2). $^{1}$H NMR ($CDCl_3$, 400 MHz) δ 7.85-7.80 (m, 2H), 7.69 (dd, $J$ = 9.2, 7.1 Hz, 1H), 7.54-7.40 (m, 7H), 7.39-7.34 (m, 3H), 7.17 (dd, $J$ = 7.5, 2.9 Hz, 1H), 6.45 (d, $J$ = 0.7 Hz, 2H), 3.74 (s, 3H), 3.44 (s, 6H). $^{13}$C{$^{1}$H} NMR ($CDCl_3$, 100 MHz) δ 152.7 (2C), 149.2 (d, $^{2}J_{CP}$ = 21.5 Hz, 1C), 144.0 (d, $^{2}J_{CP}$ = 26.8 Hz, 1C), 137.7 (1C), 134.8 (d, $^{3}J_{CP}$ = 14.8 Hz, 1C), 134.0 (d, $^{1}J_{CP}$ = 96.5 Hz, 1C), 133.0 (d, $^{4}J_{CP}$ = 1.5 Hz, 1C), 132.2 (d, $^{4}J_{CP}$ = 2.6 Hz, 1C), 131.7 (d, $^{1}J_{CP}$ = 106.3 Hz, 1C), 131.0 (d, $^{2}J_{CP}$ = 10.7 Hz, 2C), 130.4 (d, $^{1}J_{CP}$ = 99.0 Hz, 1C), 129.2 (2C), 129.1 (d, $^{3}J_{CP}$ = 10.1 Hz, 1C), 129.0 (1C), 128.97 (2C), 128.95 (d, $^{3}J_{CP}$ = 12.2 Hz, 2C), 128.6 (1C), 127.8 (d, $^{2}J_{CP}$ = 10.4 Hz, 1C), 123.9 (d, $^{2}J_{CP}$ = 10.9 Hz, 1C), 106.4 (d, $^{3}J_{CP}$ = 6.4 Hz, 2C), 60.7 (1C), 55.5 (2C). $^{31}$P{$^{1}$H} NMR ($CDCl_3$, 162 MHz) δ 38.7; HRMS (APCI) m/z ([M+H]$^{+}$) Calcd for $C_{29}H_{26}O_4P$: 469.1564, Found: 469.1543.

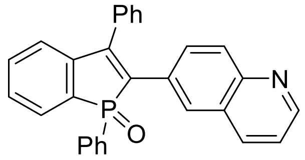


**1,3-Diphenyl-2-(quinolin-6-yl)phosphindole 1-oxide (4ak):** orange solid (0.2 mmol, 53.2 mg, 62% yield); m.p. 111.4-112.7 °C (from hexane); TLC $R_f$ 0.24 (hexane/EtOAc, 1:3). $^{1}$H NMR ($CDCl_3$, 400 MHz) δ 8.80 (dd, $J$ = 4.1, 1.5 Hz, 1H), 8.03 (s, 1H), 7.99 (d, $J$ = 8.4 Hz, 1H), 7.82-7.72 (m, 4H), 7.52-7.36 (m, 10H), 7.32-7.28 (m, 3H). $^{13}$C{$^{1}$H} NMR ($CDCl_3$, 100 MHz) δ 151.0 (d, $^{2}J_{CP}$ = 20.8 Hz, 1C), 150.7 (1C), 147.5 (1C), 143.5 (d, $^{2}J_{CP}$ = 26.7 Hz, 1C), 136.4 (1C), 133.9 (d, $^{3}J_{CP}$ = 14.5 Hz, 1C), 133.5 (d, $^{1}J_{CP}$ = 95.7 Hz, 1C), 133.1 (d, $^{4}J_{CP}$ = 1.6 Hz, 1C), 132.4 (d, $^{4}J_{CP}$ = 2.8 Hz, 1C), 132.0 (d, $^{4}J_{CP}$ = 1.9 Hz, 1C), 132.2 (d, $^{4}J_{CP}$ = 2.8 Hz, 1C), 132.0 (d, $^{1}J_{CP}$ = 106.2 Hz, 1C), 131.3 (1C), 130.9 (d, $^{2}J_{CP}$ = 10.5 Hz, 2C), 130.7 (d, $^{1}J_{CP}$ = 108.4 Hz, 1C), 130.3 (d, $^{3}J_{CP}$ = 6.1 Hz, 1C), 129.4 (d, $^{2}J_{CP}$ = 10.8 Hz, 1C), 129.3 (1C), 129.2 (2C), 129.1 (1C), 129.0 (1C), 128.9 (d, $^{3}J_{CP}$ = 12.3 Hz, 2C), 128.3 (d, $^{3}J_{CP}$ = 5.2 Hz, 1C), 128.1 (1C), 124.3 (d, $^{2}J_{CP}$ = 10.8 Hz, 1C), 121.3 (1C). $^{31}$P{$^{1}$H} NMR ($CDCl_3$, 162 MHz) δ 39.1; HRMS (APCI) m/z ([M+H]$^{+}$) Calcd for $C_{29}H_{21}NOP$: 430.1356, Found: 430.1362.

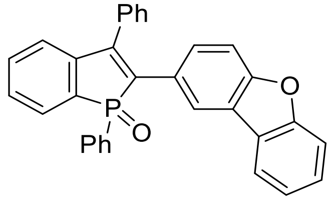


**2-(Dibenzo[*b*,*d*]furan-2-yl)-1,3-diphenylphosphindole 1-oxide (4al):** pale yellow solid (0.2 mmol, 67.4 mg, 72% yield); m.p. 108.9-109.6 °C (from hexane); TLC $R_f$ 0.22 (hexane/EtOAc, 1:2). $^{1}$H NMR ($CDCl_3$, 400 MHz) δ 7.89 (s, 1H), 7.83-7.70 (m, 4H), 7.50-7.36 (m, 12H), 7.31-7.24 (m, 4H). $^{13}$C{$^{1}$H} NMR ($CDCl_3$, 100 MHz) δ 156.4 (1C), 155.6 (1C), 149.6 (d, $^{2}J_{CP}$ = 21.7 Hz, 1C), 143.8 (d, $^{2}J_{CP}$ = 26.8 Hz, 1C), 134.3 (d, $^{3}J_{CP}$ = 14.9 Hz, 1C), 134.2 (d, $^{1}J_{CP}$ = 96.2 Hz, 1C), 133.0 (d, $^{4}J_{CP}$ = 1.9 Hz,

1C), 132.2 (d, $^4J_{CP}$ = 2.8 Hz, 1C), 131.9 (d, $^1J_{CP}$ = 106.1 Hz, 1C), 131.0 (d, $^2J_{CP}$ = 10.6 Hz, 2C), 129.9 (d, $^1J_{CP}$ = 99.8 Hz, 1C), 129.18 (2C), 129.17 (d, $^3J_{CP}$ = 9.6 Hz, 1C), 129.09 (2C), 129.06 (1C), 128.9 (d, $^3J_{CP}$ = 12.3 Hz, 2C), 128.7 (1C), 128.5 (d, $^3J_{CP}$ = 5.9 Hz, 1C), 127.5 (d, $^2J_{CP}$ = 10.0 Hz, 1C), 127.3 (1C), 124.3 (1C), 124.0 (d, $^2J_{CP}$ = 10.7 Hz, 1C), 123.9 (1C), 122.8 (1C), 121.3 (d, $^4J_{CP}$ = 5.6 Hz, 1C), 120.8 (1C), 111.6 (2C). $^{31}$P{$^1$H} NMR ($CDCl_3$, 162 MHz) δ 39.1; HRMS (APCI) m/z ([M+H]$^+$) Calcd for $C_{32}H_{22}O_2P$: 469.1352, Found: 469.1366.

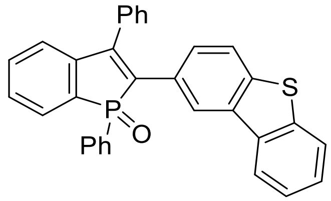


**2-(Dibenzo[*b*,*d*]thiophen-2-yl)-1,3-diphenylphosphindole 1-oxide (4am):** yellow solid (0.2 mmol, 66.8 mg, 69% yield); m.p. 108.0-109.3 °C (from hexane); TLC $R_f$ 0.22 (hexane/EtOAc, 1:2). $^1$H NMR ($CDCl_3$, 400 MHz) δ 8.03 (s, 1H), 7.86-7.81 (m, 2H), 7.78-7.74 (m, 3H), 7.55 (d, *J* = 8.4 Hz, 1H), 7.51-7.34 (m, 13H), 7.27 (dd, *J* = 7.5, 3.0 Hz, 1H). $^{13}$C{$^1$H} NMR ($CDCl_3$, 100 MHz) δ 149.9 (d, $^2J_{CP}$ = 21.7 Hz, 1C), 143.9 (d, $^2J_{CP}$ = 26.9 Hz, 1C), 139.5 (1C), 139.1 (1C), 135.5 (1C), 135.3 (1C), 134.4 (d, $^3J_{CP}$ = 14.9 Hz, 1C), 134.1 (d, $^1J_{CP}$ = 96.0 Hz, 1C), 133.0 (d, $^4J_{CP}$ = 1.8 Hz, 1C), 132.3 (d, $^4J_{CP}$ = 2.8 Hz, 1C), 132.0 (d, $^1J_{CP}$ = 106.3 Hz, 1C), 131.0 (d, $^2J_{CP}$ = 10.6 Hz, 2C), 130.0 (d, $^1J_{CP}$ = 99.6 Hz, 1C), 129.2 (2C), 129.16 (d, $^2J_{CP}$ = 10.8 Hz, 1C), 129.11 (d, $^3J_{CP}$ = 9.7 Hz, 1C), 129.10 (3C), 128.9 (d, $^3J_{CP}$ = 12.3 Hz, 2C), 128.8 (1C), 127.6 (d, $^3J_{CP}$ = 5.6 Hz, 1C), 126.8 (1C), 124.4 (1C), 124.1 (d, $^2J_{CP}$ = 10.7 Hz, 1C), 122.7 (1C), 122.6 (1C), 122.1 (d, $^3J_{CP}$ = 6.2 Hz, 1C), 121.6 (1C). $^{31}$P{$^1$H} NMR ($CDCl_3$, 162 MHz) δ 39.1; HRMS (APCI) m/z ([M+H]$^+$) Calcd for $C_{32}H_{22}OPS$: 485.1124, Found: 485.1130.

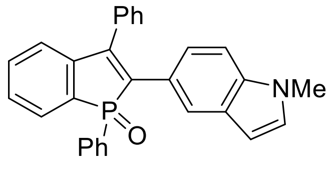


**2-(1-Methyl-1*H*-indol-5-yl)-1,3-diphenylphosphindole 1-oxide (4an):** yellow solid (0.2 mmol, 56.0 mg, 65% yield); m.p. 121.4-122.7 °C (from hexane); TLC $R_f$ 0.24 (hexane/EtOAc, 1:2). $^1$H NMR ($CDCl_3$, 400 MHz) δ 7.82-7.77 (m, 2H), 7.72-7.68 (m, 2H), 7.44-7.30 (m, 10H), 7.19 (dd, *J* = 7.6, 2.8 Hz, 1H), 7.06 (d, *J* = 8.5 Hz, 1H), 6.99 (d, *J* = 8.5 Hz, 1H), 6.91 (d, *J* = 3.0 Hz, 1H), 6.31 (d, *J* = 3.0 Hz, 1H), 3.63 (s, 3H). $^{13}$C{$^1$H} NMR ($CDCl_3$, 100 MHz) δ 147.8 (d, $^2J_{CP}$ = 22.2 Hz, 1C), 144.2 (d, $^2J_{CP}$ = 27.6 Hz, 1C), 136.1 (1C), 135.1 (d, $^1J_{CP}$ = 95.6 Hz, 1C), 135.0 (d, $^3J_{CP}$ = 15.1 Hz, 1C), 132.8 (d, $^4J_{CP}$ = 1.3 Hz, 1C), 132.1 (d, $^1J_{CP}$ = 105.4 Hz, 1C), 131.9 (d, $^4J_{CP}$ = 2.8 Hz, 1C), 131.0 (d, $^2J_{CP}$ = 10.6 Hz, 2C), 130.5 (d, $^1J_{CP}$ = 99.7 Hz, 1C), 129.3 (2C), 129.1 (1C), 129.0 (2C), 128.9 (d, $^3J_{CP}$ = 9.5 Hz, 1C), 128.7 (d, $^3J_{CP}$ = 12.2 Hz, 2C), 128.5 (d, $^2J_{CP}$ = 10.4 Hz, 1C), 128.4 (1C), 128.2 (1C),

123.8 (d, $^3J_{CP}$ = 10.2 Hz, 1C), 123.6 (d, $^2J_{CP}$ = 11.0 Hz, 1C), 123.1 (d, $^3J_{CP}$ = 6.3 Hz, 1C), 122.1 (d, $^3J_{CP}$ = 5.9 Hz, 1C), 109.1 (1C), 101.7 (1C), 32.8 (1C). $^{31}P\{^1H\}$ NMR ($CDCl_3$, 162 MHz) δ 39.3; HRMS (APCI) m/z ($[M+H]^+$) Calcd for $C_{29}H_{23}NOP$: 432.1512, Found: 432.1534.

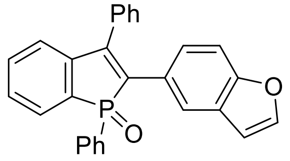

**2-(Benzofuran-5-yl)-1,3-diphenylphosphindole 1-oxide (4ao):** pale yellow solid (0.2 mmol, 46.8 mg, 56% yield); m.p. 202.8-204.1 °C (from hexane); TLC $R_f$ 0.25 (hexane/EtOAc, 1:2). $^1H$ NMR ($CDCl_3$, 400 MHz) δ 7.80-7.70 (m, 3H), 7.66 (s, 1H), 7.49 (d, $J$ = 2.3 Hz, 1H), 7.47-7.33 (m, 10H), 7.23 (dd, $J$ = 7.5, 2.9 Hz, 1H), 7.18 (dd, $J$ = 8.7, 0.6 Hz, 1H), 7.07 (dt, $J$ = 8.7, 1.4 Hz, 1H), 6.59 (dd, $J$ = 2.2, 0.9 Hz, 1H). $^{13}C\{^1H\}$ NMR ($CDCl_3$, 100 MHz) δ 154.4 (1C), 149.4 (d, $^2J_{CP}$ = 21.6 Hz, 1C), 145.2 (1C), 143.8 (d, $^2J_{CP}$ = 27.1 Hz, 1C), 134.4 (d, $^1J_{CP}$ = 95.6 Hz, 1C ), 134.3 (d, $^3J_{CP}$ = 15.4 Hz, 1C), 132.9 (d, $^4J_{CP}$ = 1.7 Hz, 1C), 132.2 (d, $^4J_{CP}$ = 2.8 Hz, 1C), 132.0 (d, $^1J_{CP}$ = 105.5 Hz, 1C), 131.0 (d, $^2J_{CP}$ = 10.6 Hz, 2C), 129.9 (d, $^1J_{CP}$ = 99.5 Hz, 1C), 129.2 (2C), 129.1 (1C), 129.05 (1C), 129.01 (2C), 128.9 (1C), 128.8 (d, $^3J_{CP}$ = 12.2 Hz, 2C), 128.6 (1C), 127.4 (d, $^2J_{CP}$ = 10.0 Hz, 1C), 125.7 (d, $^3J_{CP}$ = 6.2 Hz, 1C), 123.9 (d, $^2J_{CP}$ = 10.9 Hz, 1C), 122.1 (d, $^3J_{CP}$ = 5.3 Hz, 1C), 111.3 (1C), 106.9 (1C). $^{31}P\{^1H\}$ NMR ($CDCl_3$, 162 MHz) δ 39.1; HRMS (APCI) m/z ($[M+H]^+$) Calcd for $C_{28}H_{20}O_2P$: 419.1196, Found: 419.1197.

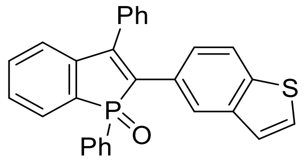

**2-(Benzo[*b*]thiophen-5-yl)-1,3-diphenylphosphindole 1-oxide (4ap):** pale yellow solid (0.2 mmol, 53.8 mg, 62% yield); m.p. 200.1-201.5 °C (from hexane); TLC $R_f$ 0.25 (hexane/EtOAc, 1:2). $^1H$ NMR ($CDCl_3$, 400 MHz) δ 7.91 (s, 1H), 7.81-7.71 (m, 3H), 7.54 (dd, $J$ = 8.5, 0.6 Hz, 1H), 7.49-7.34 (m, 10H), 7.32 (d, $J$ = 5.5 Hz, 1H), 7.24 (dd, $J$ = 7.5, 2.9 Hz, 1H), 7.16 (dd, $J$ = 5.5, 0.6 Hz, 1H), 7.08 (d, $J$ = 8.5 Hz, 1H). $^{13}C\{^1H\}$ NMR ($CDCl_3$, 100 MHz) δ 149.8 (d, $^2J_{CP}$ = 21.5 Hz, 1C), 143.8 (d, $^2J_{CP}$ = 27.0 Hz, 1C), 139.6 (1C), 139.2 (1C), 134.3 (d, $^3J_{CP}$ = 15.2 Hz, 1C), 134.2 (d, $^1J_{CP}$ = 95.7 Hz, 1C), 132.9 (d, $^4J_{CP}$ = 1.7 Hz, 1C), 132.2 (d, $^4J_{CP}$ = 2.8 Hz, 1C), 132.1 (d, $^1J_{CP}$ = 105.7 Hz, 1C), 130.9 (d, $^2J_{CP}$ = 10.8 Hz, 2C), 129.8 (d, $^1J_{CP}$ = 100.2 Hz, 1C), 129.2 (3C), 129.0 (3C), 128.93 (d, $^2J_{CP}$ = 10.6 Hz, 1C), 128.90 (d, $^3J_{CP}$ = 12.4 Hz, 2C), 128.7 (1C), 126.6 (1C), 125.2 (d, $^3J_{CP}$ = 6.4 Hz, 1C), 124.3 (1C), 124.2 (d, $^3J_{CP}$ = 5.3 Hz, 1C), 124.0 (d, $^2J_{CP}$ = 10.8 Hz, 1C), 122.2 (1C). $^{31}P\{^1H\}$ NMR ($CDCl_3$, 162 MHz) δ 39.2; HRMS (APCI) m/z ($[M+H]^+$) Calcd for $C_{28}H_{20}OPS$: 435.0967, Found: 435.0970.

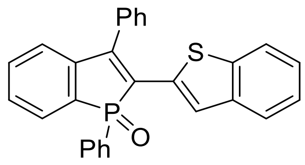

**2-(Benzo[*b*]thiophen-2-yl)-1,3-diphenylphosphindole 1-oxide (4aq):** yellow solid (0.2 mmol, 41.6 mg, 48% yield); m.p. 125.4-126.7 °C (from hexane); TLC $R_f$ 0.22 (hexane/EtOAc, 1:2). $^1$H NMR ($CDCl_3$, 400 MHz) δ 7.91-7.85 (m, 2H), 7.74 (s, 1H), 7.73-7.69 (m, 1H), 7.64-7.58 (m, 4H), 7.52-7.48 (m, 2H), 7.46-7.41 (m, 5H), 7.38-7.33 (m, 1H), 7.23-7.15 (m, 2H), 7.02 (dd, $J$ = 7.5, 2.9 Hz, 1H). $^{13}$C{$^1$H} NMR ($CDCl_3$, 100 MHz) δ 148.8 (d, $^2J_{CP}$ = 20.2 Hz, 1C), 144.2 (d, $^2J_{CP}$ = 26.1 Hz, 1C), 140.4 (1C), 138.8 (1C), 135.3 (d, $^3J_{CP}$ = 14.8 Hz, 1C), 133.7 (1C), 133.5 (1C), 133.2 (d, $^4J_{CP}$ = 1.9 Hz, 1C), 132.4 (d, $^4J_{CP}$ = 2.8 Hz, 1C), 131.5 (d, $^1J_{CP}$ = 107.3 Hz, 1C), 130.8 (d, $^2J_{CP}$ = 10.6 Hz, 2C), 130.1 (d, $^1J_{CP}$ = 100.5 Hz, 1C), 129.5 (1C), 129.4 (1C), 129.3 (2C), 129.2 (1C), 129.1 (d, $^3J_{CP}$ = 12.6 Hz, 2C), 128.96 (d, $^1J_{CP}$ = 95.7 Hz, 1C), 128.91 (d, $^2J_{CP}$ = 10.1 Hz, 1C), 126.6 (d, $^3J_{CP}$ = 4.6 Hz, 1C), 125.1 (1C), 124.3 (1C), 124.2 (d, $^2J_{CP}$ = 10.4 Hz, 1C), 124.1 (1C), 121.7 (1C). $^{31}$P{$^1$H} NMR ($CDCl_3$, 162 MHz) δ 38.7; HRMS (APCI) m/z ([M+H]$^+$) Calcd for $C_{28}H_{20}OPS$: 435.0967, Found: 435.0968.

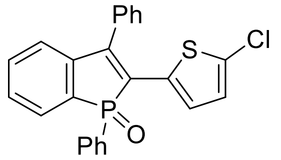

**2-(5-Chlorothiophen-2-yl)-1,3-diphenylphosphindole 1-oxide (4ar):** pale yellow solid (0.2 mmol, 35.9 mg, 43% yield); m.p. 175.8-177.1 °C (from hexane); TLC $R_f$ 0.25 (hexane/EtOAc, 1:2). $^1$H NMR ($CDCl_3$, 400 MHz) δ 7.85-7.80 (m, 2H), 7.68-7.64 (m, 1H), 7.60-7.51 (m, 4H), 7.48-7.42 (m, 3H), 7.40-7.30 (m, 3H), 7.20 (dd, $J$ = 4.0, 1.0 Hz, 1H), 6.96 (dd, $J$ = 7.5, 2.9 Hz, 1H), 6.65 (d, $J$ = 4.0 Hz, 1H). $^{13}$C{$^1$H} NMR ($CDCl_3$, 100 MHz) δ 146.5 (d, $^2J_{CP}$ = 20.3 Hz, 1C), 144.2 (d, $^2J_{CP}$ = 25.8 Hz, 1C), 134.2 (d, $^3J_{CP}$ = 15.7 Hz, 1C), 133.6 (1C), 133.3 (d, $^4J_{CP}$ = 1.9 Hz, 1C), 132.9 (d, $^1J_{CP}$ = 98.3 Hz, 1C), 132.5 (d, $^4J_{CP}$ = 2.8 Hz, 1C), 131.0 (d, $^1J_{CP}$ = 107.3 Hz, 1C), 130.8 (d, $^2J_{CP}$ = 10.6 Hz, 2C), 130.5 (1C), 130.0 (d, $^1J_{CP}$ = 100.2 Hz, 1C), 129.8 (1C), 129.6 (1C), 129.1 (d, $^3J_{CP}$ = 12.4 Hz, 2C), 129.0 (d, $^3J_{CP}$ = 10.6 Hz, 1C), 129.0 (1C), 128.9 (1C), 128.88 (2C), 128.85 (d, $^2J_{CP}$ = 10.3 Hz, 1C), 126.0 (1C), 123.9 (d, $^2J_{CP}$ = 10.9 Hz, 1C). $^{31}$P{$^1$H} NMR ($CDCl_3$, 162 MHz) δ 38.5; HRMS (APCI) m/z ([M+H]$^+$) Calcd for $C_{24}H_{17}ClOPS$: 419.0421, Found: 419.0437.

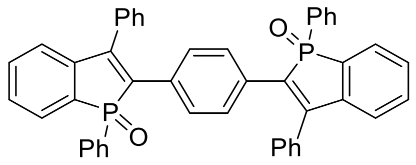

**2,2'-(1,4-Phenylene)bis(1,3-diphenylphosphindole 1-oxide) (4as, *syn* + *anti*, ca. 1:1 mixture):**

yellow solid (0.08 mmol scale, 28.7 mg, 53% yield); m.p. 267.6-268.6 °C (from hexane); TLC $R_f$ 0.20 (hexane/EtOAc, 1:3). $^1$H NMR ($CDCl_3$, 400 MHz) δ 7.71-7.62 (m, 6H), 7.51-7.45 (m, 2H), 7.43-7.31 (m, 14H), 7.23-7.19 (m, 4H), 7.15 (dd, $J$ = 7.5, 2.9 Hz, 2H), 6.93 (d, $J$ = 10.7 Hz, 4H). $^{13}$C{$^1$H} NMR ($CDCl_3$, 100 MHz) δ 150.2 (d, $^2J_{CP}$ = 21.5 Hz, 1C), 150.1 (d, $^2J_{CP}$ = 21.3 Hz, 1C), 143.7 (d, $^2J_{CP}$ = 27.0 Hz, 1C), 143.6 (d, $^2J_{CP}$ = 27.0 Hz, 1C), 134.1 (d, $^3J_{CP}$ = 14.8 Hz, 1C), 134.0 (d, $^3J_{CP}$ = 14.8 Hz, 1C), 133.6 (d, $^1J_{CP}$ = 95.6 Hz, 1C), 133.5 (d, $^1J_{CP}$ = 95.6 Hz, 1C), 132.9 (2H), 132.3 (d, $^3J_{CP}$ = 4.7 Hz, 1C), 132.2 (d, $^3J_{CP}$ = 4.7 Hz, 1C), 132.1 (d, $^4J_{CP}$ = 2.7 Hz, 2C), 132.0 (d, $^1J_{CP}$ = 106.4 Hz, 1C), 131.9 (d, $^1J_{CP}$ = 106.4 Hz, 1C), 130.9 (d, $^2J_{CP}$ = 10.8 Hz, 2C), 130.8 (d, $^2J_{CP}$ = 10.8 Hz, 2C), 129.97 (d, $^1J_{CP}$ = 99.3 Hz, 1C), 129.95 (d, $^1J_{CP}$ = 99.3 Hz, 1C), 129.2, 129.07, 129.05, 129.01, 128.9, 128.8, 128.79, 128.74, 124.1 (d, $^2J_{CP}$ = 10.9 Hz, 2C) (All observed signals are just shown because of complexity associated with C–P couplings and diastereomers.); $^{31}$P{$^1$H} NMR ($CDCl_3$, 162 MHz) δ 39.04, 38.97; HRMS (APCI) m/z ([M+H]$^+$) Calcd for $C_{46}H_{33}O_2P_2$: 679.1951, Found: 679.1956.

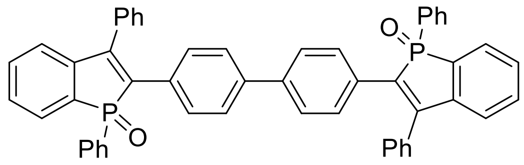


**2,2'-([1,1'-Biphenyl]-4,4'-diyl)bis(1,3-diphenylphosphindole 1-oxide) (4at, *syn* + *anti*, ca. 1:1 mixture):** yellow solid (0.08 mmol scale, 29.6 mg, 49% yield); m.p. 190.5-191.8 °C (from hexane); TLC $R_f$ 0.20 (hexane/EtOAc, 1:3). $^1$H NMR ($CDCl_3$, 400 MHz) δ 7.78-7.73 (m, 4H), 7.72-7.68 (m, 2H), 7.48-7.31 (m, 20H), 7.26-7.18 (m, 10H). $^{13}$C{$^1$H} NMR ($CDCl_3$, 100 MHz) δ 149.93 (d, $^2J_{CP}$ = 21.5 Hz, 1C), 149.91 (d, $^2J_{CP}$ = 21.5 Hz, 1C), 143.8 (d, $^2J_{CP}$ = 26.8 Hz, 2C), 139.4, 134.3 (d, $^3J_{CP}$ = 15.1 Hz, 2C), 133.71 (d, $^1J_{CP}$ = 98.7 Hz, 1C), 133.74 (d, $^1J_{CP}$ = 93.8 Hz, 1C), 132.9, 132.2, 132.0 (d, $^1J_{CP}$ = 106.2 Hz, 2C), 131.9 (d, $^2J_{CP}$ = 9.9 Hz, 2C), 130.9 (d, $^2J_{CP}$ = 10.8 Hz, 4C), 129.9 (d, $^1J_{CP}$ = 99.8 Hz, 2C), 129.4, 129.07, 129.01, 128.8, 128.7, 126.5, 124.1, 124.0, 123.99 (All observed signals are just shown because of complexity associated with C–P couplings and diastereomers.); $^{31}$P{$^1$H} NMR ($CDCl_3$, 162 MHz) δ 39.18, 39.15; HRMS (APCI) m/z ([M+H]$^+$) Calcd for $C_{52}H_{37}O_2P_2$: 755.2264, Found: 755.2282.

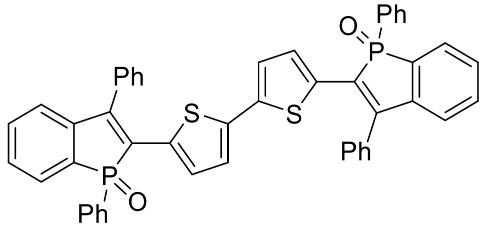


**2,2'-([2,2'-Bithiophene]-5,5'-diyl)bis(1,3-diphenylphosphindole 1-oxide) (4au, *syn* + *anti*, ca. 1:1 mixture):** dark orange solid (0.08 mmol scale, 42.2 mg, 69% yield); m.p. 179.8-181.1 °C (from hexane); TLC $R_f$ 0.20 (hexane/EtOAc, 1:3). $^1$H NMR ($CDCl_3$, 400 MHz) δ 7.82-7.77 (m, 4H), 7.64

(dd, $J$ = 9.9, 7.1 Hz, 2H), 7.56-7.54 (m, 6H), 7.52-7.47 (m, 2H), 7.44-7.27 (m, 12H), 7.21-7.19 (m, 2H), 6.95 (dt, $J$ = 7.7, 1.4 Hz, 2H), 6.65 (d, $J$ = 4.0 Hz, 1H), 6.63 (d, $J$ = 4.0 Hz, 1H). $^{13}C\{^{1}H\}$ NMR ($CDCl_3$, 100 MHz) δ 146.4, 146.2, 144.6, 144.3, 138.7, 135.0, 134.8, 133.9, 133.7, 133.2, 132.4, 132.3, 131.7, 130.9, 130.8, 130.7, 130.65, 130.62, 130.5, 130.4, 129.7, 129.6, 129.4, 129.1, 129.0, 128.95, 128.90, 128.79, 128.07, 128.04, 123.85, 123.80, 123.7 (All observed signals are just shown because of complexity associated with C–P couplings and diastereomers.); $^{31}P\{^{1}H\}$ NMR ($CDCl_3$, 162 MHz) δ 38.47, 38.41; HRMS (APCI) m/z ($[M+H]^+$) Calcd for $C_{48}H_{33}O_2P_2S_2$: 767.1392, Found: 767.1388.

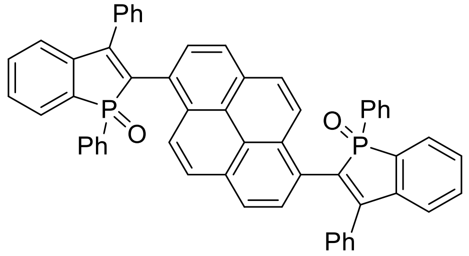


**2,2'-(Pyrene-1,6-diyl)bis(1,3-diphenylphosphindole 1-oxide) (4av, *syn* + *anti*, the ratio was not determined):** yellow solid (0.08 mmol scale, 23.1 mg, 36% yield); m.p. over 270 °C (from hexane); TLC $R_f$ 0.22 (hexane/EtOAc, 1:3). $^{1}H$ NMR ($CDCl_3$, 400 MHz) δ 8.51 (bs, 2H), 7.86-7.30 (m, 23H), 7.20 (bs, 4H), 7.10 (bs, 7H). $^{13}C\{^{1}H\}$ NMR ($CDCl_3$, 100 MHz) δ 152.5, 143.8, 143.6, 133.7, 133.5, 133.1, 132.7, 132.3, 131.7, 131.3, 131.2, 129.8, 129.7, 129.5, 129.4, 129.3, 128.8, 128.7, 128.6, 128.5, 128.4, 128.3, 127.7, 127.6, 127.5, 124.7, 124.4, 124.3 (All observed signals are just shown because of complexity associated with C–P couplings and diastereomers.); $^{31}P\{^{1}H\}$ NMR ($CDCl_3$, 162 MHz) δ 39.1; HRMS (APCI) m/z ($[M+H]^+$) Calcd for $C_{56}H_{37}O_2P_2$: 803.2264, Found: 803.2275.

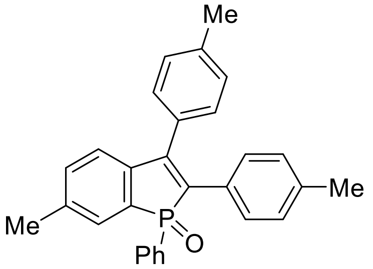


**6-Methyl-1-phenyl-2,3-di-*p*-tolylphosphindole 1-oxide (4ba):** colorless solid (0.2 mmol scale, 65.5 mg, 78% yield); m.p. 84.9-86.3 °C (from hexane); TLC $R_f$ 0.24 (hexane/EtOAc, 1:2). $^{1}H$ NMR ($CDCl_3$, 400 MHz) δ 7.81-7.75 (m, 2H), 7.50-7.43 (m, 2H), 7.40-7.35 (m, 2H), 7.24-7.20 (m, 5H), 7.14 (d, $J$ = 7.5 Hz, 2H), 7.08 (dd, $J$ = 7.8, 3.1 Hz, 1H), 6.88 (d, $J$ = 8.3 Hz, 2H), 2.40 (s, 3H), 2.33 (s, 3H), 2.18 (s, 3H). $^{13}C\{^{1}H\}$ NMR ($CDCl_3$, 100 MHz) δ 149.6 (d, $^{2}J_{CP}$ = 22.0 Hz, 1C), 141.4 (d, $^{2}J_{CP}$ = 27.2 Hz, 1C), 139.2 (d, $^{3}J_{CP}$ = 10.4 Hz, 1C), 138.4 (1C), 137.5 (1C), 133.2 (d, $^{4}J_{CP}$ = 1.5 Hz, 1C), 132.6 (d, $^{1}J_{CP}$ = 96.9 Hz, 1C), 132.2 (d, $^{1}J_{CP}$ = 105.4 Hz, 1C), 132.0 (d, $^{4}J_{CP}$ = 2.8 Hz, 1C), 131.6 (d, $^{3}J_{CP}$ = 15.3 Hz, 1C), 131.0 (d, $^{2}J_{CP}$ = 10.5 Hz, 2C), 130.5 (d, $^{1}J_{CP}$ = 98.0 Hz, 1C), 130.1 (1C), 129.7

(d, $^2J_{CP}$ = 9.7 Hz, 1C), 129.6 (2C), 129.0 (2C), 128.9 (2C), 128.89 (d, $^3J_{CP}$ = 6.0 Hz, 2C), 128.80 (d, $^3J_{CP}$ = 12.1 Hz, 2C), 123.8 (d, $^2J_{CP}$ = 11.6 Hz, 1C), 21.5 (1C), 21.26 (1C), 21.24 (1C). $^{31}$P{$^1$H} NMR ($CDCl_3$, 162 MHz) δ 39.2; HRMS (APCI) m/z ([M+H]$^+$) Calcd for $C_{29}H_{26}OP$: 421.1716, Found: 421.1720.

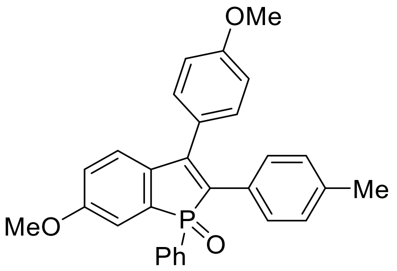


**6-Methoxy-3-(4-methoxyphenyl)-1-phenyl-2-(*p*-tolyl)phosphindole 1-oxide (4da):** colorless solid (0.2 mmol scale, 69.6 mg, 77% yield); m.p. 89.9-91.2 °C (from hexane); TLC $R_f$ 0.22 (hexane/EtOAc, 1:2). $^1$H NMR ($CDCl_3$, 400 MHz) δ 7.80-7.75 (m, 2H), 7.48-7.44 (m, 1H), 7.41-7.36 (m, 2H), 7.27-7.22 (m, 3H), 7.16 (dd, $J$ = 8.5, 3.6 Hz, 1H), 7.11 (d, $J$ = 7.4 Hz, 2H), 6.96-6.88 (m, 5H), 3.85 (s, 3H), 3.79 (s, 3H), 2.19 (s, 3H). $^{13}$C{$^1$H} NMR ($CDCl_3$, 100 MHz) δ 160.5 (d, $^3J_{CP}$ = 13.4 Hz, 1C), 159.7 (1C), 149.3 (d, $^2J_{CP}$ = 21.3 Hz, 1C), 137.2 (1C), 136.4 (d, $^2J_{CP}$ = 27.0 Hz, 1C), 134.2 (d, $^1J_{CP}$ = 104.5 Hz, 1C), 132.0 (d, $^4J_{CP}$ = 2.7 Hz, 1C), 131.2 (d, $^1J_{CP}$ = 98.1 Hz, 1C), 131.0 (d, $^2J_{CP}$ = 10.7 Hz, 2C), 130.4 (2C), 130.3 (d, $^1J_{CP}$ = 99.1 Hz, 1C), 130.1 (d, $^2J_{CP}$ = 10.3 Hz, 1C), 129.0 (2C), 128.83 (d, $^3J_{CP}$ = 12.2 Hz, 2C), 128.82 (d, $^3J_{CP}$ = 5.9 Hz, 2C), 126.7 (d, $^3J_{CP}$ = 15.6 Hz, 1C), 125.1 (d, $^3J_{CP}$ = 12.8 Hz, 1C), 117.8 (1C), 114.5 (d, $^2J_{CP}$ = 10.6 Hz, 1C), 114.3 (2C), 55.7 (1C), 55.3 (1C), 21.2 (1C). $^{31}$P{$^1$H} NMR ($CDCl_3$, 162 MHz) δ 38.7; HRMS (APCI) m/z ([M+H]$^+$) Calcd for $C_{29}H_{26}O_3P$: 453.1615, Found: 453.1613.

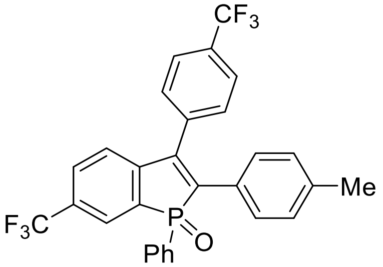


**1-Phenyl-2-(*p*-tolyl)-6-(trifluoromethyl)-3-(4-(trifluoromethyl)phenyl)phosphindole 1-oxide (4ea):** colorless solid (0.2 mmol scale, 53.8 mg, 51% yield); m.p. 151.3-152.6 °C (from hexane); TLC $R_f$ 0.25 (hexane/EtOAc, 1:2). $^1$H NMR ($CDCl_3$, 400 MHz) δ 7.95 (d, $J$ = 9.7 Hz, 1H), 7.79-7.70 (m, 5H), 7.56-7.42 (m, 5H), 7.23 (dd, $J$ = 8.0, 2.7 Hz, 1H), 7.13 (d, $J$ = 8.2 Hz, 2H), 6.94 (d, $J$ = 8.2 Hz, 2H), 2.22 (s, 3H). $^{13}$C{$^1$H} NMR ($CDCl_3$, 100 MHz) δ 146.6, 146.4, 146.3, 146.1, 139.1, 138.7, 137.8, 137.7, 133.6, 132.84, 132.81, 132.5, 131.2, 131.1, 131.0, 130.95, 130.93, 130.8, 130.34, 130.32, 130.30, 129.5, 129.4, 129.3, 129.1, 128.9, 128.8, 128.6, 128.5, 127.8, 126.33, 126.30, 126.2, 126.1,

126.06, 126.03, 125.2, 124.9, 123.7, 123.6, 122.5, 21.3. (All observed signals are just shown because of complexity associated with C–F and C–P couplings.); $^{19}F\{^{1}H\}$ NMR ($CDCl_3$, 376 MHz) δ -62.6, -62.7. $^{31}P\{^{1}H\}$ NMR ($CDCl_3$, 162 MHz) δ 37.9; HRMS (APCI) m/z ($[M+H]^+$) Calcd for $C_{29}H_{20}F_6OP$: 529.1151, Found: 529.1154.

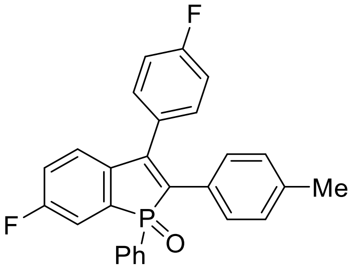


**6-Fluoro-3-(4-fluorophenyl)-1-phenyl-2-(*p*-tolyl)phosphindole 1-oxide (4fa):** pale yellow solid (0.2 mmol scale, 60.7 mg, 71% yield); m.p. 218.1-219.4 °C (from hexane); TLC $R_f$ 0.25 (hexane/EtOAc, 1:2). $^{1}H$ NMR ($CDCl_3$, 400 MHz) δ 7.78-7.73 (m, 2H), 7.53-7.48 (m, 1H), 7.43-7.37 (m, 3H), 7.33-7.29 (m, 2H), 7.17-7.09 (m, 6H), 6.92 (d, $J$ = 8.5 Hz, 2H), 2.21 (s, 3H). $^{13}C\{^{1}H\}$ NMR ($CDCl_3$, 100 MHz) δ 164.6, 164.5, 164.1, 162.1, 162.0, 161.6, 147.66, 147.65, 147.45, 147.44, 139.64, 139.62, 139.38, 139.35, 138.1, 135.38, 135.31, 134.9, 134.3, 134.27, 133.9, 132.5, 132.48, 131.3, 131.2, 131.0, 130.9, 130.89, 130.84, 130.6, 130.2, 130.17, 130.05, 130.01, 129.7, 129.4, 129.3, 129.2, 129.1, 129.0, 128.8, 128.7, 128.6, 128.4, 125.4, 125.3, 125.2, 125.1, 119.5, 119.3, 117.0, 116.9, 116.7, 116.6, 116.5, 116.2, 21.2 (All observed signals are just shown because of complexity associated with C–F and C–P couplings.); $^{19}F\{^{1}H\}$ NMR ($CDCl_3$, 376 MHz) δ -111.1 (d, $J_{FP}$ = 5.3 Hz, 1F), -111.7. $^{31}P\{^{1}H\}$ NMR ($CDCl_3$, 162 MHz) δ 37.7 (d, $J_{PF}$ = 5.3 Hz); HRMS (APCI) m/z ($[M+H]^+$) Calcd for $C_{27}H_{20}F_2OP$: 429.1215, Found: 429.1229.

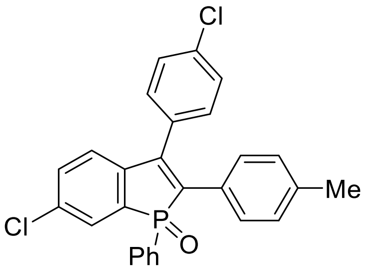


**6-Chloro-3-(4-chlorophenyl)-1-phenyl-2-(*p*-tolyl)phosphindole 1-oxide (4ga):** pale yellow solid (0.2 mmol scale, 60.7 mg, 66% yield); m.p. 107.2-108.6 °C (from hexane); TLC $R_f$ 0.25 (hexane/EtOAc, 1:2). $^{1}H$ NMR ($CDCl_3$, 400 MHz) δ 7.77-7.72 (m, 2H), 7.64 (dd, $J$ = 9.8, 2.0 Hz, 1H), 7.53-7.48 (m, 1H), 7.44-7.39 (m, 5H), 7.26 (d, $J$ = 8.1 Hz, 2H), 7.11 (d, $J$ = 7.8 Hz, 2H), 7.09 (dd, $J$ = 8.1, 3.3 Hz, 1H), 6.92 (d, $J$ = 8.1 Hz, 2H), 2.21 (s, 3H). $^{13}C\{^{1}H\}$ NMR ($CDCl_3$, 100 MHz) δ 147.1 (d, $^{2}J_{CP}$ = 21.1 Hz, 1C), 141.7 (d, $^{2}J_{CP}$ = 26.5 Hz, 1C), 138.4 (1C), 135.5 (d, $^{3}J_{CP}$ = 13.6 Hz, 1C), 135.0 (d, $^{1}J_{CP}$ = 95.6 Hz, 1C), 134.9 (1C), 134.2 (d, $^{1}J_{CP}$ = 103.3 Hz, 1C), 132.8 (d, $^{4}J_{CP}$ = 1.2

Hz, 1C), 132.6 (d, $^4J_{CP}$ = 2.5 Hz, 1C), 132.4 (1C), 130.9 (d, $^2J_{CP}$ = 10.7 Hz, 2C), 130.4 (2C), 129.5 (2C), 129.4 (d, $^2J_{CP}$ = 10.0 Hz, 1C), 129.3 (2C), 129.2 (d, $^3J_{CP}$ = 15.3 Hz, 1C), 129.1 (d, $^3J_{CP}$ = 12.2 Hz, 2C), 128.9 (d, $^1J_{CP}$ = 100.8 Hz, 1C), 128.8 (d, $^3J_{CP}$ = 5.9 Hz, 2C), 124.7 (d, $^2J_{CP}$ = 11.7 Hz, 1C), 21.3 (1C). $^{31}P\{^1H\}$ NMR ($CDCl_3$, 162 MHz) δ 38.0; HRMS (APCI) m/z ($[M+H]^+$) Calcd for $C_{27}H_{20}Cl_2OP$: 461.0629, Found: 461.0657.

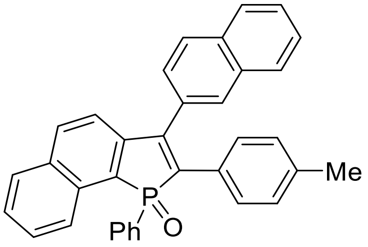


**3-(Naphthalen-2-yl)-1-phenyl-2-(*p*-tolyl)benzo[*g*]phosphindole 1-oxide (4ha):** yellow solid (0.2 mmol scale, 64.9 mg, 66% yield); m.p. 135.3-136.6 ºC (from hexane); TLC $R_f$ 0.20 (hexane/EtOAc, 1:2). $^1$H NMR ($CDCl_3$, 400 MHz) δ 8.13 (dd, $J$ = 8.2, 0.5 Hz, 1H), 7.93-7.85 (m, 7H), 7.81 (d, $J$ = 7.9 Hz, 1H), 7.58-7.51 (m, 2H), 7.50-7.33 (m, 7H), 7.23 (d, $J$ = 7.9 Hz, 2H), 6.86 (d, $J$ = 8.4 Hz, 2H), 2.16 (s, 3H). $^{13}C\{^1H\}$ NMR ($CDCl_3$, 100 MHz) δ 148.5 (d, $^2J_{CP}$ = 23.2 Hz, 1C), 143.3 (d, $^2J_{CP}$ = 26.2 Hz, 1C), 137.9 (1C), 135.0 (d, $^1J_{CP}$ = 95.6 Hz, 1C), 133.6 (d, $^4J_{CP}$ = 2.1 Hz, 1C), 133.5 (d, $^3J_{CP}$ = 7.9 Hz, 1C), 133.4 (1C), 133.2 (1C), 132.3 (d, $^3J_{CP}$ = 15.7 Hz, 1C), 132.1 (d, $^4J_{CP}$ = 2.7 Hz, 1C), 131.8 (d, $^2J_{CP}$ = 9.2 Hz, 1C), 130.9 (d, $^2J_{CP}$ = 10.4 Hz, 2C), 129.6 (d, $^2J_{CP}$ = 10.6 Hz, 1C), 129.4 (d, $^1J_{CP}$ = 95.1 Hz, 1C), 129.1 (1C), 129.08 (1C), 129.07 (d, $^3J_{CP}$ = 12.5 Hz, 2C), 129.02 (d, $^3J_{CP}$ = 6.1 Hz, 2C), 128.9 (1C), 128.7 (d, $^4J_{CP}$ = 1.5 Hz, 1C), 128.5 (1C), 128.3 (1C), 128.2 (1C), 128.0 (1C), 127.4 (d, $^1J_{CP}$ = 102.2 Hz, 1C), 126.9 (1C), 126.76 (1C), 126.75 (1C), 126.6 (1C), 125.7 (d, $^3J_{CP}$ = 4.9 Hz, 1C), 121.3 (d, $^3J_{CP}$ = 12.5 Hz, 1C), 21.3 (1C). $^{31}P\{^1H\}$ NMR ($CDCl_3$, 162 MHz) δ 39.9; HRMS (APCI) m/z ($[M+H]^+$) Calcd for $C_{35}H_{26}OP$: 493.1716, Found: 493.1723.

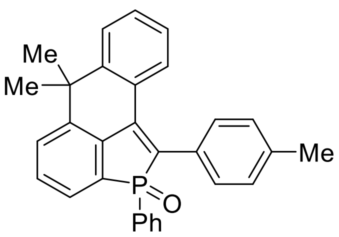


**6,6-Dimethyl-2-phenyl-1-(*p*-tolyl)-6*H*-naphtho[1,2,3-*cd*]phosphindole 2-oxide (4ia):** colorless solid (0.2 mmol scale, 54.4 mg, 63% yield); m.p. 131.3-132.6 ºC (from hexane); TLC $R_f$ 0.22 (hexane/EtOAc, 1:2). $^1$H NMR ($CDCl_3$, 400 MHz) δ 7.71 (d, $J$ = 8.0 Hz, 1H), 7.67-7.57 (m, 5H), 7.48-7.42 (m, 2H), 7.39-7.33 (m, 3H), 7.12 (bs, 4H), 6.96 (td, $J$ = 7.5, 1.2 Hz, 1H), 2.33 (d, $J$ = 1.1 Hz, 3H), 1.77 (s, 3H), 1.72 (s, 3H). $^{13}C\{^1H\}$ NMR ($CDCl_3$, 100 MHz) δ 146.9 (1C), 141.5 (d, $^3J_{CP}$ = 11.6 Hz, 1C), 139.0 (d, $^2J_{CP}$ = 23.2 Hz, 1C), 137.7 (d, $^4J_{CP}$ = 2.1 Hz, 1C), 137.4 (d, $^2J_{CP}$ = 29.3 Hz, 1C), 132.0 (d, $^4J_{CP}$ = 2.7 Hz, 1C), 131.3 (d, $^2J_{CP}$ = 10.8 Hz, 2C), 131.28 (1C), 130.8 (d, $^1J_{CP}$ = 98.9

Hz, 1C), 130.5 (d, $^4J_{CP}$ = 1.6 Hz, 1C), 130.4 (1C), 130.3 (d, $^1J_{CP}$ = 103.9 Hz, 1C), 130.0 (2C), 128.9 (d, $^1J_{CP}$ = 100.7 Hz, 1C), 129.5 (d, $^3J_{CP}$ = 11.1 Hz, 1C), 128.6 (d, $^3J_{CP}$ = 12.2 Hz, 2C), 128.5 (1C), 128.4 (1C), 128.3 (1C), 127.5 (1C), 127.4 (d, $^2J_{CP}$ = 9.0 Hz, 1C), 127.2 (d, $^2J_{CP}$ = 17.0 Hz, 1C), 125.9 (1C), 38.6 (d, $^4J_{CP}$ = 1.1 Hz, 1C), 33.4 (1C), 33.0 (1C), 21.4 (1C). $^{31}P\{^1H\}$ NMR ($CDCl_3$, 162 MHz) δ 39.8; HRMS (APCI) m/z ($[M+H]^+$) Calcd for $C_{30}H_{26}OP$: 433.1716, Found: 433.1738.

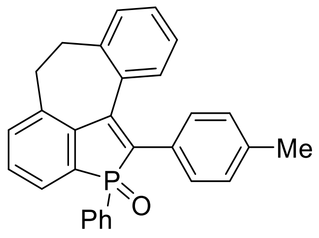


**6-Phenyl-5-(*p*-tolyl)-10,11-dihydrobenzo[6,7]cyclohepta[1,2,3-*cd*]phosphindole 6-oxide (4ja):** colorless solid (0.2 mmol scale, 46.0 mg, 55% yield); m.p. 126.4-127.7 °C (from hexane); TLC $R_f$ 0.24 (hexane/EtOAc, 1:2). $^1H$ NMR ($CDCl_3$, 400 MHz) δ 7.72 (bs, 2H), 7.53 (ddd, $J$ = 10.2, 6.4, 1.7 Hz, 1H), 7.43-7.36 (m, 3H), 7.25-7.18 (m, 7H), 6.93-6.87 (m, 3H), 3.20-3.07 (m, 4H), 2.21 (s, 3H). $^{13}C\{^1H\}$ NMR ($CDCl_3$, 100 MHz) δ 150.1 (d, $^2J_{CP}$ = 19.5 Hz, 1C), 143.3 (1C), 140.2 (d, $^2J_{CP}$ = 28.2 Hz, 1C), 139.5 (d, $^3J_{CP}$ = 10.6 Hz, 1C), 137.6 (1C), 135.6 (d, $^4J_{CP}$ = 1.5 Hz, 1C), 134.1 (d, $^3J_{CP}$ = 15.0 Hz, 1C), 133.2 (1C), 131.9 (d, $^4J_{CP}$ = 2.5 Hz, 1C), 131.4 (d, $^1J_{CP}$ = 93.9 Hz, 1C), 131.3 (d, $^2J_{CP}$ = 8.7 Hz, 1C), 131.0 (d, $^2J_{CP}$ = 10.3 Hz, 2C), 130.3 (d, $^1J_{CP}$ = 102.5 Hz, 1C), 129.8 (1C), 129.3 (d, $^3J_{CP}$ = 5.4 Hz, 2C), 129.2 (2C), 128.8 (d, $^3J_{CP}$ = 11.9 Hz, 2C), 128.3 (1C), 128.1 (d, $^3J_{CP}$ = 11.5 Hz, 1C), 127.5 (d, $^1J_{CP}$ = 98.5 Hz, 1C), 126.9 (d, $^2J_{CP}$ = 9.6 Hz, 1C), 125.8 (1C), 35.4 (1C), 34.7 (1C), 21.3 (1C). $^{31}P\{^1H\}$ NMR ($CDCl_3$, 162 MHz) δ 35.5; HRMS (APCI) m/z ($[M+H]^+$) Calcd for $C_{29}H_{24}OP$: 419.1560, Found: 419.1562.

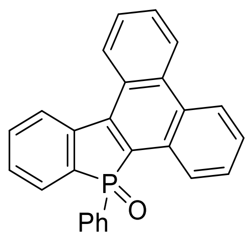


**9-Phenyltribenzo[*b*,*e*,*g*]phosphindole 9-oxide (5aa):** colorless solid (0.2 mmol scale, 56.3 mg, 75% yield); TLC $R_f$ 0.28 (hexane/EtOAc, 1:2). $^1H$ NMR ($CDCl_3$, 400 MHz) δ 8.97-8.95 (m, 1H), 8.80-8.78 (m, 1H), 8.64 (d, $J$ = 8.5 Hz, 1H), 8.51 (dd, $J$ = 8.0, 3.5 Hz, 1H), 8.31 (d, $J$ = 8.0 Hz, 1H), 7.85-7.72 (m, 5H), 7.68-7.60 (m, 2H), 7.56-7.52 (m, 1H), 7.46-7.40 (m, 2H), 7.36-7.32 (m, 2H). $^{13}C\{^1H\}$ NMR ($CDCl_3$, 100 MHz) δ 142.5 (d, $^2J_{CP}$ = 23.4 Hz, 1C), 139.5 (d, $^2J_{CP}$ = 20.0 Hz, 1C), 134.6 (d, $^1J_{CP}$ = 106.5 Hz, 1C), 133.9 (d, $^4J_{CP}$ = 1.9 Hz, 1C), 133.1 (d, $^4J_{CP}$ = 2.0 Hz, 1C), 132.2 (d, $^4J_{CP}$ = 2.8 Hz, 1C), 131.1 (d, $^2J_{CP}$ = 10.8 Hz, 2C), 130.8 (d, $^1J_{CP}$ = 102.1 Hz, 1C), 130.6 (d, $^3J_{CP}$ = 14.4 Hz, 1C), 130.09 (d, $^3J_{CP}$ = 9.9 Hz, 1C), 130.05 (d, $^1J_{CP}$ = 108.8 Hz, 1C), 129.3 (d, $^2J_{CP}$ = 8.9 Hz, 1C), 128.9 (d,

$^{3}J_{CP}$ = 11.4 Hz, 1C), 128.8 (d, $^{3}J_{CP}$ = 12.5 Hz, 2C), 128.6 (1C), 128.0 (1C), 127.9 (d, $^{3}J_{CP}$ = 13.9 Hz, 1C), 127.8 (1C), 127.4 (1C), 127.0 (d, $^{3}J_{CP}$ = 5.4 Hz, 1C), 125.9 (1C), 125.6 (d, $^{2}J_{CP}$ = 10.9 Hz, 1C), 124.1 (1C), 123.1 (1C). $^{31}P\{^{1}H\}$ NMR ($CDCl_3$, 162 MHz) δ 34.0; HRMS (APCI) m/z ($[M+H]^+$) Calcd for $C_{26}H_{18}OP$: 377.1089, Found: 377.1091.

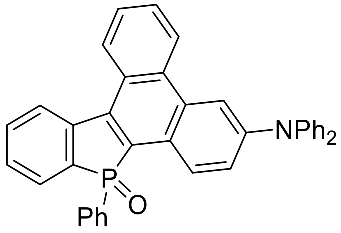


**6-(Diphenylamino)-9-phenyltribenzo[*b*,*e*,*g*]phosphindole 9-oxide (5ab):** yellow solid (0.2 mmol scale, 64.1 mg, 59% yield); m.p. 225.9-226.9 °C (from hexane); TLC $R_f$ 0.20 (hexane/EtOAc, 1:2). $^{1}H$ NMR ($CDCl_3$, 400 MHz) δ 8.93 (d, $J$ = 7.8 Hz, 1H), 8.48 (dd, $J$ = 8.0, 3.4 Hz, 1H), 8.41 (d, $J$ = 7.8 Hz, 1H), 8.25 (s, 1H), 8.12 (d, $J$ = 8.8 Hz, 1H), 7.82-7.70 (m, 4H), 7.68-7.63 (m, 2H), 7.48-7.34 (m, 4H), 7.30-7.25 (m, 5H), 7.17-7.15 (m, 4H), 7.08 (t, $J$ = 7.3 Hz, 2H). $^{13}C\{^{1}H\}$ NMR ($CDCl_3$, 100 MHz) δ 147.6 (1C), 147.3 (2C), 142.9 (d, $^{2}J_{CP}$ = 23.5 Hz, 1C), 137.1 (d, $^{2}J_{CP}$ = 20.0 Hz, 1C), 134.4 (d, $^{1}J_{CP}$ = 106.5 Hz, 1C), 133.1 (d, $^{4}J_{CP}$ = 2.5 Hz, 1C), 132.1 (d, $^{4}J_{CP}$ = 2.9 Hz, 1C), 132.0 (d, $^{3}J_{CP}$ = 9.5 Hz, 1C), 131.1 (d, $^{2}J_{CP}$ = 10.8 Hz, 2C), 130.9 (d, $^{1}J_{CP}$ = 102.0 Hz, 1C), 130.1 (d, $^{3}J_{CP}$ = 9.7 Hz, 1C), 130.0 (d, $^{1}J_{CP}$ = 102.2 Hz, 1C), 129.5 (4C), 128.8 (d, $^{3}J_{CP}$ = 11.4 Hz, 1C), 128.8 (d, $^{3}J_{CP}$ = 12.5 Hz, 2C), 128.5 (1C), 128.4 (d, $^{4}J_{CP}$ = 2.7 Hz, 1C), 128.1 (1C), 127.8 (d, $^{3}J_{CP}$ = 5.6 Hz, 1C), 127.4 (1C), 125.6 (1C), 125.2 (d, $^{2}J_{CP}$ = 10.9 Hz, 1C), 125.0 (4C), 124.7 (d, $^{2}J_{CP}$ = 8.9 Hz, 1C), 124.6 (1C), 124.2 (1C), 123.7 (2C), 115.2 (1C). $^{31}P\{^{1}H\}$ NMR ($CDCl_3$, 162 MHz) δ 34.0; HRMS (APCI) m/z ($[M+H]^+$) Calcd for $C_{38}H_{27}NOP$: 544.1825, Found: 544.1853.

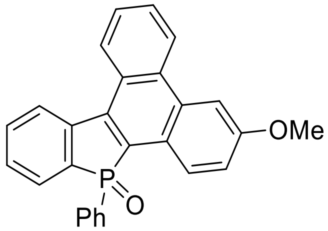


**6-Methoxy-9-phenyltribenzo[*b*,*e*,*g*]phosphindole 9-oxide (5ac):** yellow solid (0.2 mmol scale, 53.6 mg, 66% yield); m.p. 215.3-216.3 °C (from hexane); TLC $R_f$ 0.24 (hexane/EtOAc, 1:2). $^{1}H$ NMR ($CDCl_3$, 400 MHz) δ 8.94 (dd, $J$ = 5.2, 4.1 Hz, 1H), 8.70 (dd, $J$ = 5.2, 4.1 Hz, 1H), 8.47 (dd, $J$ = 7.8, 3.4 Hz, 1H), 8.23 (d, $J$ = 8.9 Hz, 1H), 8.00 (s, 1H), 7.83-7.70 (m, 5H), 7.64 (t, $J$ = 7.8 Hz, 1H), 7.46-7.32 (m, 4H), 7.19 (dd, $J$ = 8.9, 2.4 Hz, 1H), 3.96 (s, 3H). $^{13}C\{^{1}H\}$ NMR ($CDCl_3$, 100 MHz) δ 159.3 (1C), 142.8 (d, $^{2}J_{CP}$ = 22.9 Hz, 1C), 136.8 (d, $^{2}J_{CP}$ = 20.0 Hz, 1C), 134.3 (d, $^{1}J_{CP}$ = 106.5 Hz, 1C), 133.2 (d, $^{4}J_{CP}$ = 1.9 Hz, 1C), 133.1 (d, $^{4}J_{CP}$ = 1.9 Hz, 1C), 132.4 (d, $^{2}J_{CP}$ = 8.6 Hz, 1C), 132.1 (d, $^{4}J_{CP}$ = 2.8 Hz, 1C), 131.05 (d, $^{2}J_{CP}$ = 10.9 Hz, 2C), 131.03 (d, $^{1}J_{CP}$ = 102.1 Hz, 1C), 130.02 (d, $^{3}J_{CP}$ = 9.7

Hz, 1C), 130.0 (d, $^1J_{CP}$ = 102.8 Hz, 1C), 128.8 (d, $^3J_{CP}$ = 12.5 Hz, 2C), 128.5 (d, $^3J_{CP}$ = 5.6 Hz, 1C), 128.4 (d, $^3J_{CP}$ = 10.8 Hz, 1C), 128.3 (d, $^3J_{CP}$ = 12.1 Hz, 1C), 128.1 (1C), 127.5 (1C), 125.7 (1C), 125.2 (d, $^2J_{CP}$ = 10.9 Hz, 1C), 124.1 (1C), 123.9 (d, $^3J_{CP}$ = 9.2 Hz, 1C), 117.9 (1C), 104.7 (1C), 55.5 (1C). $^{31}P\{^1H\}$ NMR ($CDCl_3$, 162 MHz) δ 34.0; HRMS (APCI) m/z ($[M+H]^+$) Calcd for $C_{27}H_{20}O_2P$: 407.1196, Found: 407.1182.

[$^{1}H$, $^{13}C\{^{1}H\}$, and $^{31}P\{^{1}H\}$ NMR Spectra of **2a**]

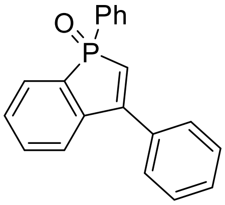


**2a**

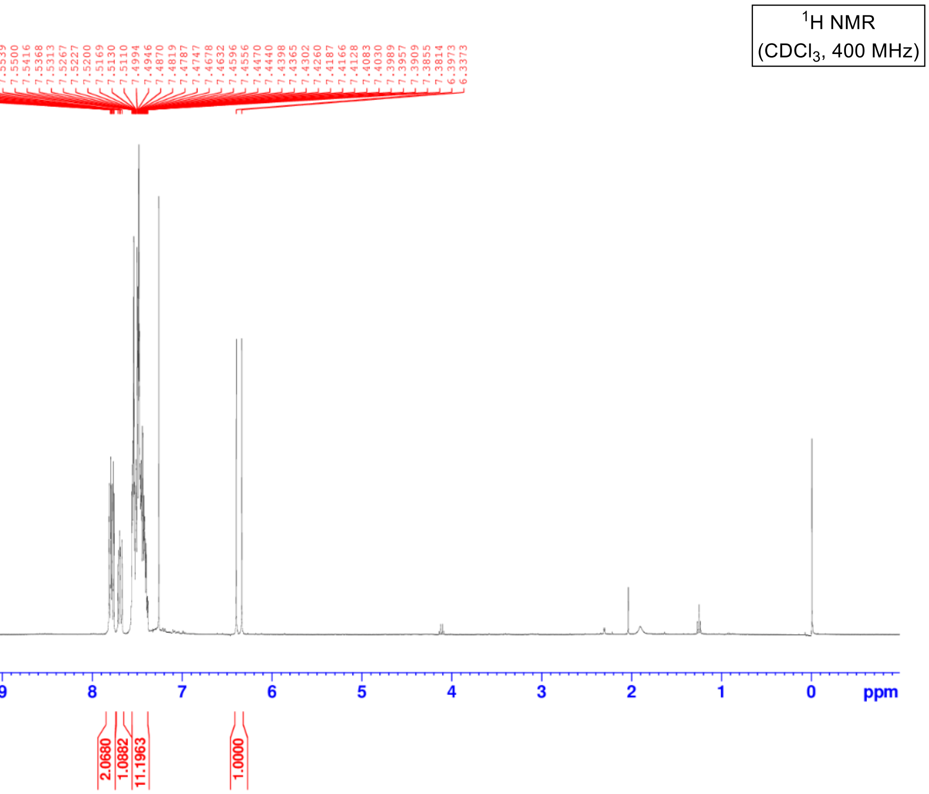


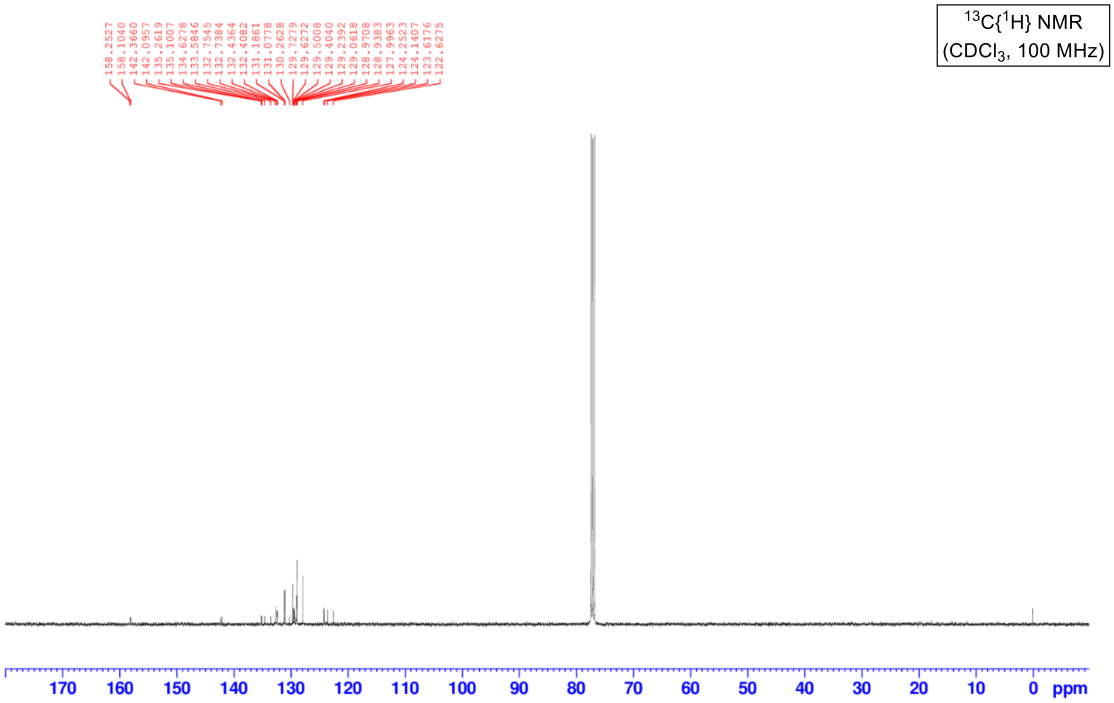

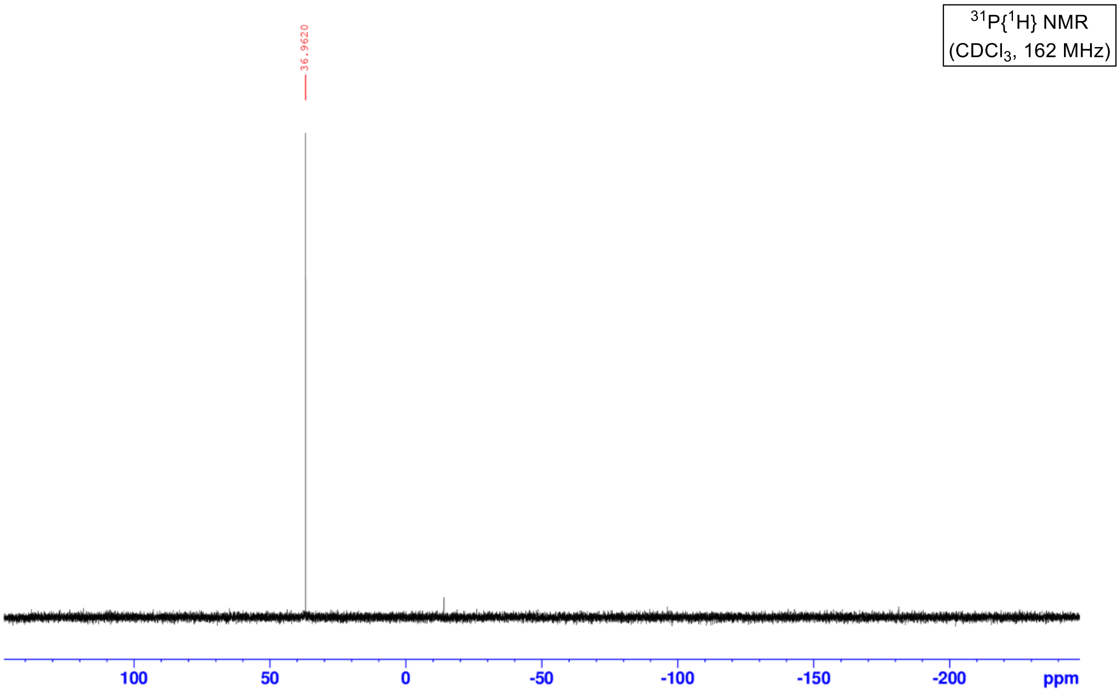
36.9620
$^{31}P\{^1H\}$ NMR
($CDCl_3$, 162 MHz)
100
50
0
-50
-100
-150
-200
ppm

[$^{1}H$, $^{13}C\{^{1}H\}$, and $^{31}P\{^{1}H\}$ NMR Spectra of **2b**]

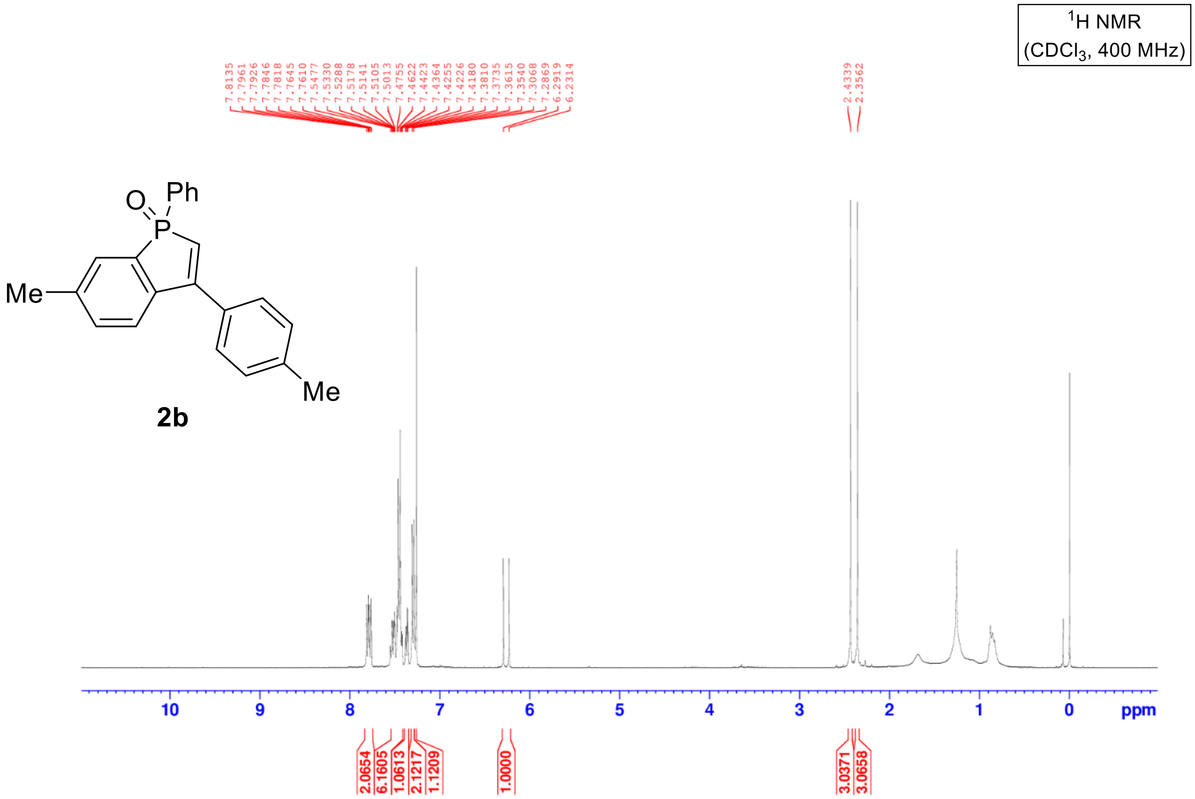


**2b**

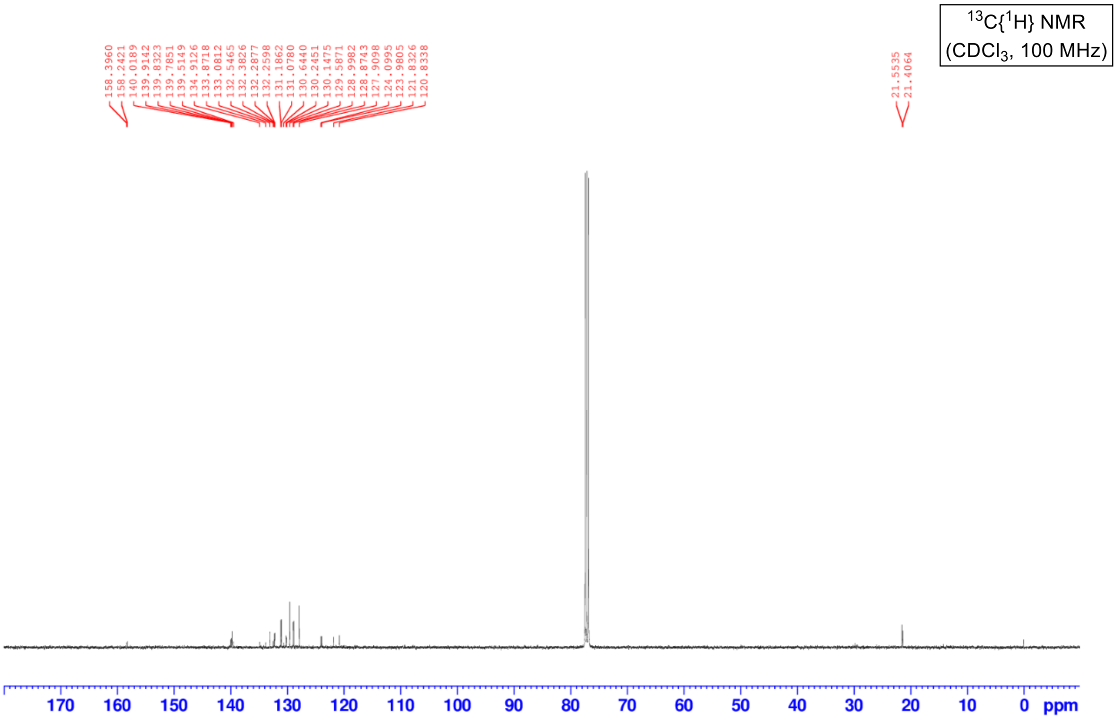

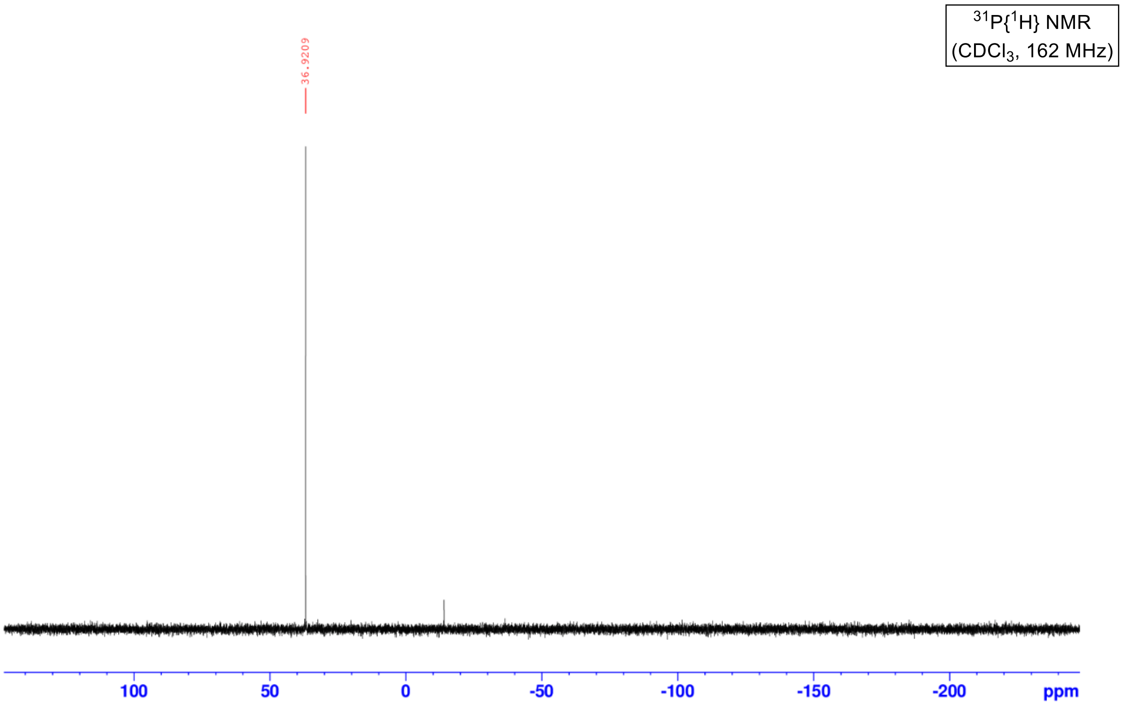
36.9209
$^{31}P\{^1H\}$ NMR
($CDCl_3$, 162 MHz)
100
50
0
-50
-100
-150
-200
ppm

[$^1$H, $^{13}$C{$^1$H}, and $^{31}$P{$^1$H} NMR Spectra of **2c**]

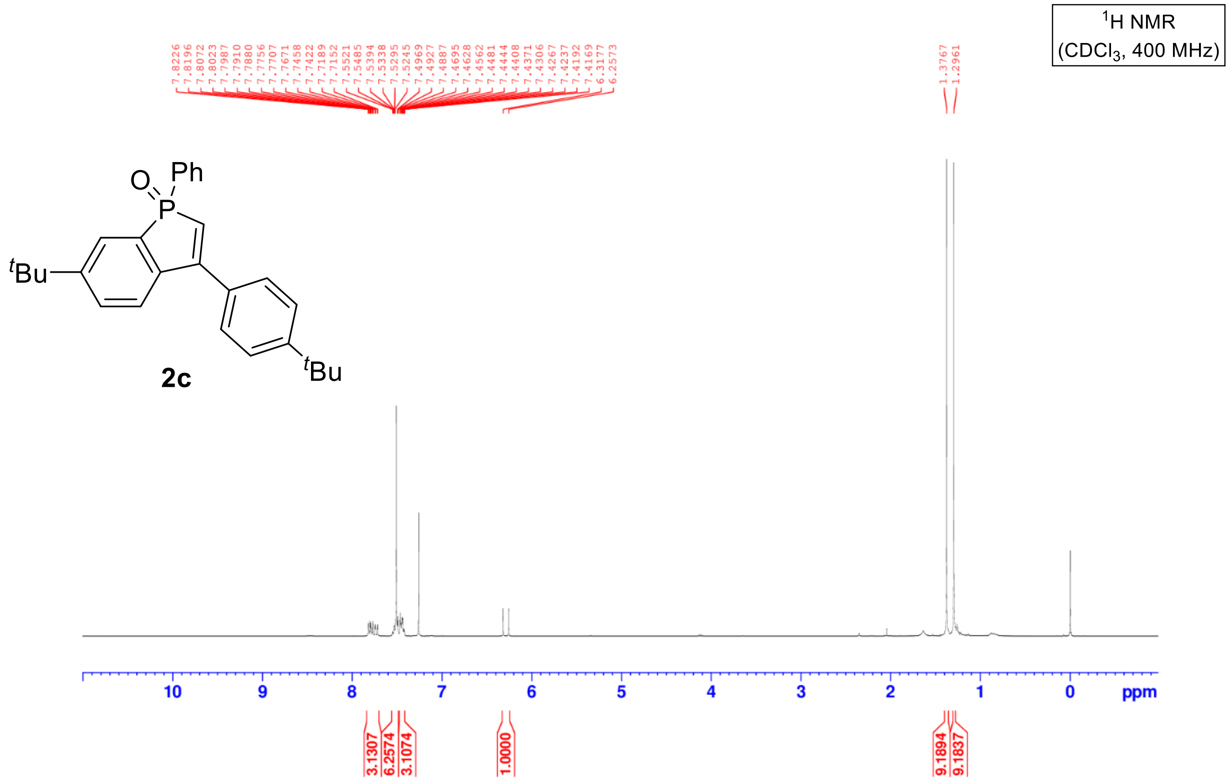


**2c**

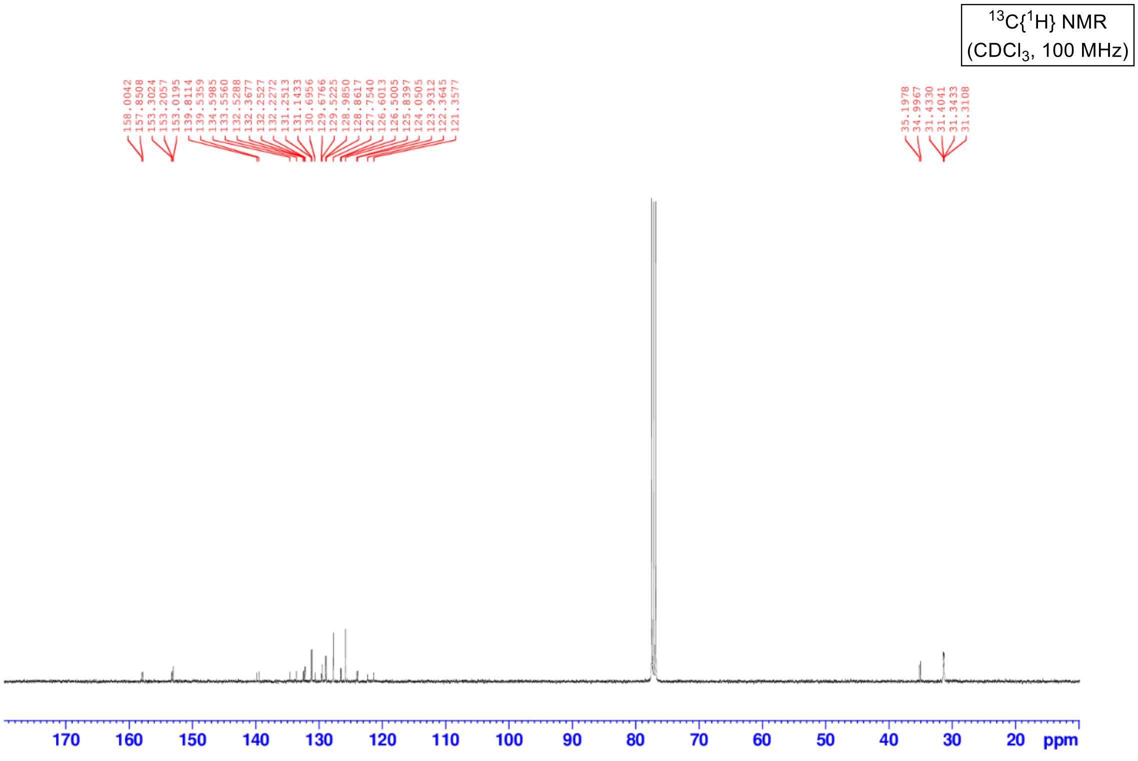

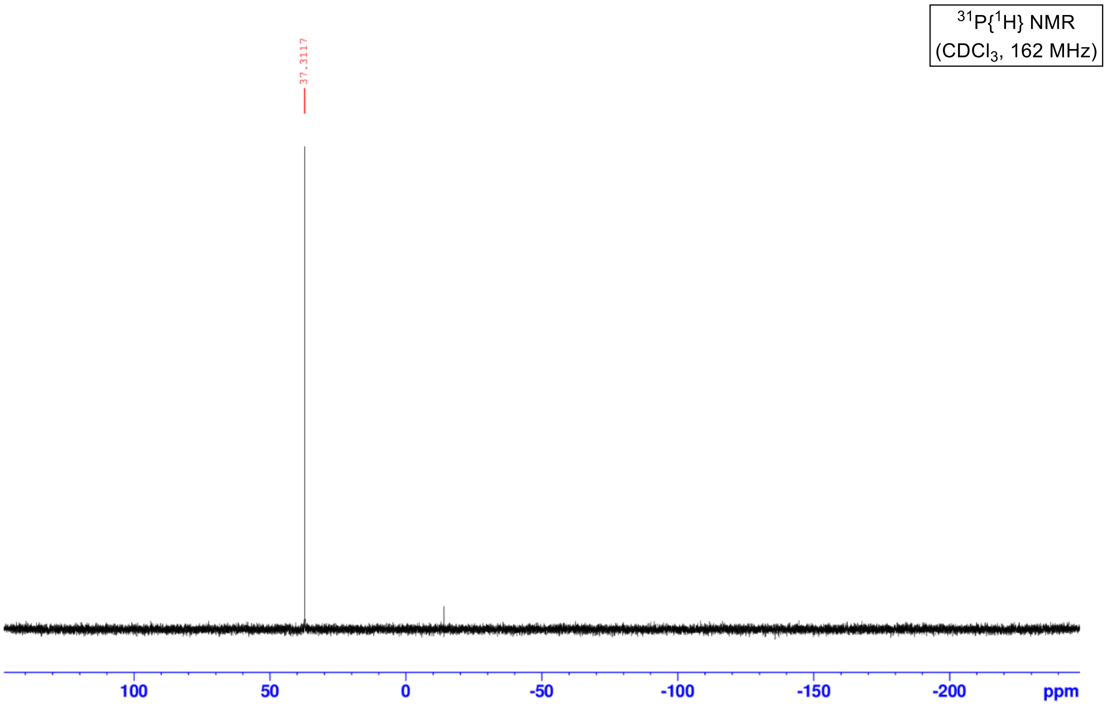

37.3117
$^{31}P\{^{1}H\}$ NMR
($CDCl_3$, 162 MHz)
100
50
0
-50
-100
-150
-200
ppm

[$^{1}H$, $^{13}C\{^{1}H\}$, and $^{31}P\{^{1}H\}$ NMR of **2d**]

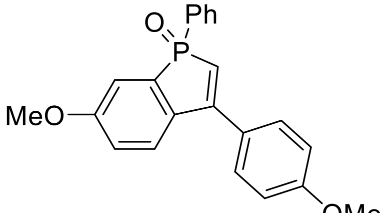


**2d**

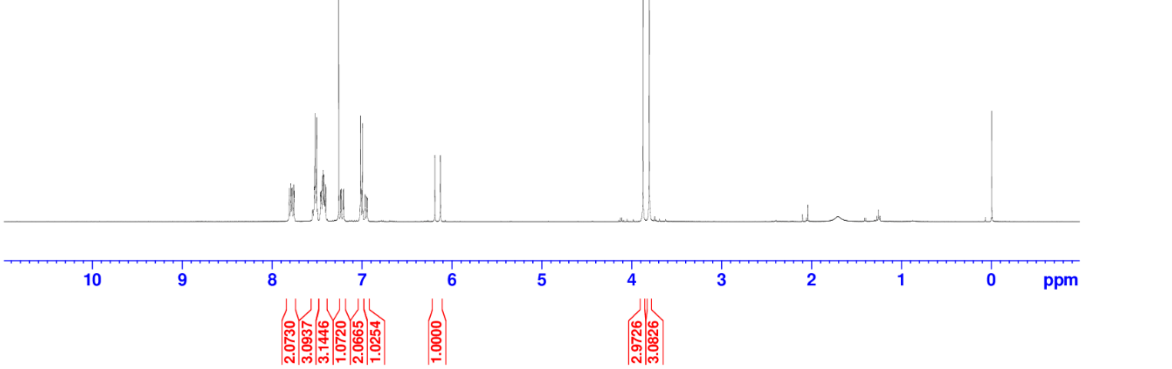


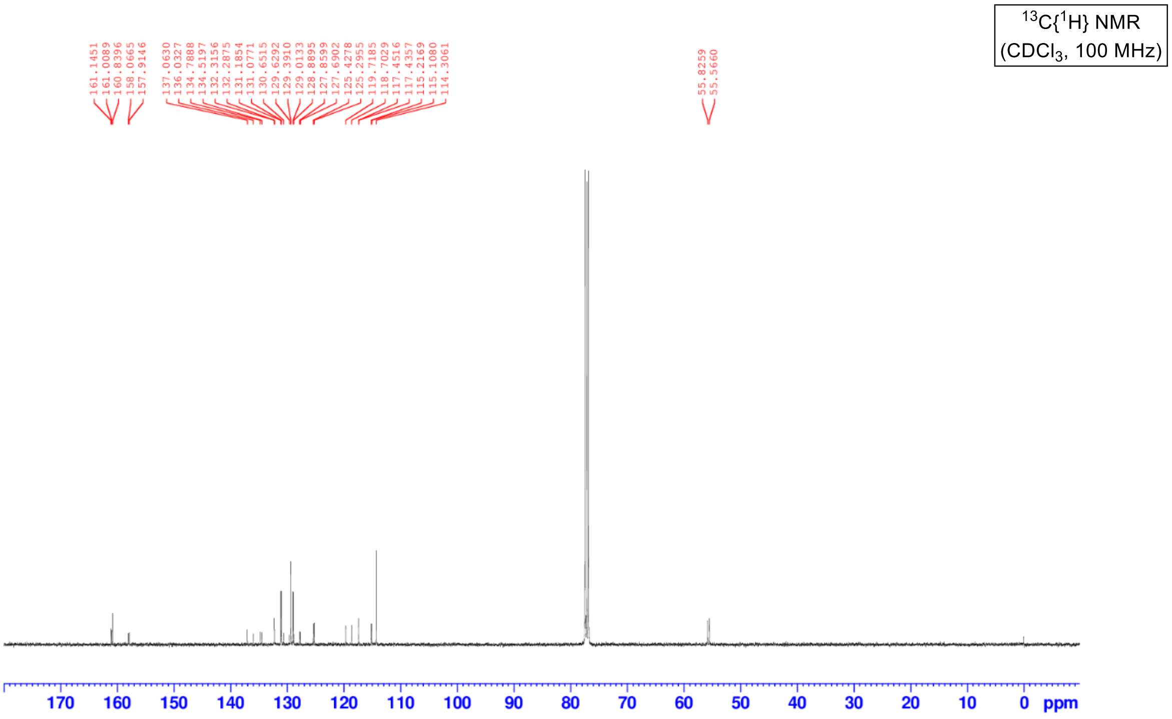

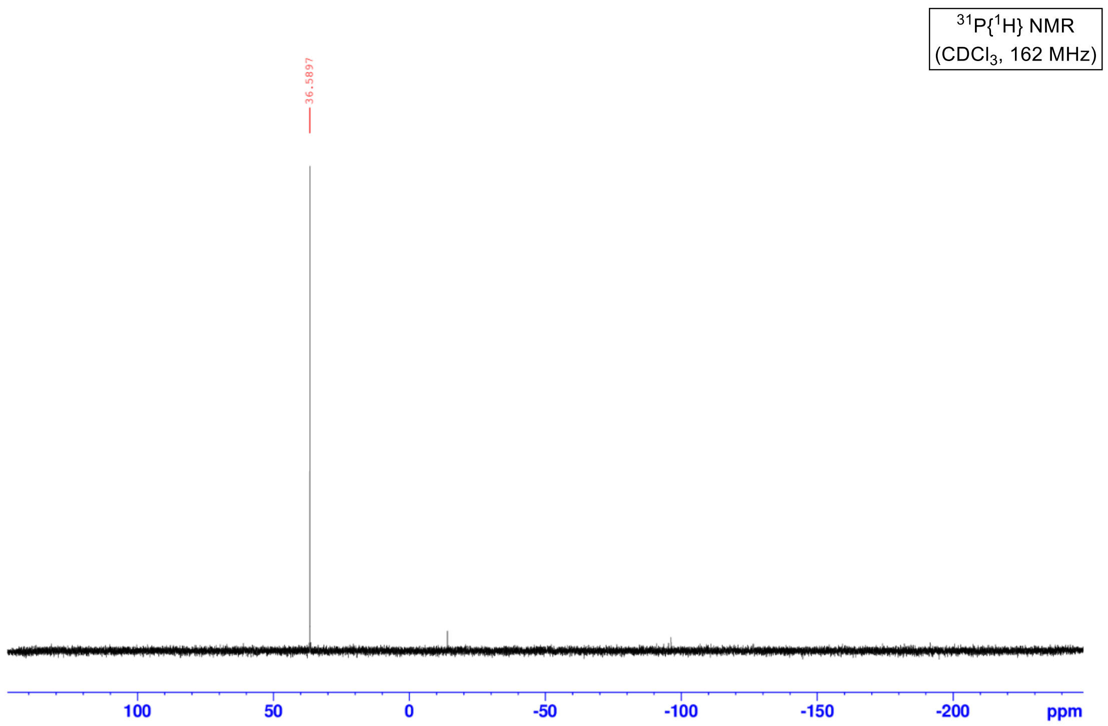

$^{31}P\{^1H\}$ NMR
($CDCl_3$, 162 MHz)
36.5897
100
50
0
-50
-100
-150
-200
ppm

[$^{1}$H, $^{13}$C{$^{1}$H}, $^{19}$F{$^{1}$H}, and $^{31}$P{$^{1}$H} NMR Spectra of **2e**]

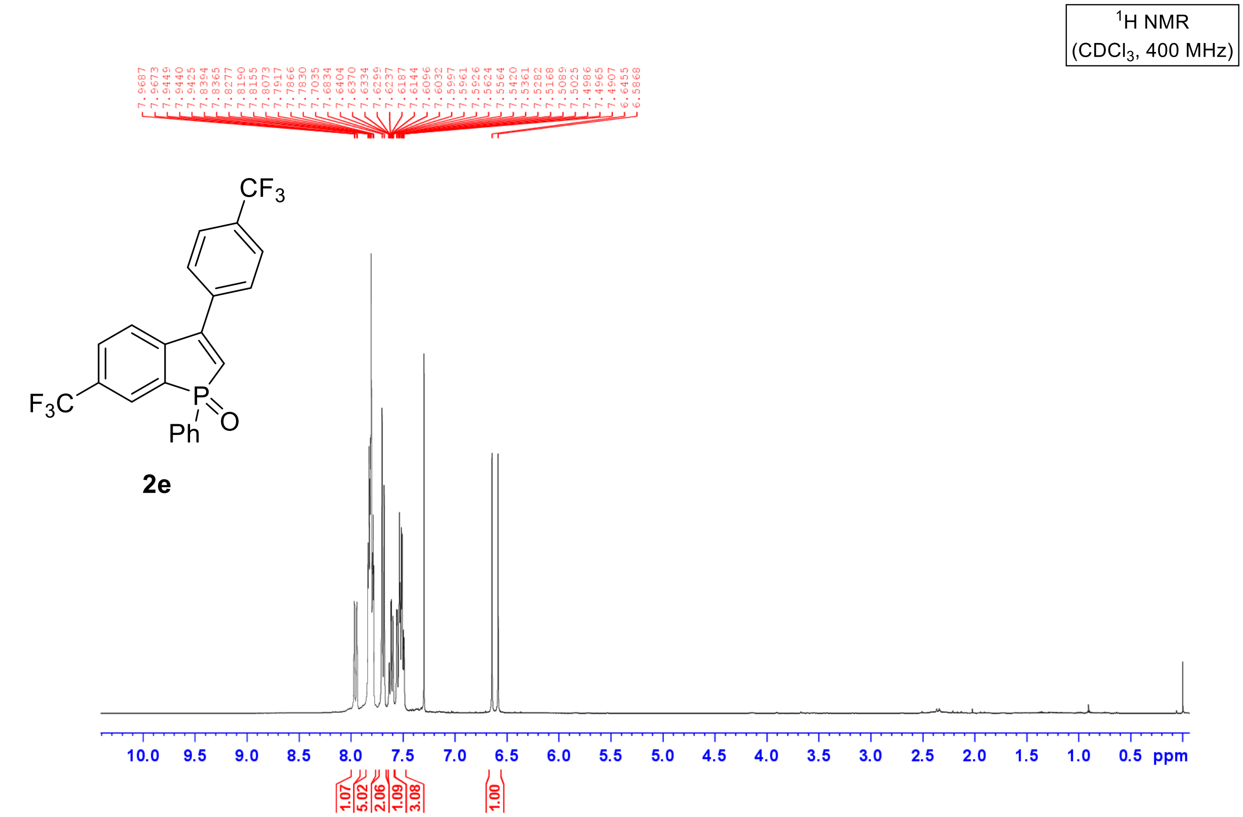


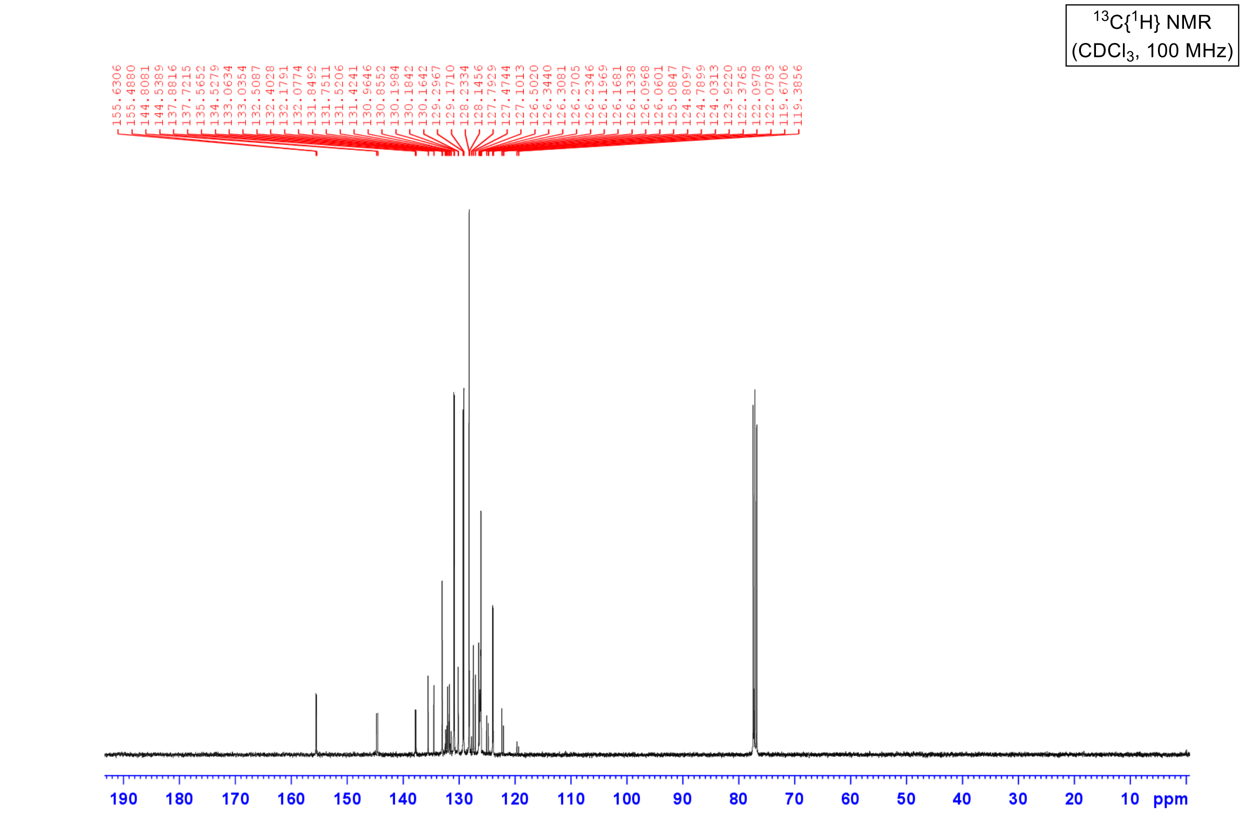

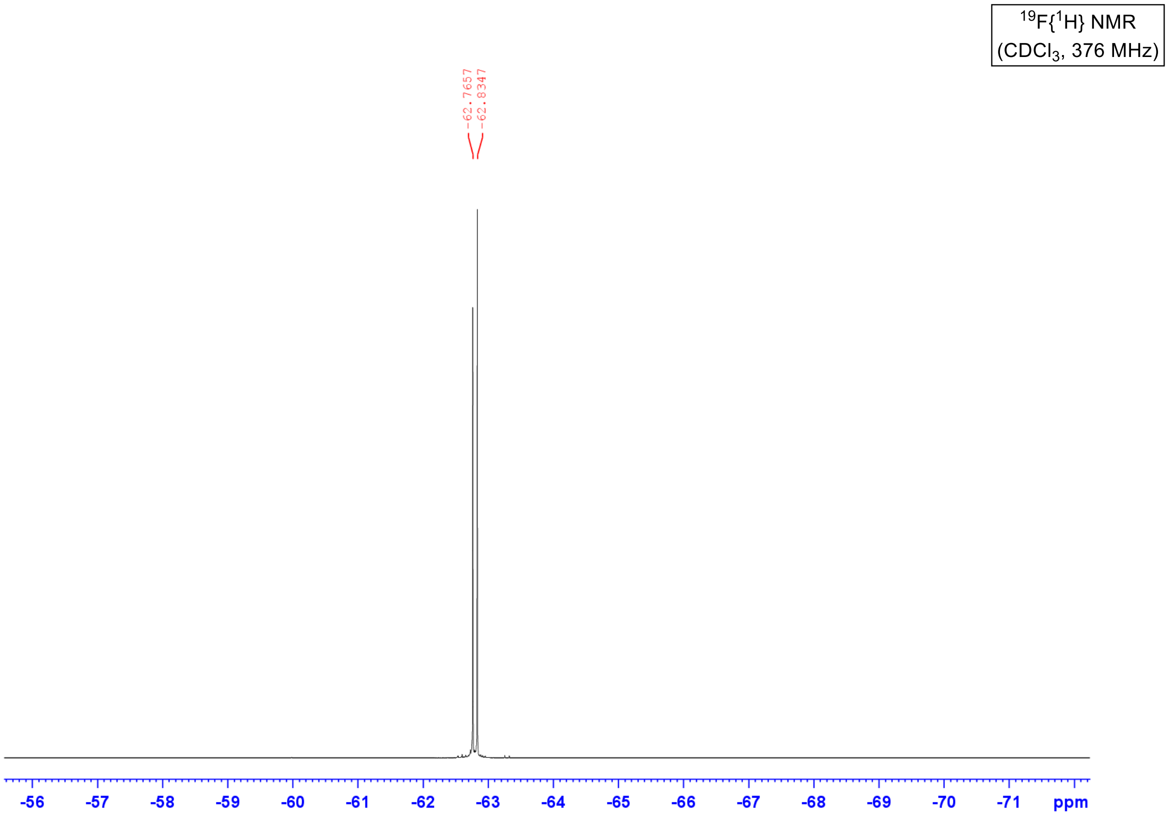

$^{19}F\{^1H\}$ NMR
($CDCl_3$, 376 MHz)
-62.7657
-62.8347
-56 -57 -58 -59 -60 -61 -62 -63 -64 -65 -66 -67 -68 -69 -70 -71 ppm


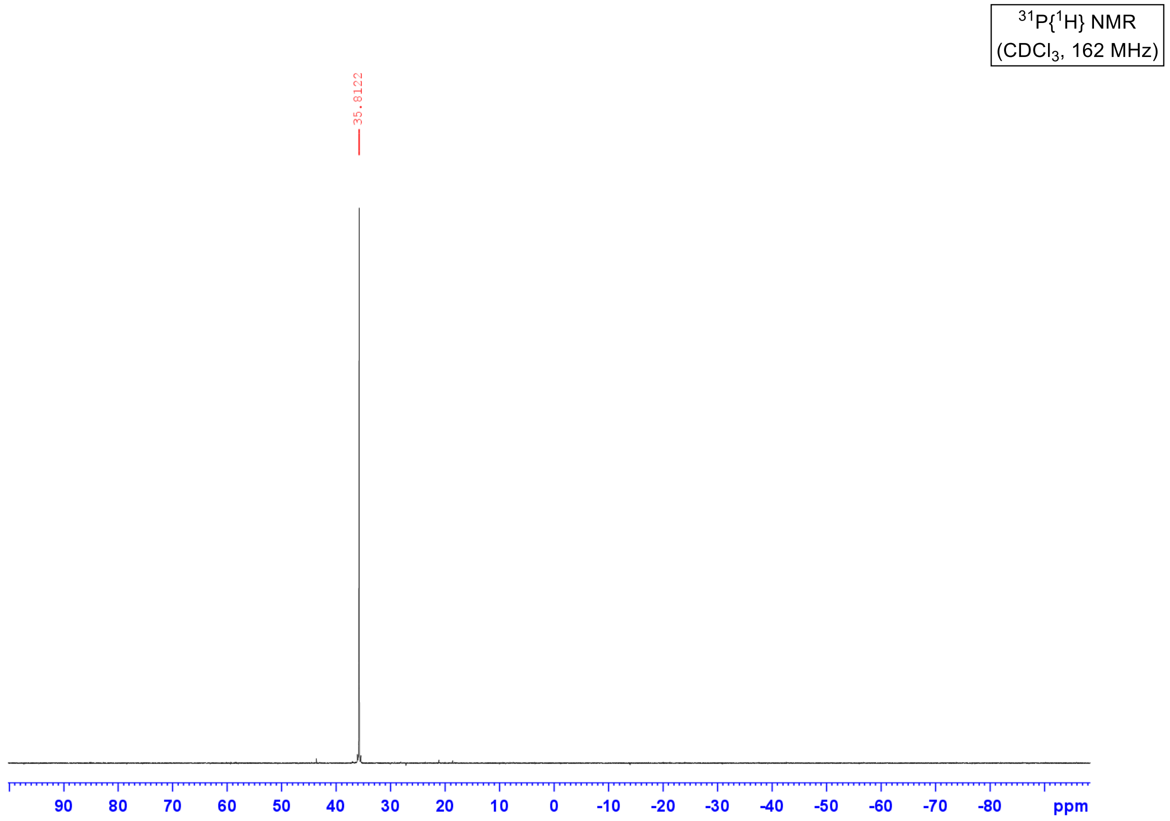

$^{31}P\{^1H\}$ NMR
($CDCl_3$, 162 MHz)
35.8122
90 80 70 60 50 40 30 20 10 0 -10 -20 -30 -40 -50 -60 -70 -80 ppm

[$^{1}$H, $^{13}$C{$^{1}$H}, $^{19}$F{$^{1}$H}, and $^{31}$P{$^{1}$H} NMR Spectra of **2f**]

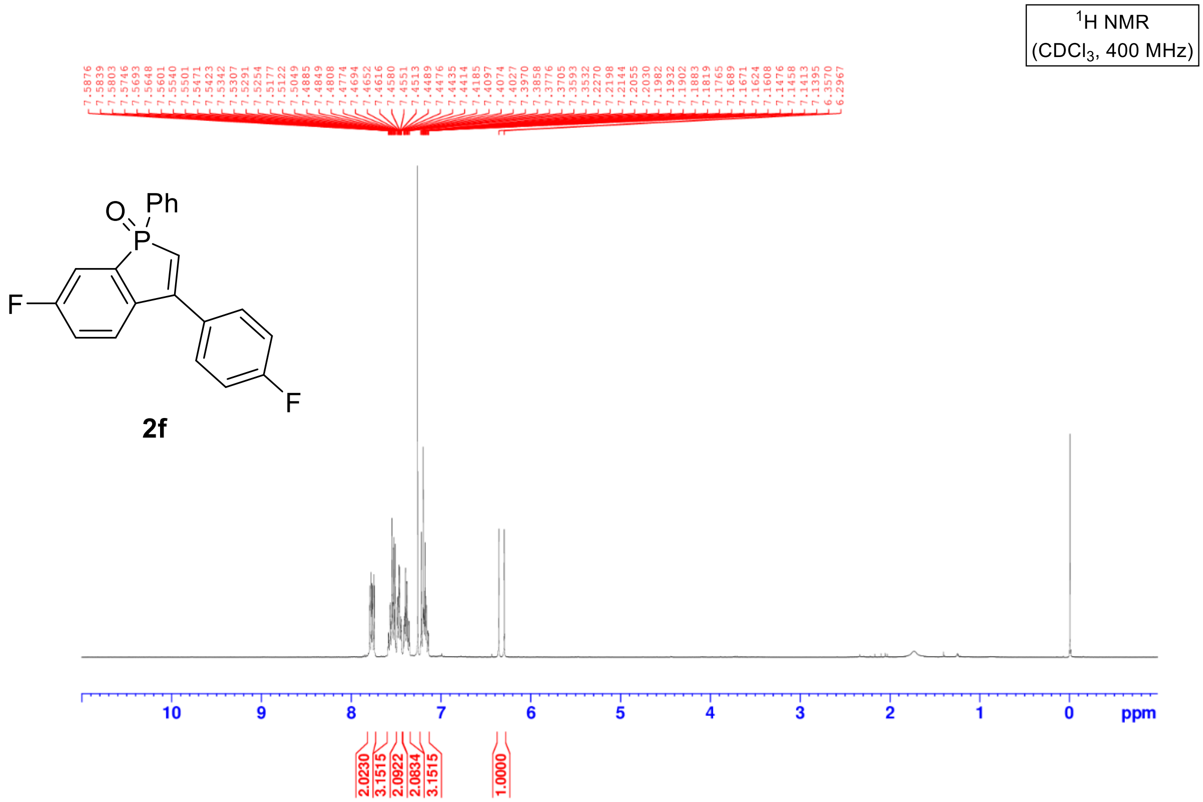


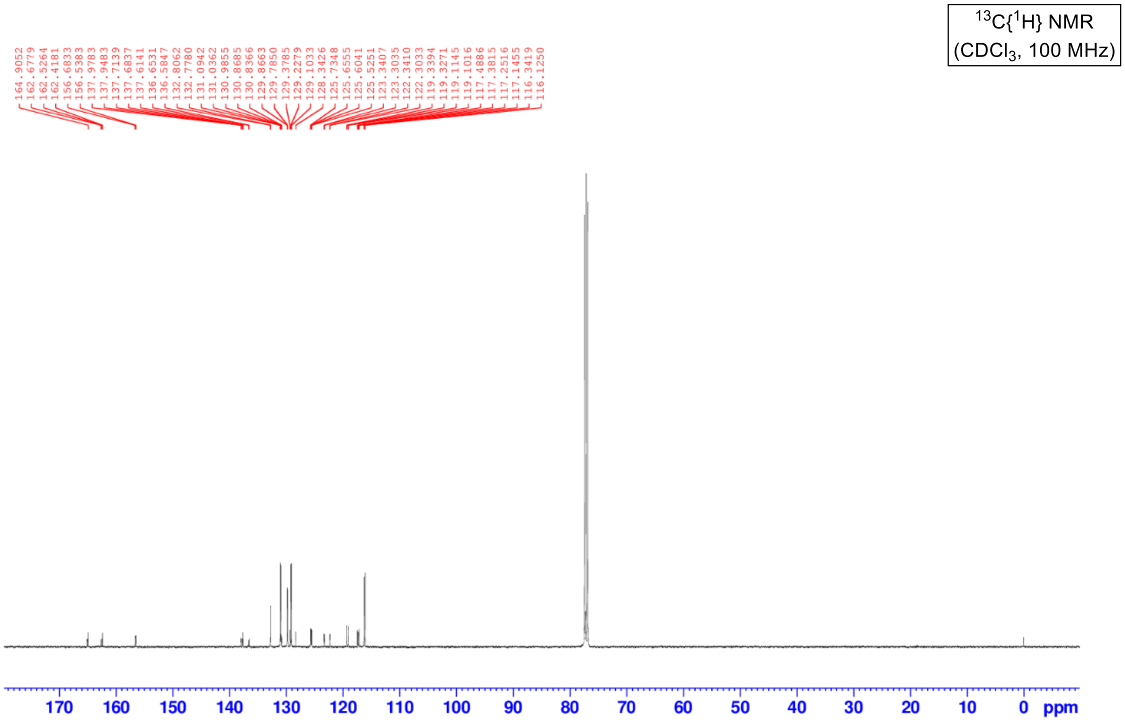

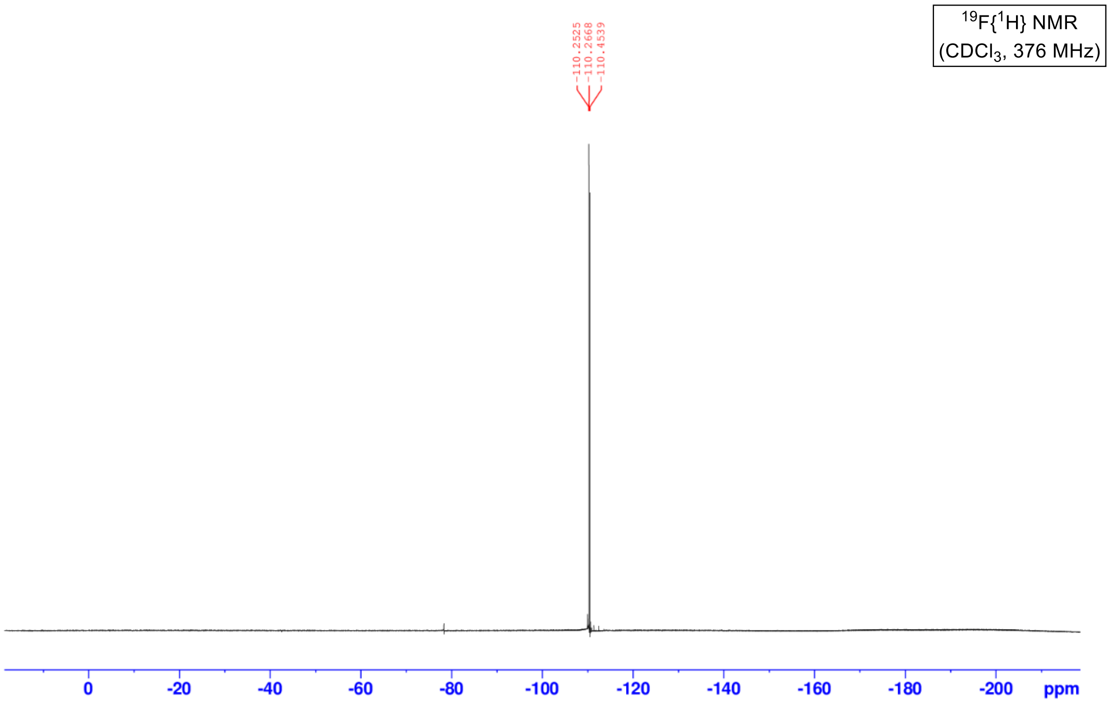


$^{19}F\{^1H\}$ NMR ($CDCl_3$, 376 MHz)

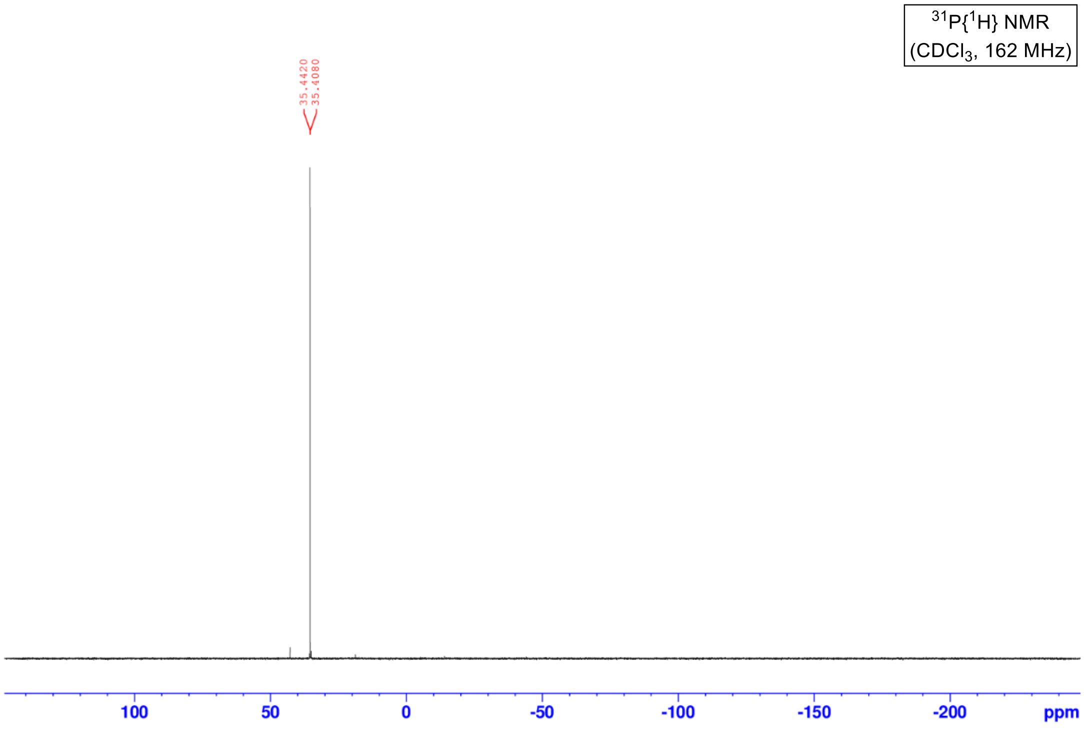


$^{31}P\{^1H\}$ NMR ($CDCl_3$, 162 MHz)

[$^{1}H$, $^{13}C\{^{1}H\}$, and $^{31}P\{^{1}H\}$ NMR Spectra of **2g**]

$^{1}H$ NMR
($CDCl_3$, 400 MHz)

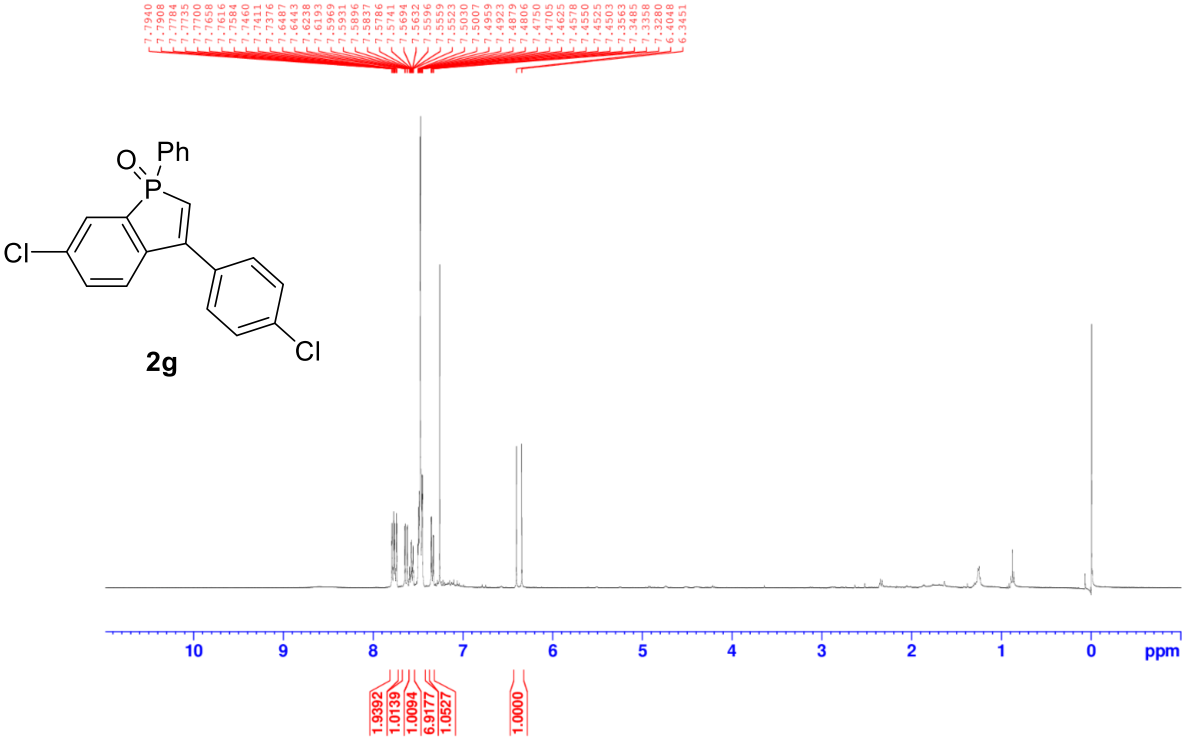


$^{13}C\{^{1}H\}$ NMR
($CDCl_3$, 100 MHz)

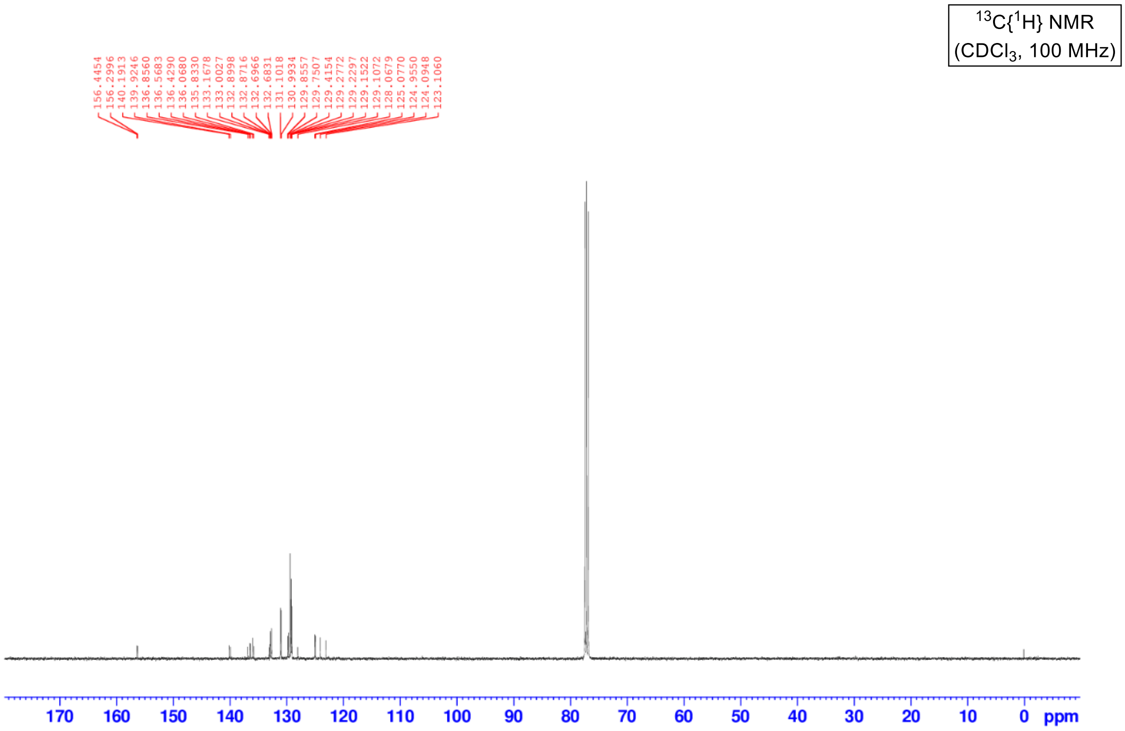

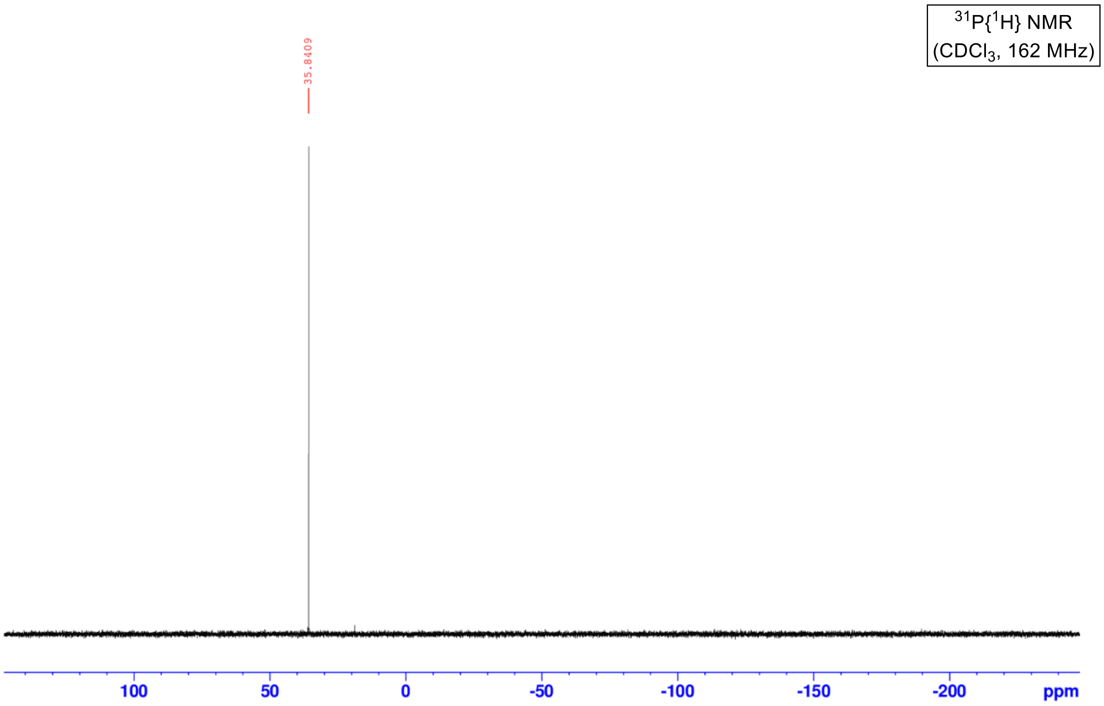
$^{31}P\{^{1}H\}$ NMR
($CDCl_3$, 162 MHz)
35.8409
100
50
0
-50
-100
-150
-200
ppm

[$^{1}H$, $^{13}C\{^{1}H\}$, and $^{31}P\{^{1}H\}$ NMR Spectra of **2h**]

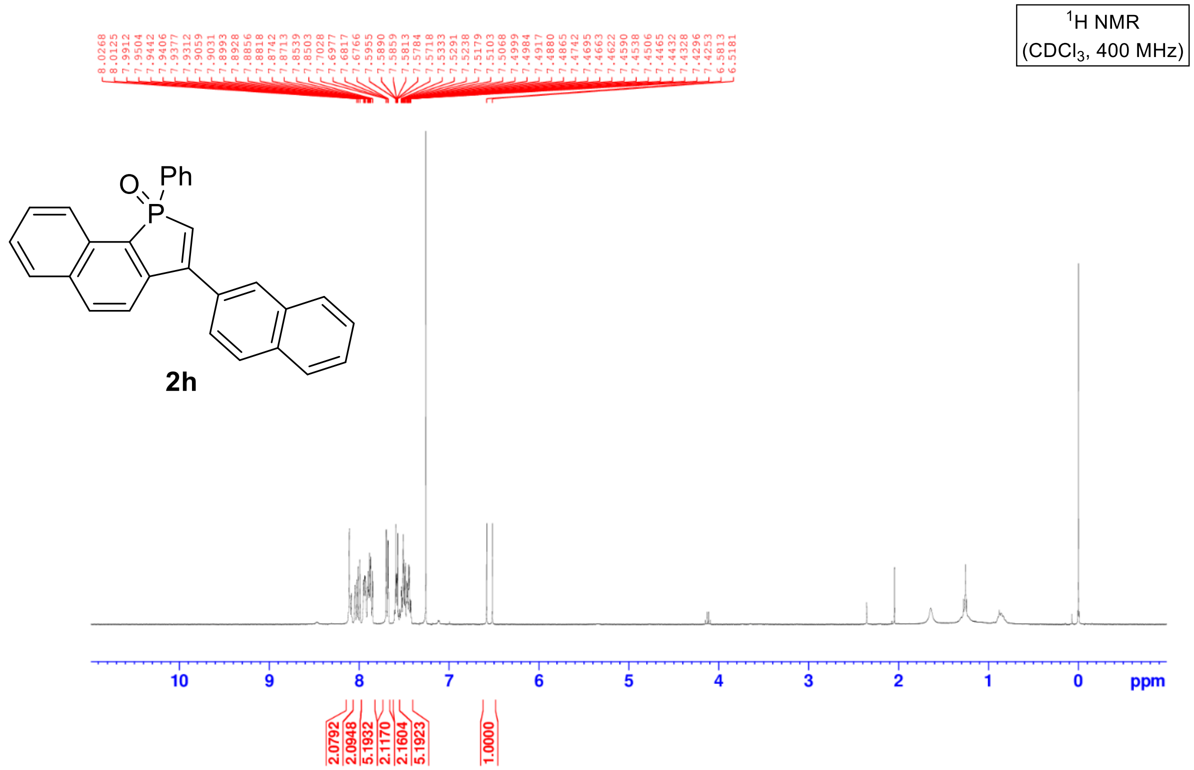


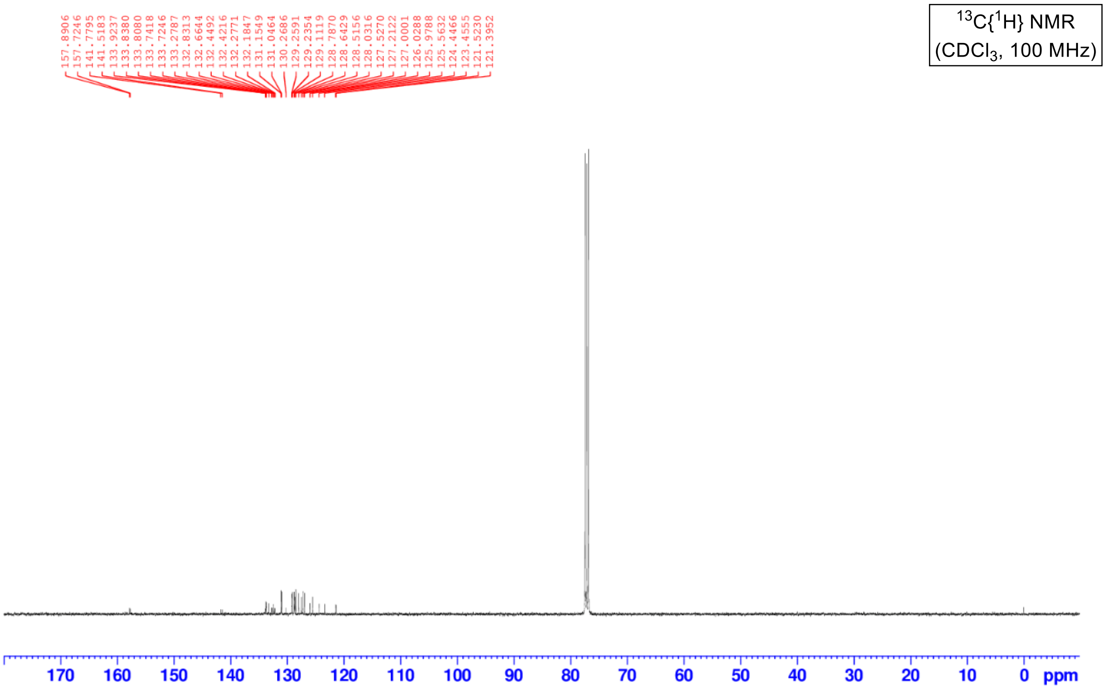

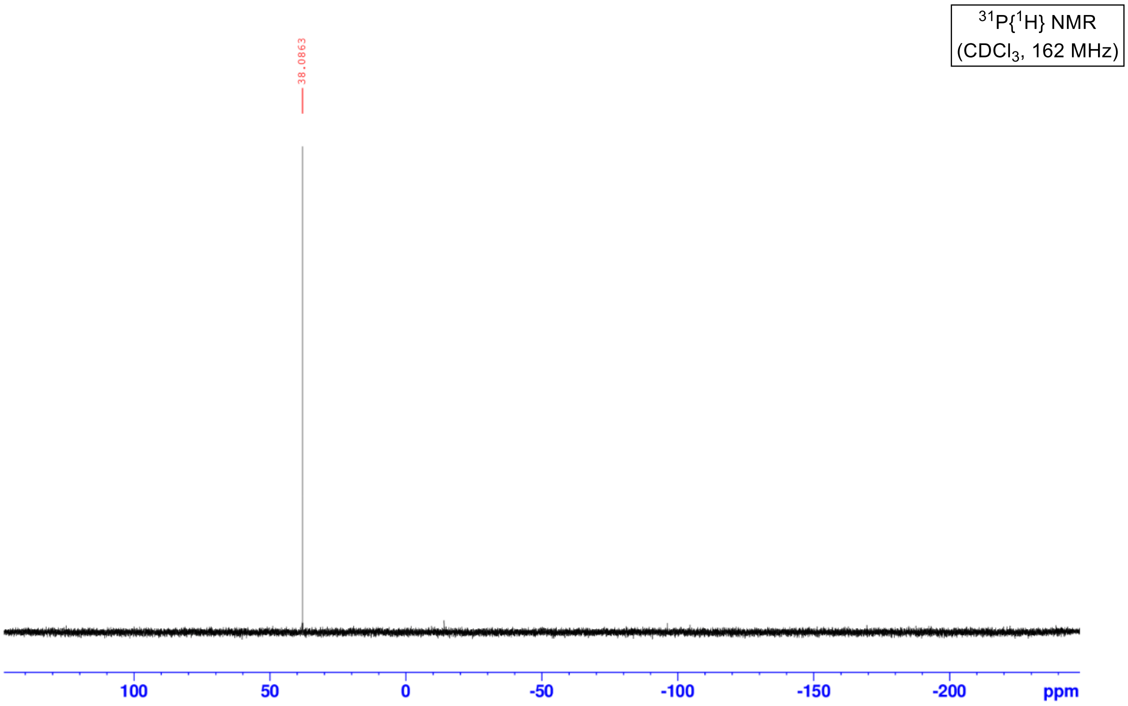
38.0863
$^{31}P\{^1H\}$ NMR
($CDCl_3$, 162 MHz)
100
50
0
-50
-100
-150
-200
ppm

[$^{1}H$, $^{13}C\{^{1}H\}$, and $^{31}P\{^{1}H\}$ NMR Spectra of **2i**]

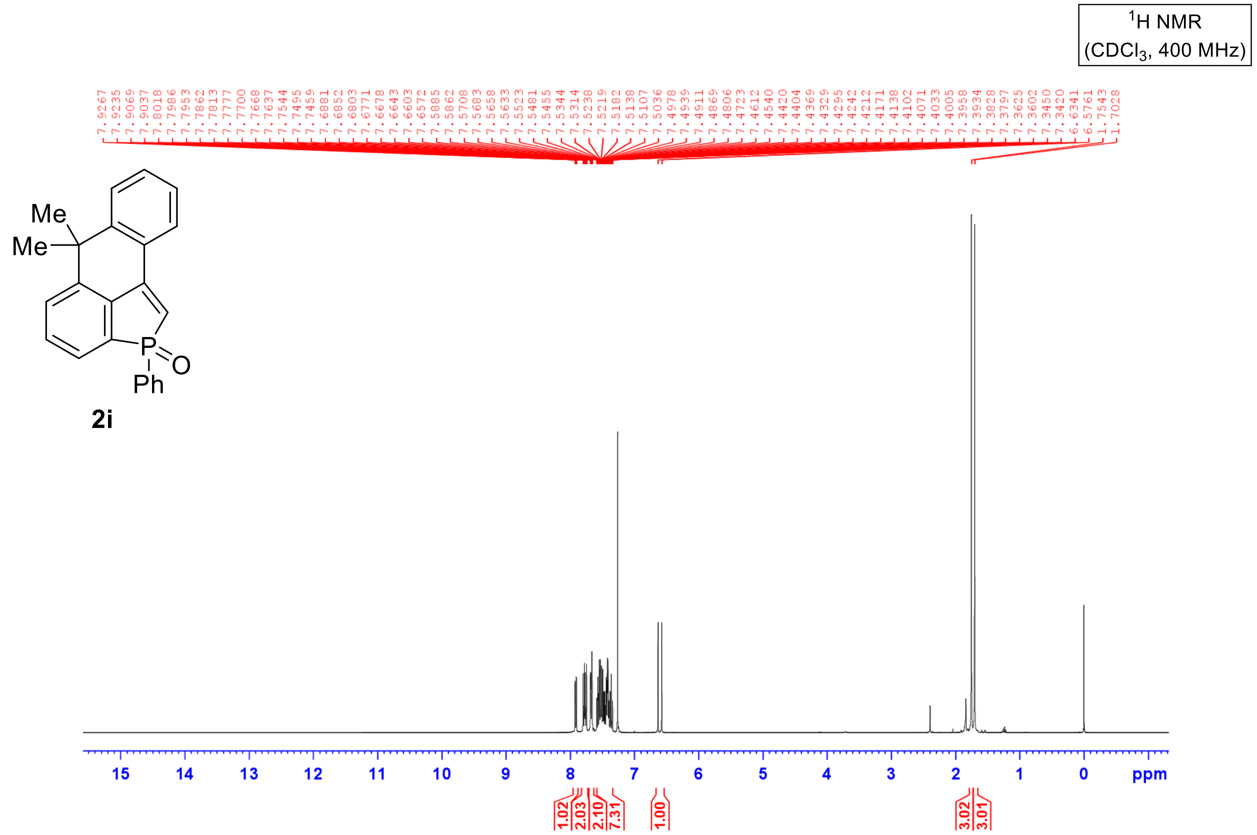


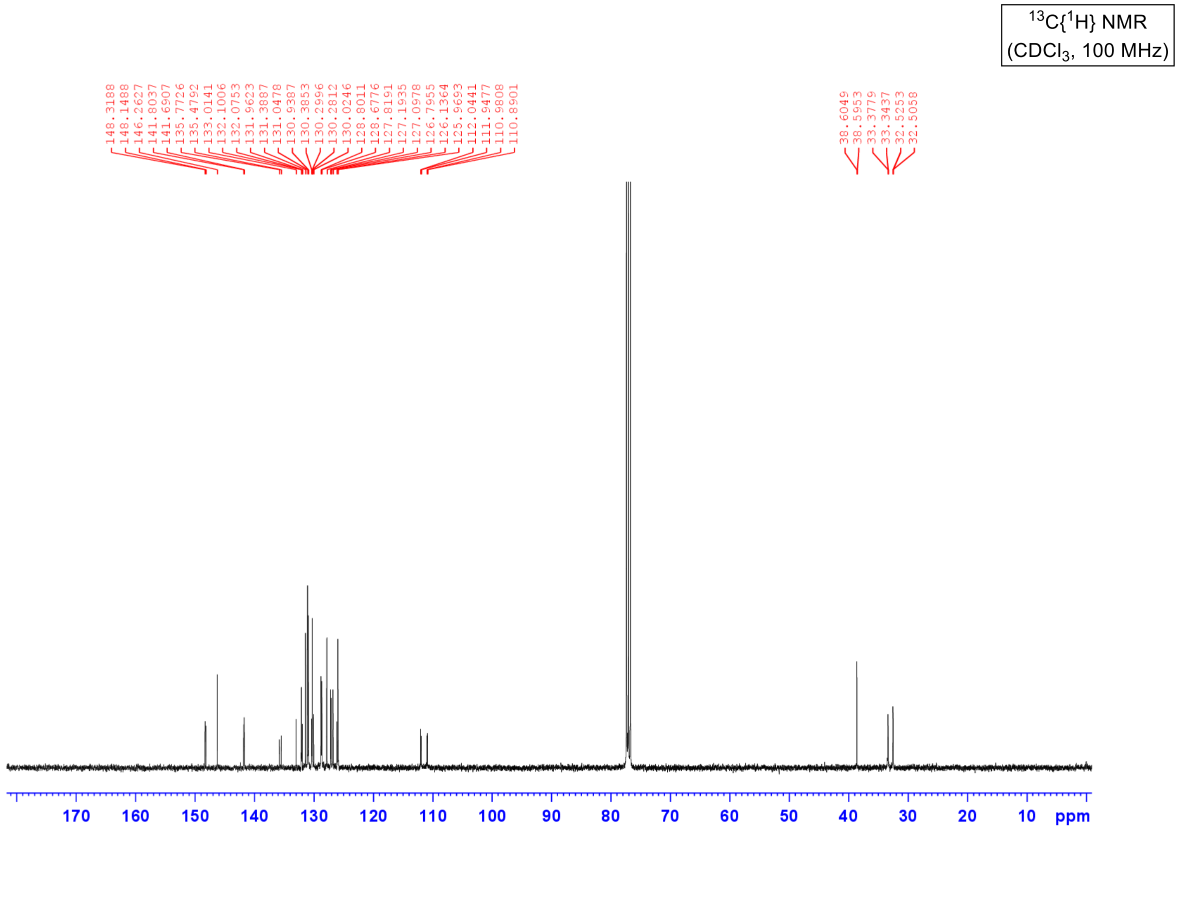

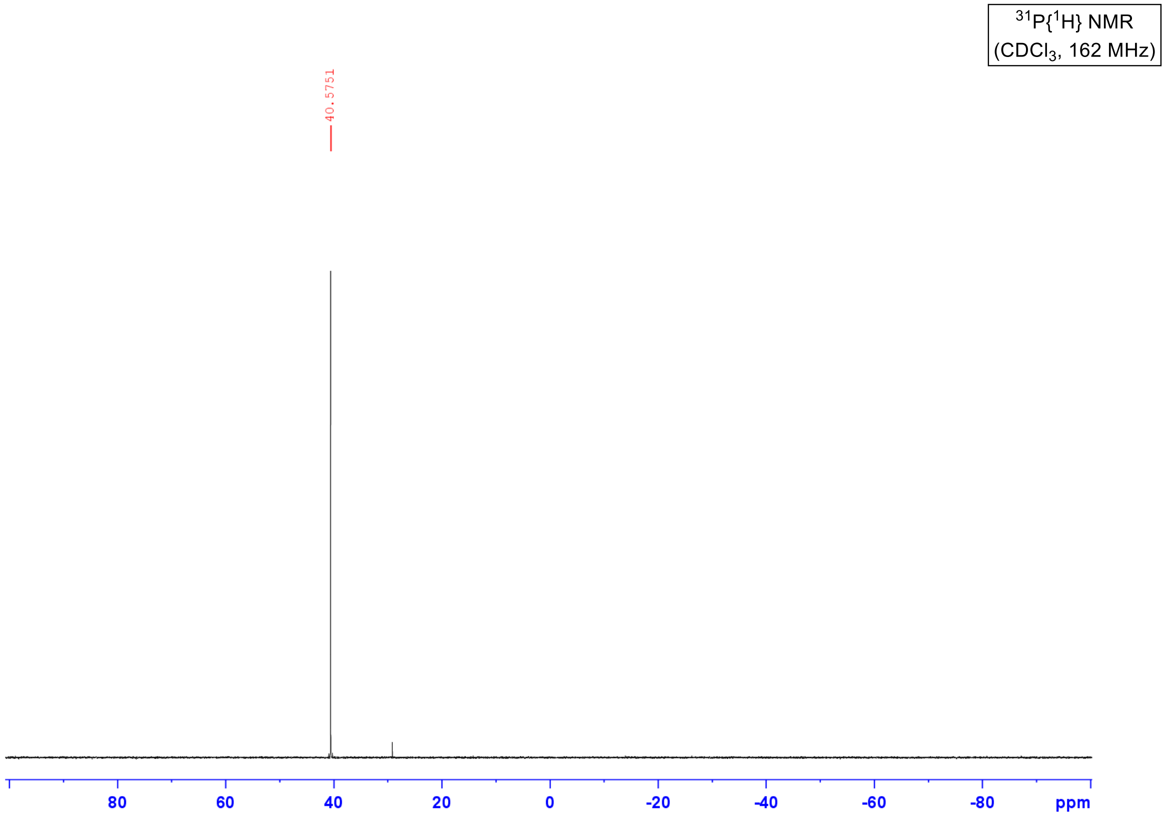
$^{31}P\{^{1}H\}$ NMR
($CDCl_3$, 162 MHz)
40.5751
80
60
40
20
0
-20
-40
-60
-80
ppm

[$^{1}H$, $^{13}C\{^{1}H\}$, and $^{31}P\{^{1}H\}$ NMR Spectra of **2j**]

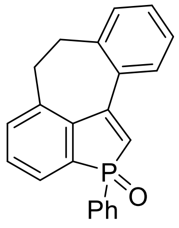

**2j**

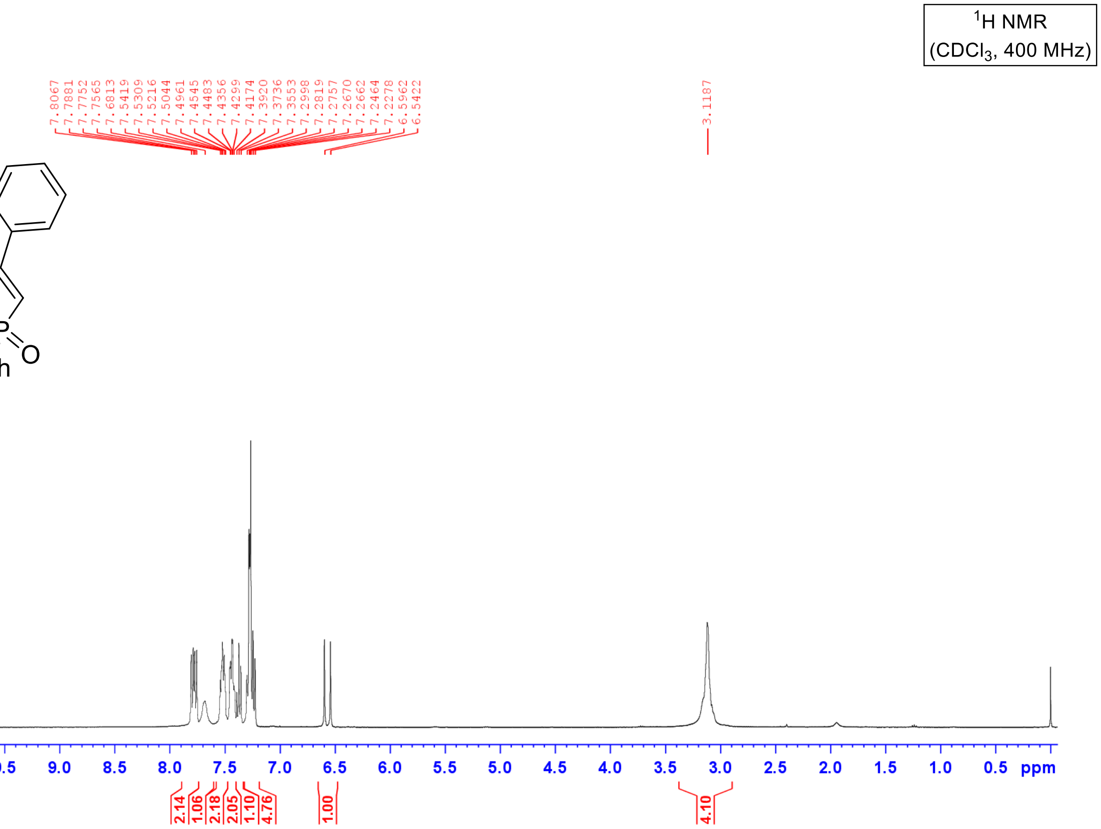


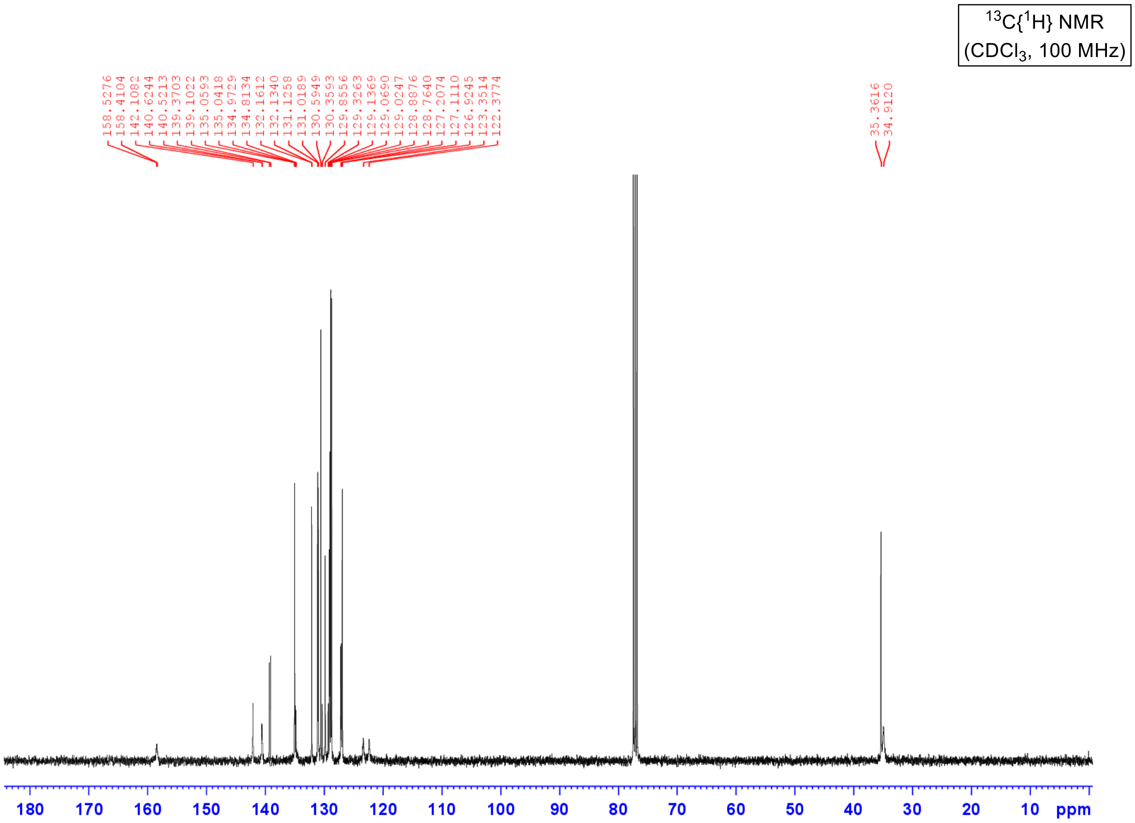

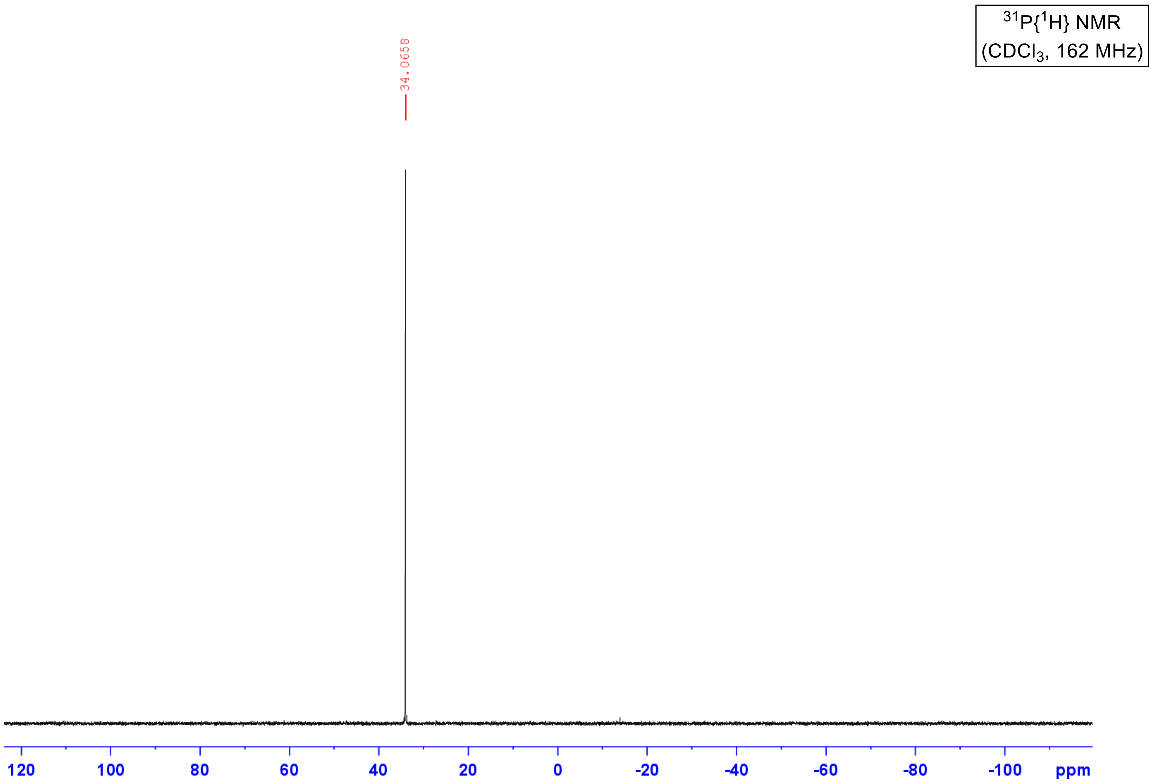
$^{31}P\{^1H\}$ NMR
($CDCl_3$, 162 MHz)
34.0658
120
100
80
60
40
20
0
-20
-40
-60
-80
-100
ppm

[$^{1}H$, $^{13}C\{^{1}H\}$, $^{19}F\{^{1}H\}$, and $^{31}P\{^{1}H\}$ NMR Spectra of **2k**]

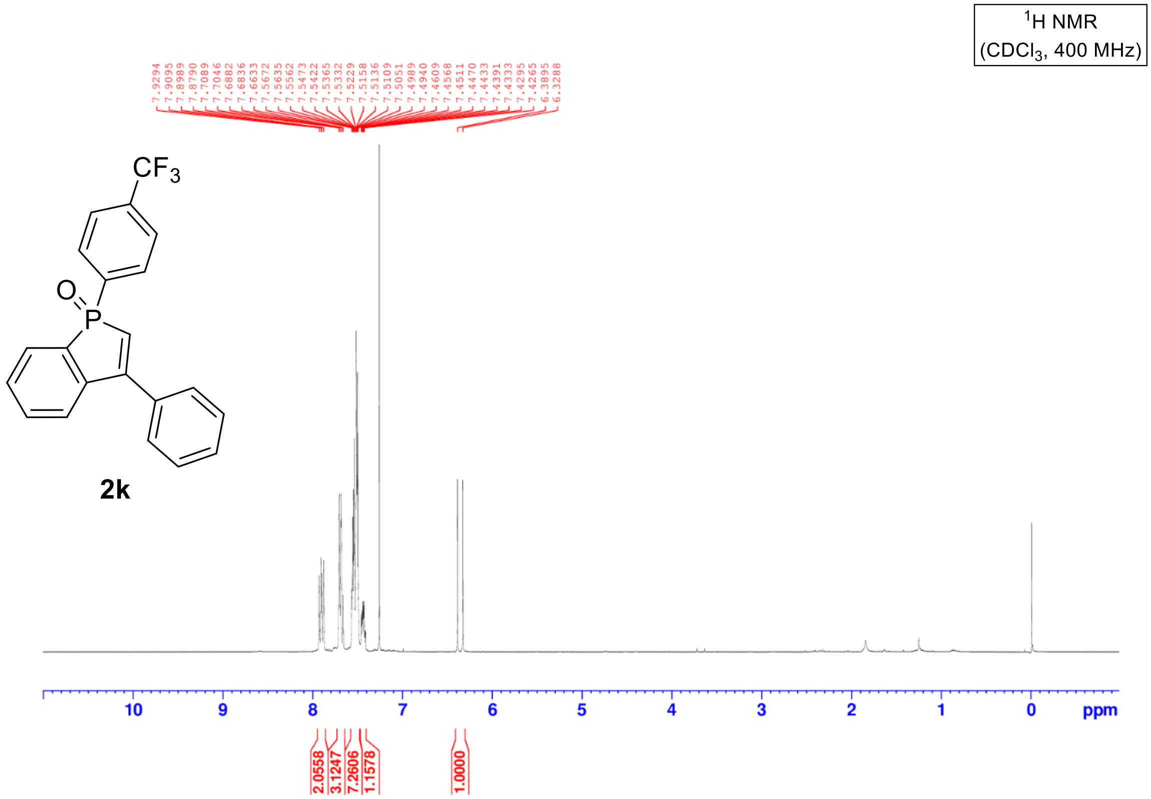


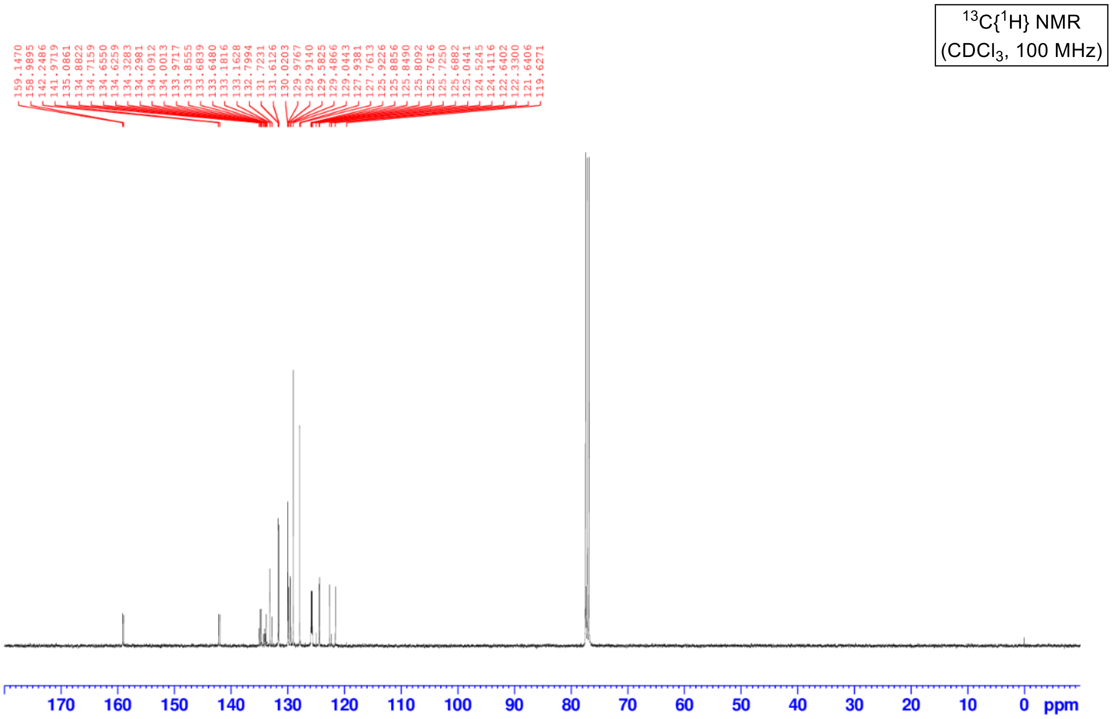

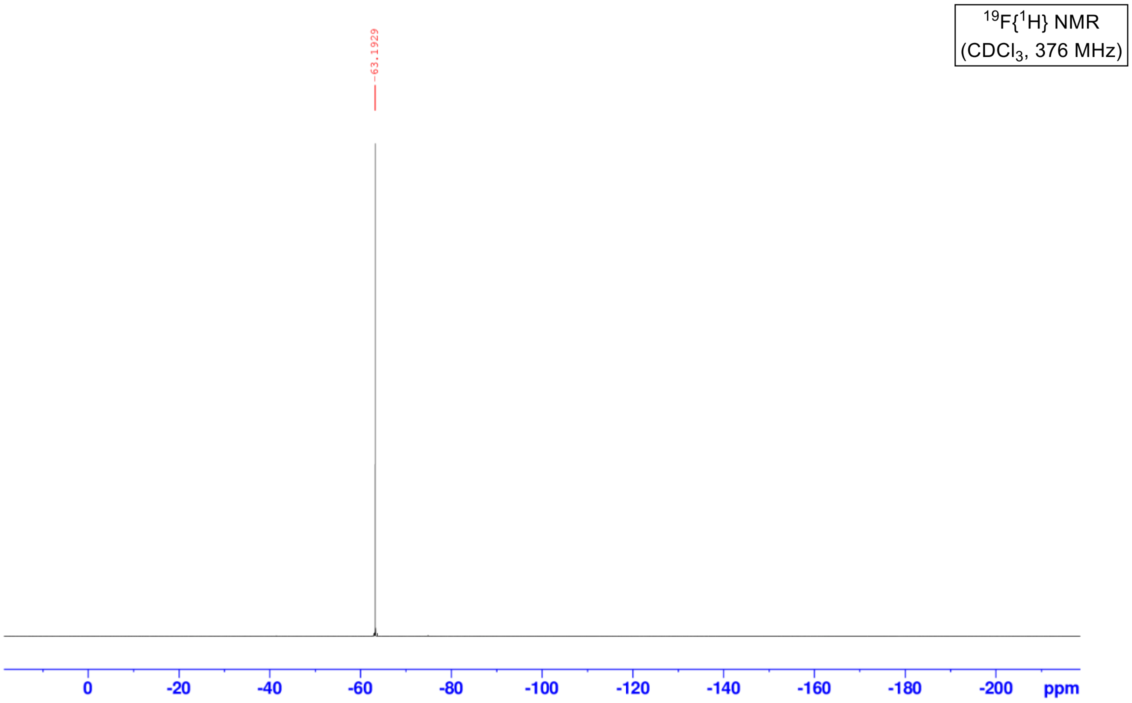

-63.1929
$^{19}F\{^{1}H\}$ NMR
($CDCl_3$, 376 MHz)
0
-20
-40
-60
-80
-100
-120
-140
-160
-180
-200
ppm


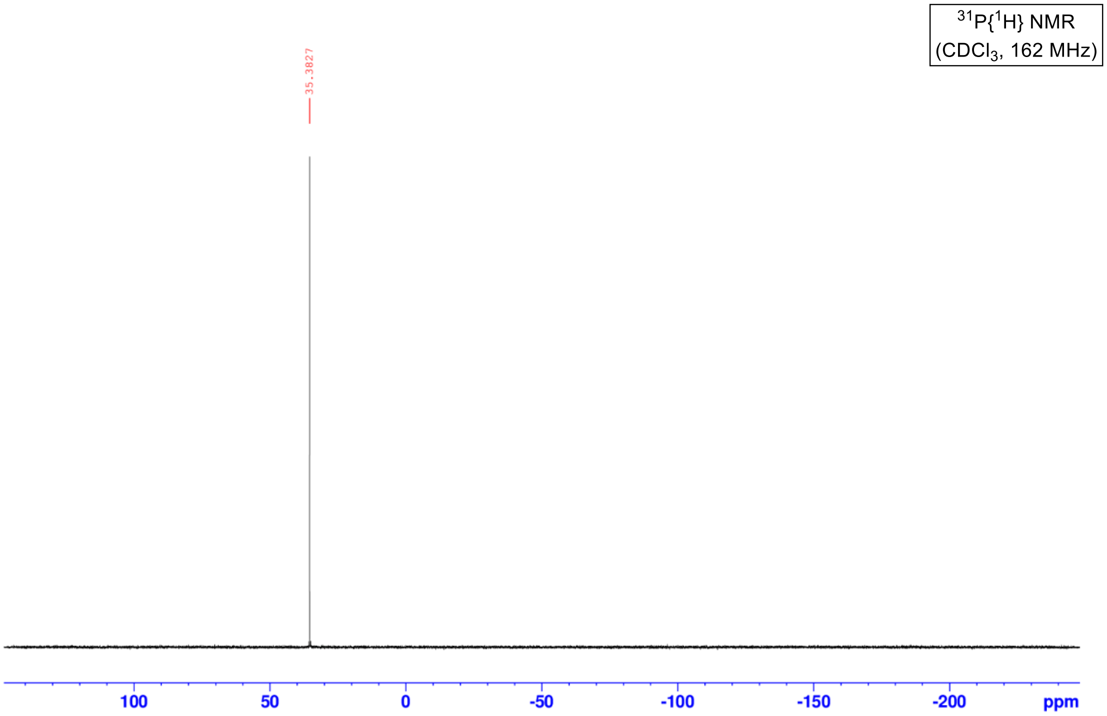

35.3827
$^{31}P\{^{1}H\}$ NMR
($CDCl_3$, 162 MHz)
100
50
0
-50
-100
-150
-200
ppm

[$^{1}H$, $^{13}C\{^{1}H\}$, and $^{31}P\{^{1}H\}$ NMR Spectra of **2l**]

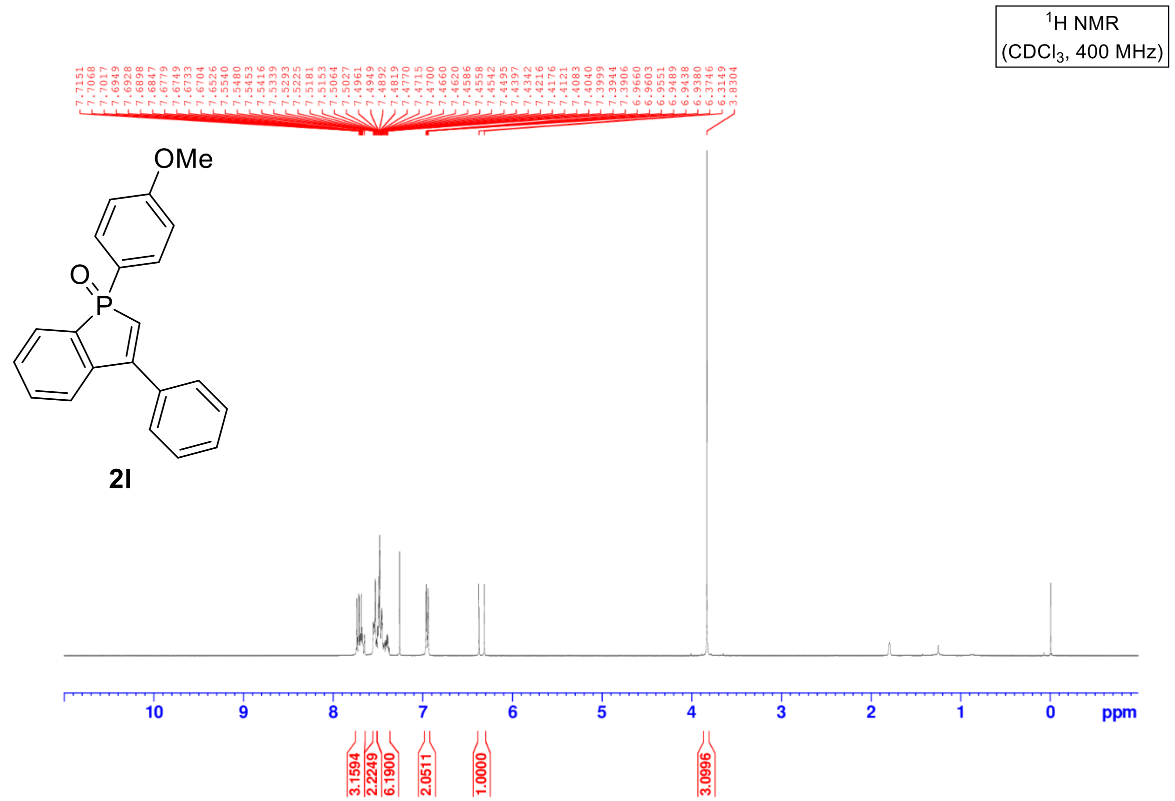


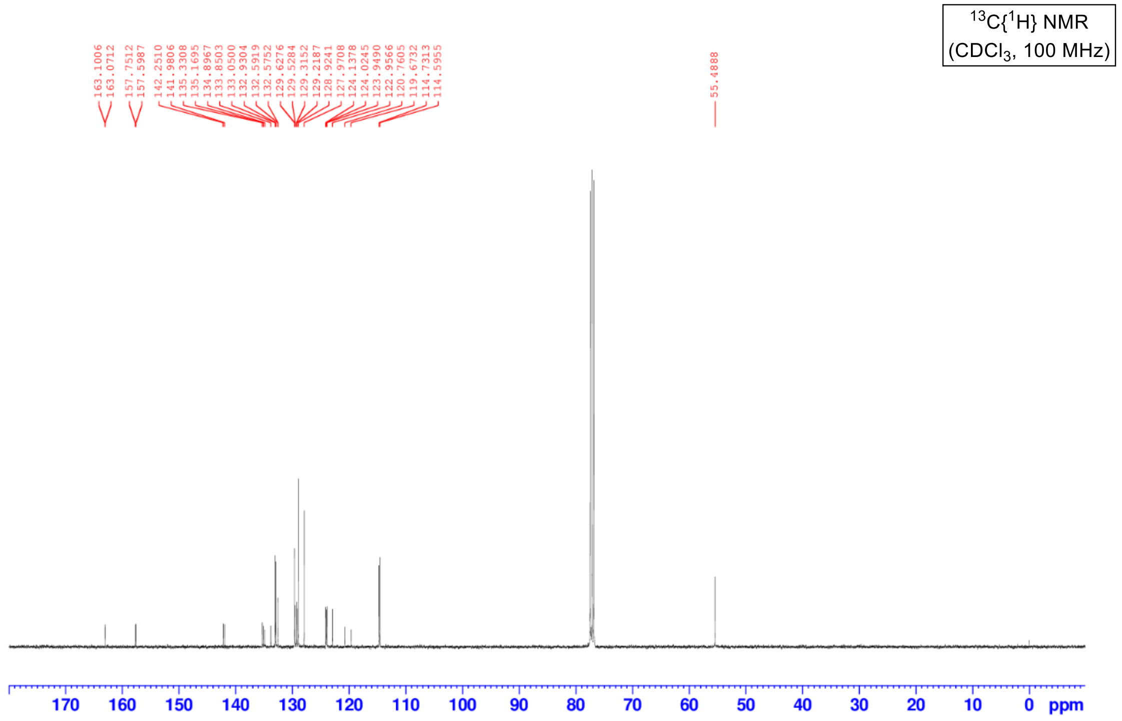

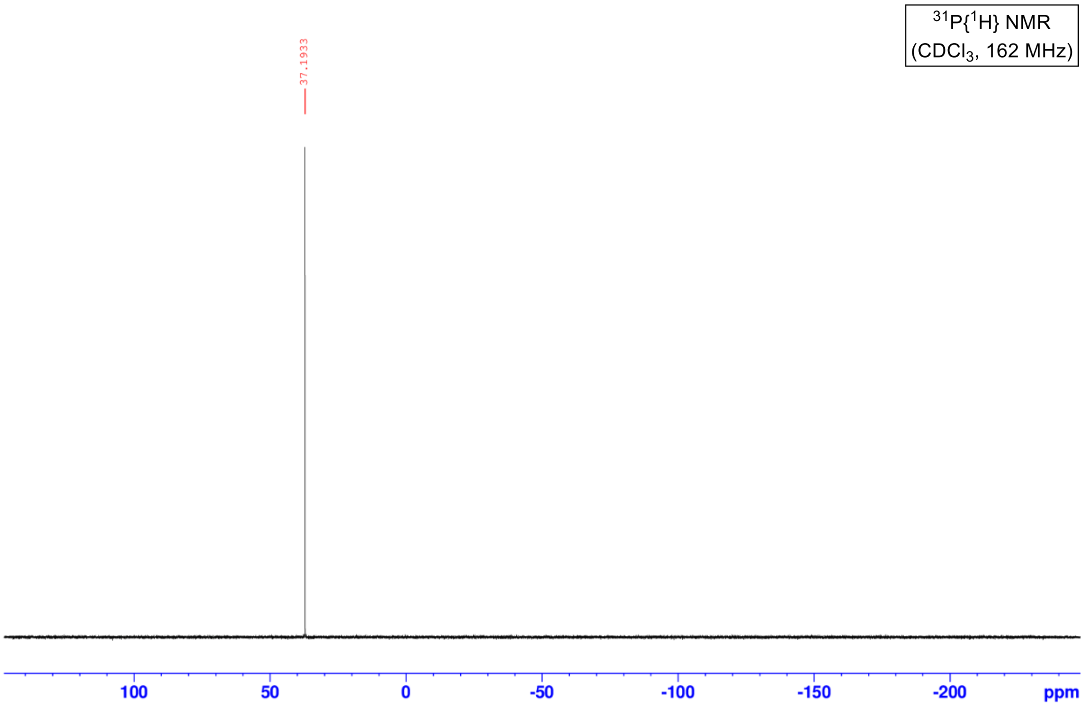
37.1933
$^{31}P\{^1H\}$ NMR
($CDCl_3$, 162 MHz)
100
50
0
-50
-100
-150
-200
ppm

[$^{1}H$, $^{13}C\{^{1}H\}$, and $^{31}P\{^{1}H\}$ NMR Spectra of **2m** + **2m'**]

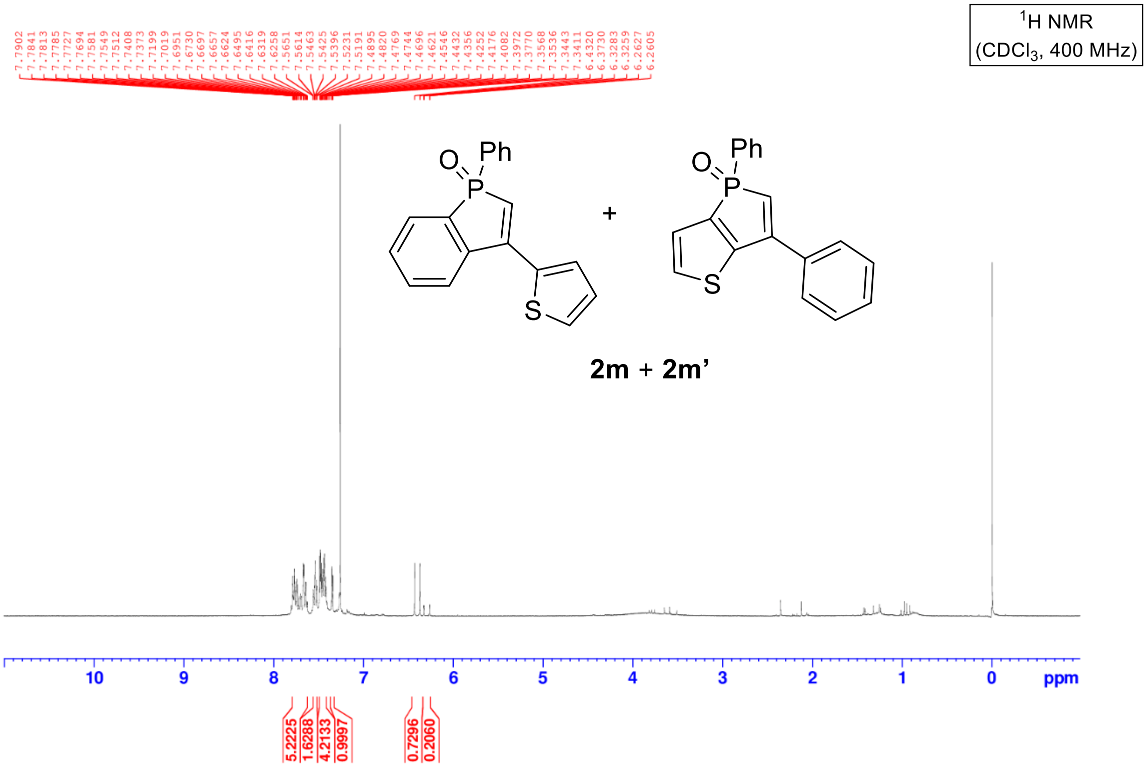


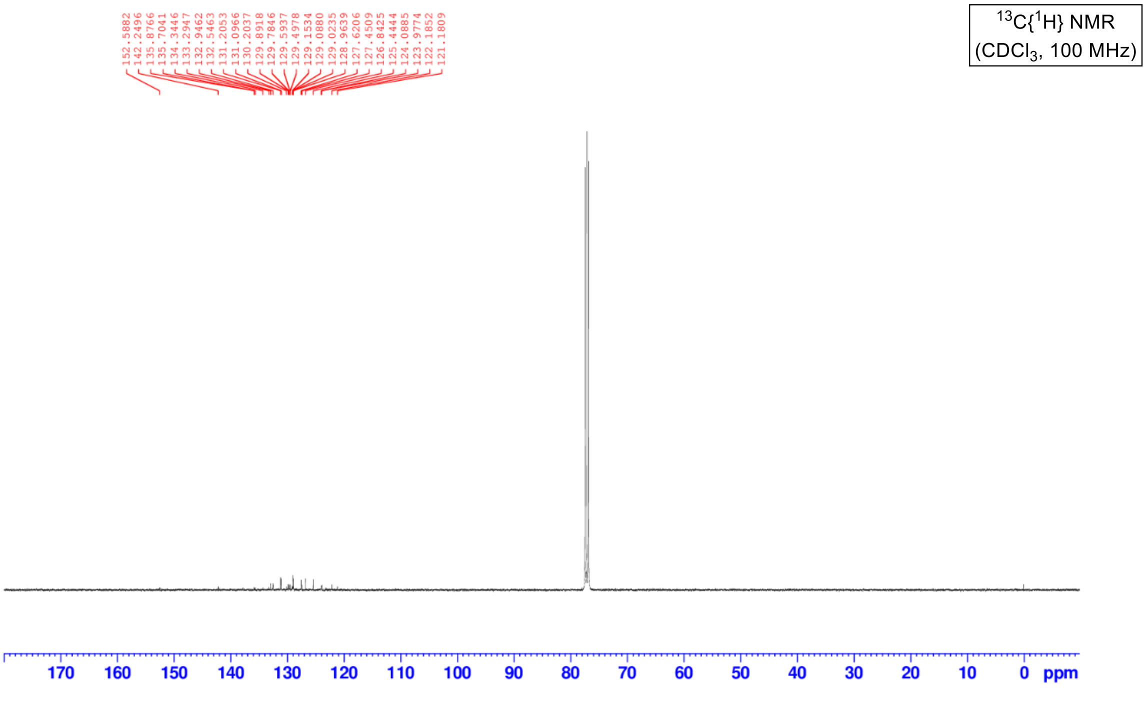

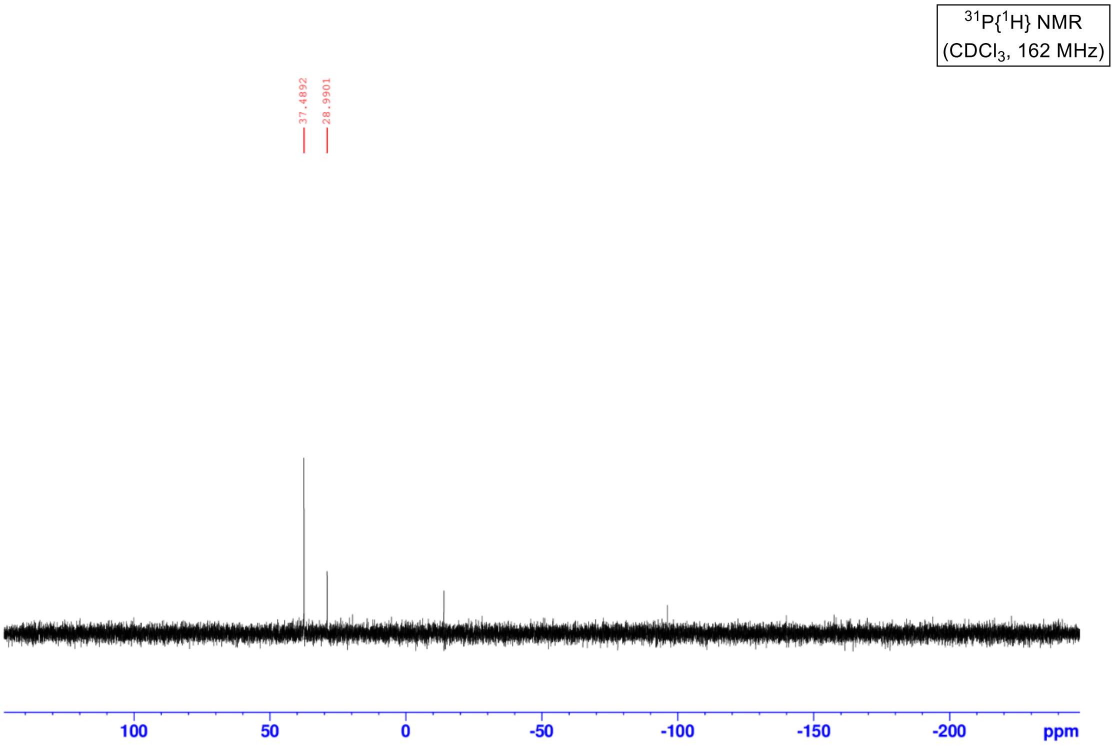
$^{31}P\{^1H\}$ NMR
($CDCl_3$, 162 MHz)
37.4892
28.9901
100
50
0
-50
-100
-150
-200
ppm

[$^{1}$H, $^{13}$C{$^{1}$H}, and $^{31}$P{$^{1}$H} NMR Spectra of **2n**]

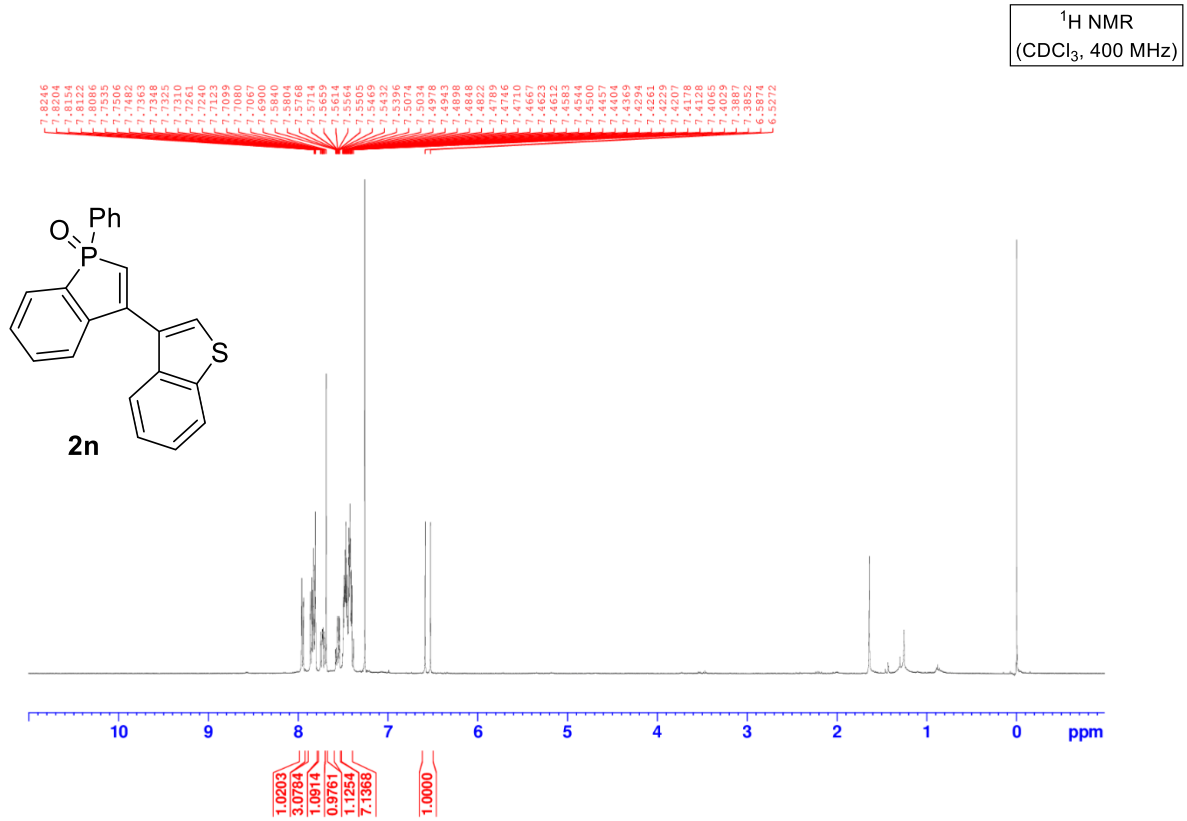


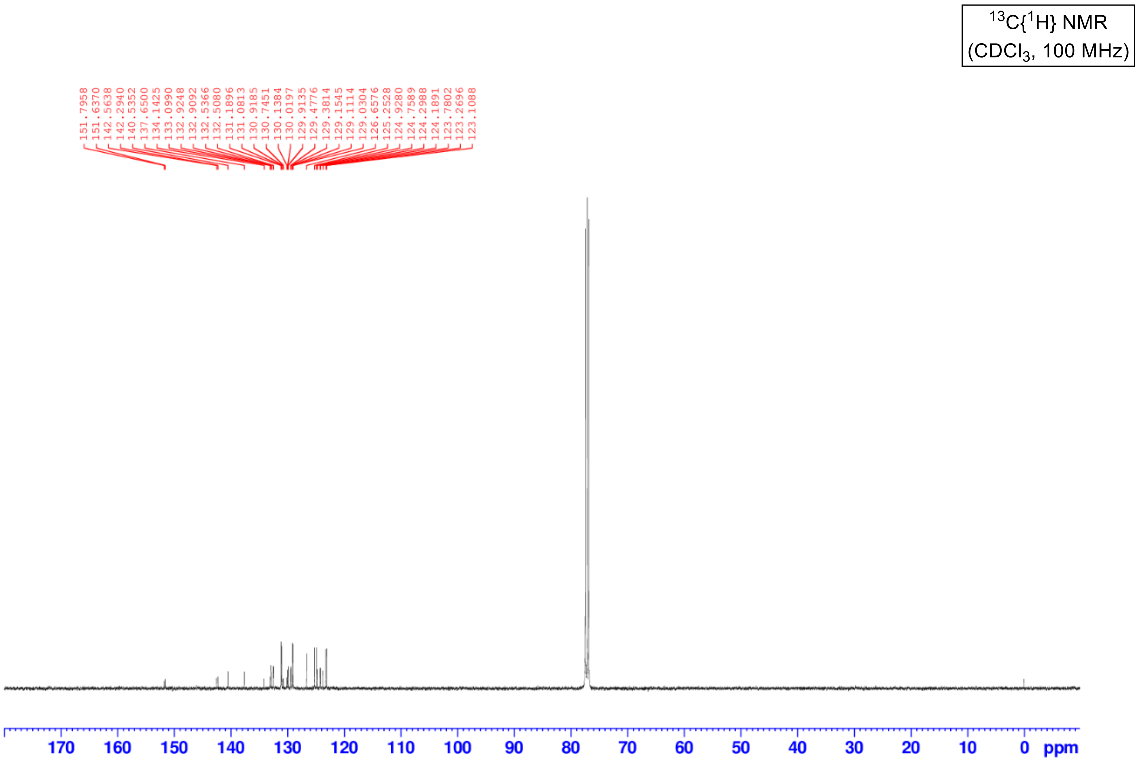

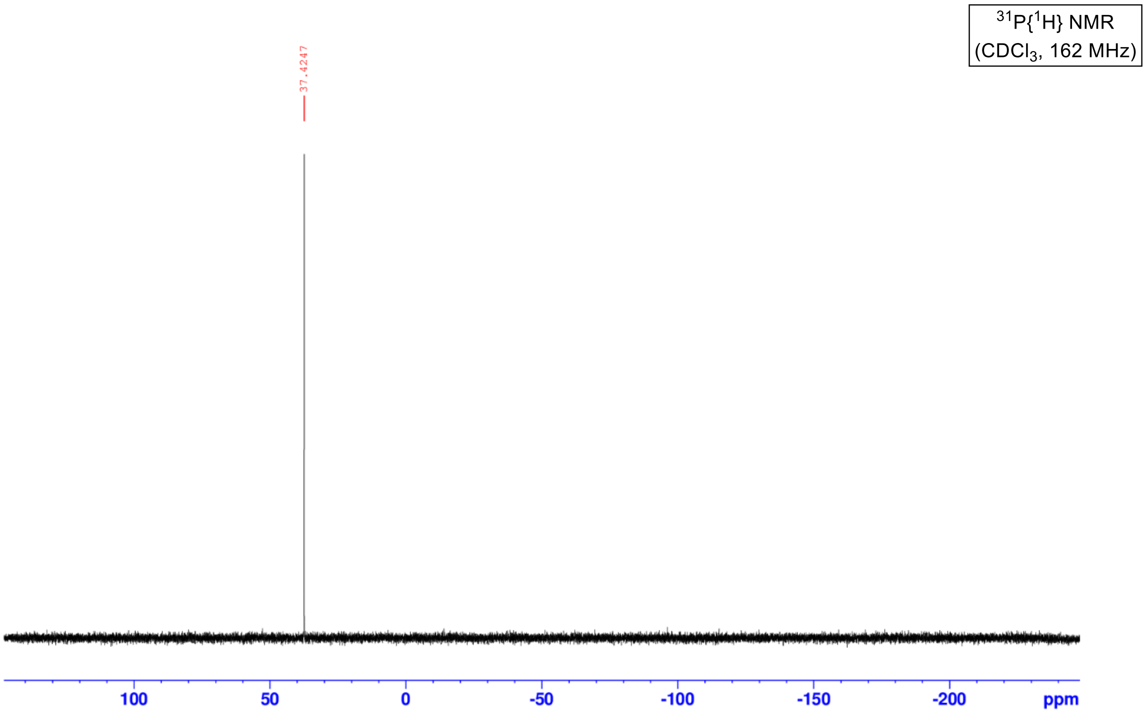

37.4247
$^{31}P\{^1H\}$ NMR
($CDCl_3$, 162 MHz)
100
50
0
-50
-100
-150
-200
ppm

[$^{1}$H and $^{31}$P{$^{1}$H} NMR Spectra of **2n'**]

$^{1}$H NMR
(CDCl$_3$, 400 MHz)

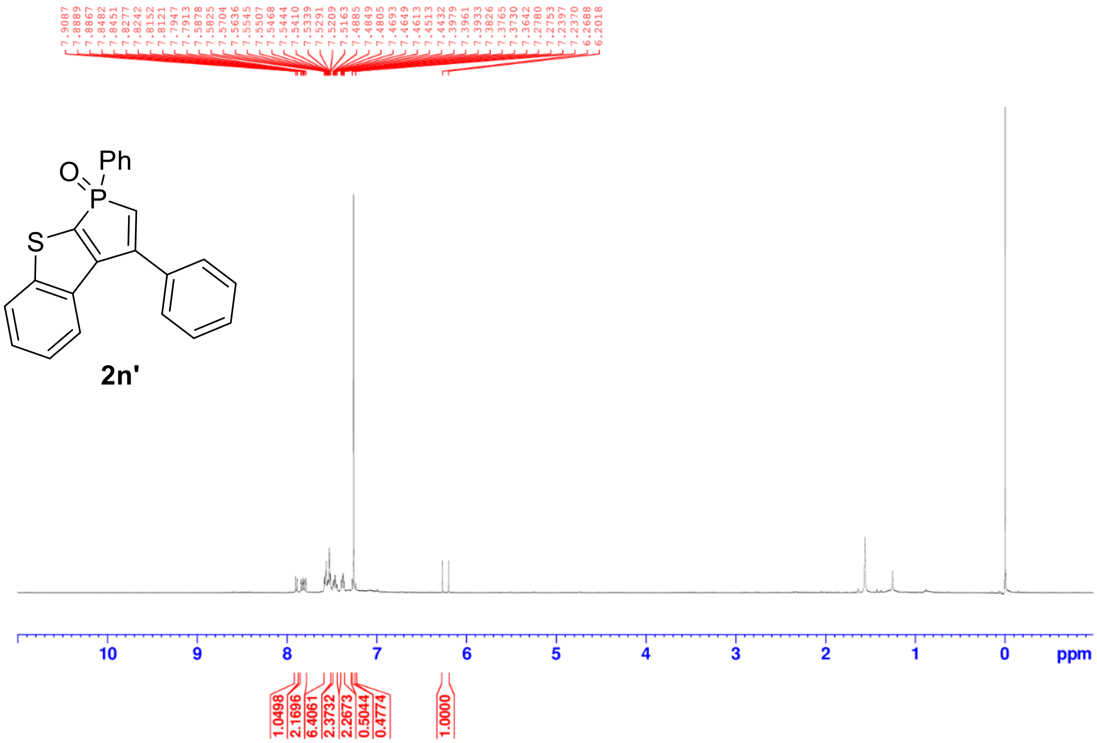


**2n'**

$^{31}$P{$^{1}$H} NMR
(CDCl$_3$, 162 MHz)

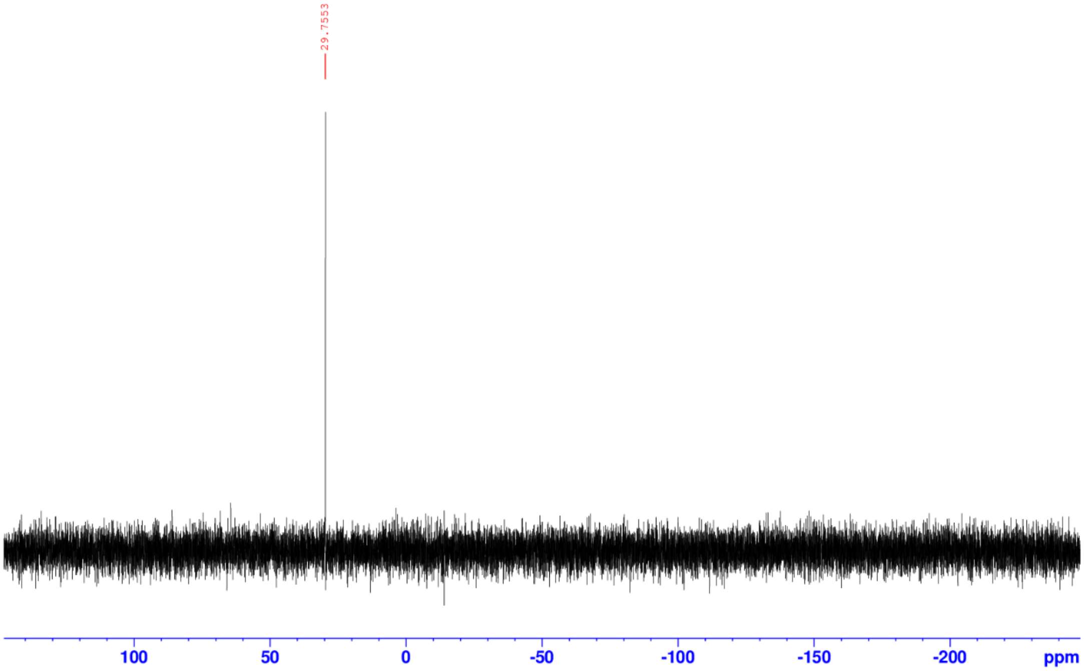

[$^{1}$H, $^{13}$C{$^{1}$H}, and $^{31}$P{$^{1}$H} NMR Spectra of **4aa**]

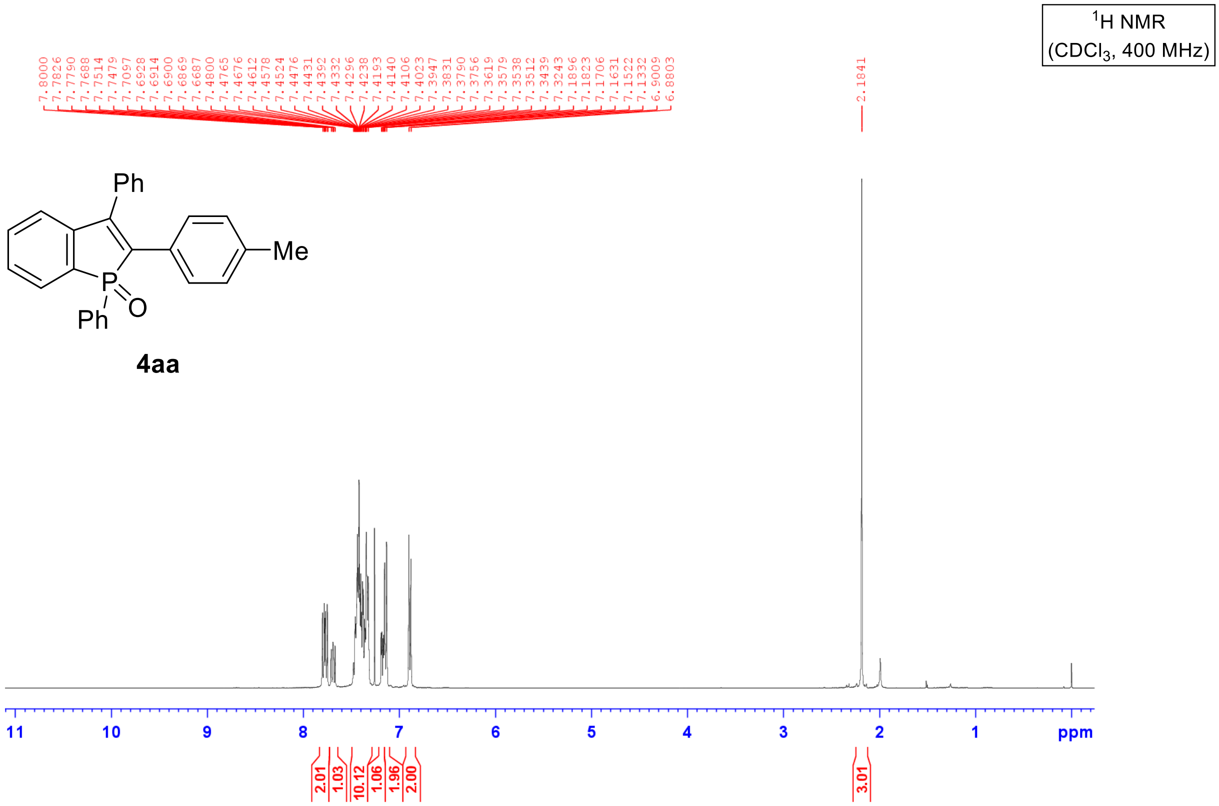


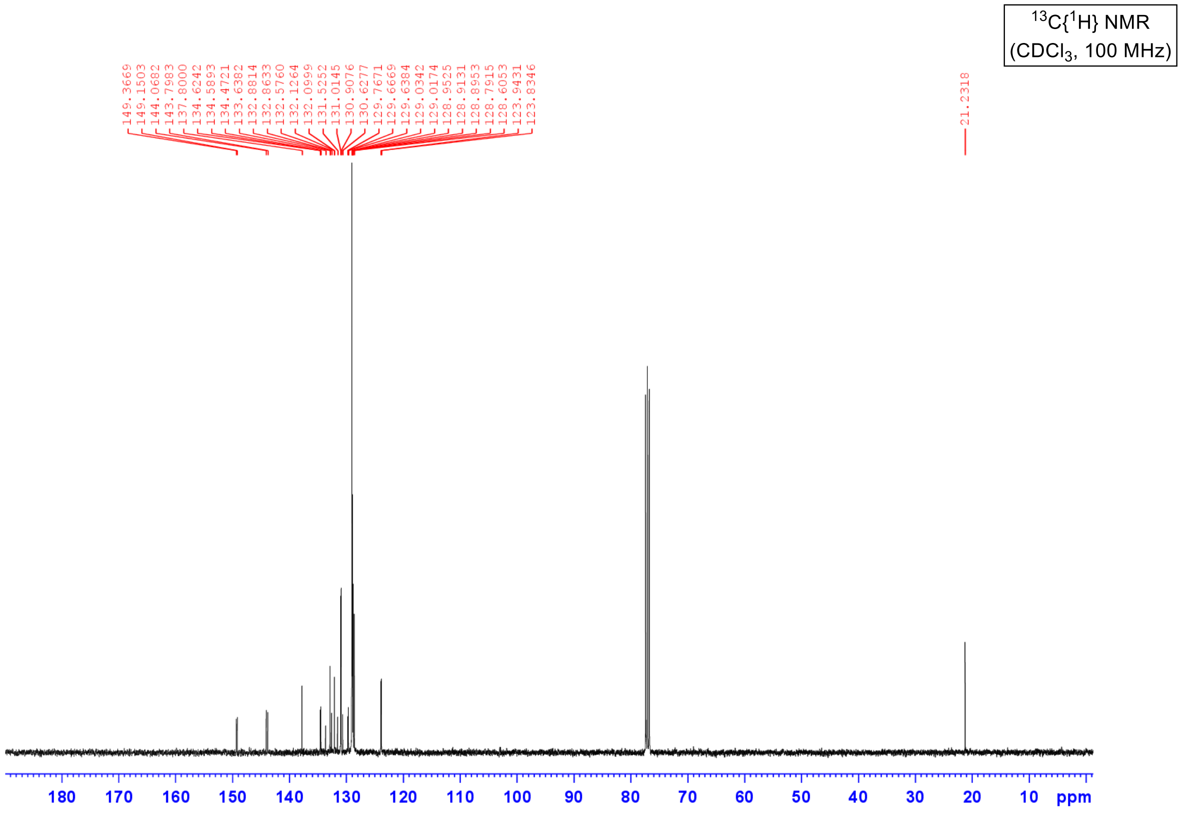

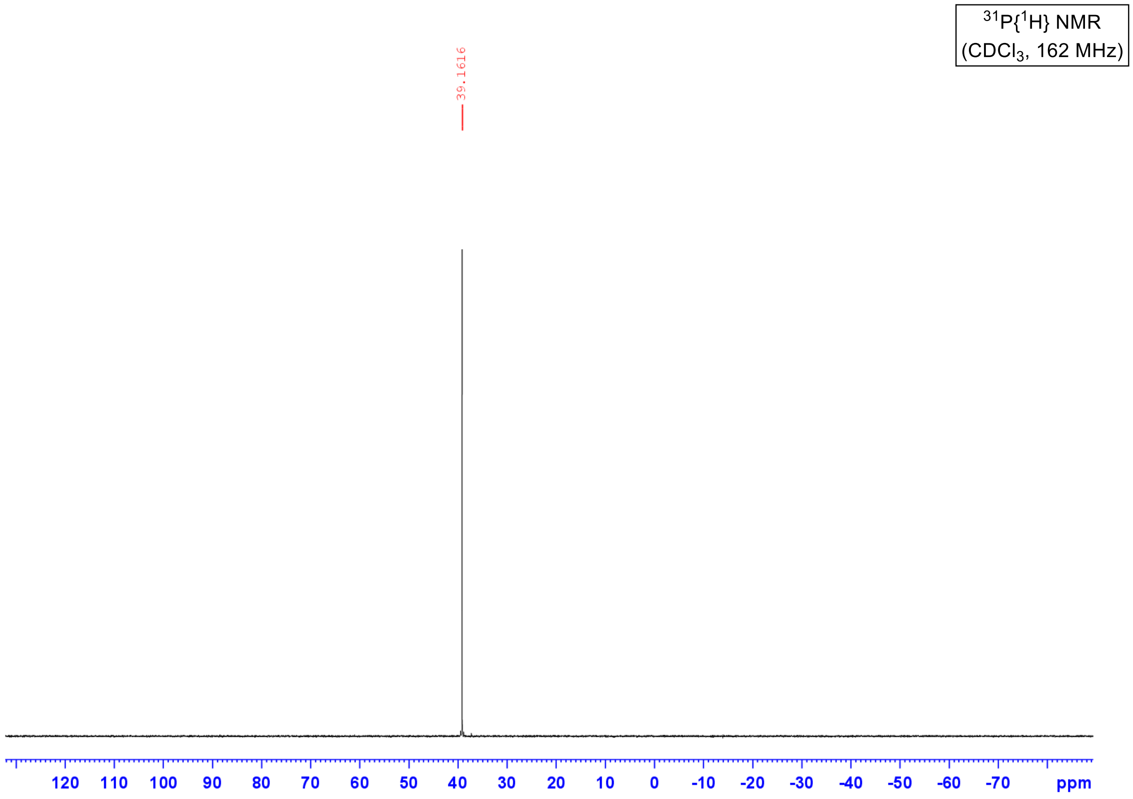

$^{31}P\{^1H\}$ NMR
($CDCl_3$, 162 MHz)
39.1616
120 110 100 90 80 70 60 50 40 30 20 10 0 -10 -20 -30 -40 -50 -60 -70 ppm

[$^{1}H$, $^{13}C\{^{1}H\}$, and $^{31}P\{^{1}H\}$ NMR Spectra of **4ab**]

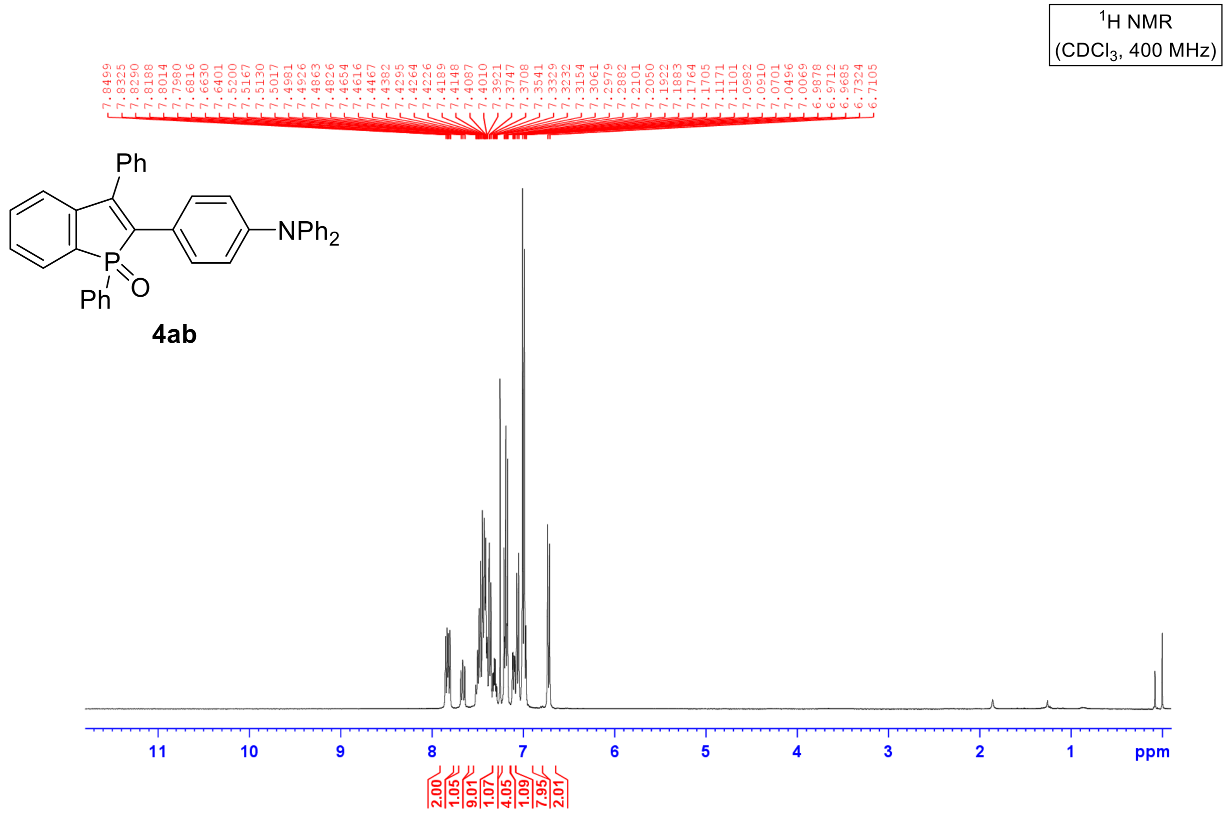


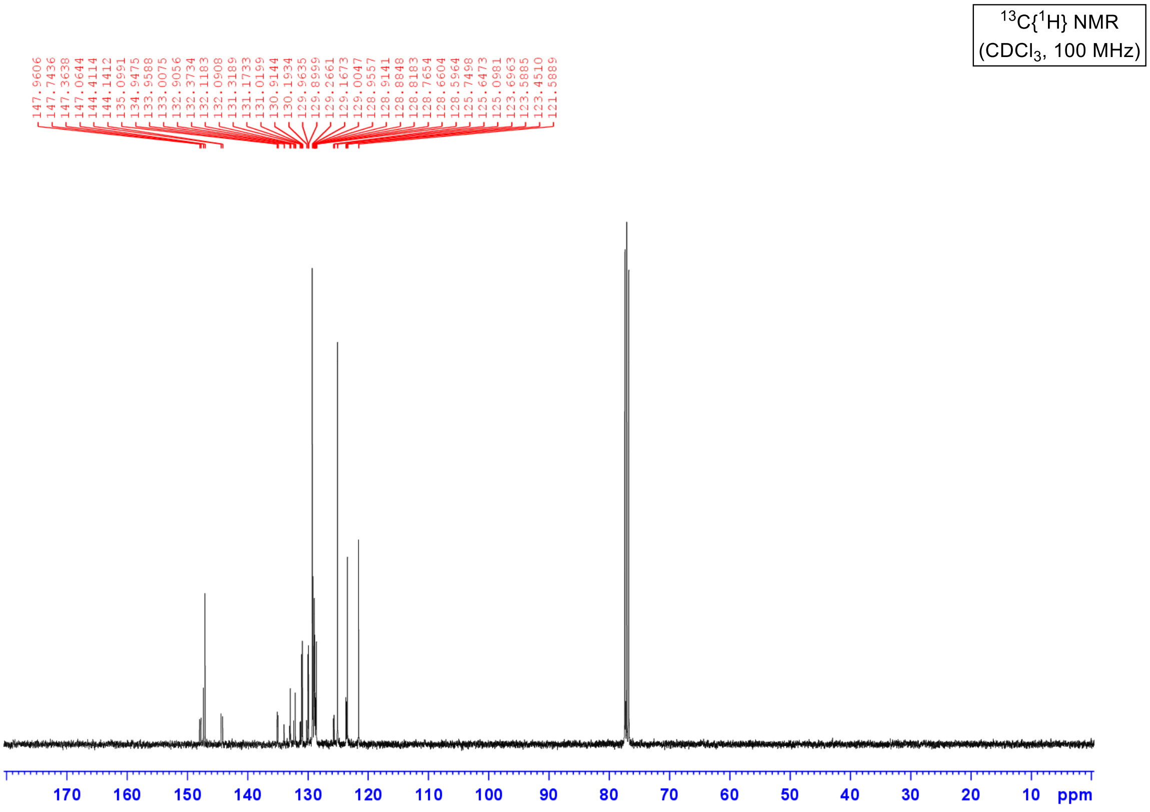

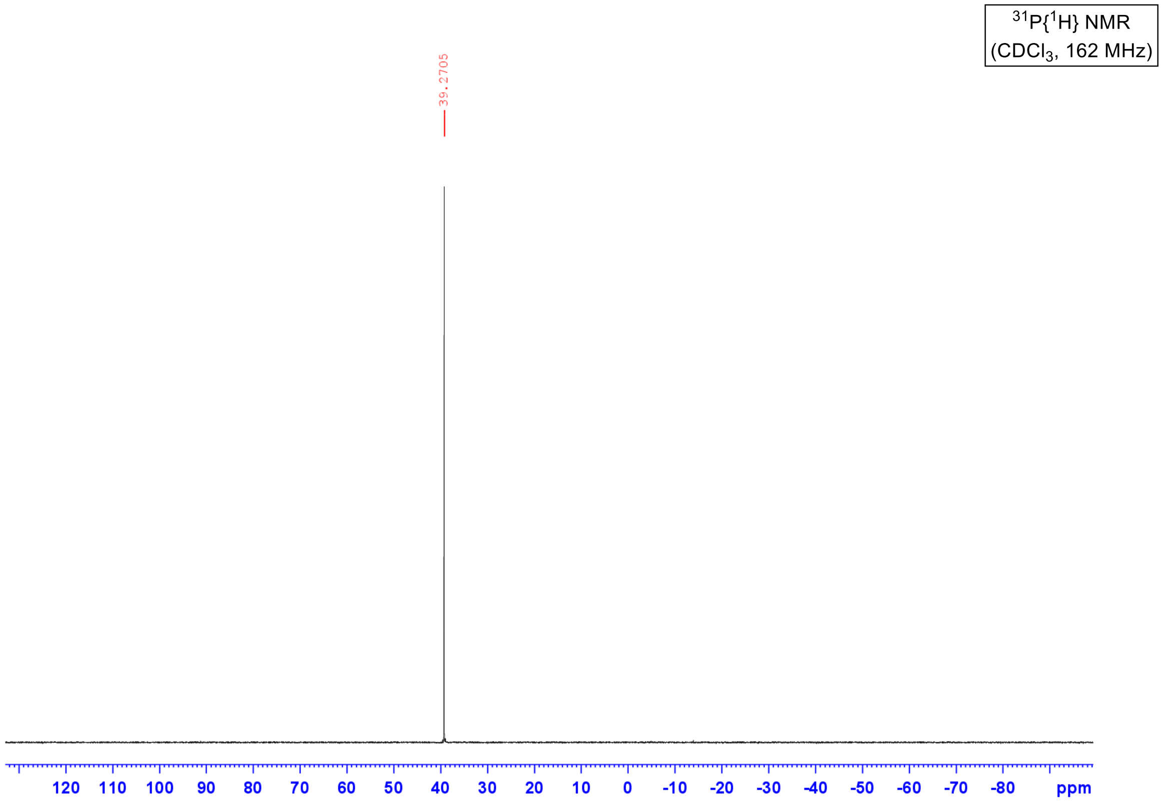

39.2705
$^{31}P\{^1H\}$ NMR
($CDCl_3$, 162 MHz)
120 110 100 90 80 70 60 50 40 30 20 10 0 -10 -20 -30 -40 -50 -60 -70 -80 ppm

[$^{1}$H, $^{13}$C{$^{1}$H}, and $^{31}$P{$^{1}$H} NMR Spectra of **4ac**]

$^{1}$H NMR
(CDCl$_3$, 400 MHz)

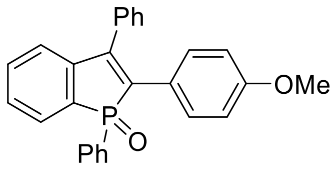

**4ac**

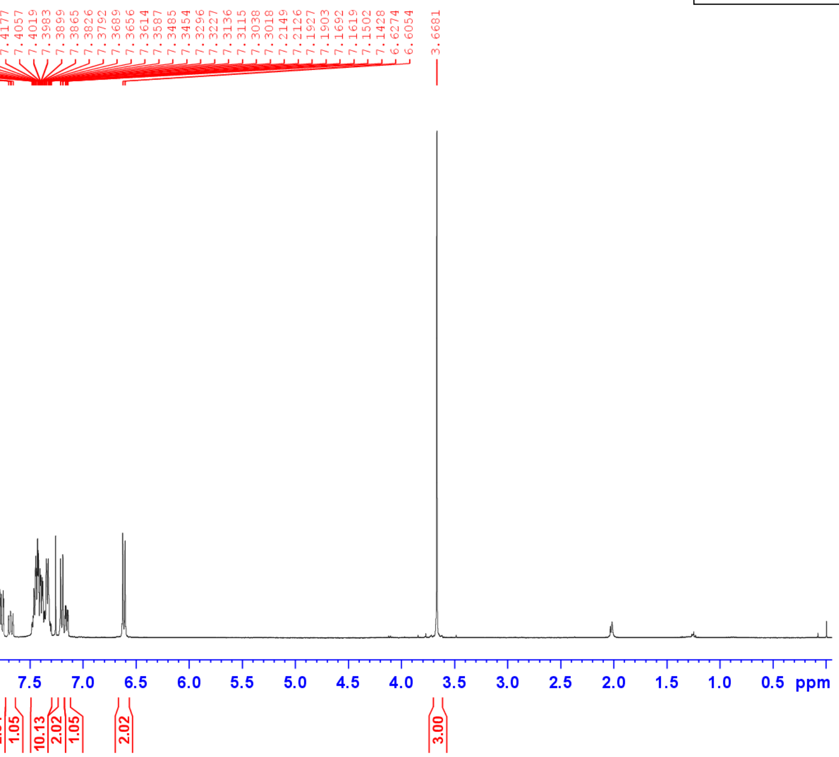


$^{13}$C{$^{1}$H} NMR
(CDCl$_3$, 100 MHz)

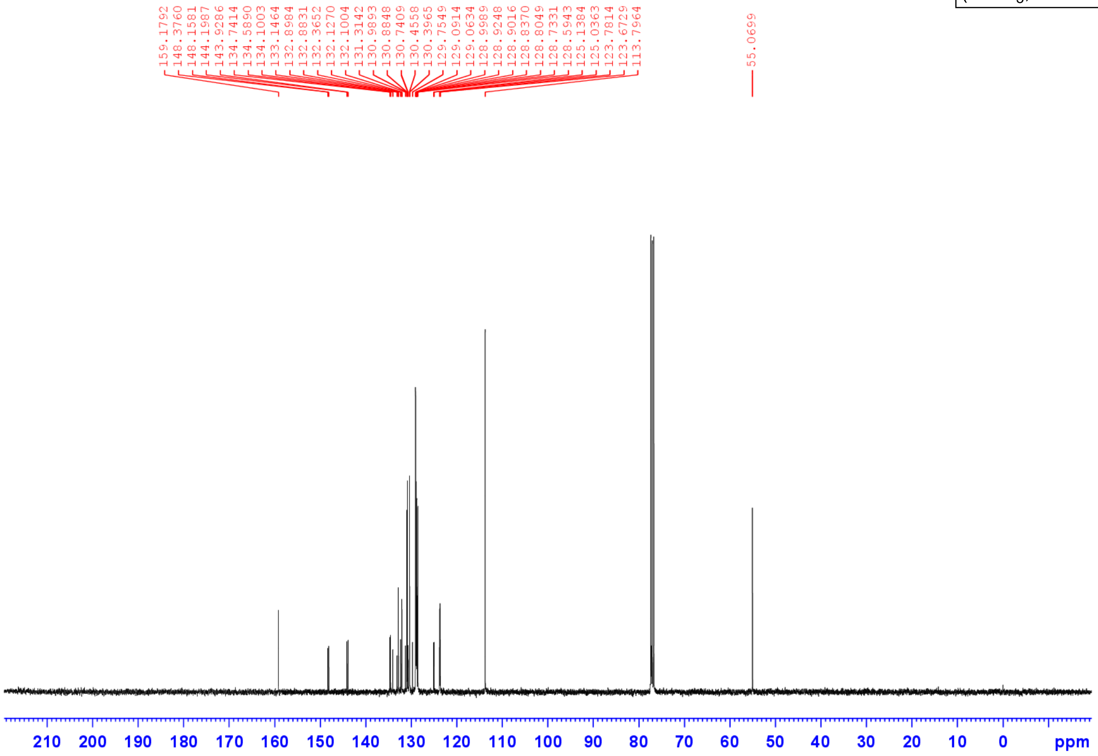

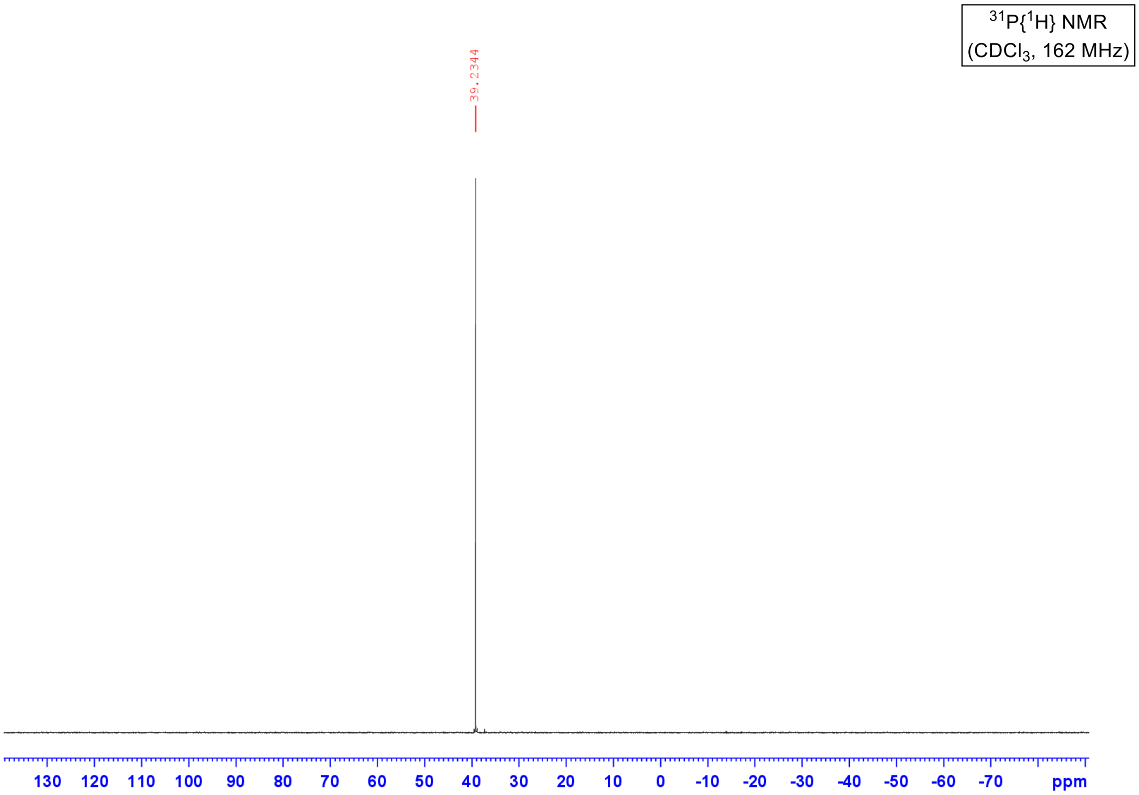
$^{31}P\{^1H\}$ NMR
($CDCl_3$, 162 MHz)
39.2344
130 120 110 100 90 80 70 60 50 40 30 20 10 0 -10 -20 -30 -40 -50 -60 -70 ppm

[$^{1}H$, $^{13}C\{^{1}H\}$, and $^{31}P\{^{1}H\}$ NMR Spectra of **4ad**]

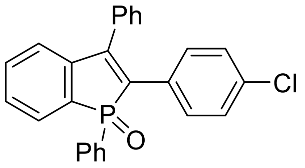


**4ad**

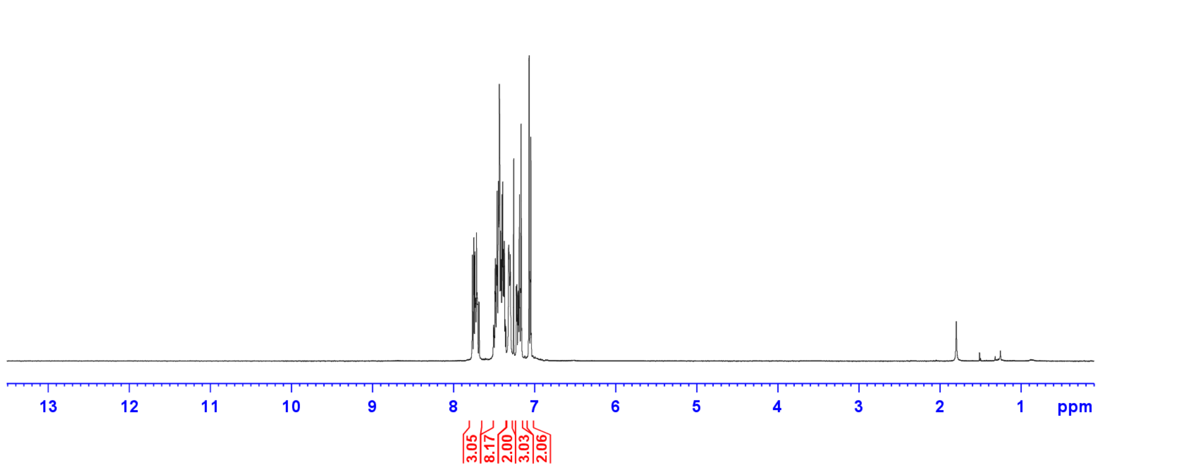


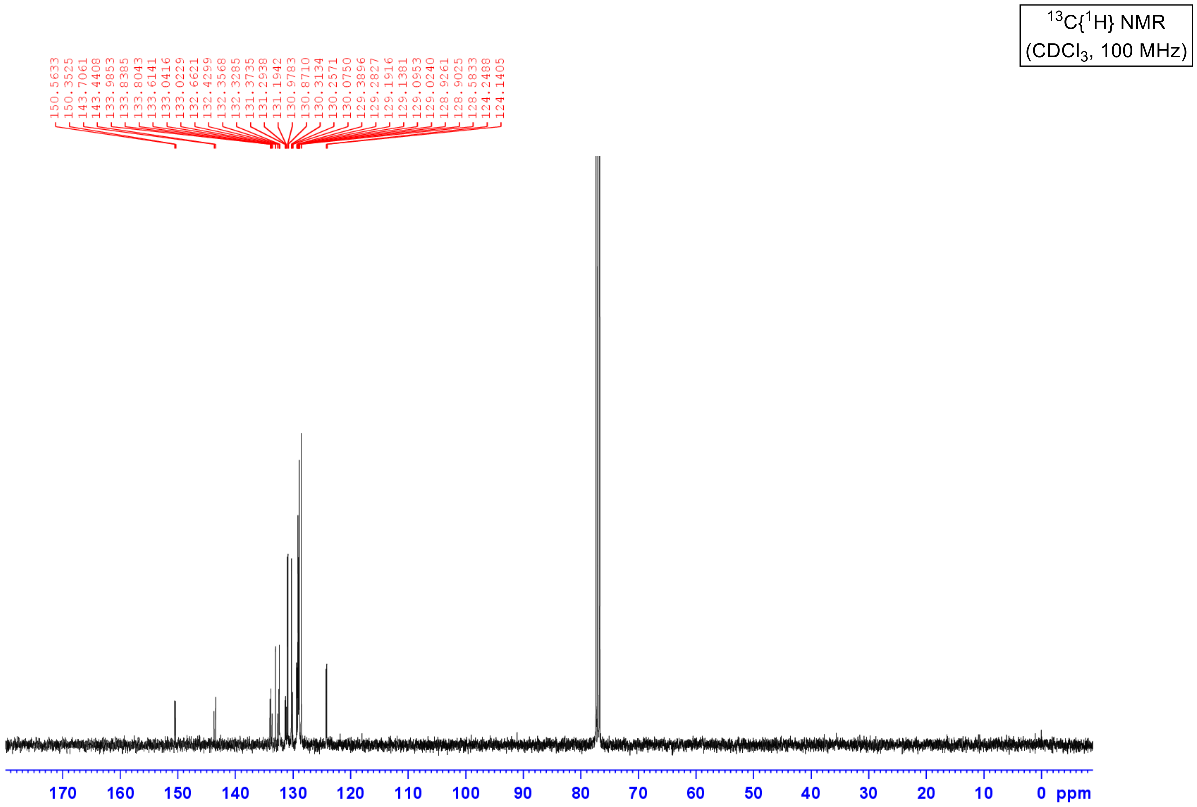

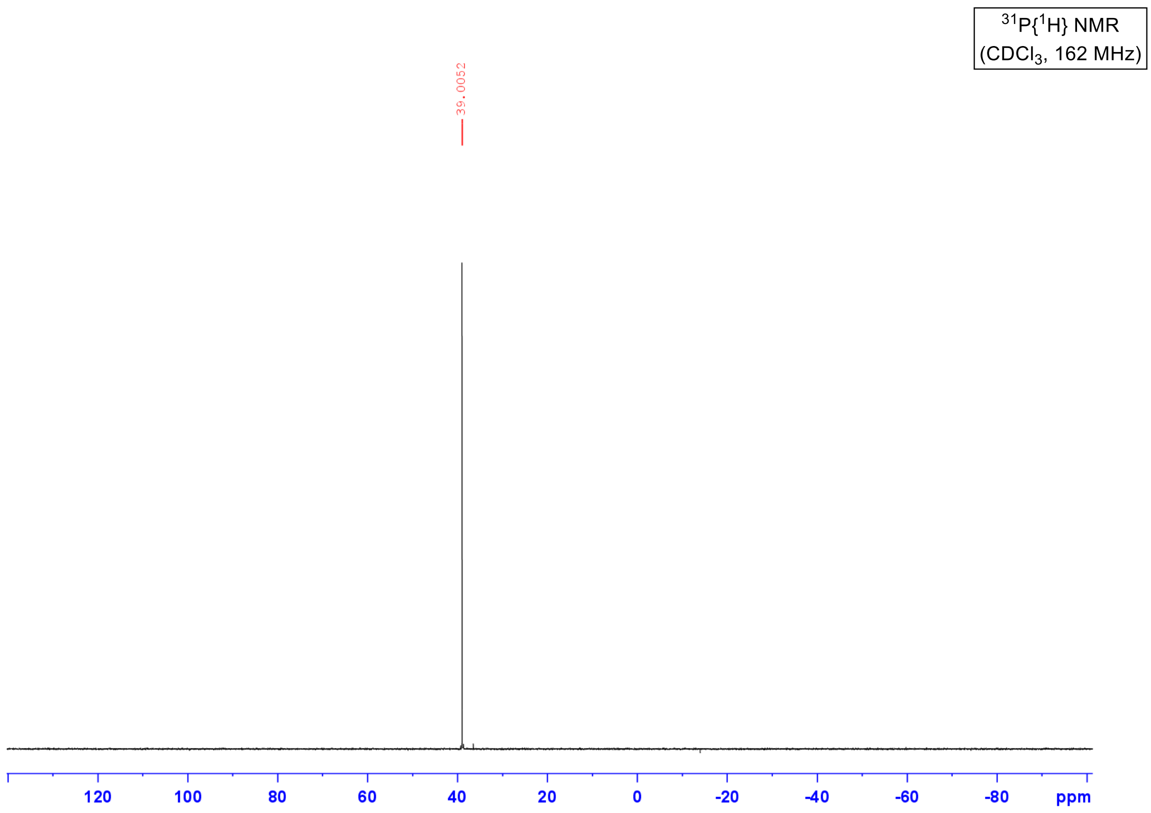
$^{31}P\{^1H\}$ NMR
($CDCl_3$, 162 MHz)
39.0052
120
100
80
60
40
20
0
-20
-40
-60
-80
ppm

[$^{1}$H, $^{13}$C{$^{1}$H}, $^{19}$F{$^{1}$H}, and $^{31}$P{$^{1}$H} NMR Spectra of **4ae**]

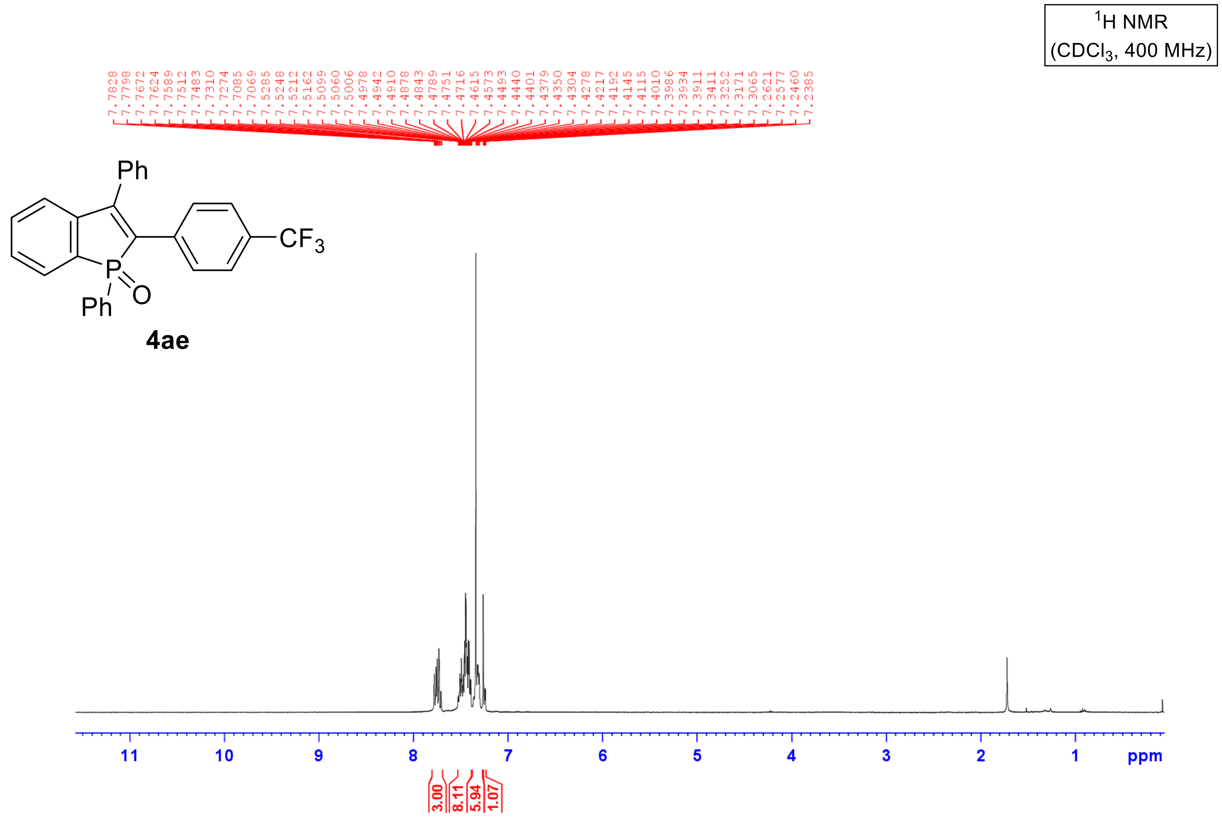


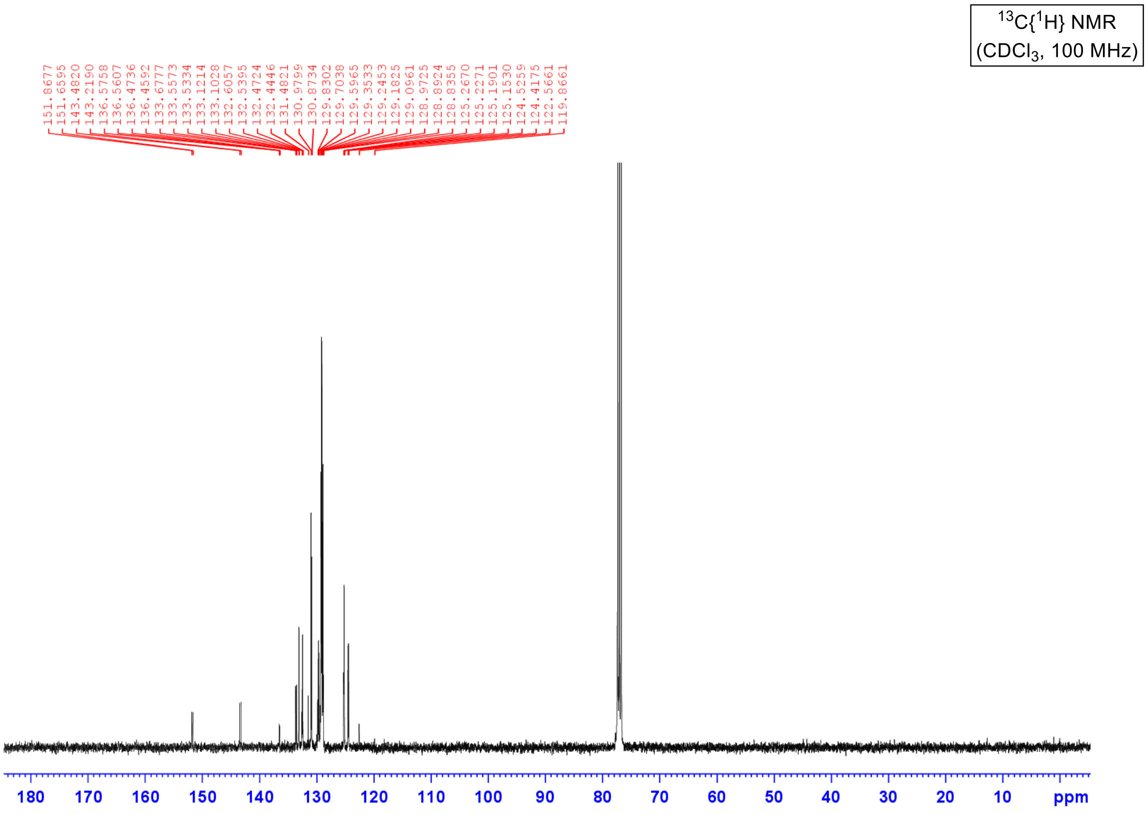

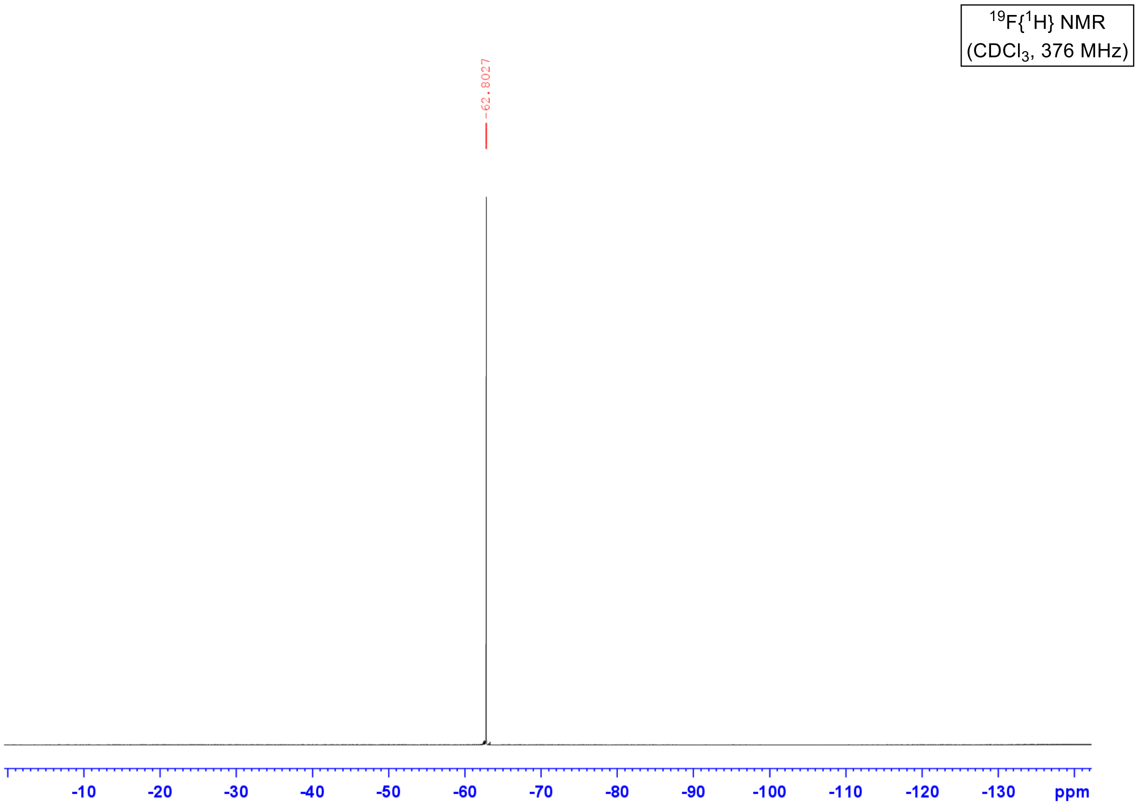

$^{19}F\{^{1}H\}$ NMR
($CDCl_3$, 376 MHz)
-62.8027
-10
-20
-30
-40
-50
-60
-70
-80
-90
-100
-110
-120
-130
ppm


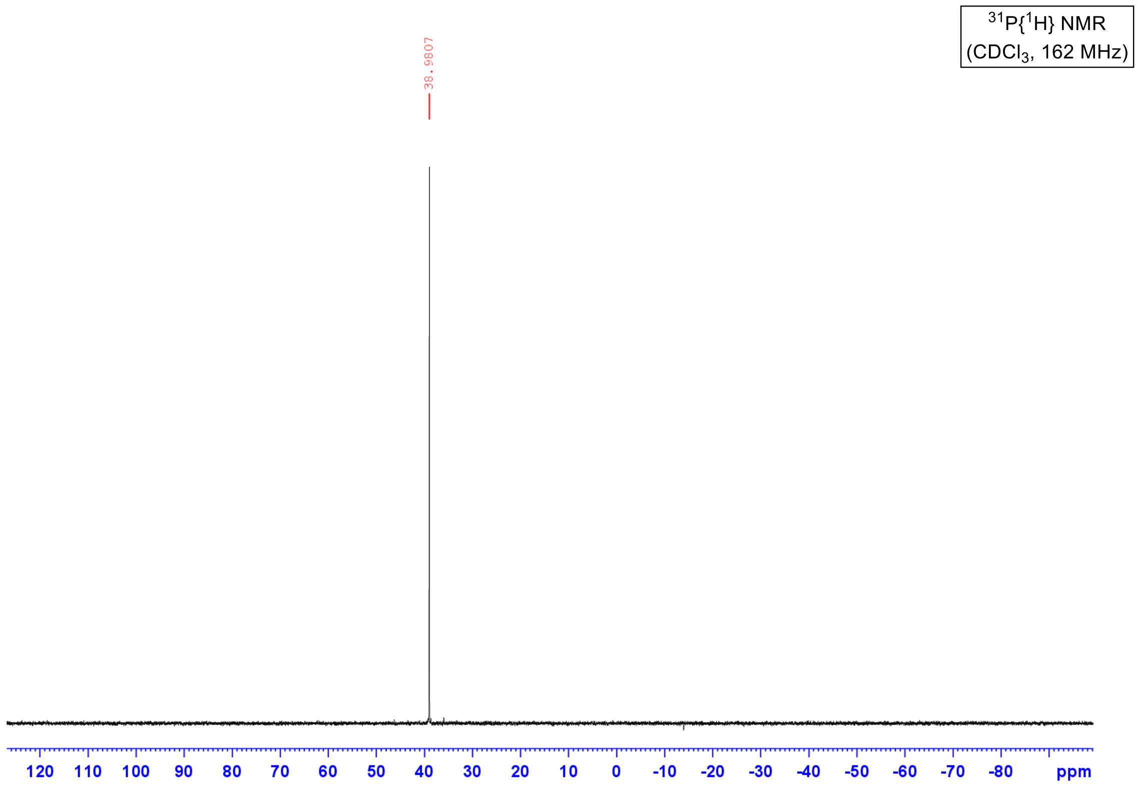

$^{31}P\{^{1}H\}$ NMR
($CDCl_3$, 162 MHz)
38.9807
120
110
100
90
80
70
60
50
40
30
20
10
0
-10
-20
-30
-40
-50
-60
-70
-80
ppm

[$^{1}$H, $^{13}$C{$^{1}$H}, and $^{31}$P{$^{1}$H} NMR Spectra of **4af**]

$^{1}$H NMR
(CDCl$_3$, 400 MHz)

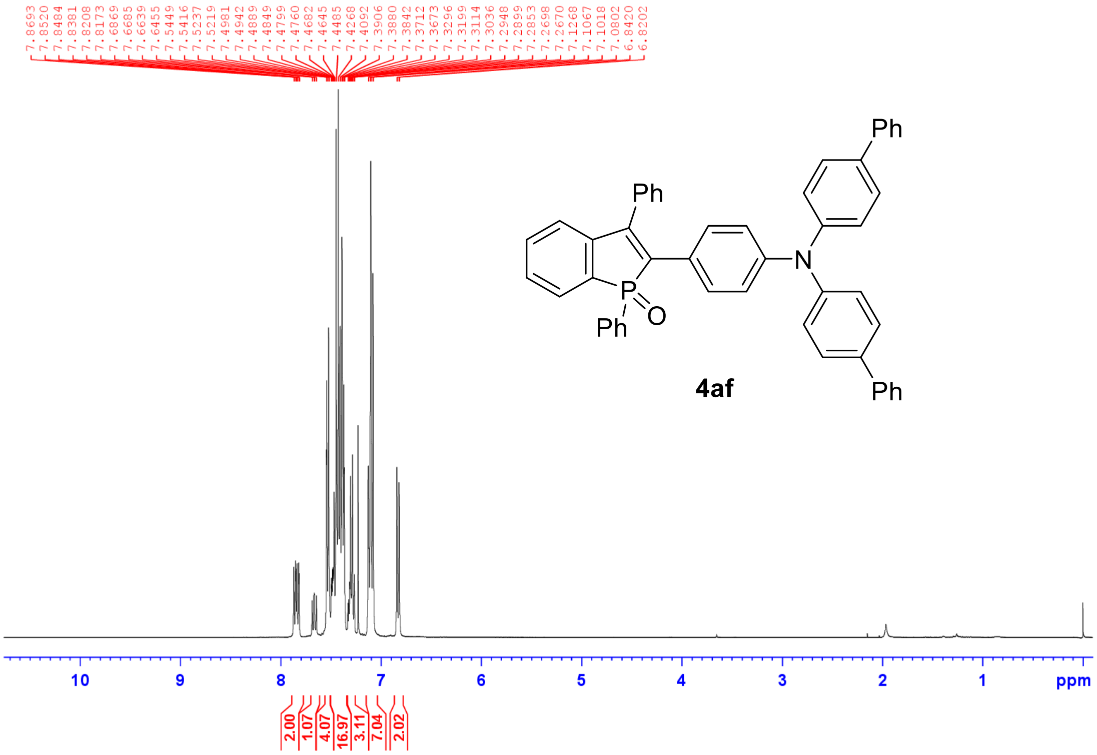


$^{13}$C{$^{1}$H} NMR
(CDCl$_3$, 100 MHz)

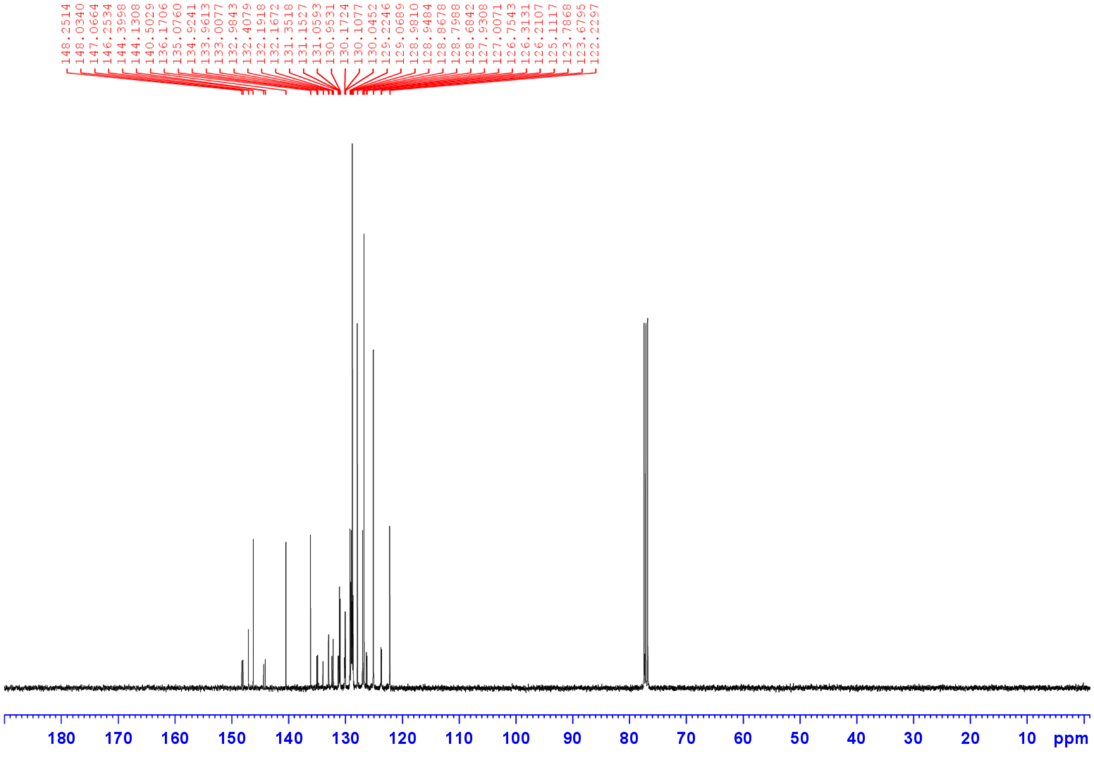

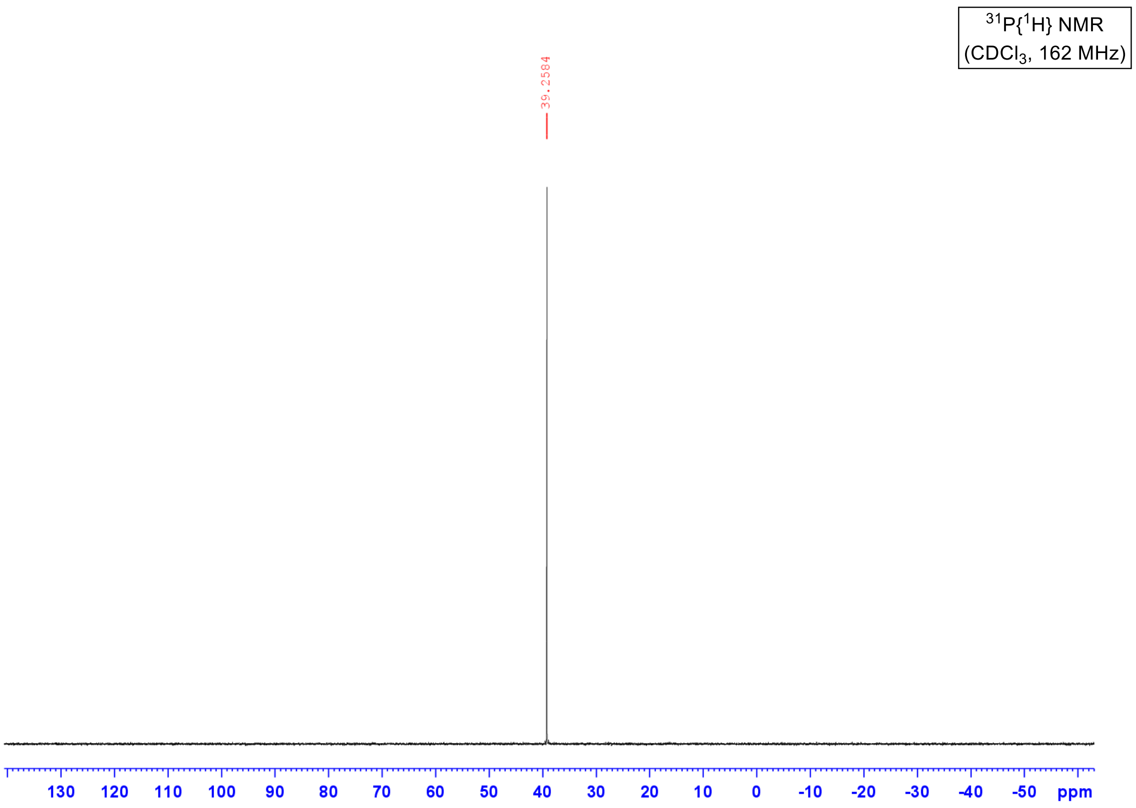

$^{31}P\{^1H\}$ NMR
($CDCl_3$, 162 MHz)
39.2584
130
120
110
100
90
80
70
60
50
40
30
20
10
0
-10
-20
-30
-40
-50
ppm

[$^{1}H$, $^{13}C\{^{1}H\}$, and $^{31}P\{^{1}H\}$ NMR Spectra of **4ag**]

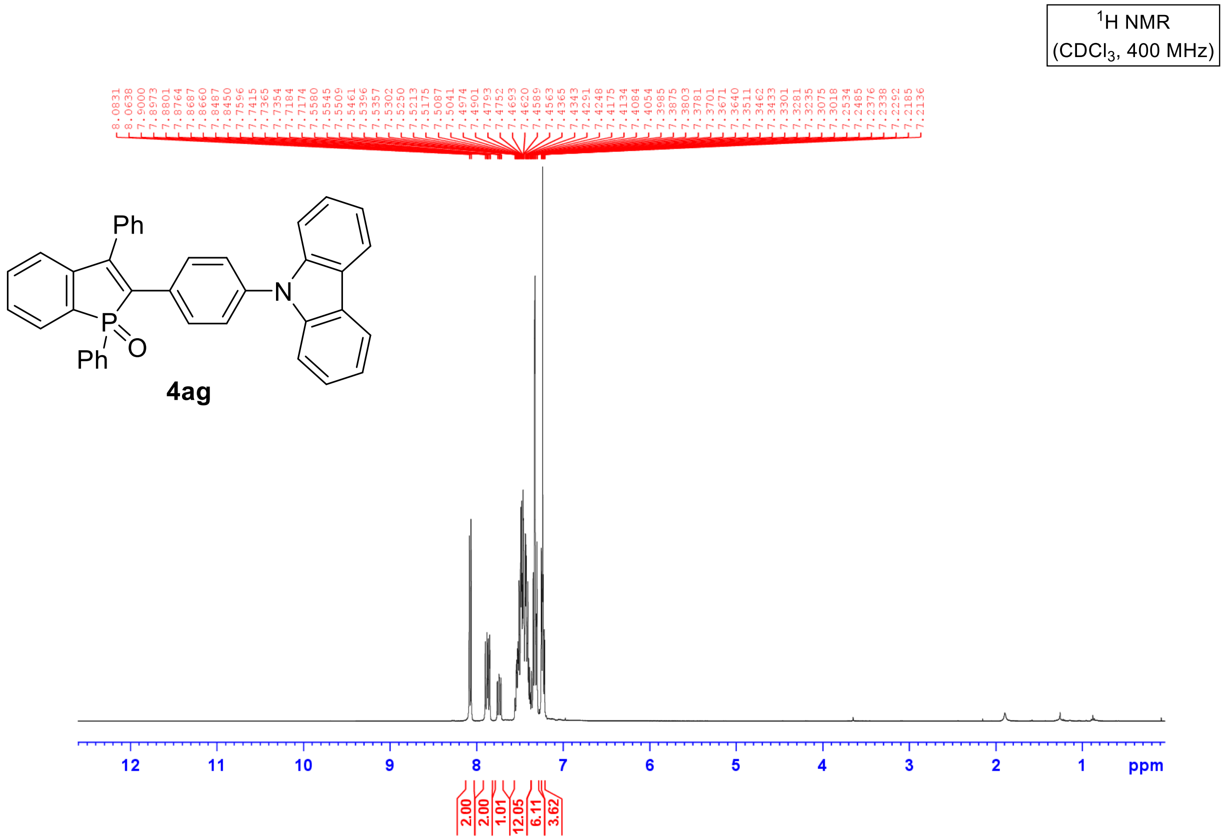


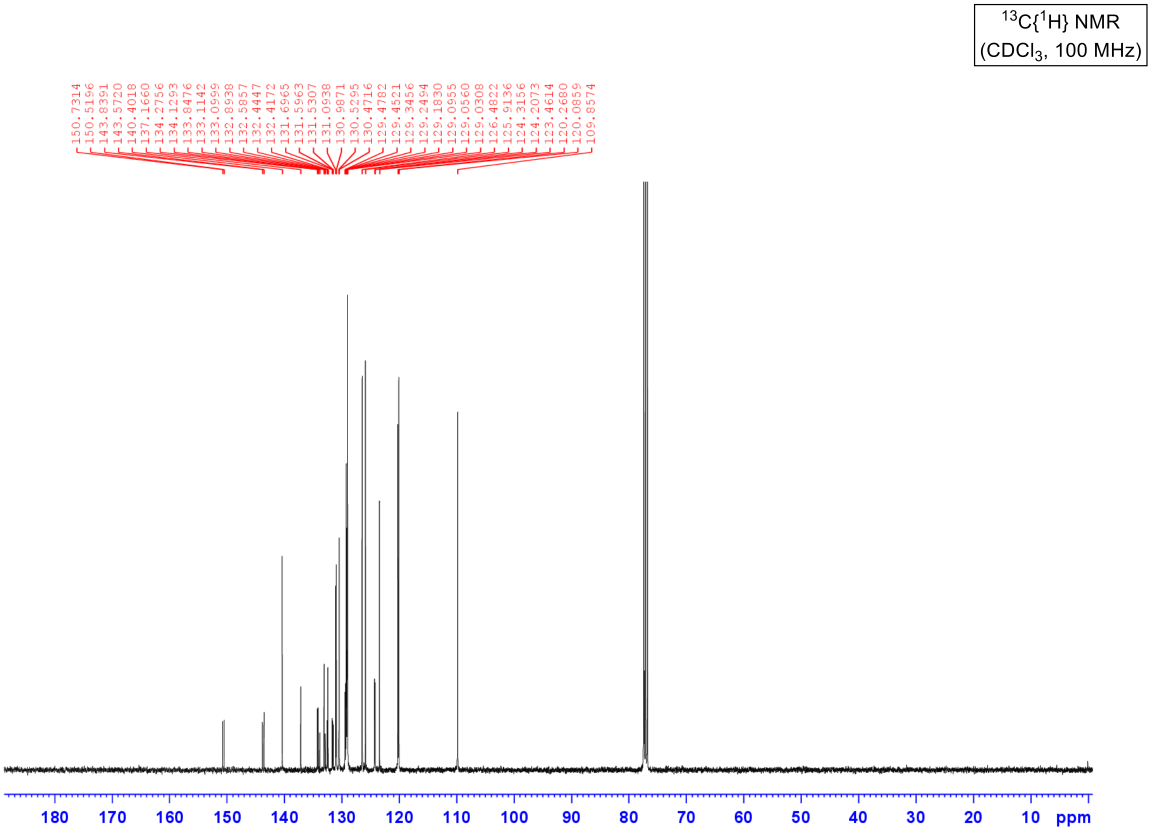

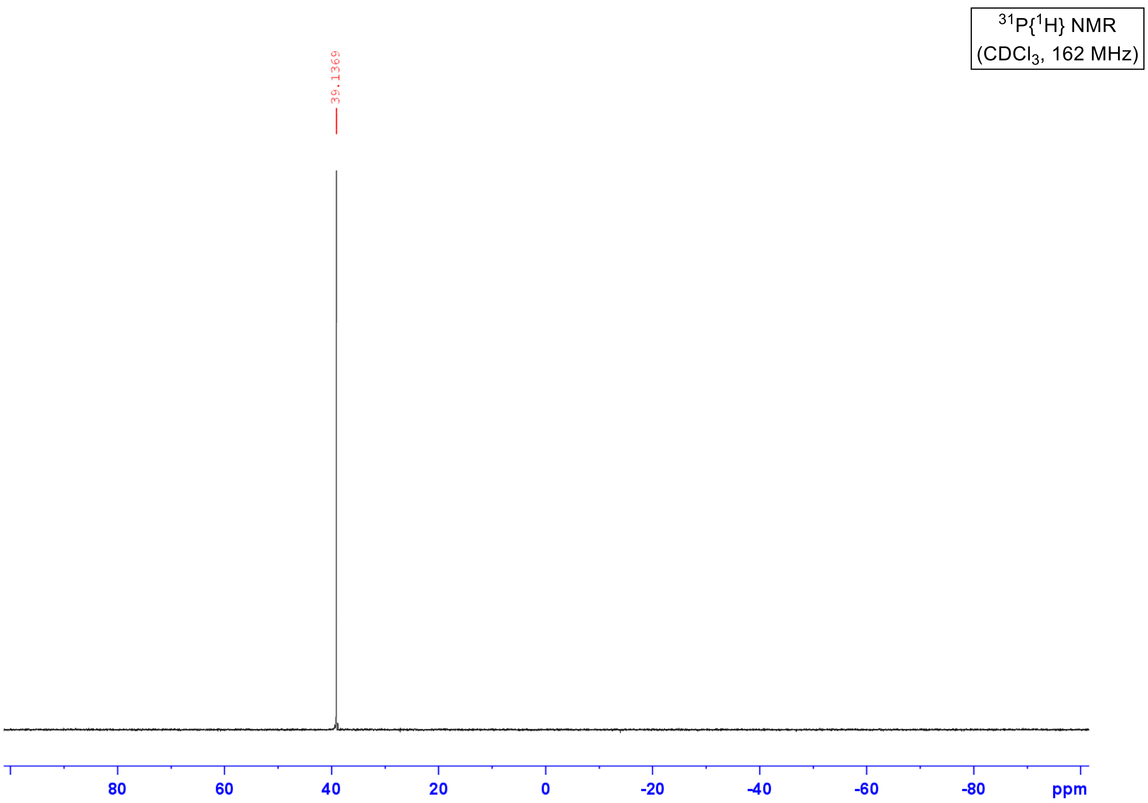
39.1369
$^{31}P\{^1H\}$ NMR
($CDCl_3$, 162 MHz)
80
60
40
20
0
-20
-40
-60
-80
ppm

[$^{1}H$, $^{13}C\{^{1}H\}$, and $^{31}P\{^{1}H\}$ NMR Spectra of **4ah**]

$^{1}H$ NMR
($CDCl_3$, 400 MHz)

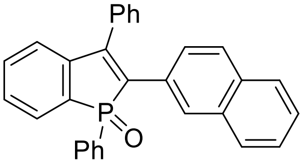

**4ah**

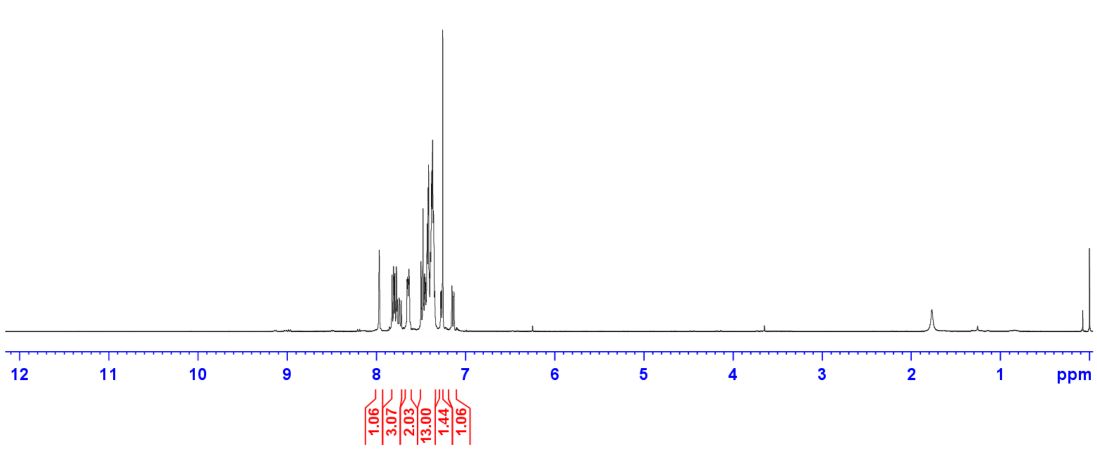

$^{13}C\{^{1}H\}$ NMR
($CDCl_3$, 100 MHz)

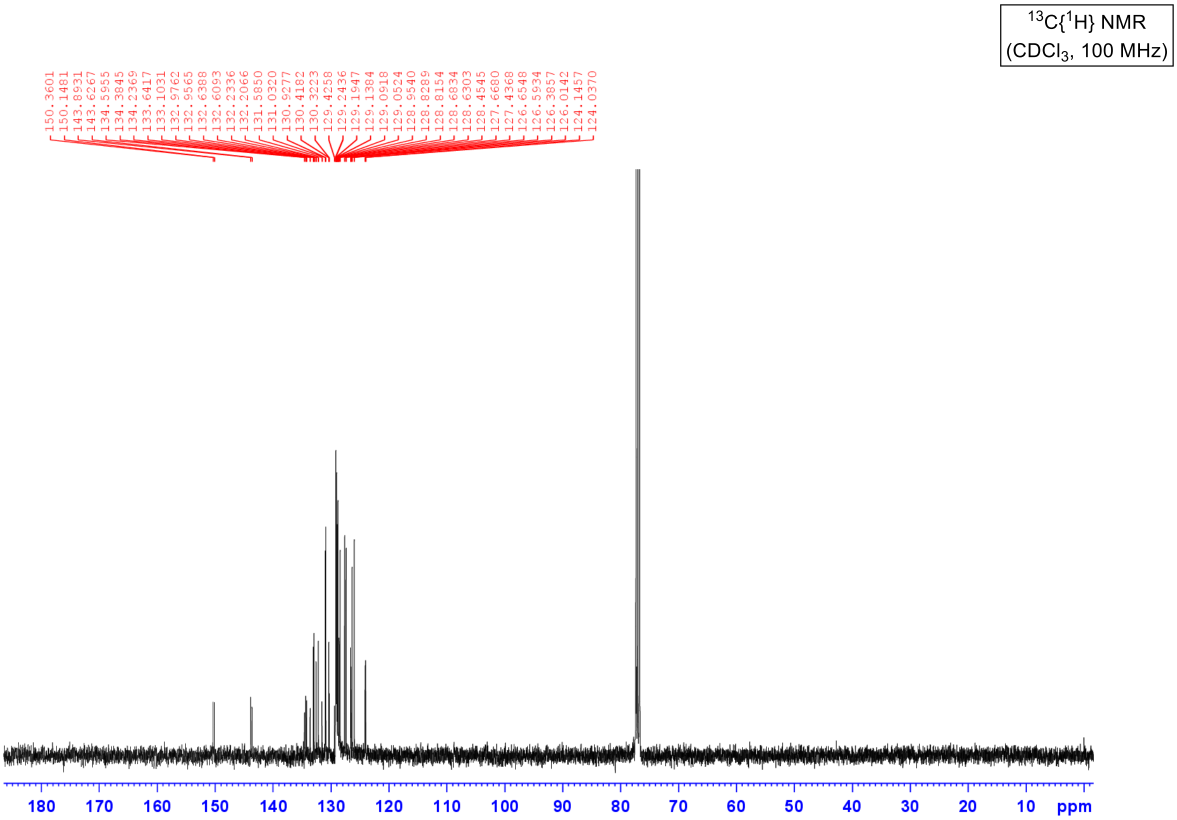

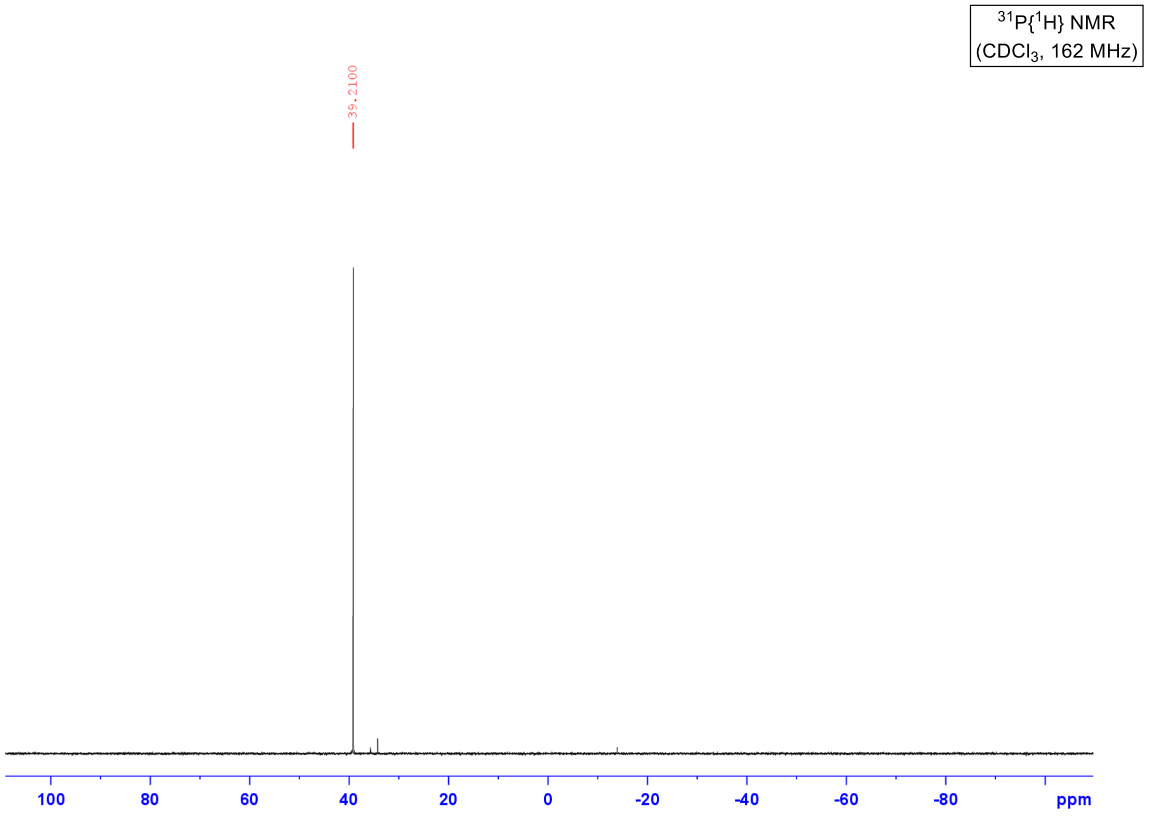

$^{31}P\{^1H\}$ NMR
($CDCl_3$, 162 MHz)
39.2100
100
80
60
40
20
0
-20
-40
-60
-80
ppm

[$^{1}H$, $^{13}C\{^{1}H\}$, and $^{31}P\{^{1}H\}$ NMR Spectra of **4ai**]

$^{1}H$ NMR
($CDCl_3$, 400 MHz)

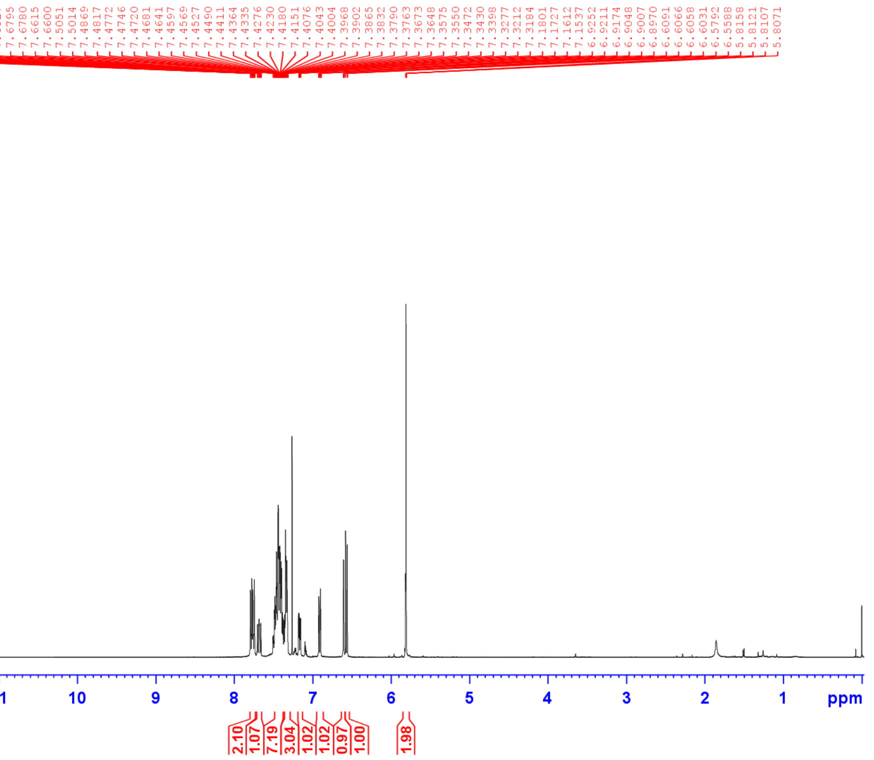


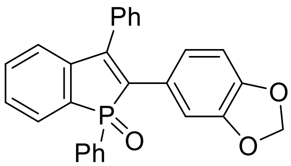


**4ai**

$^{13}C\{^{1}H\}$ NMR
($CDCl_3$, 100 MHz)

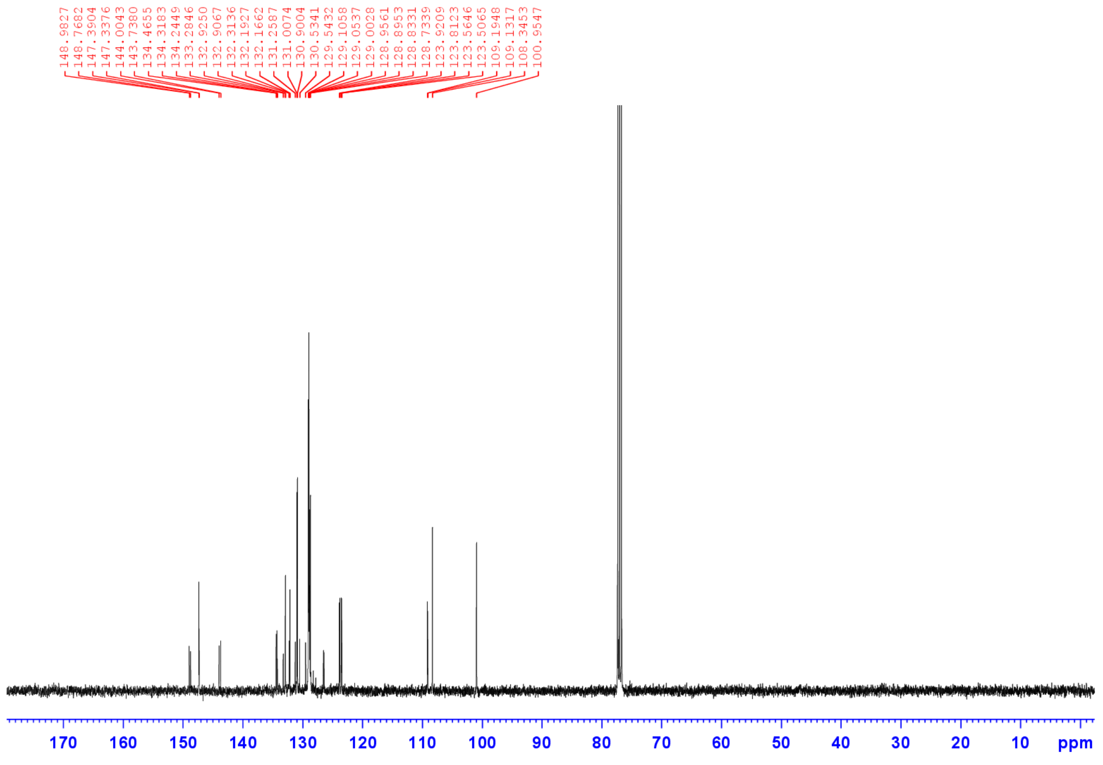

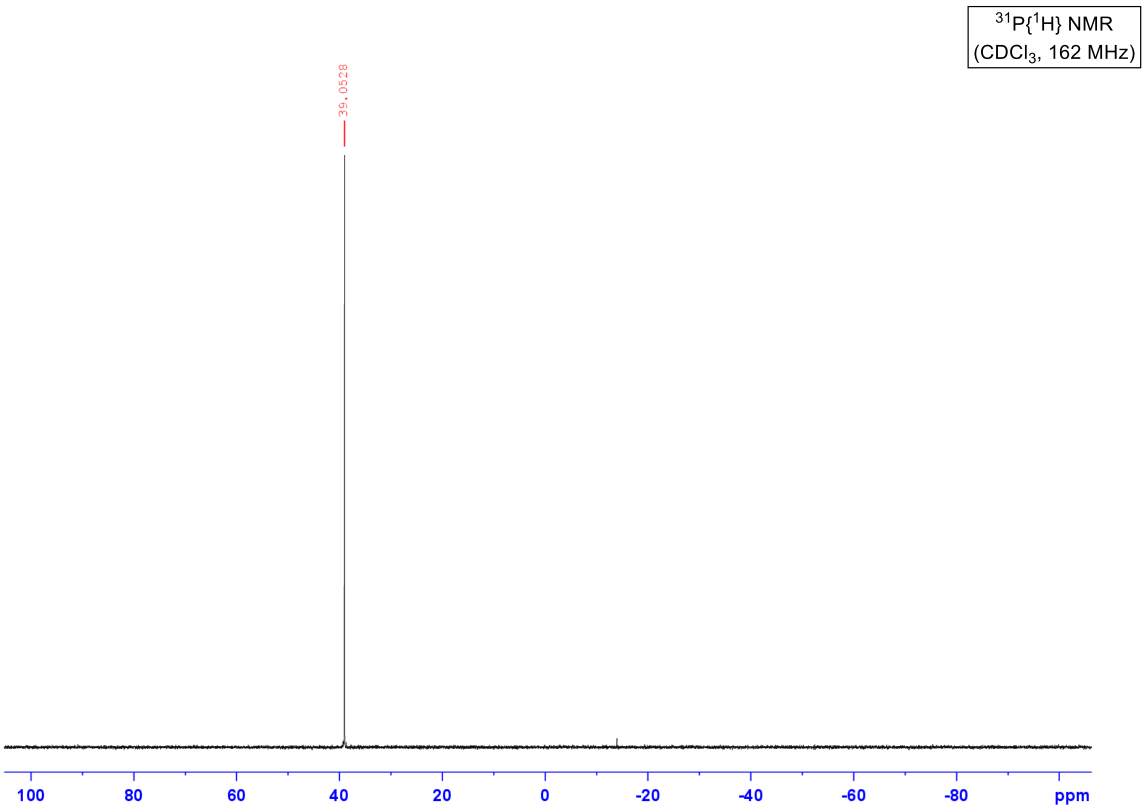
$^{31}P\{^1H\}$ NMR
($CDCl_3$, 162 MHz)
39.0528
100
80
60
40
20
0
-20
-40
-60
-80
ppm

[$^1$H, $^{13}$C{$^1$H}, and $^{31}$P{$^1$H} NMR Spectra of **4aj**]

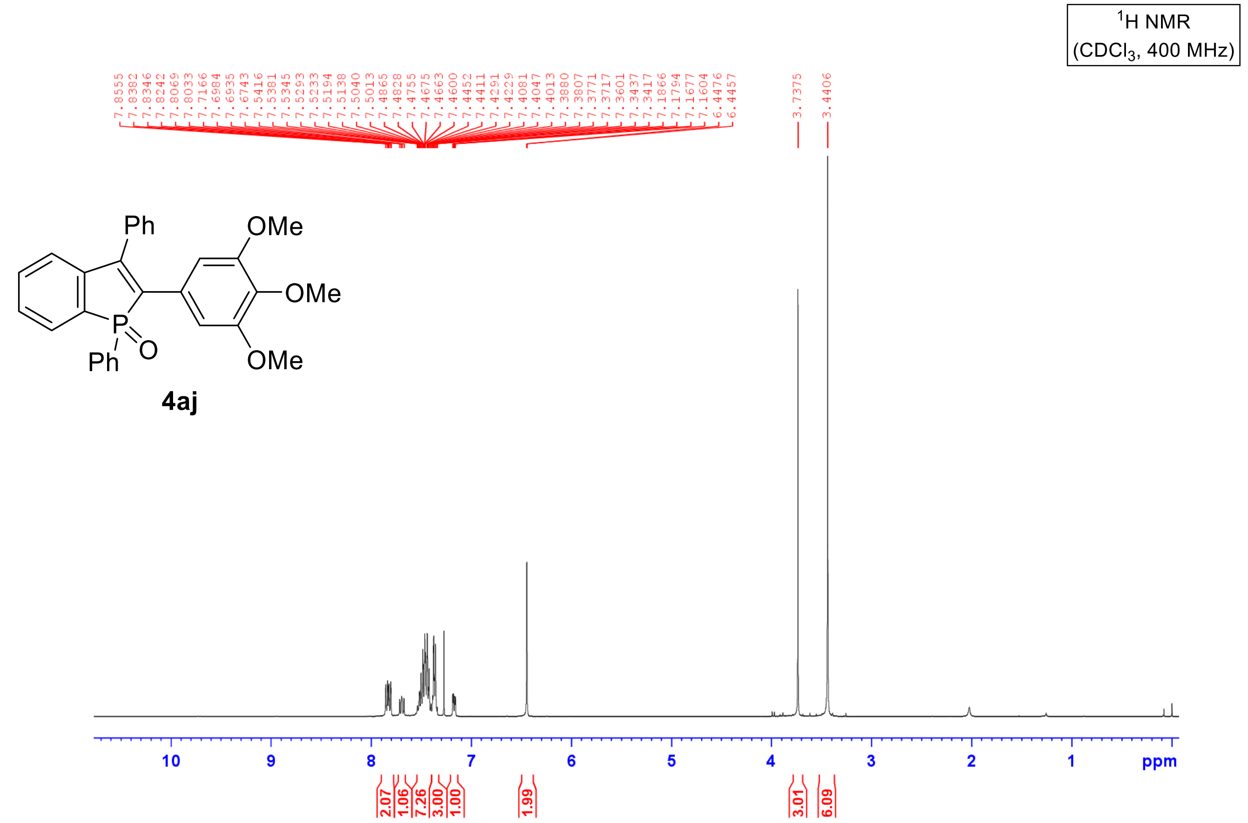


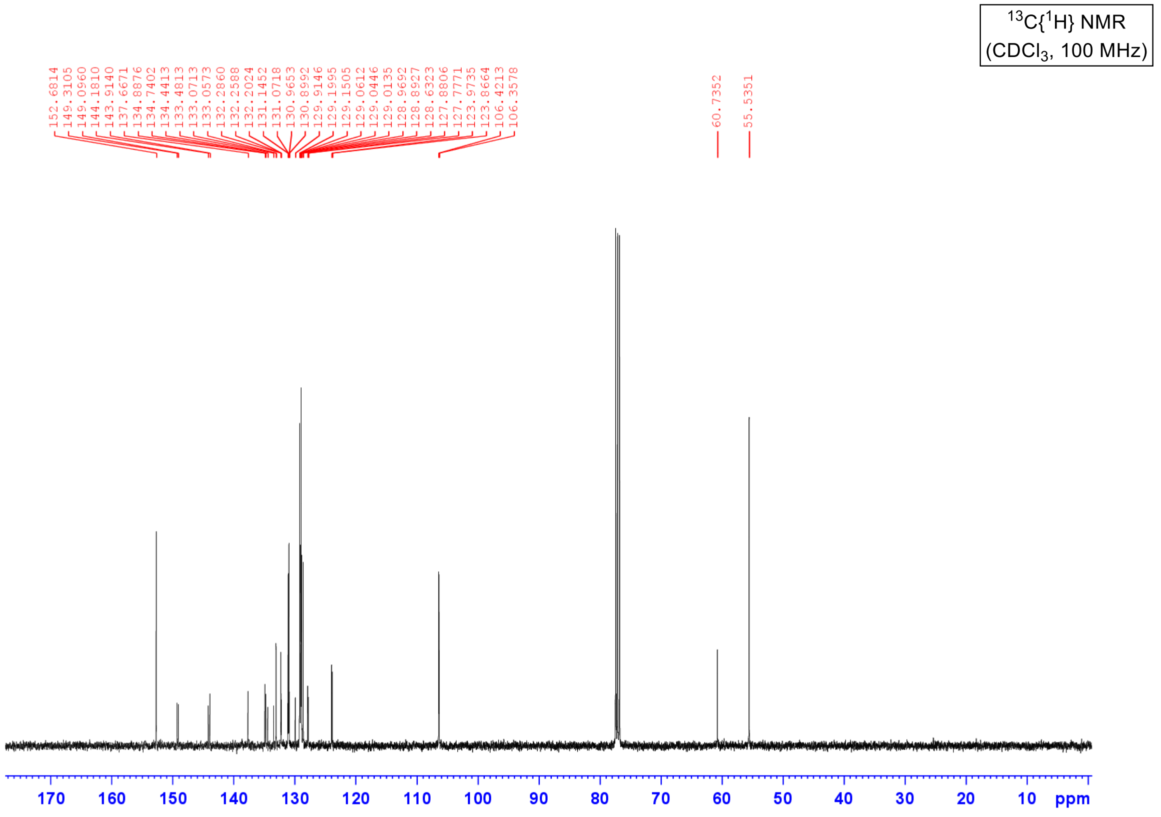

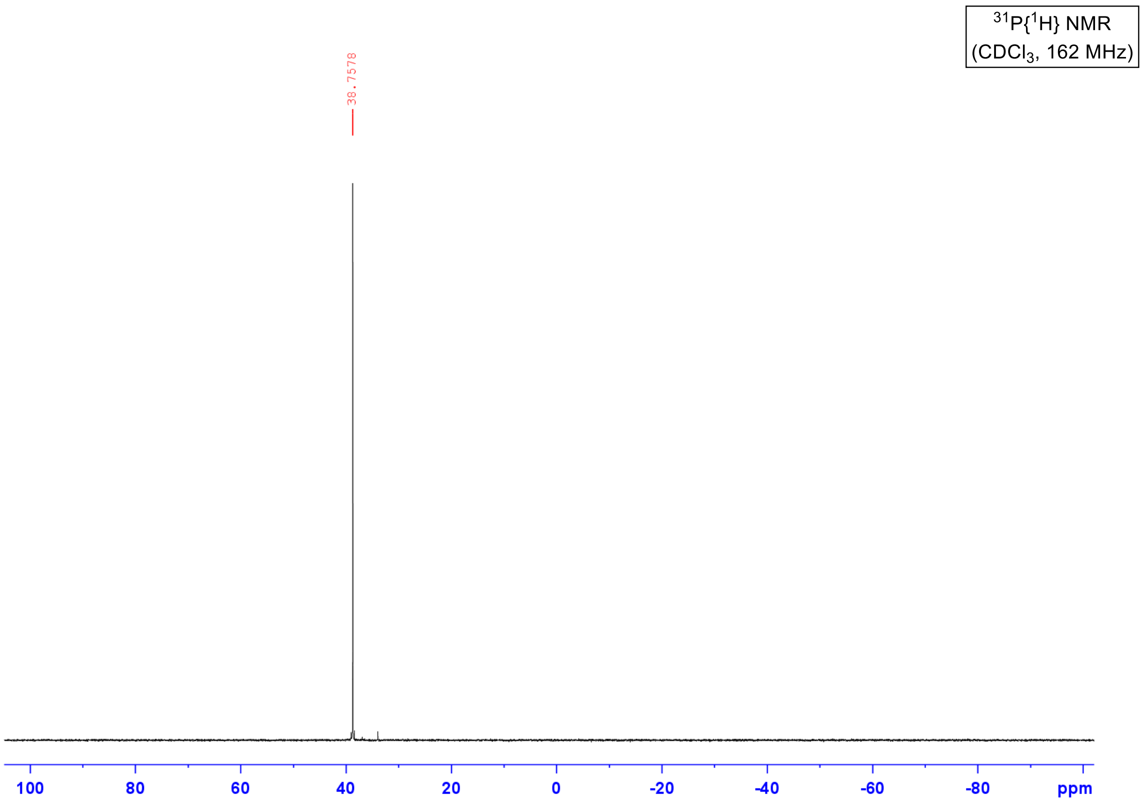

$^{31}P\{^1H\}$ NMR
($CDCl_3$, 162 MHz)
38.7578
100
80
60
40
20
0
-20
-40
-60
-80
ppm

[$^{1}H$, $^{13}C\{^{1}H\}$, and $^{31}P\{^{1}H\}$ NMR Spectra of **4ak**]

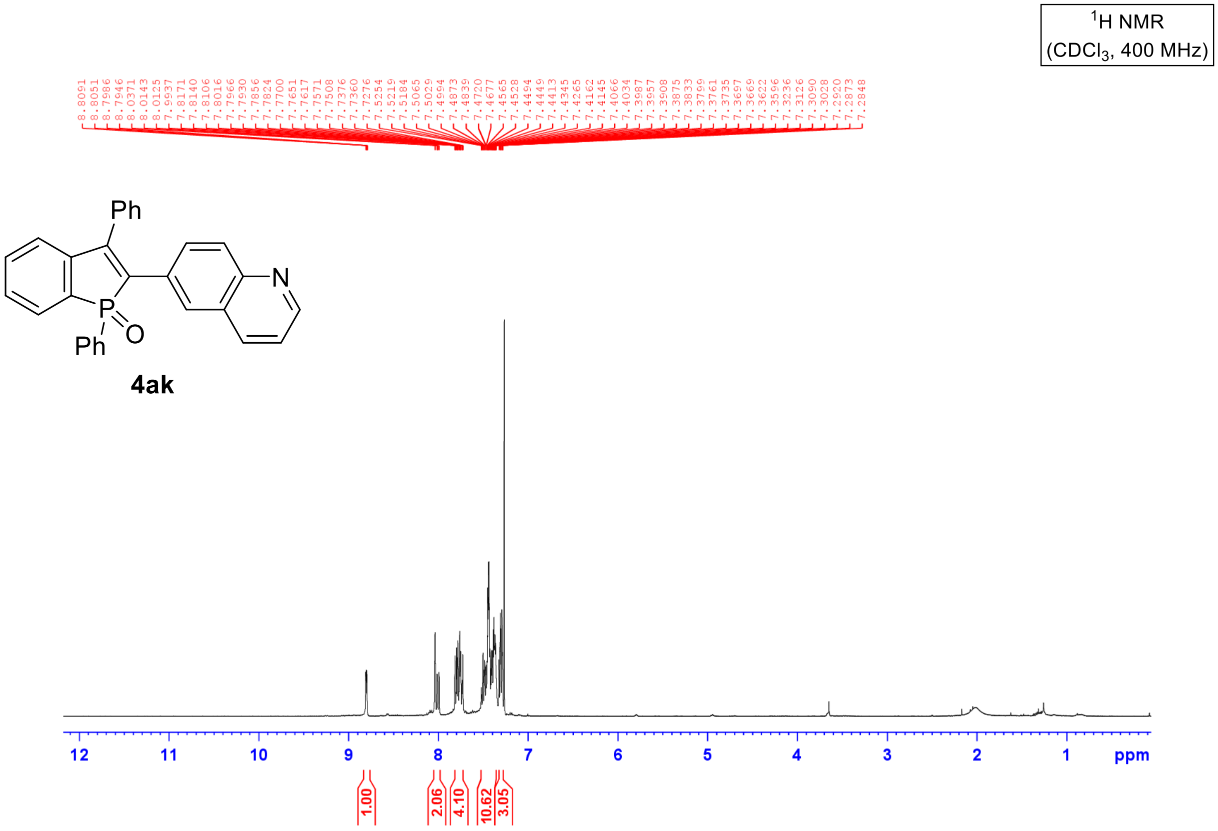


**4ak**

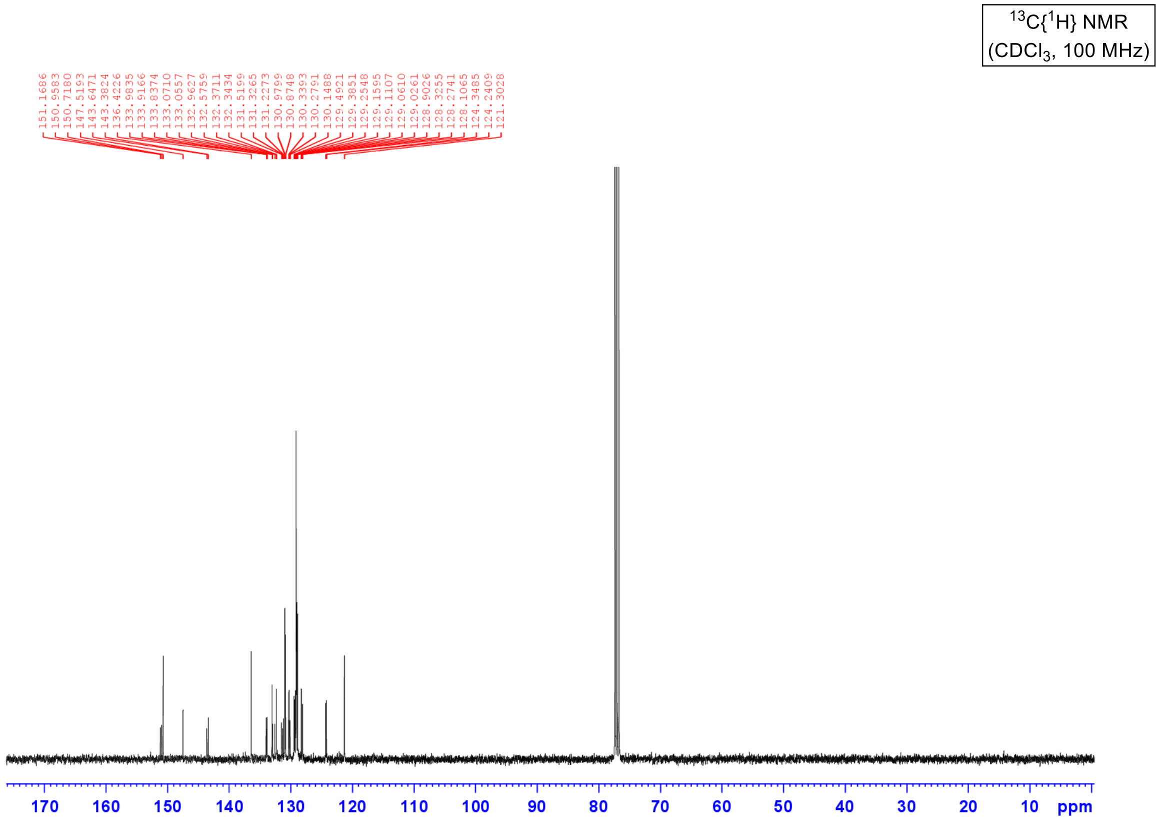

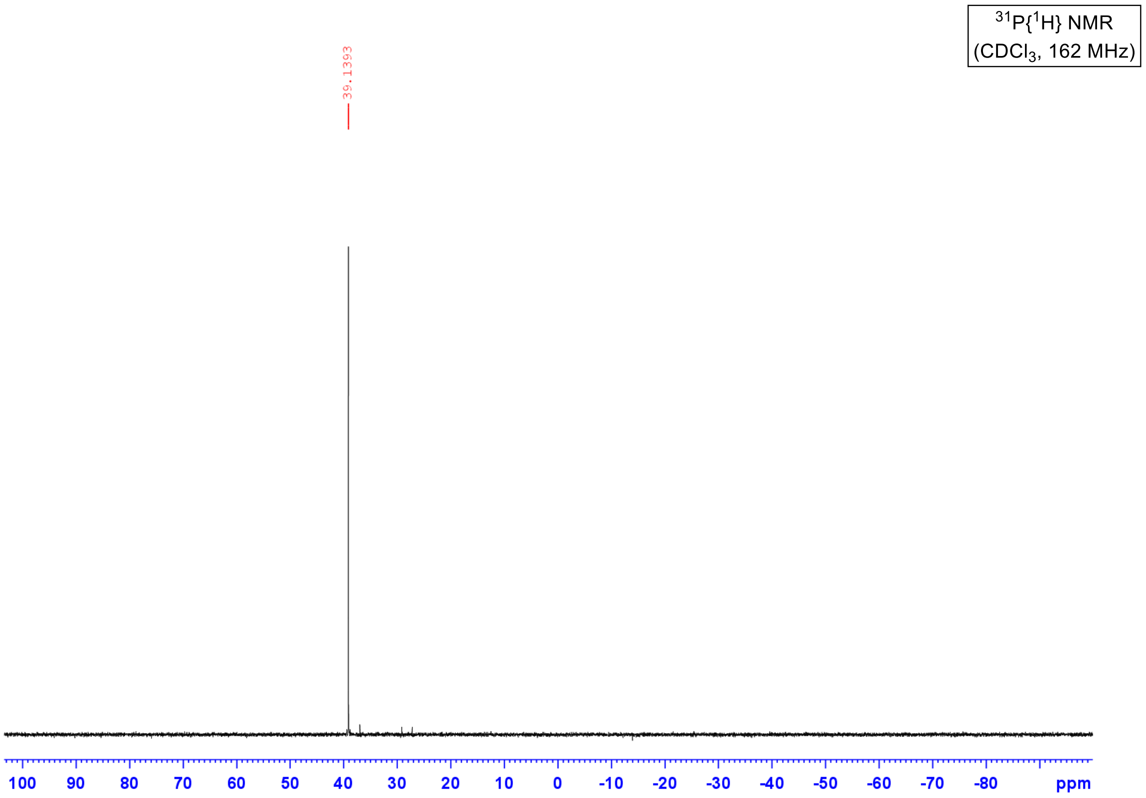

$^{31}P\{^1H\}$ NMR
($CDCl_3$, 162 MHz)
39.1393
100
90
80
70
60
50
40
30
20
10
0
-10
-20
-30
-40
-50
-60
-70
-80
ppm

[$^{1}H$, $^{13}C\{^{1}H\}$, and $^{31}P\{^{1}H\}$ NMR Spectra of **4al**]

$^{1}H$ NMR
($CDCl_3$, 400 MHz)

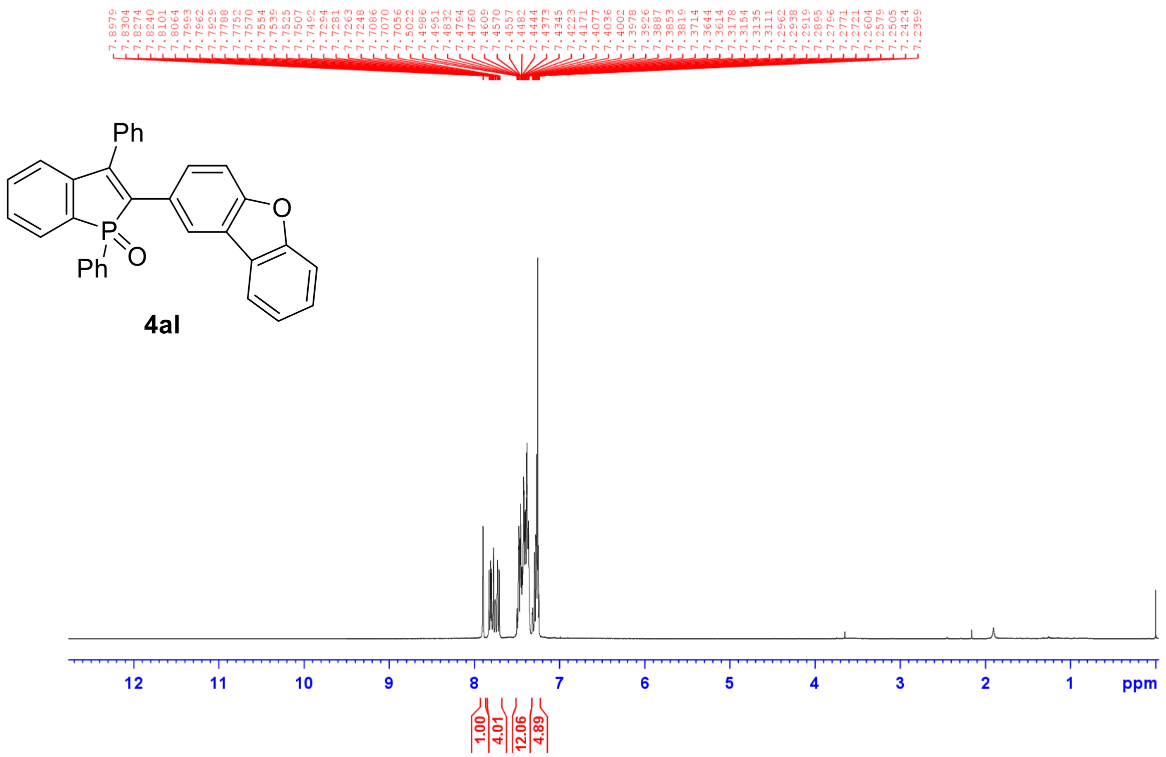

**4al**

$^{13}C\{^{1}H\}$ NMR
($CDCl_3$, 100 MHz)

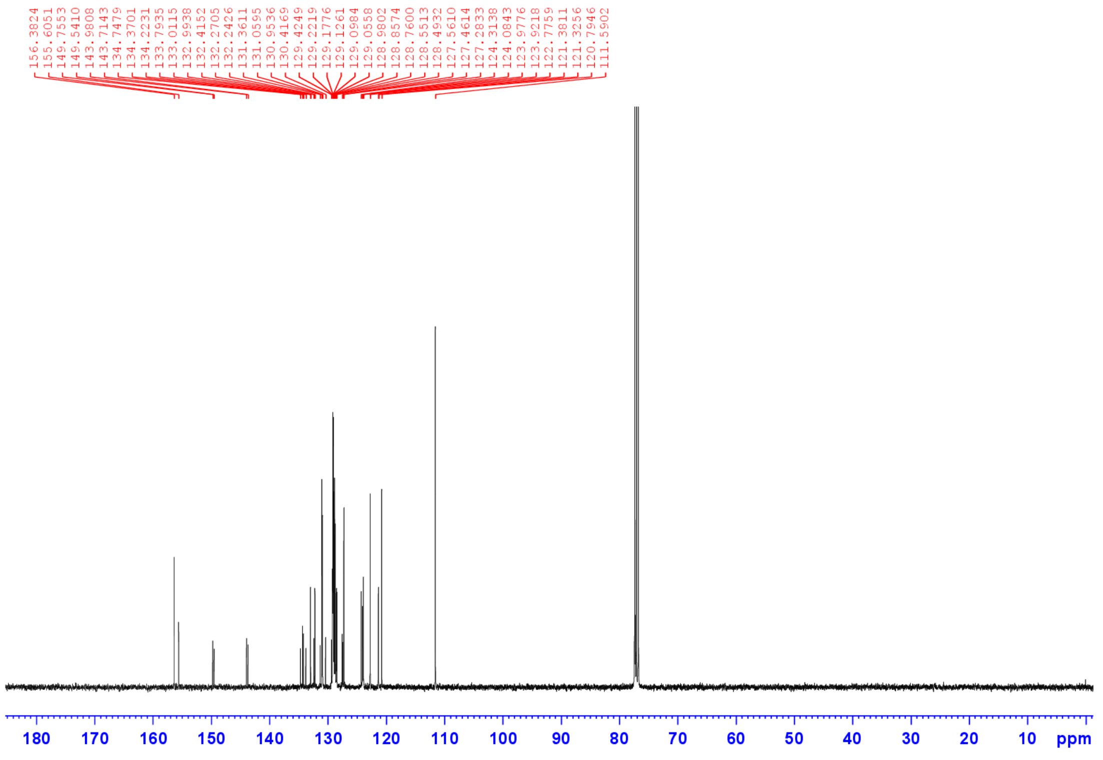

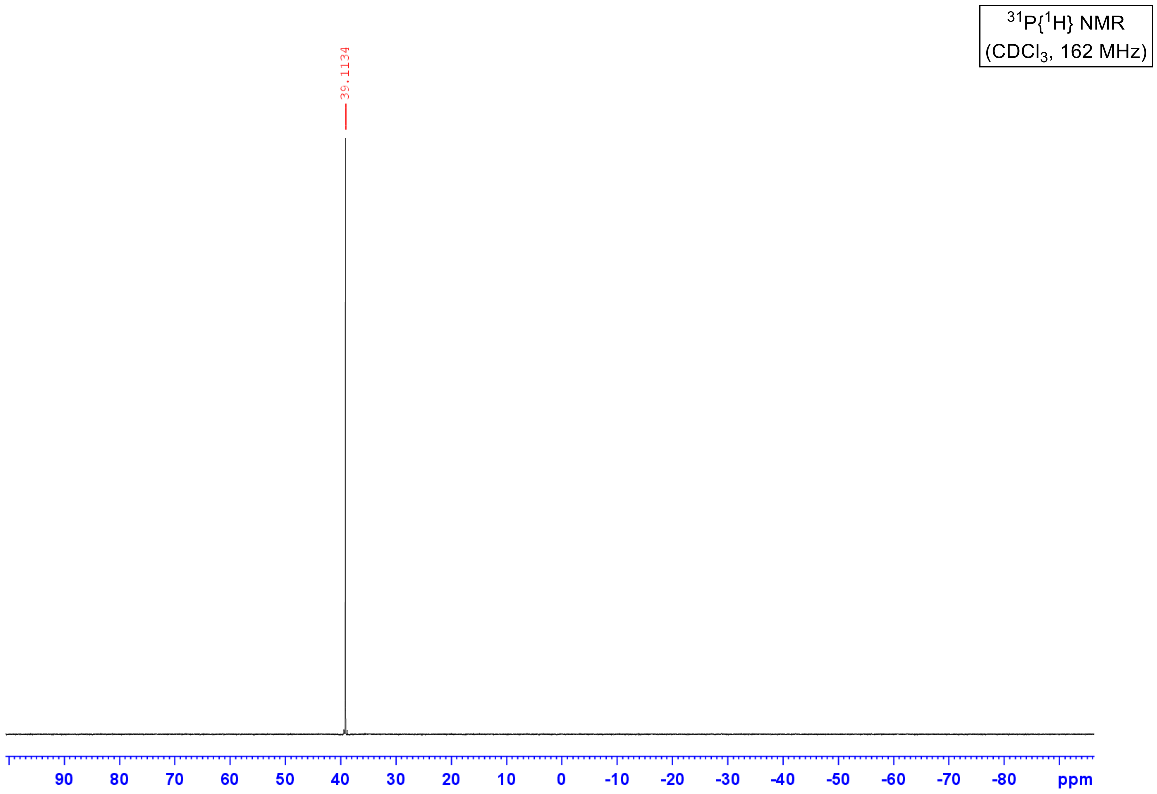
$^{31}P\{^1H\}$ NMR
($CDCl_3$, 162 MHz)
39.1134
90
80
70
60
50
40
30
20
10
0
-10
-20
-30
-40
-50
-60
-70
-80
ppm

[$^{1}H$, $^{13}C\{^{1}H\}$, and $^{31}P\{^{1}H\}$ NMR Spectra of **4am**]

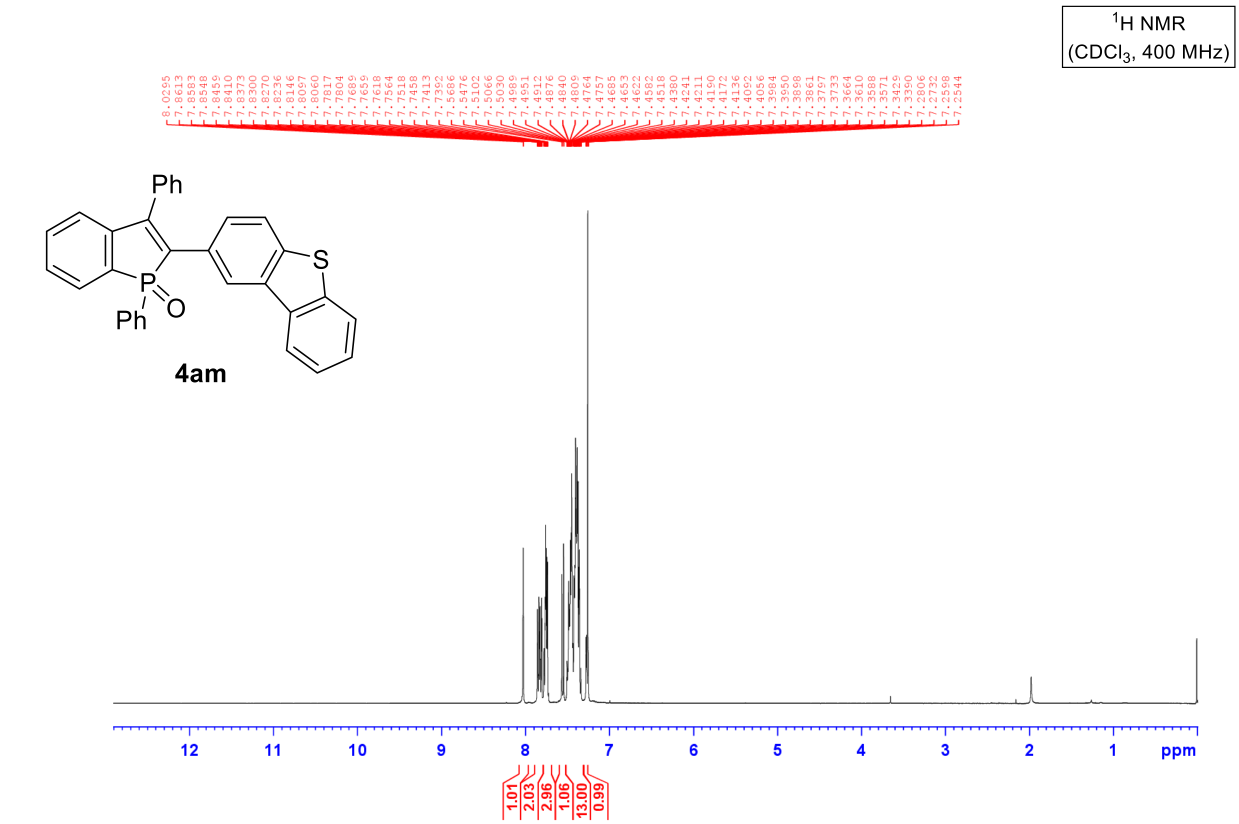


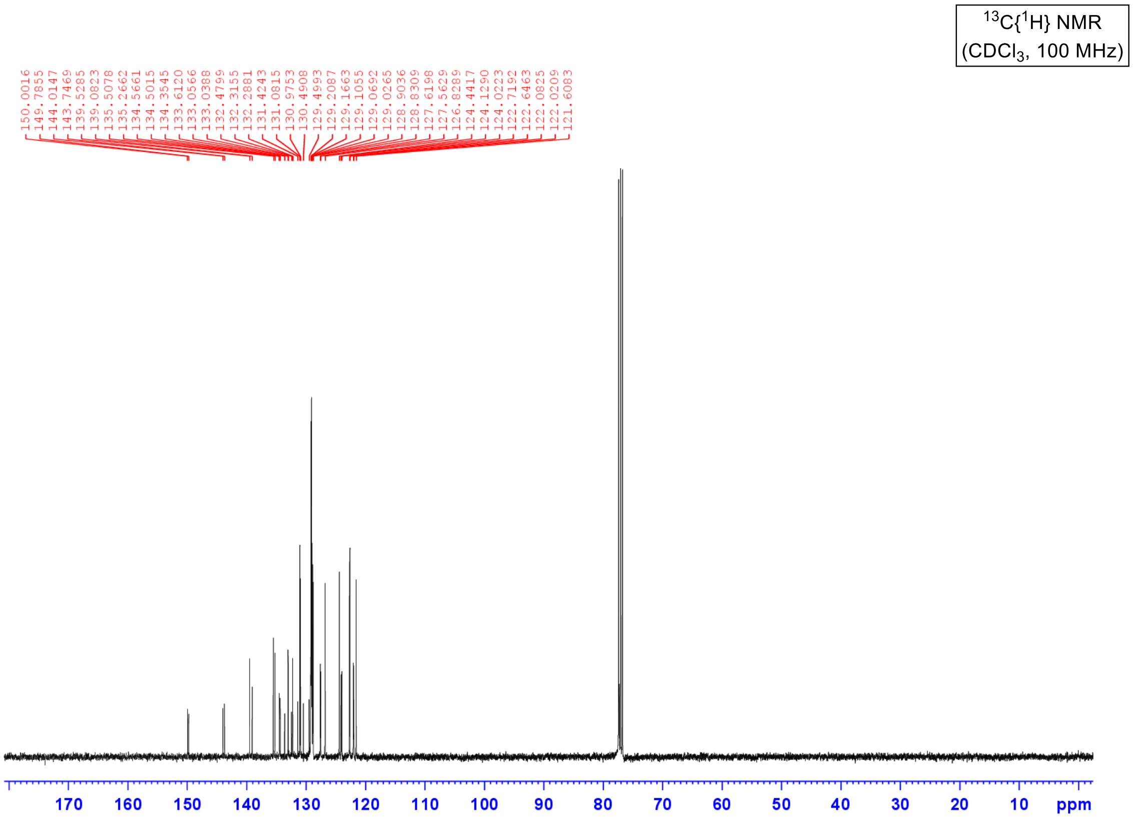

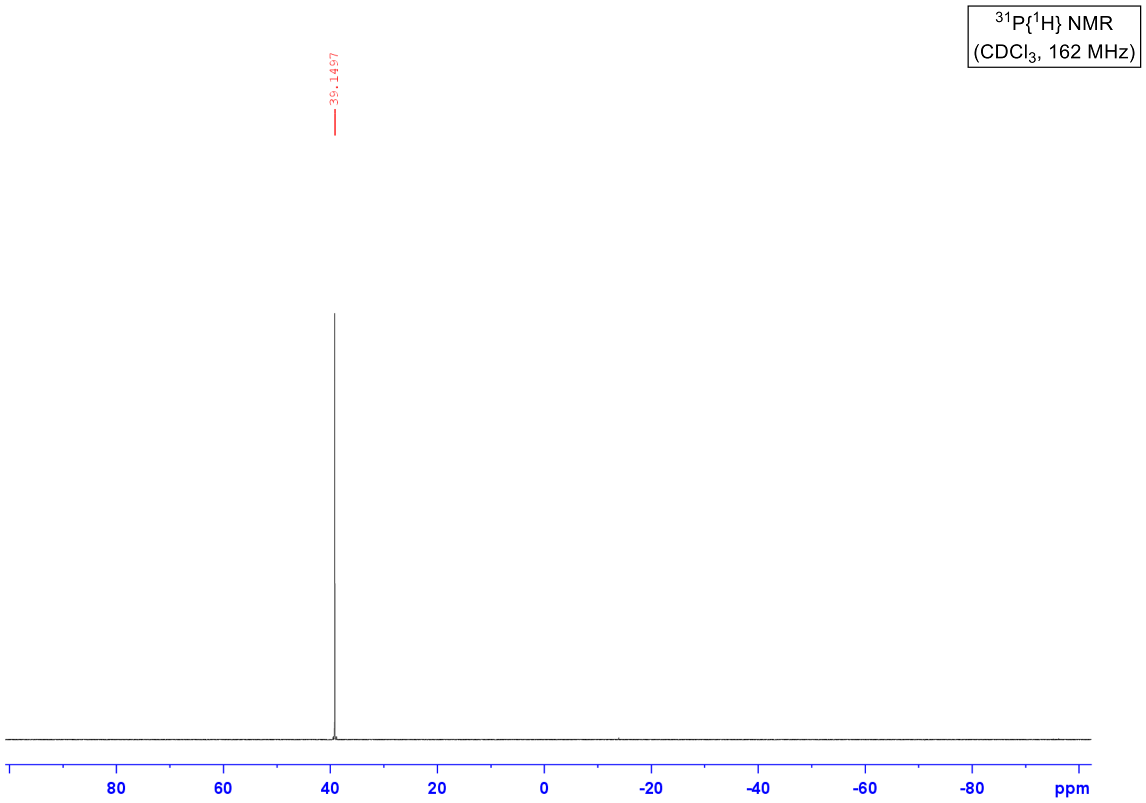

$^{31}P\{^1H\}$ NMR
($CDCl_3$, 162 MHz)
39.1497
80
60
40
20
0
-20
-40
-60
-80
ppm

[$^1$H, $^{13}$C{$^1$H}, and $^{31}$P{$^1$H} NMR Spectra of **4an**]

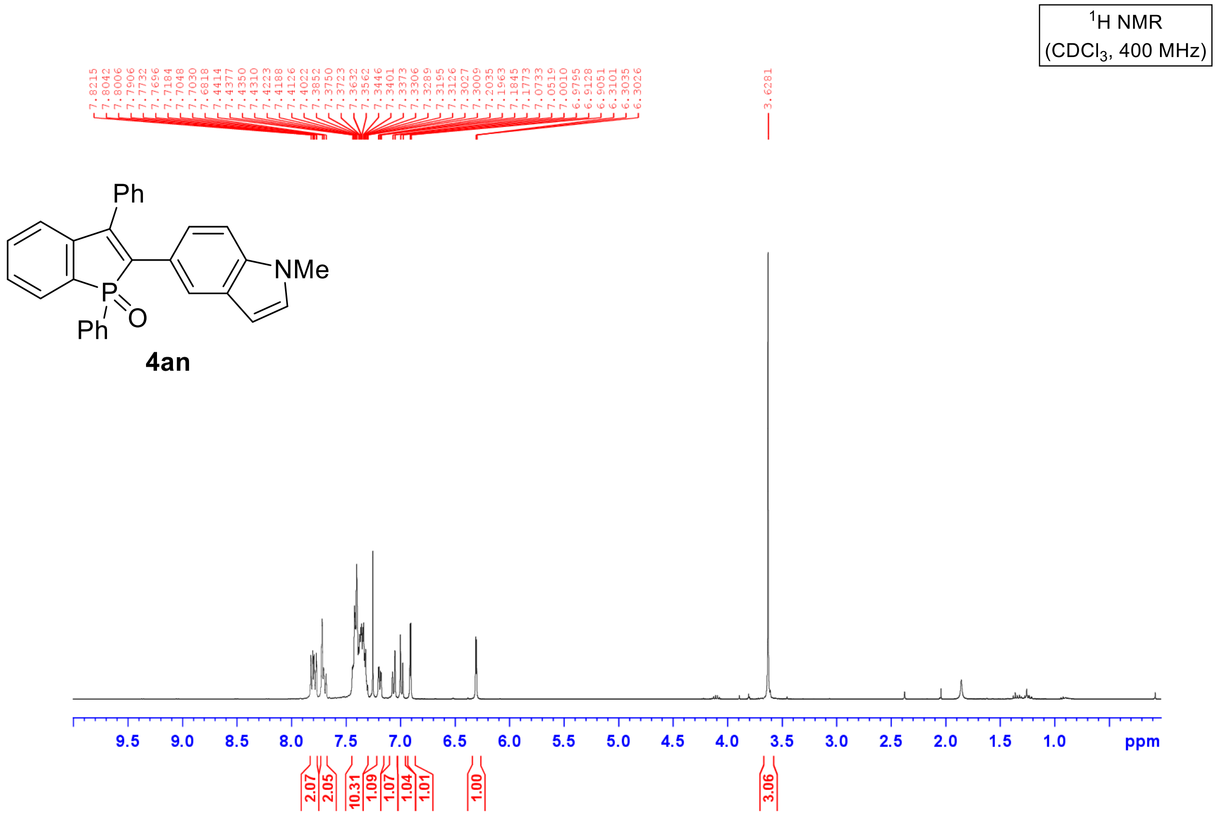


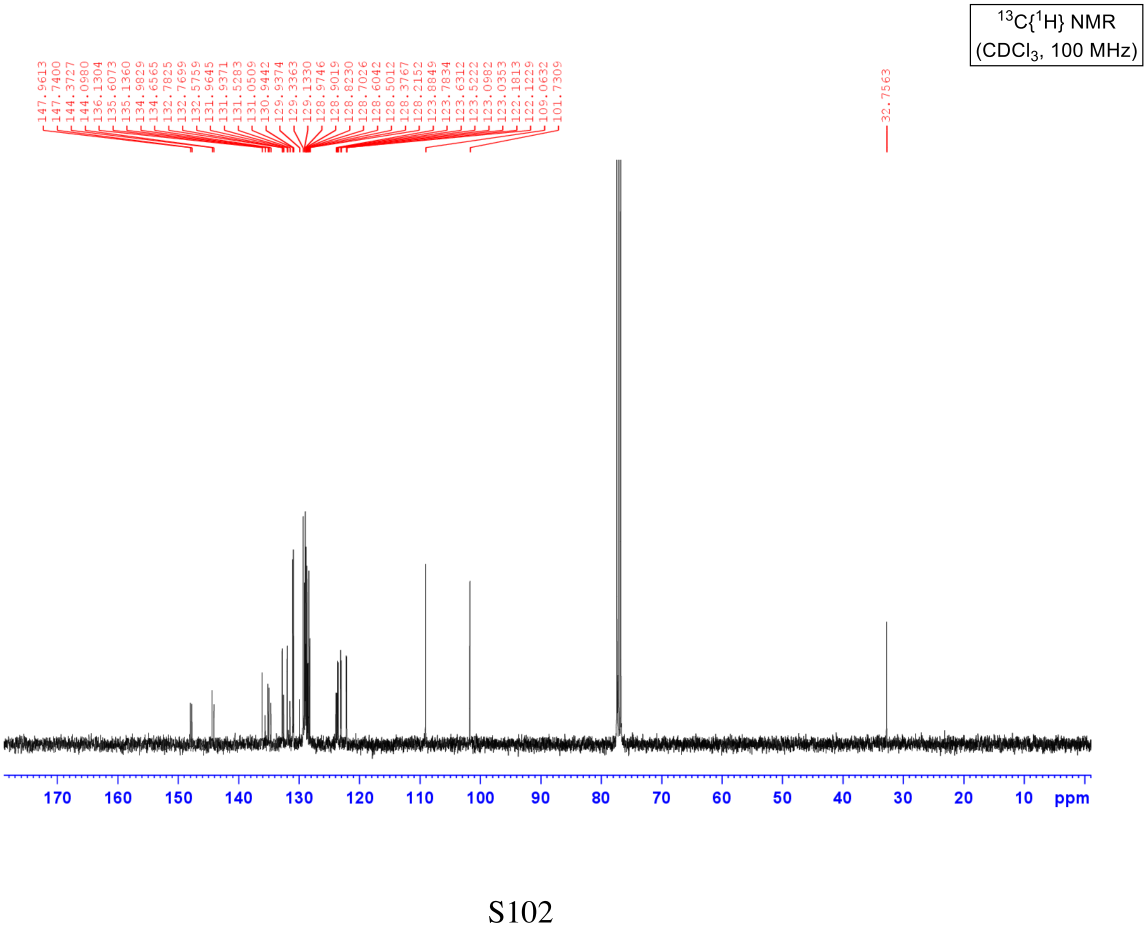

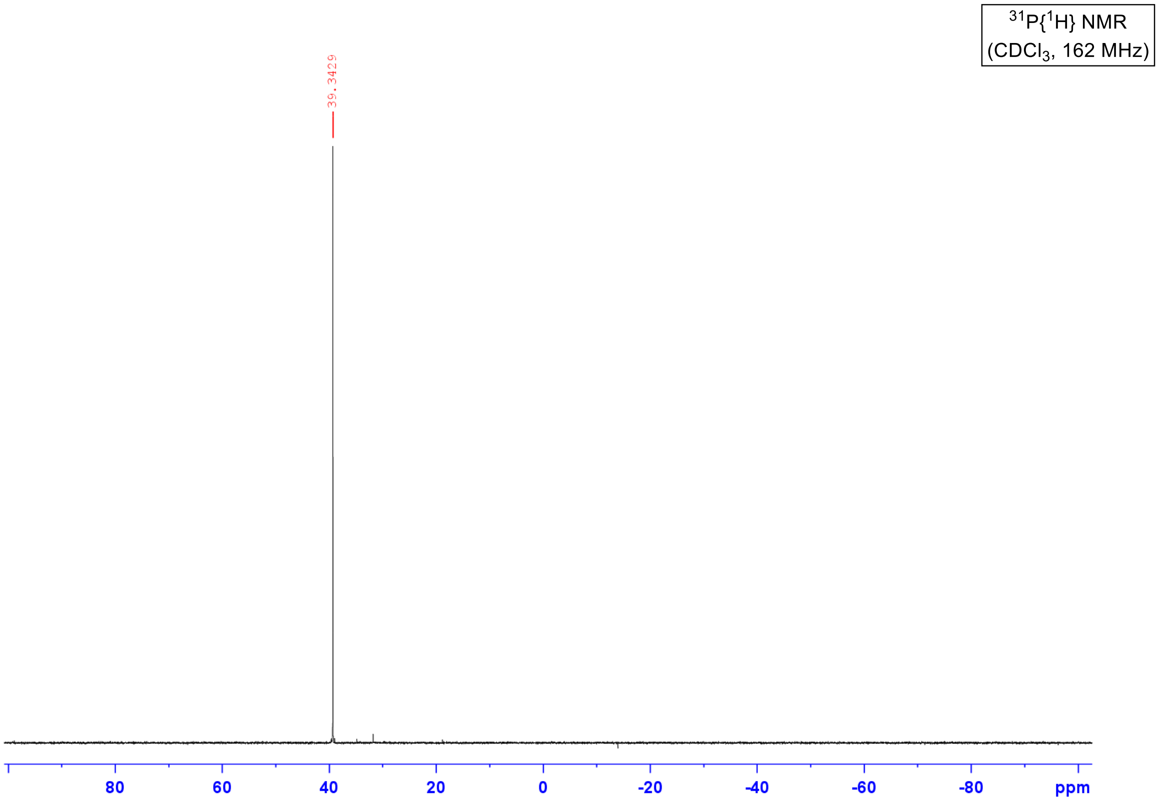

$^{31}P\{^1H\}$ NMR
($CDCl_3$, 162 MHz)
39.3429
80
60
40
20
0
-20
-40
-60
-80
ppm

[$^{1}H$, $^{13}C\{^{1}H\}$, and $^{31}P\{^{1}H\}$ NMR Spectra of **4ao**]

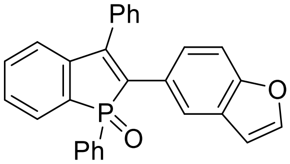


**4ao**

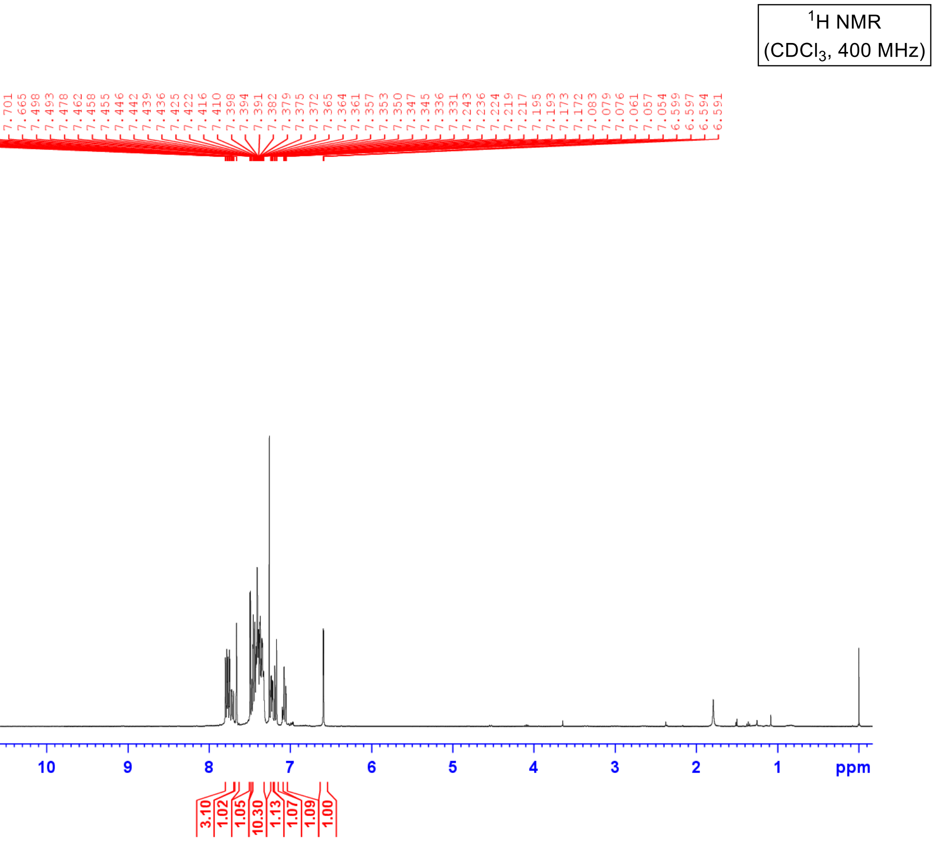


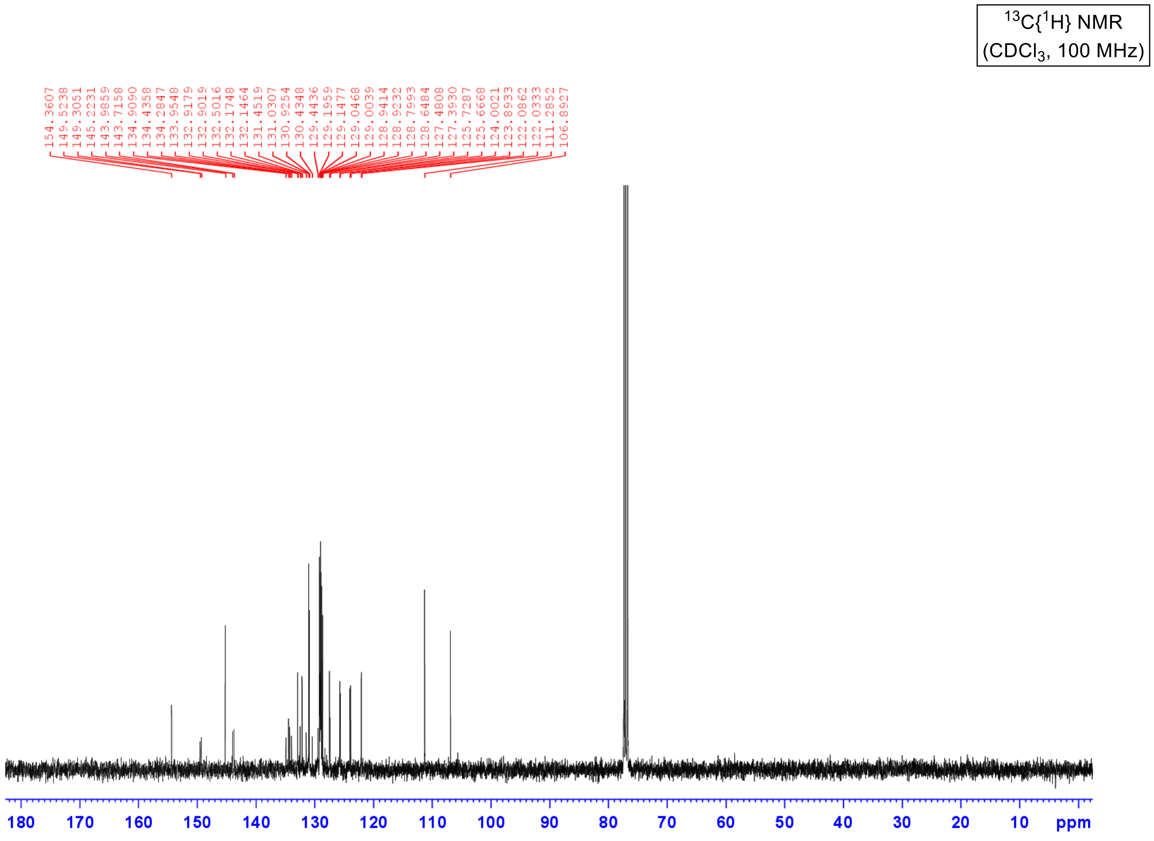

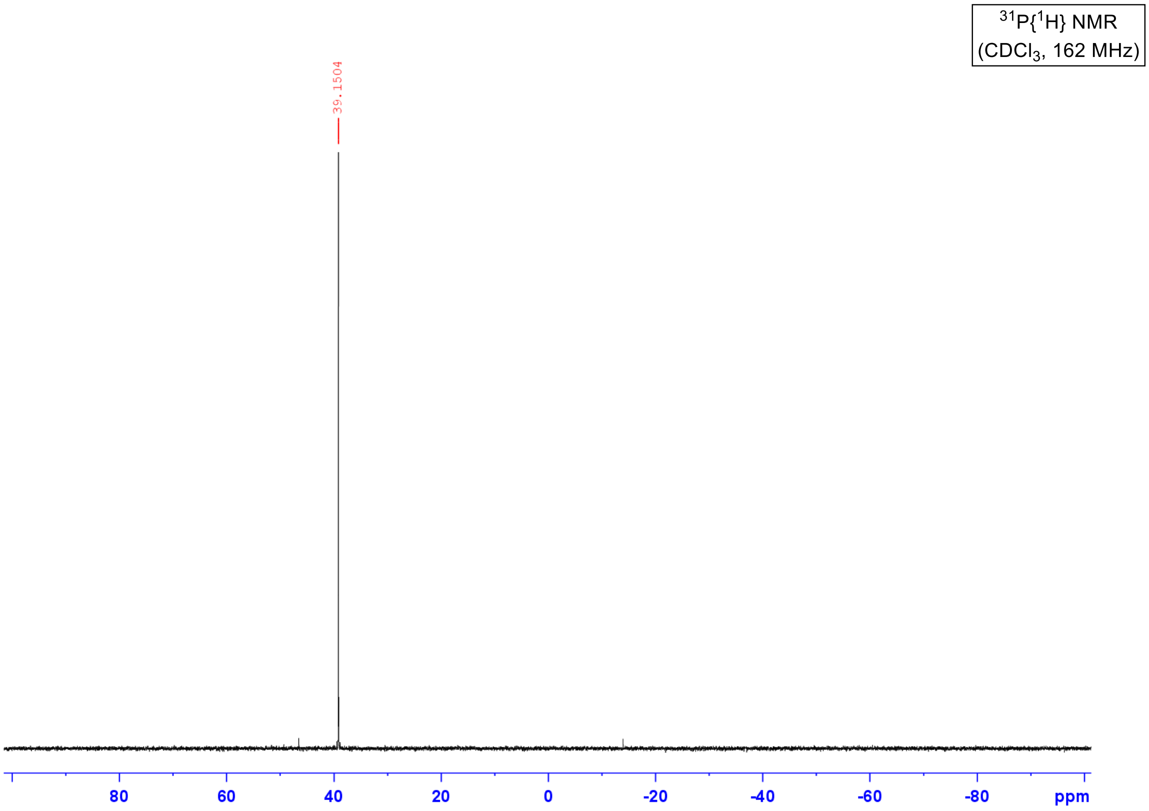
$^{31}P\{^1H\}$ NMR
($CDCl_3$, 162 MHz)
39.1504
80
60
40
20
0
-20
-40
-60
-80
ppm

[$^{1}H$, $^{13}C\{^{1}H\}$, and $^{31}P\{^{1}H\}$ NMR Spectra of **4ap**]

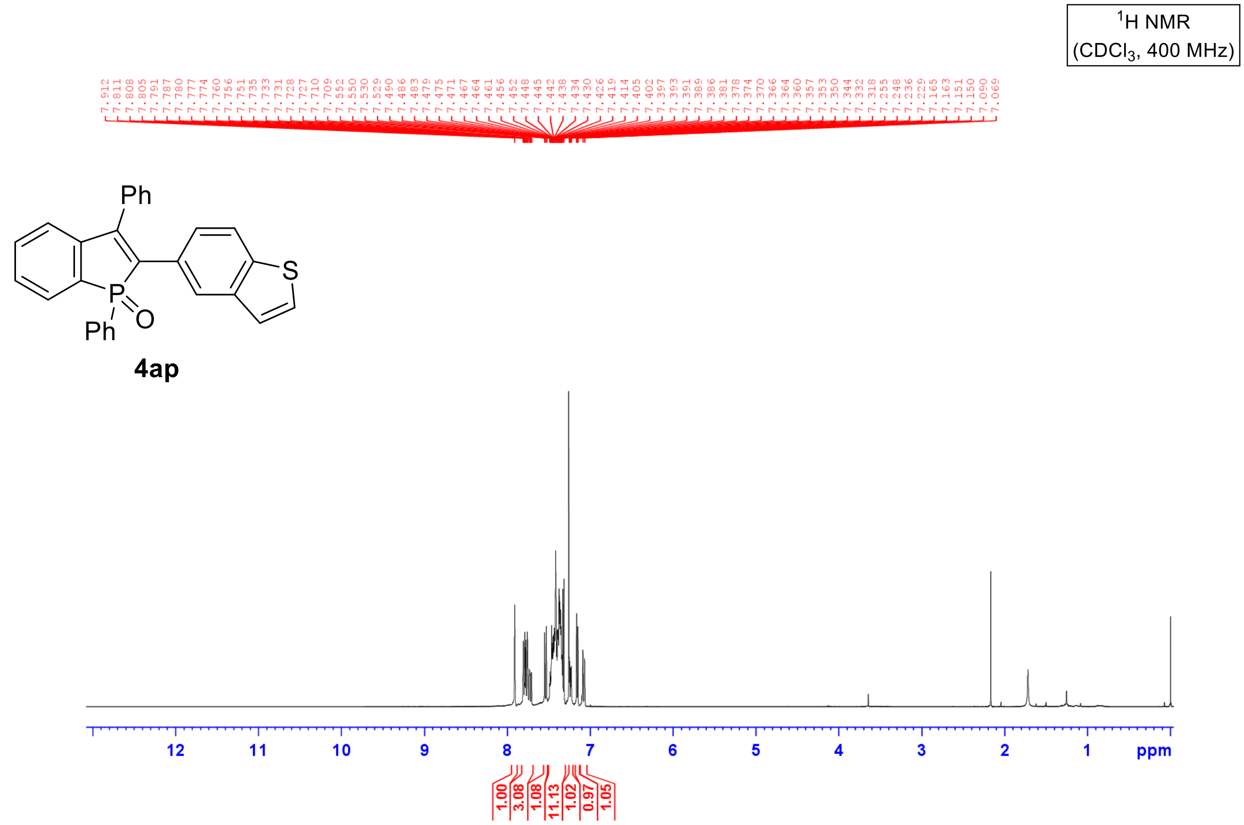


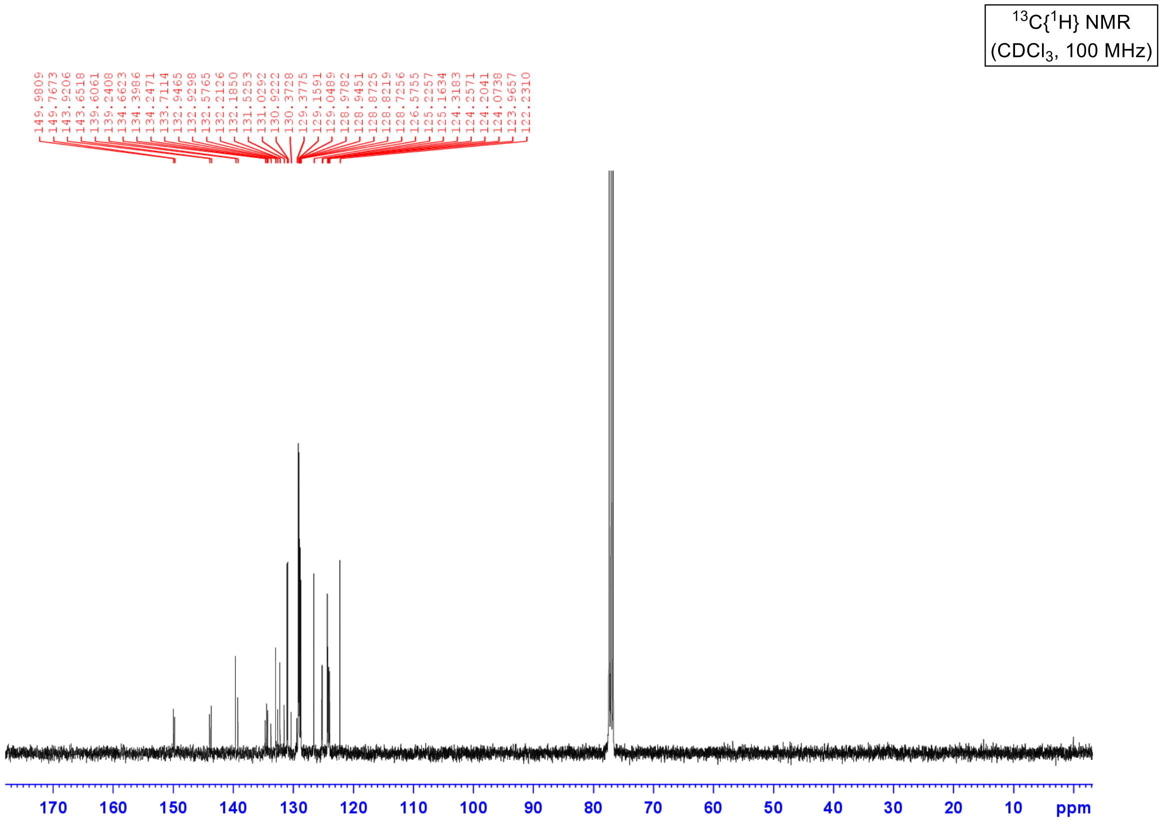

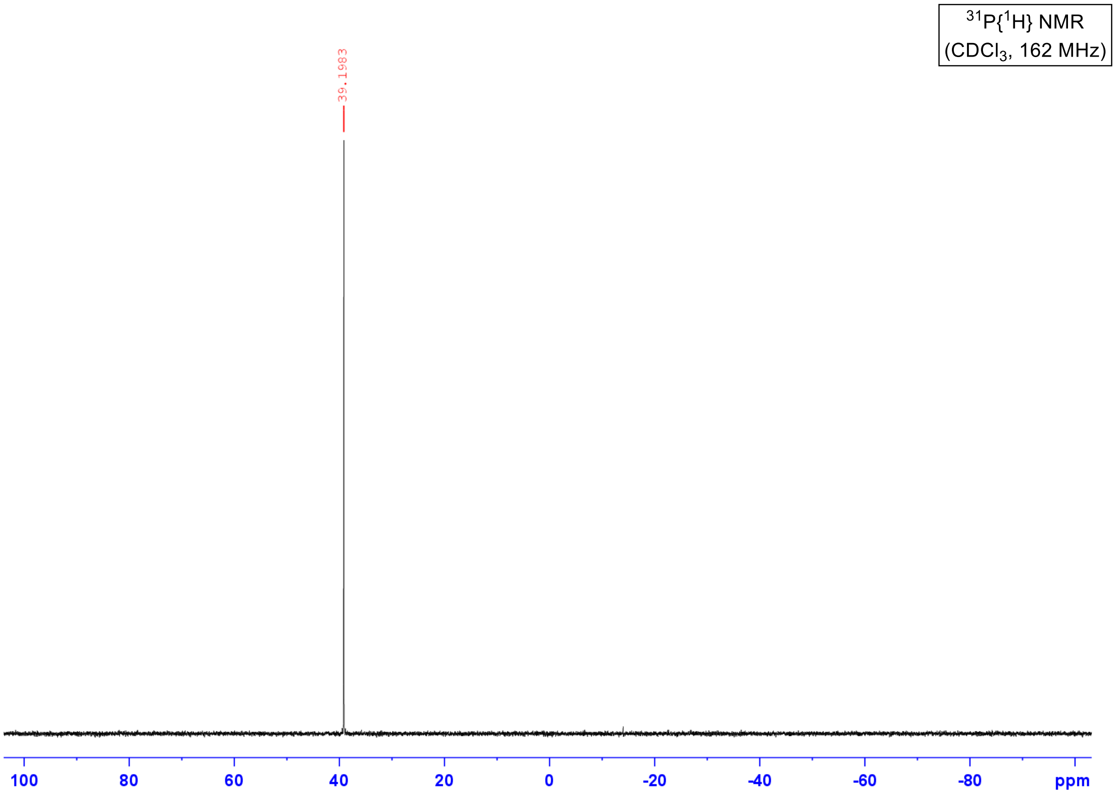

$^{31}P\{^1H\}$ NMR
($CDCl_3$, 162 MHz)
39.1983
100
80
60
40
20
0
-20
-40
-60
-80
ppm

[$^{1}H$, $^{13}C\{^{1}H\}$, and $^{31}P\{^{1}H\}$ NMR Spectra of **4aq**]

$^{1}H$ NMR
($CDCl_3$, 400 MHz)

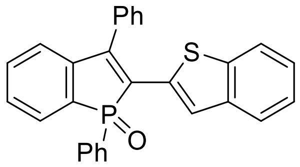

**4aq**

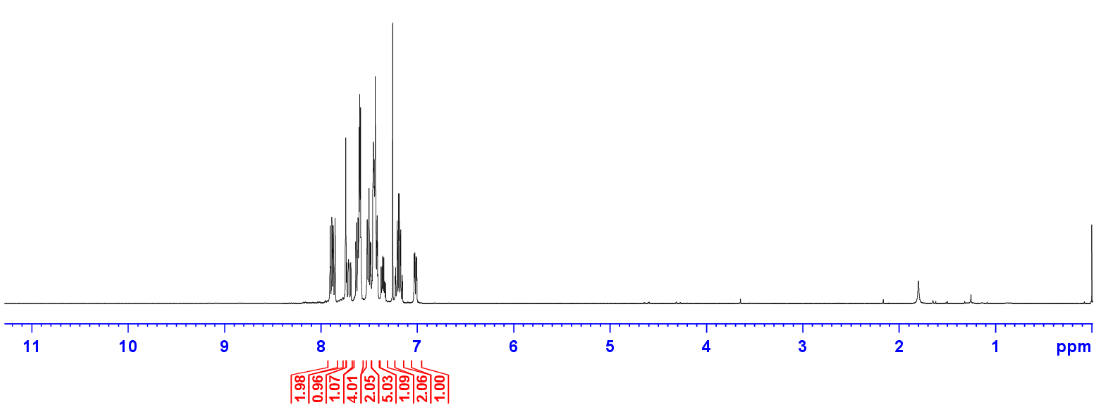


$^{13}C\{^{1}H\}$ NMR
($CDCl_3$, 100 MHz)

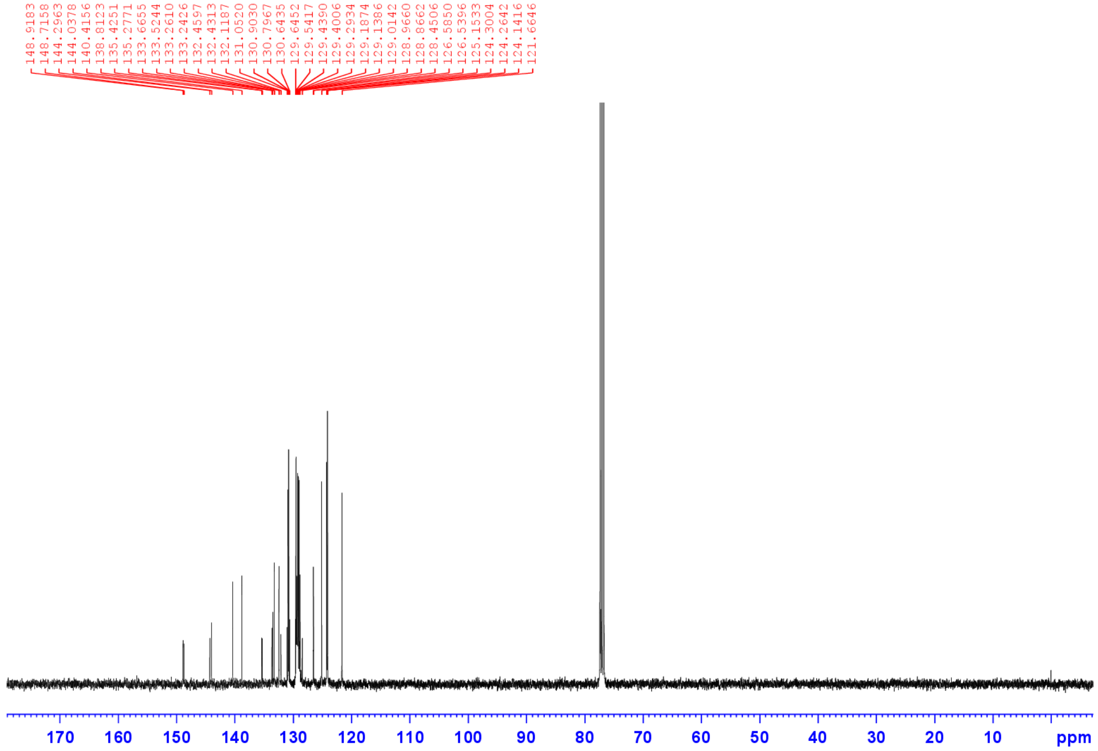

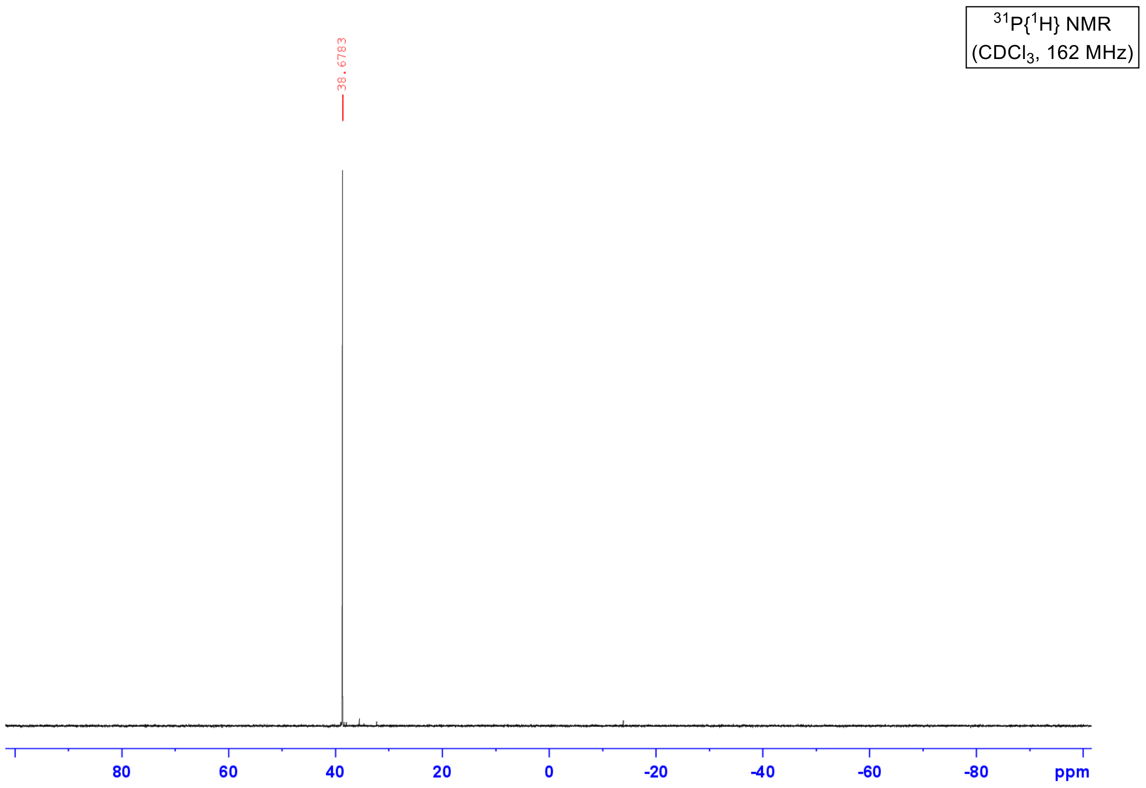

$^{31}P\{^{1}H\}$ NMR
($CDCl_3$, 162 MHz)
38.6783
80
60
40
20
0
-20
-40
-60
-80
ppm

[$^{1}H$, $^{13}C\{^{1}H\}$, and $^{31}P\{^{1}H\}$ NMR Spectra of **4ar**]

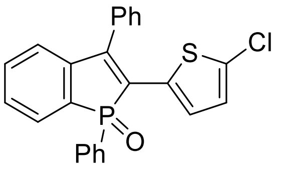

**4ar**

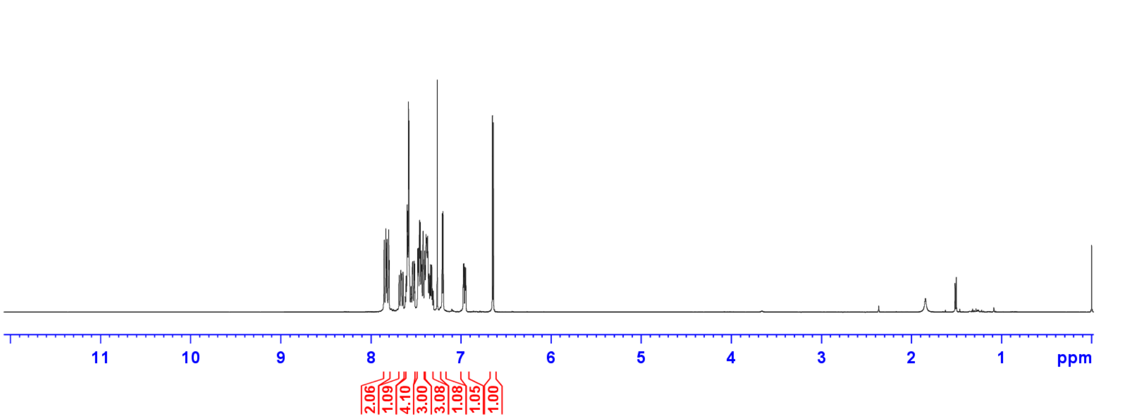


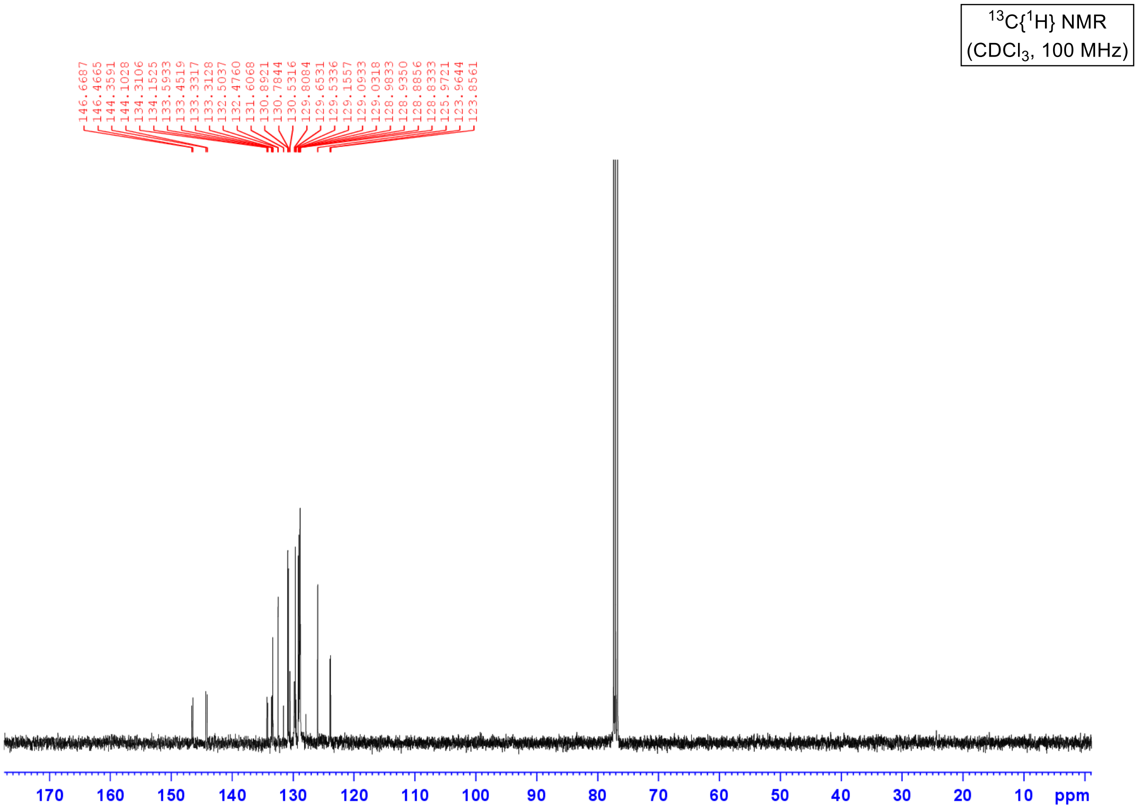

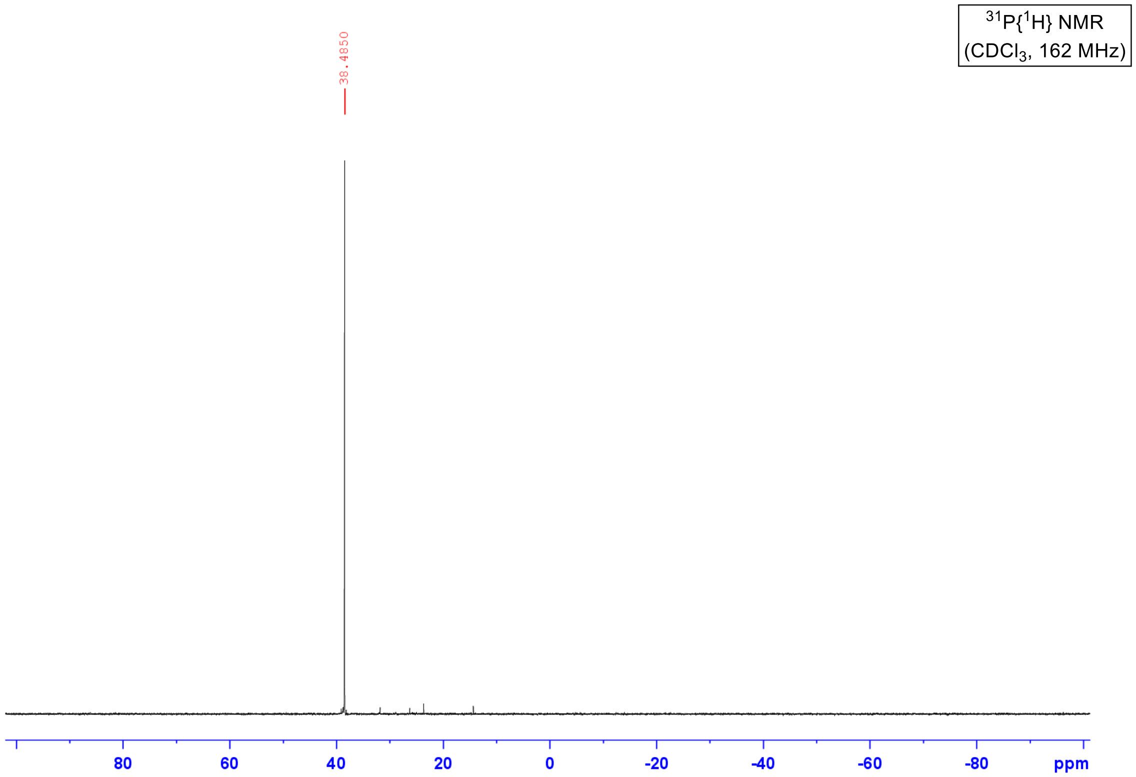
38.4850
$^{31}P\{^1H\}$ NMR
($CDCl_3$, 162 MHz)
80
60
40
20
0
-20
-40
-60
-80
ppm

[$^{1}H$, $^{13}C\{^{1}H\}$, and $^{31}P\{^{1}H\}$ NMR Spectra of **4as**]

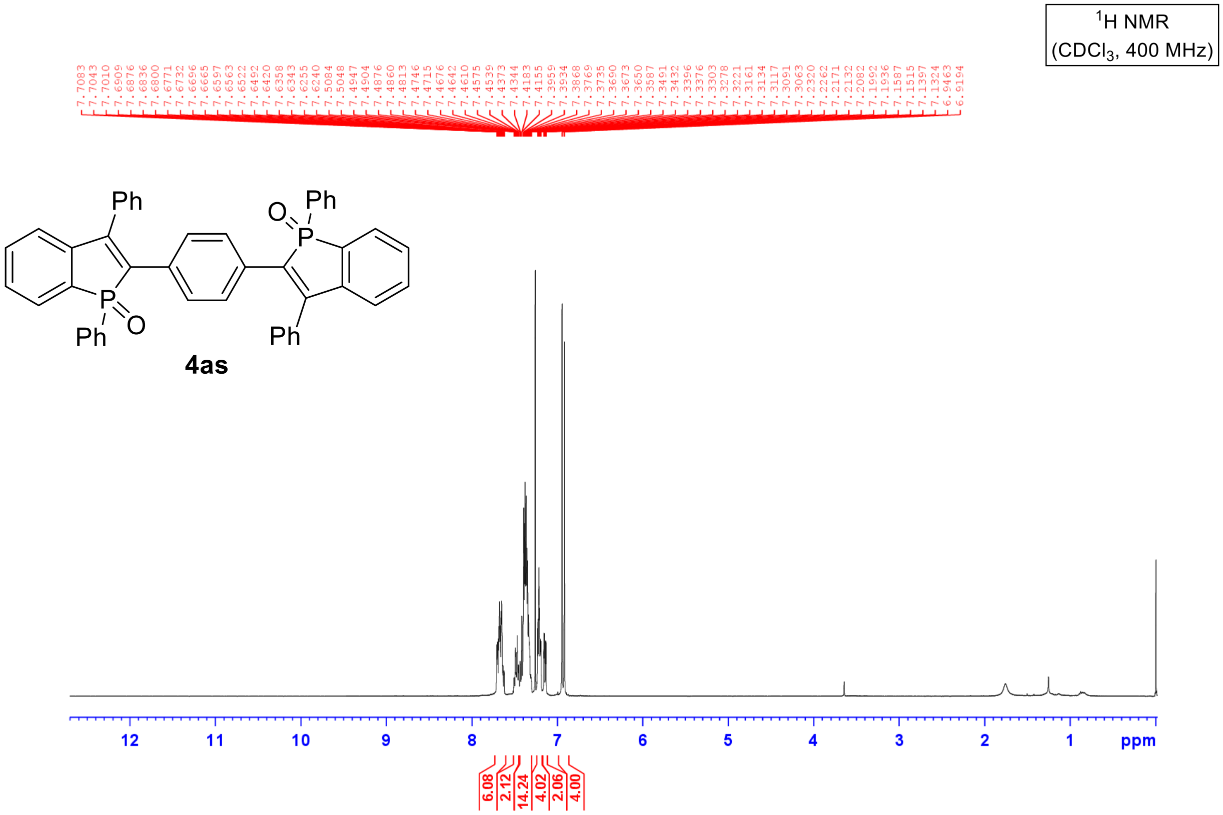


**4as**

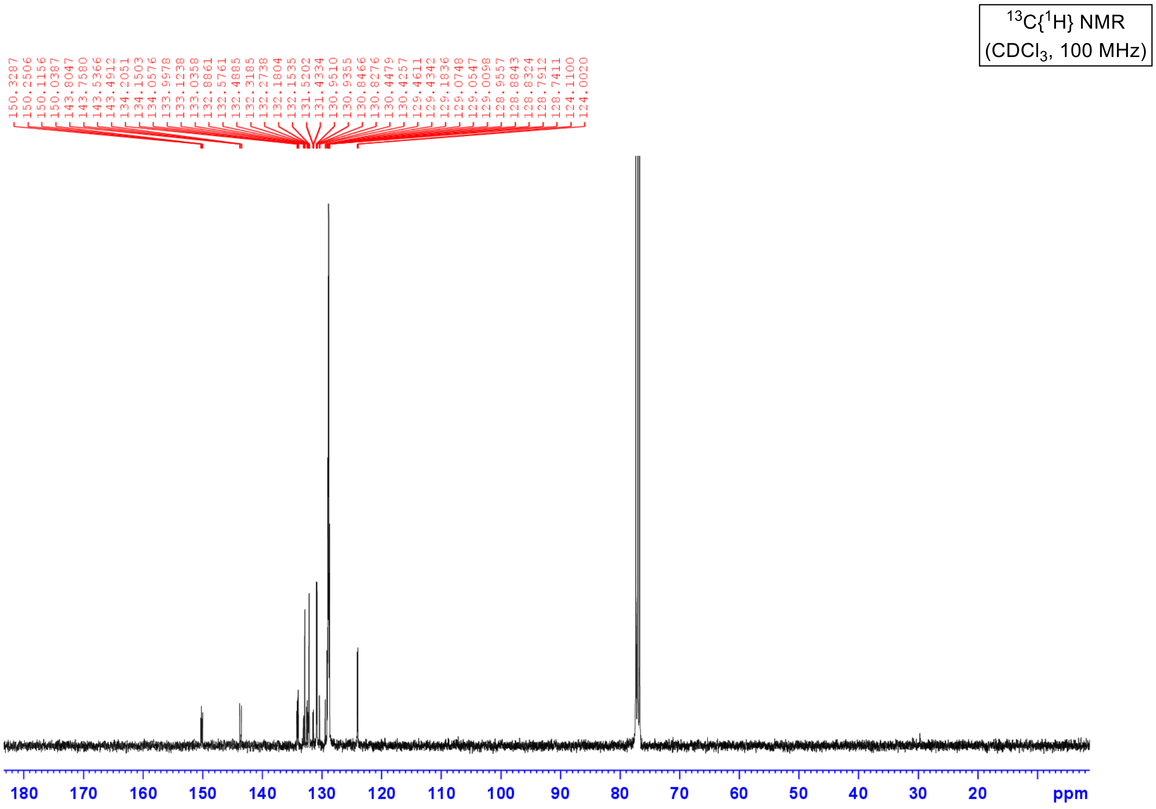

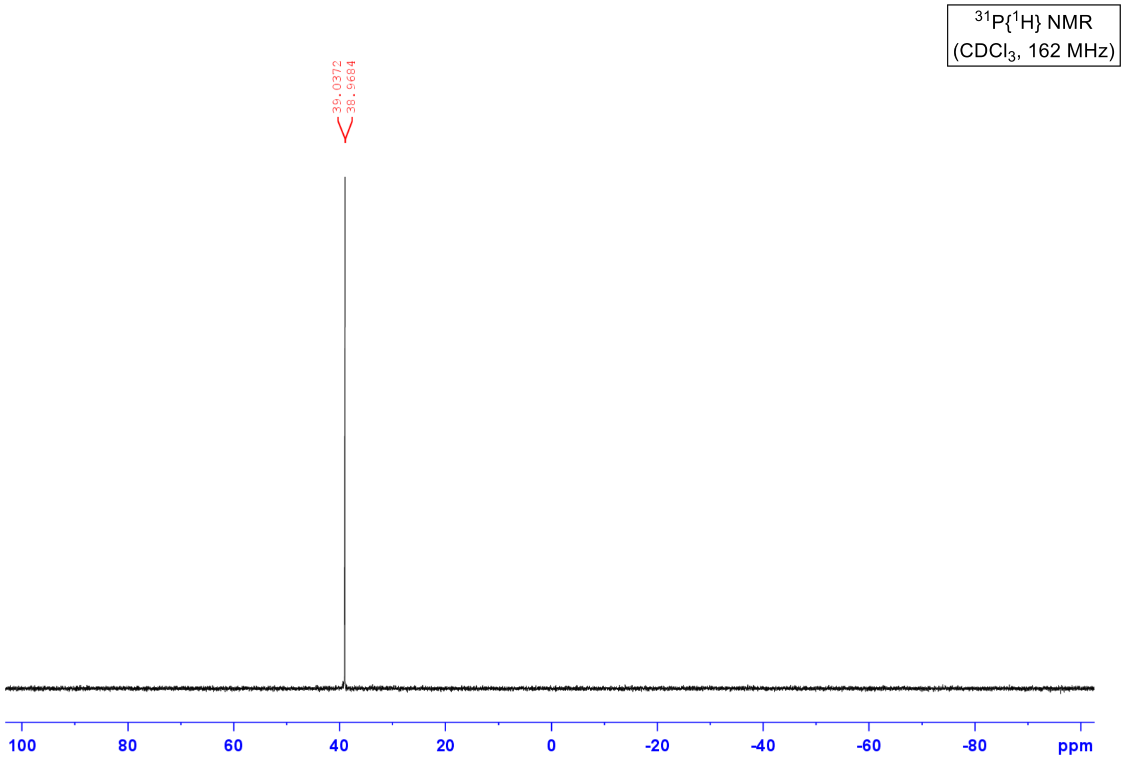

$^{31}P\{^{1}H\}$ NMR
($CDCl_3$, 162 MHz)
39.0372
38.9684
100
80
60
40
20
0
-20
-40
-60
-80
ppm

[$^{1}$H, $^{13}$C{$^{1}$H}, and $^{31}$P{$^{1}$H} NMR Spectra of **4at**]

$^{1}$H NMR
(CDCl$_3$, 400 MHz)

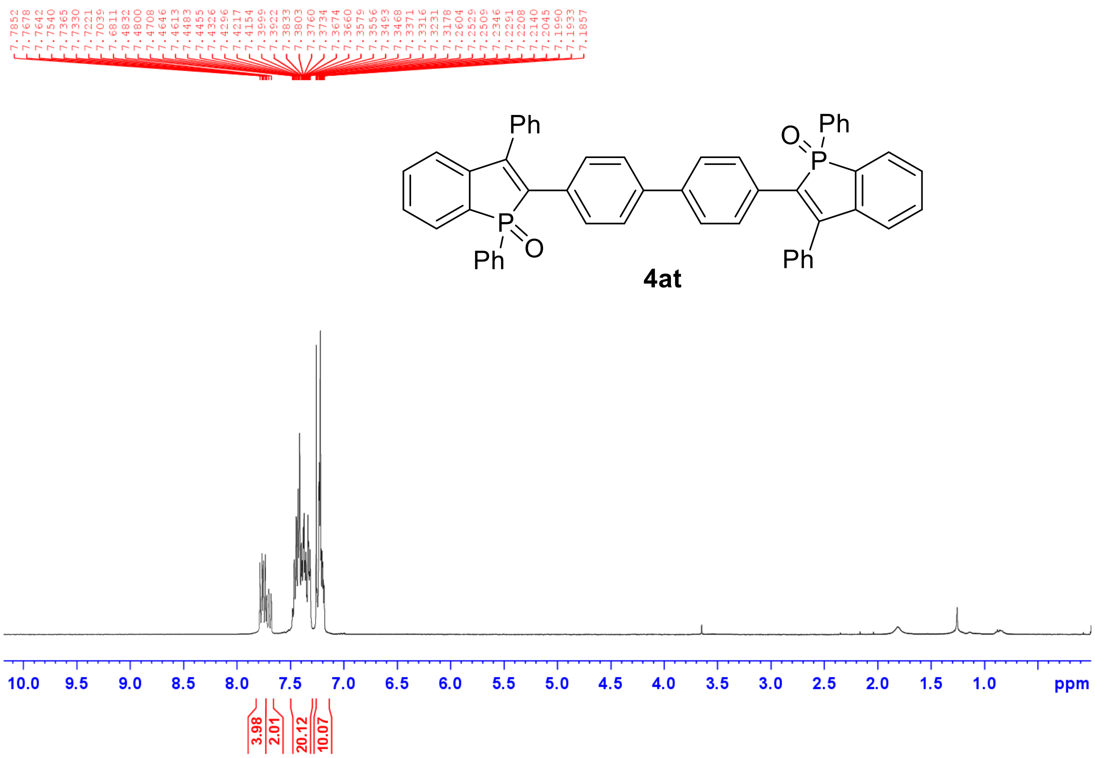


$^{13}$C{$^{1}$H} NMR
(CDCl$_3$, 100 MHz)

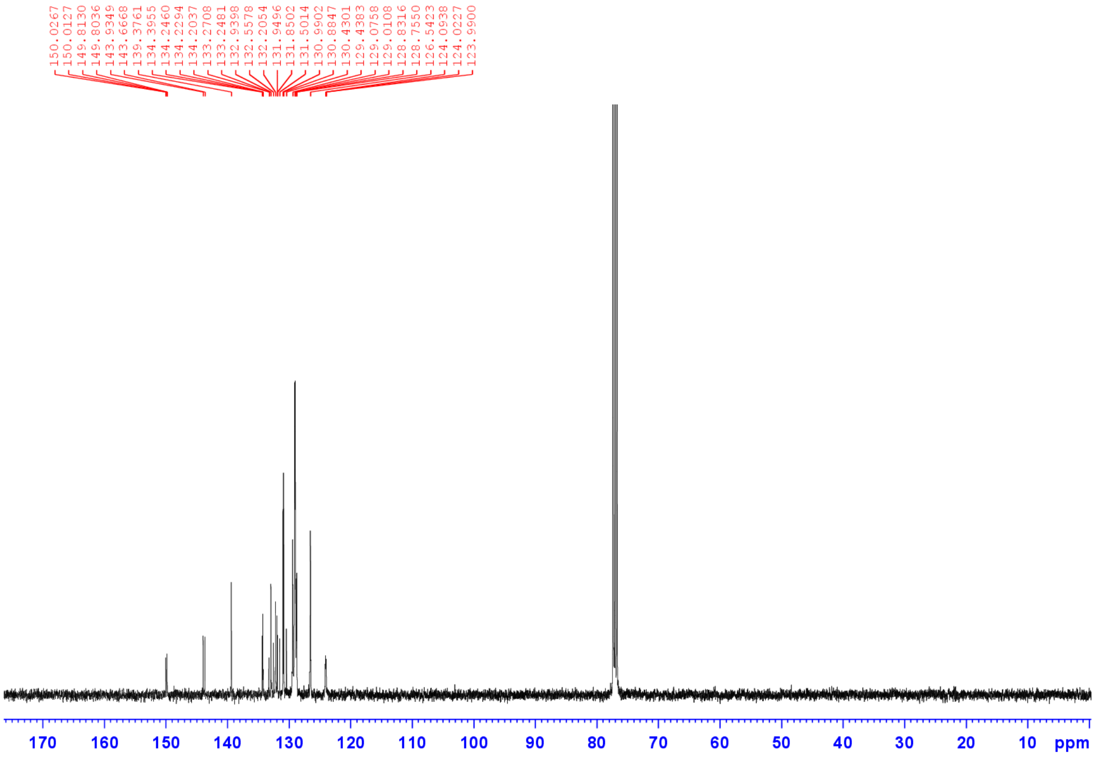

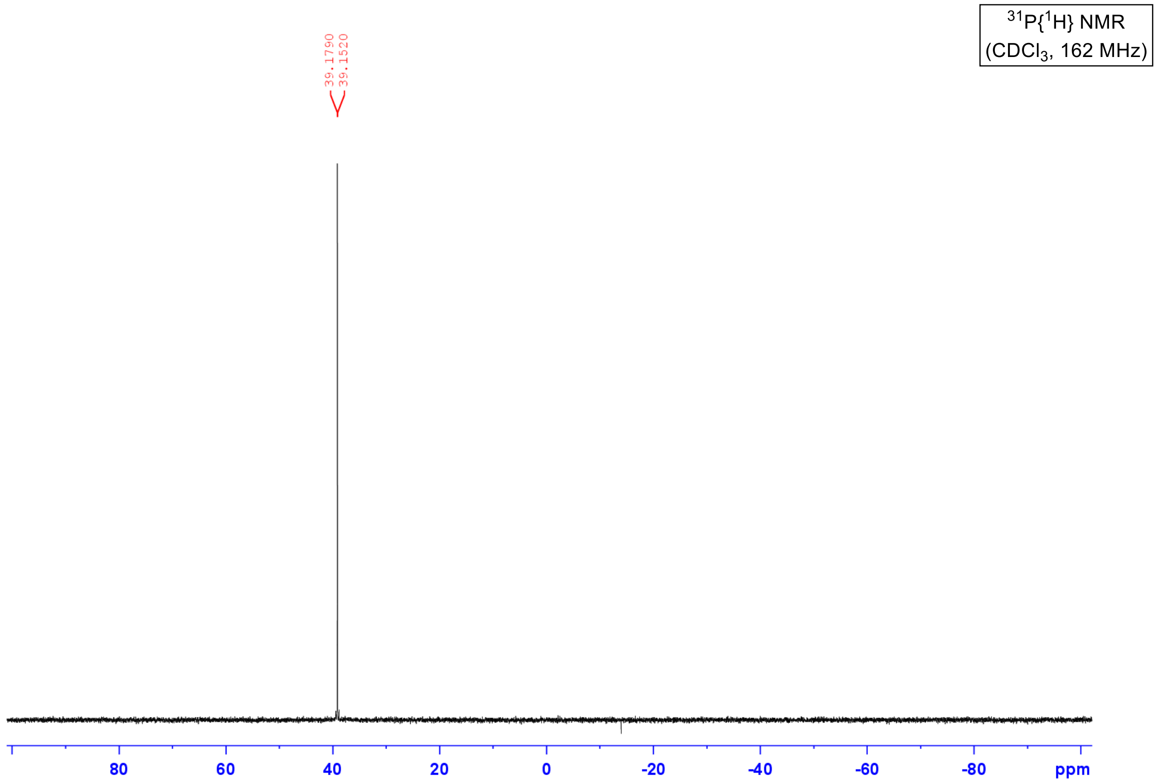

$^{31}P\{^{1}H\}$ NMR
($CDCl_3$, 162 MHz)
39.1790
39.1520
80
60
40
20
0
-20
-40
-60
-80
ppm

[$^{1}H$, $^{13}C\{^{1}H\}$, and $^{31}P\{^{1}H\}$ NMR Spectra of **4au**]

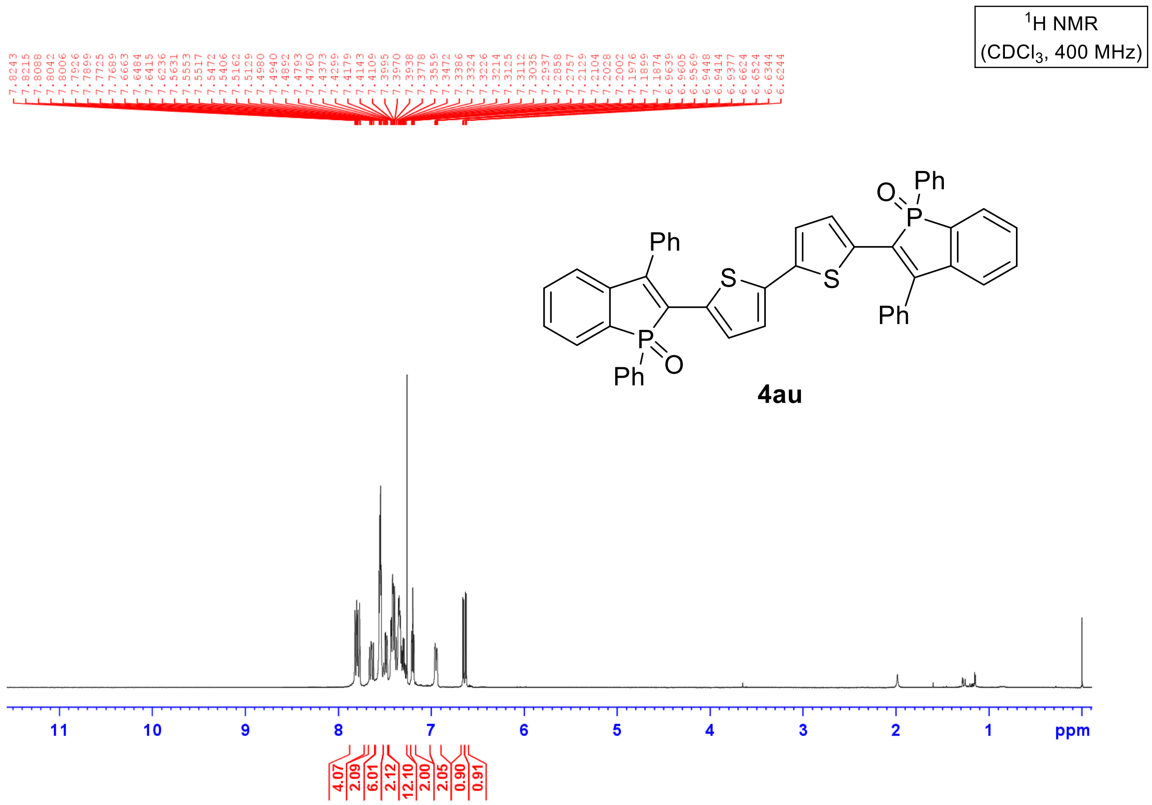


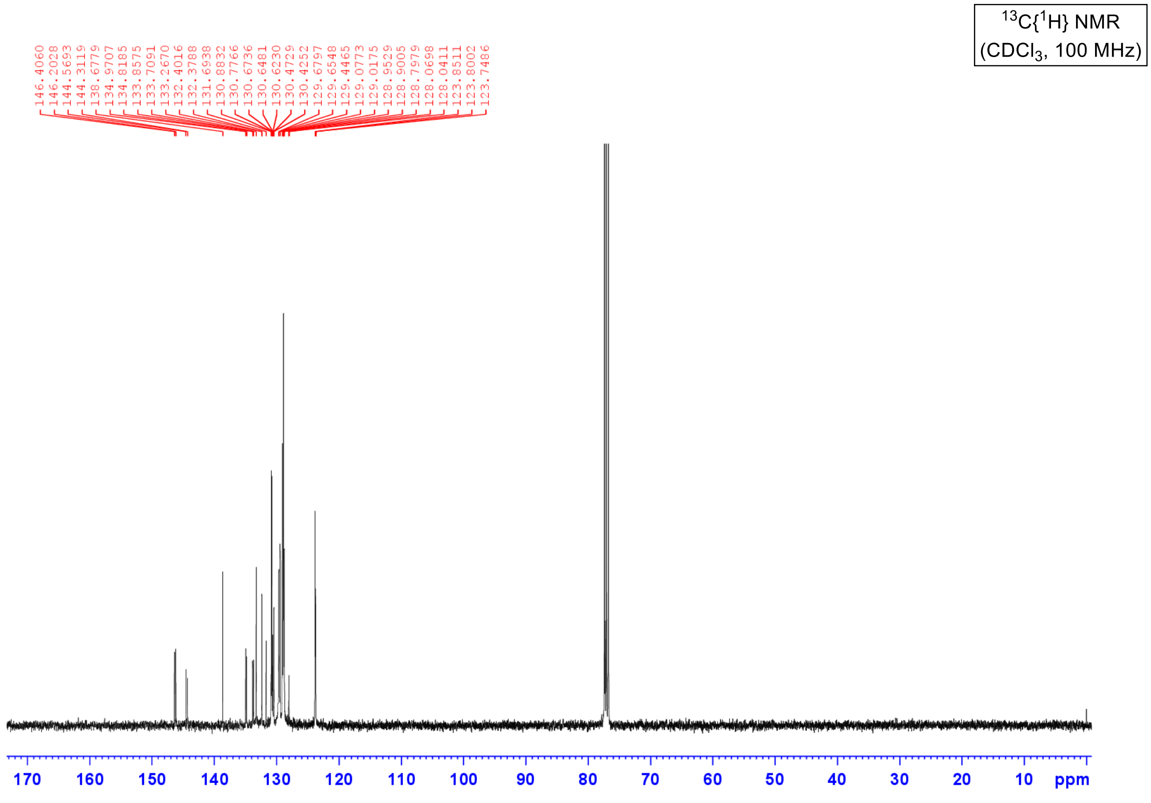

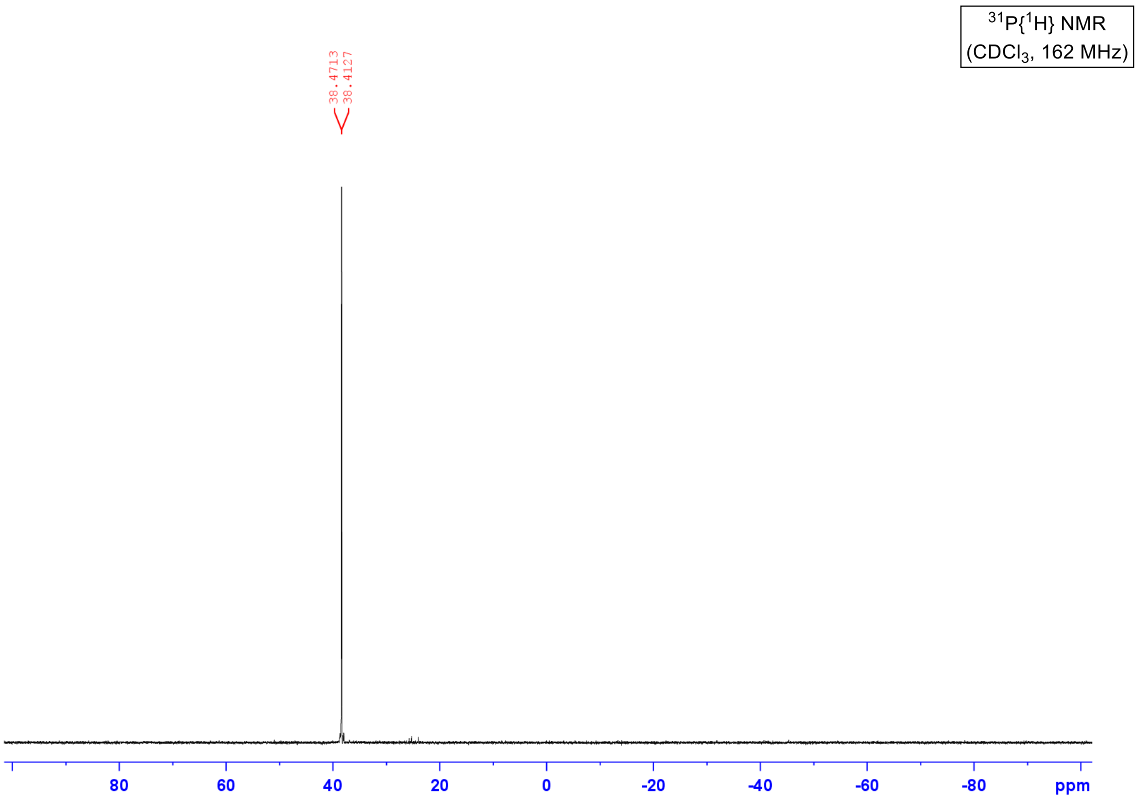
$^{31}P\{^1H\}$ NMR
($CDCl_3$, 162 MHz)
38.4713
38.4127
80
60
40
20
0
-20
-40
-60
-80
ppm

[$^{1}H$, $^{13}C\{^{1}H\}$, and $^{31}P\{^{1}H\}$ NMR Spectra of **4av**]

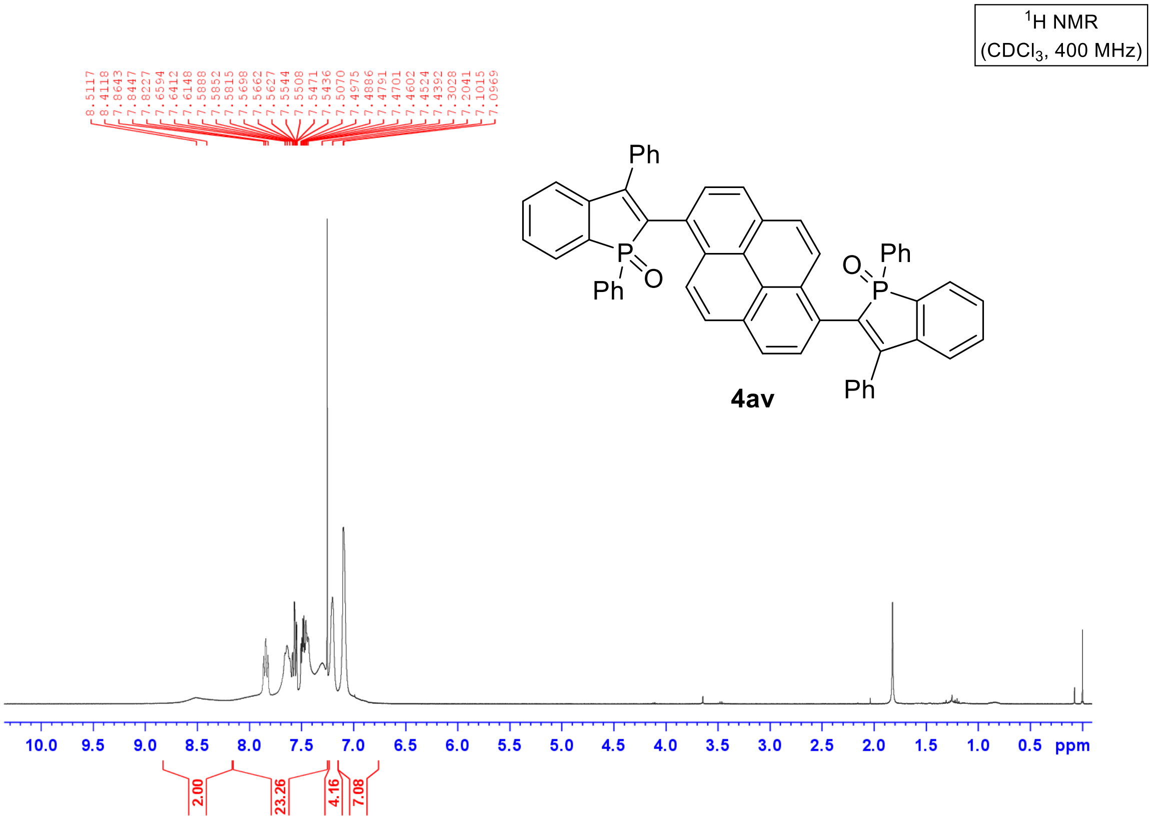


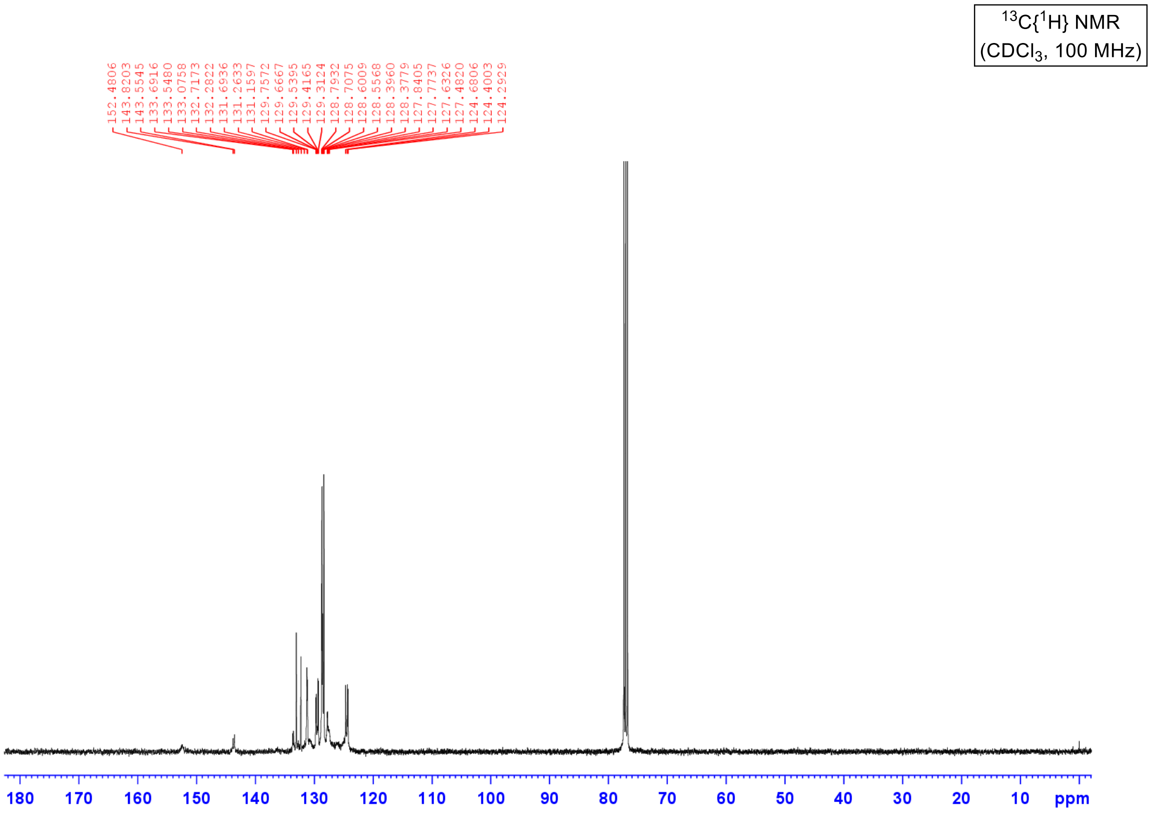

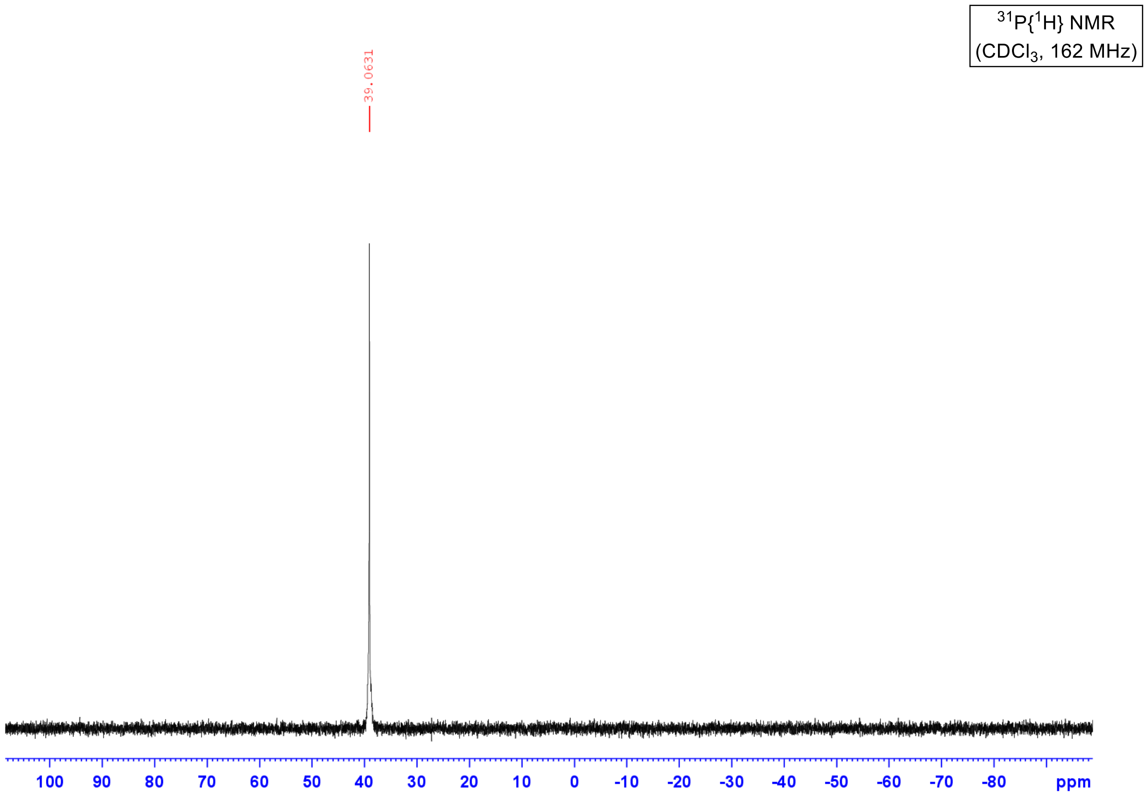
31P{1H} NMR
(CDCl3, 162 MHz)
39.0631
100
90
80
70
60
50
40
30
20
10
0
-10
-20
-30
-40
-50
-60
-70
-80
ppm

[$^{1}H$, $^{13}C\{^{1}H\}$, and $^{31}P\{^{1}H\}$ NMR Spectra of **4ba**]

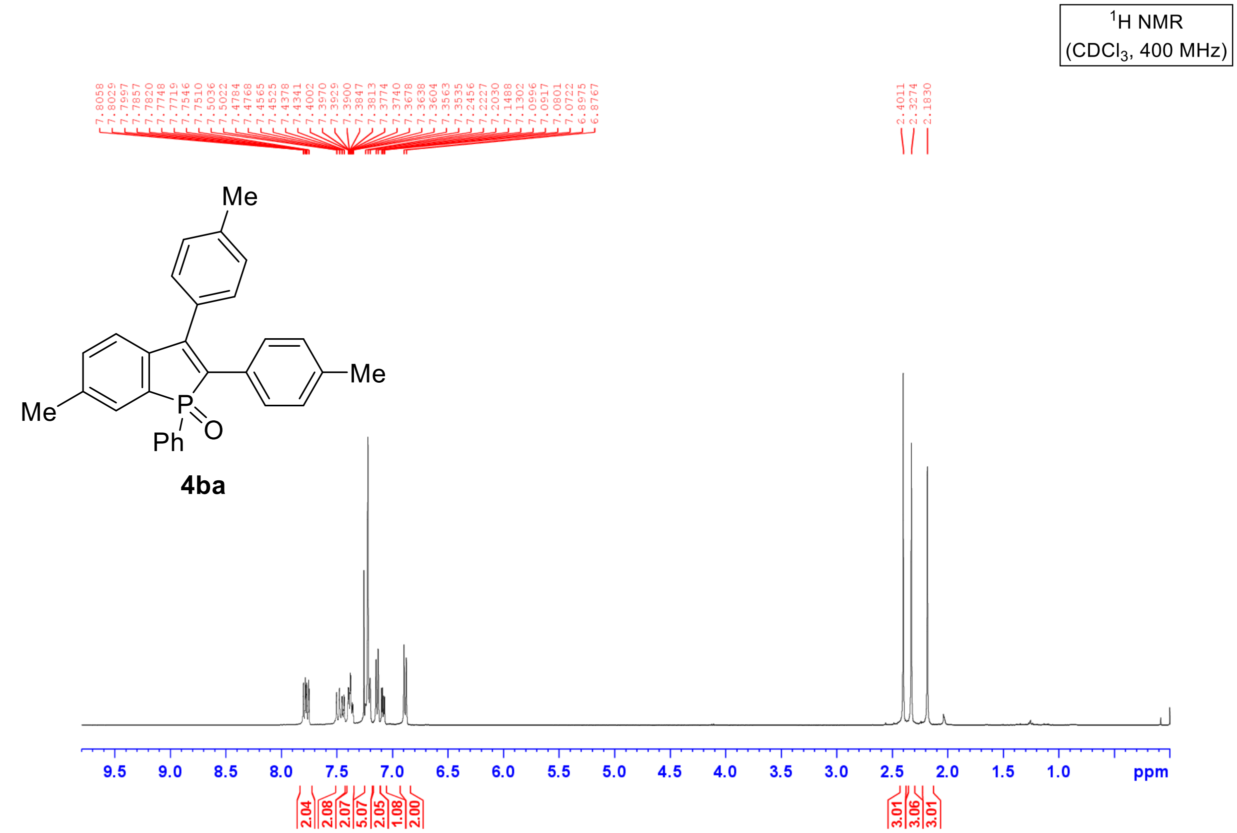


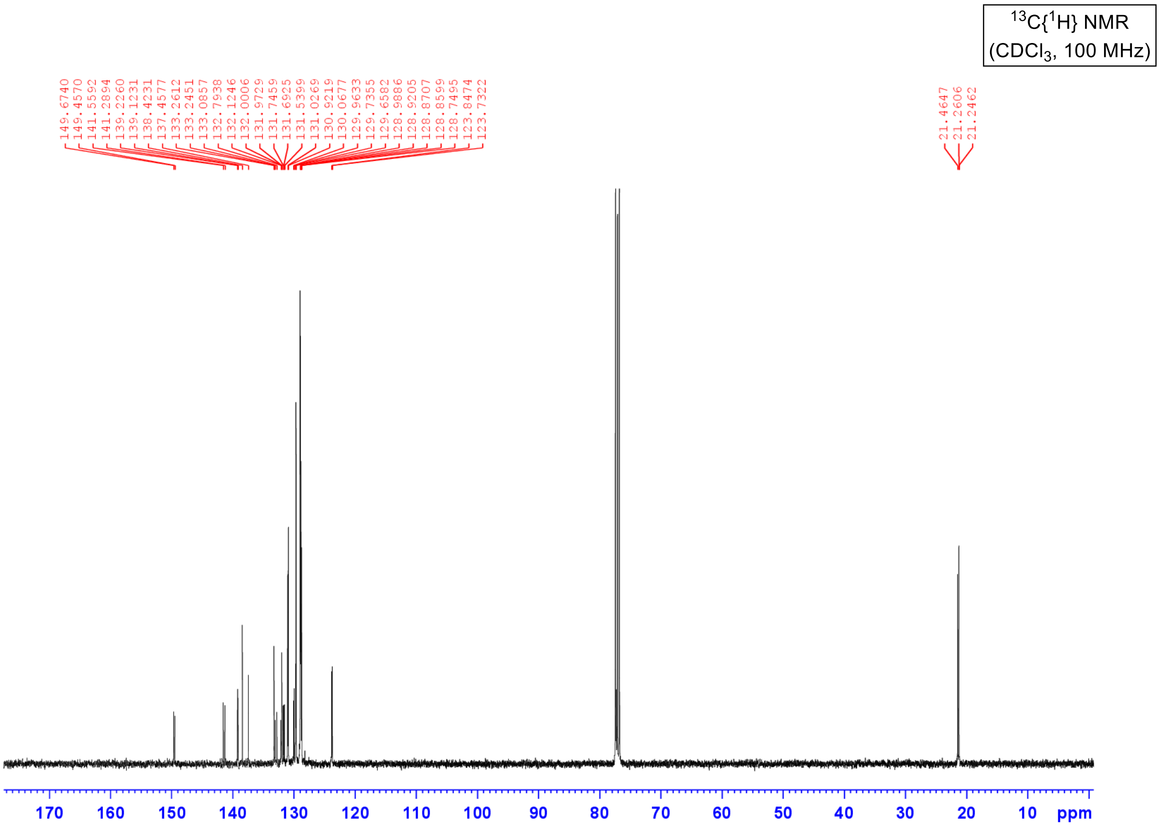

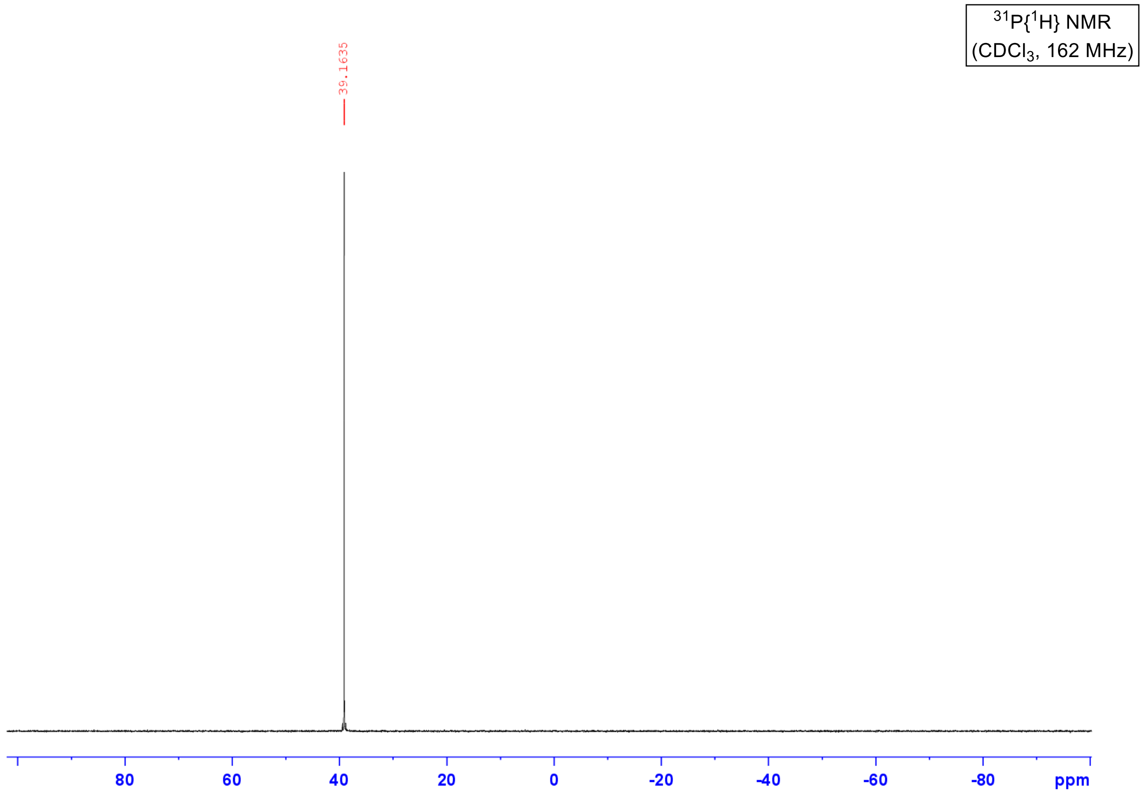
$^{31}P\{^1H\}$ NMR
($CDCl_3$, 162 MHz)
39.1635
80
60
40
20
0
-20
-40
-60
-80
ppm

[$^1$H, $^{13}$C{$^1$H}, and $^{31}$P{$^1$H} NMR Spectra of **4da**]

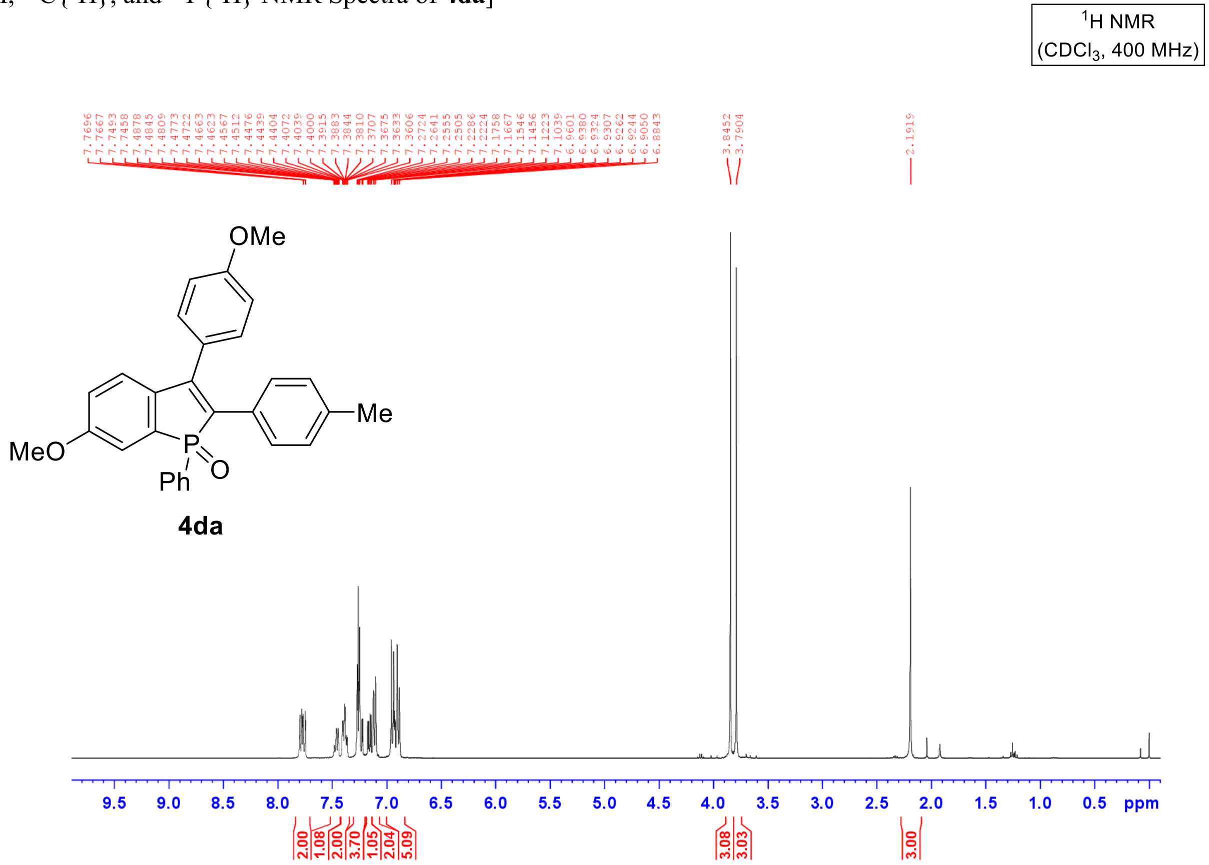


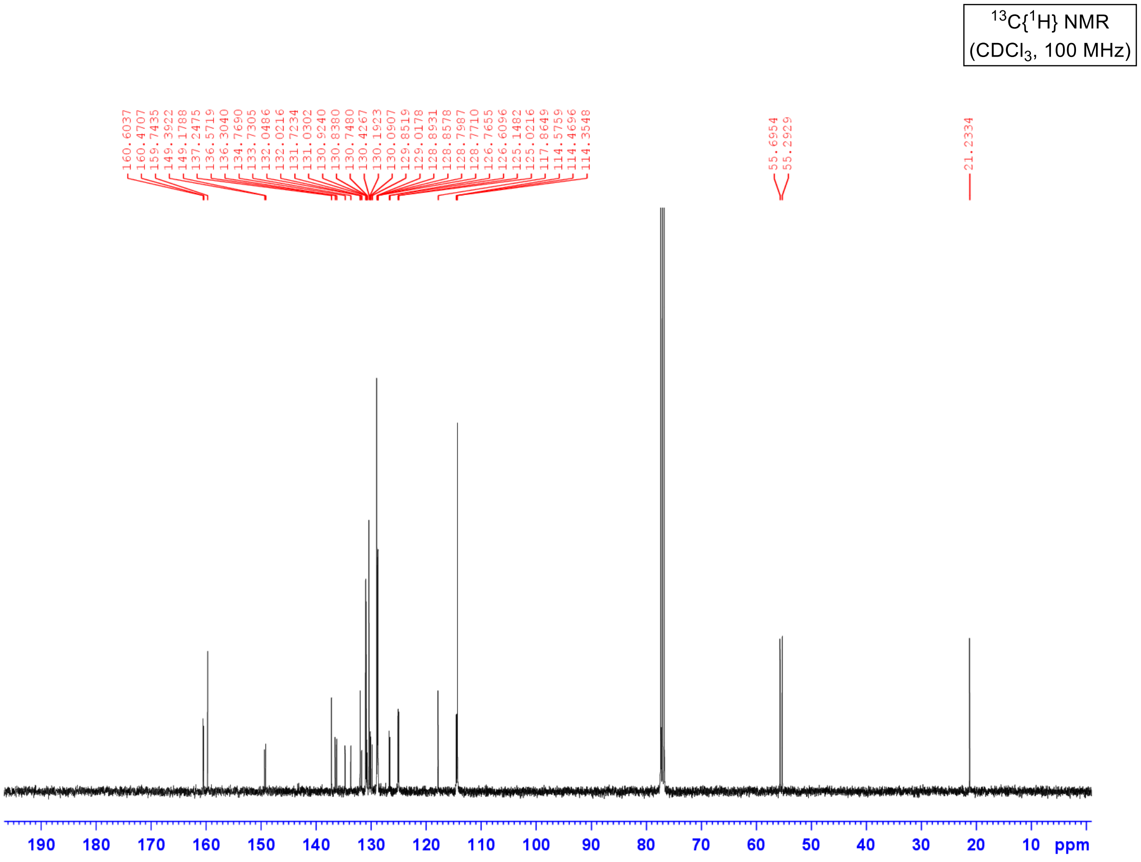

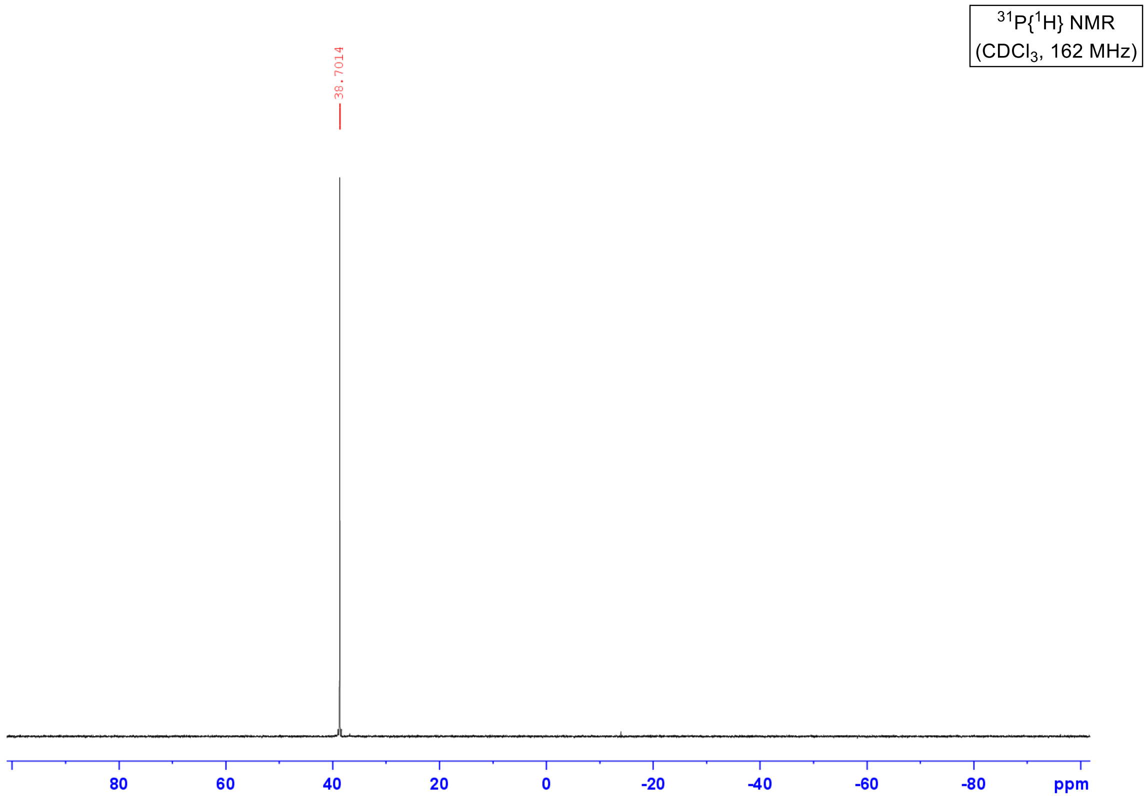
$^{31}P\{^1H\}$ NMR
($CDCl_3$, 162 MHz)
38.7014
80
60
40
20
0
-20
-40
-60
-80
ppm

[$^{1}H$, $^{13}C\{^{1}H\}$, $^{19}F\{^{1}H\}$, and $^{31}P\{^{1}H\}$ NMR Spectra of **4ea**]

$^{1}H$ NMR
($CDCl_3$, 400 MHz)

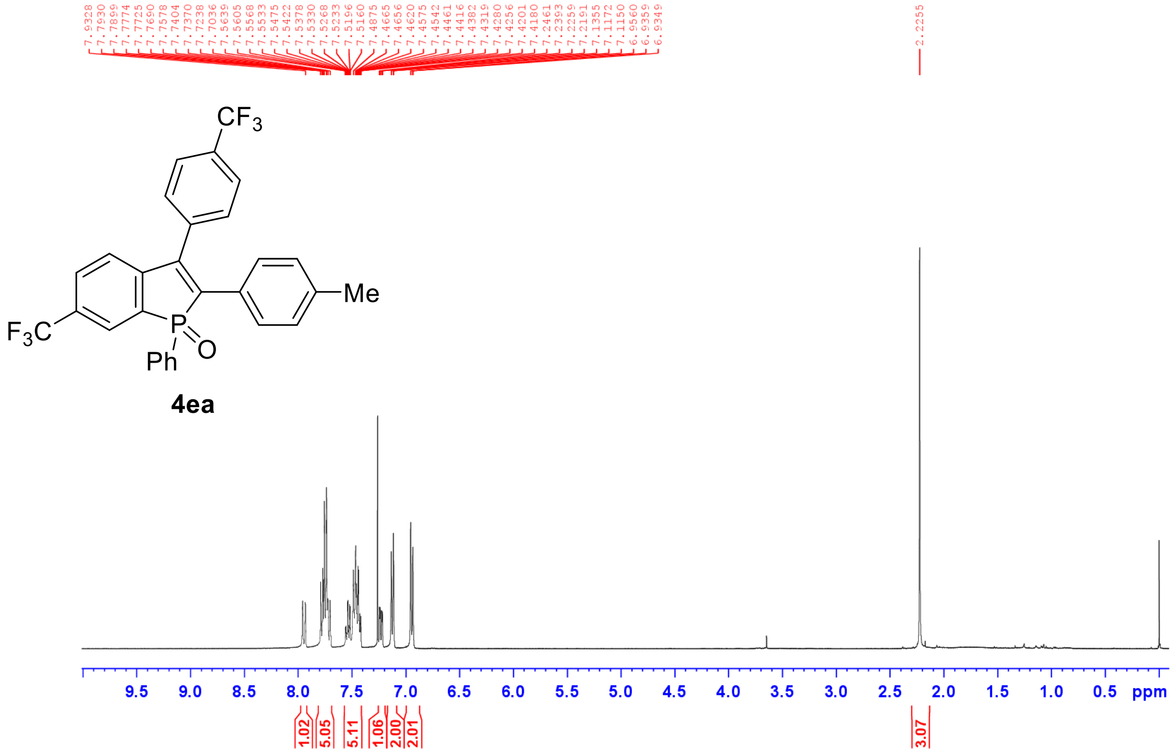


$^{13}C\{^{1}H\}$ NMR
($CDCl_3$, 100 MHz)

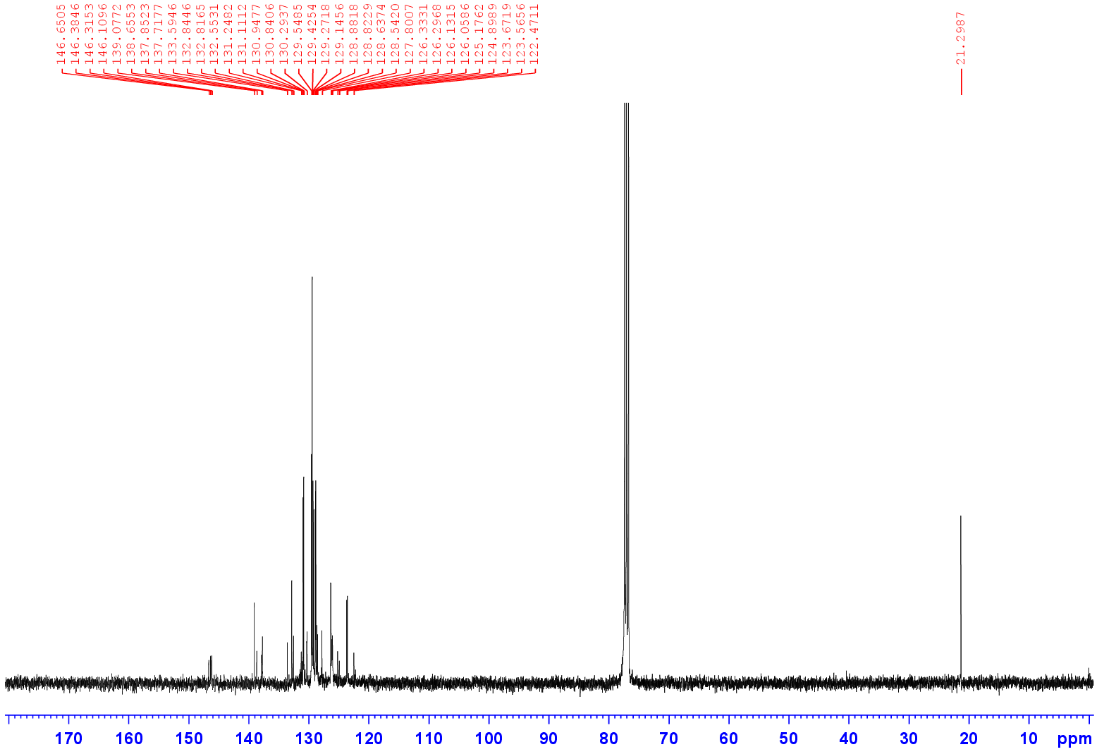

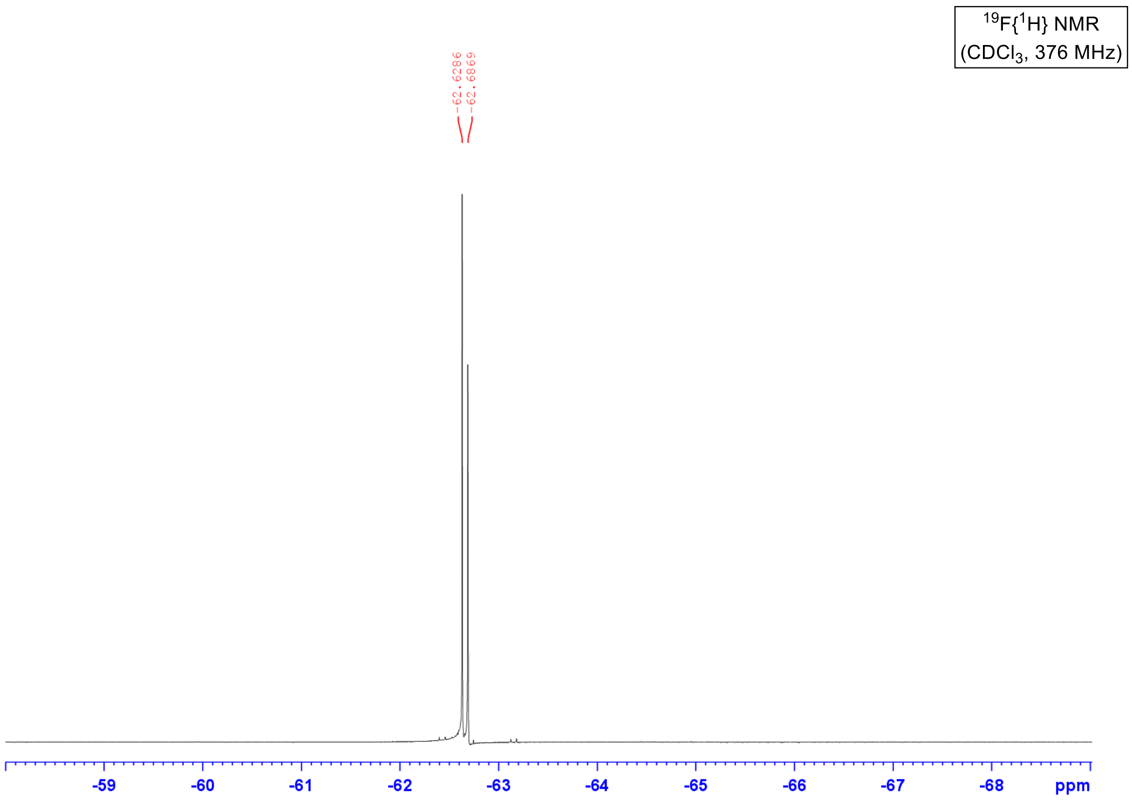

$^{19}F\{^1H\}$ NMR
($CDCl_3$, 376 MHz)
-62.6286
-62.6869
-59
-60
-61
-62
-63
-64
-65
-66
-67
-68
ppm


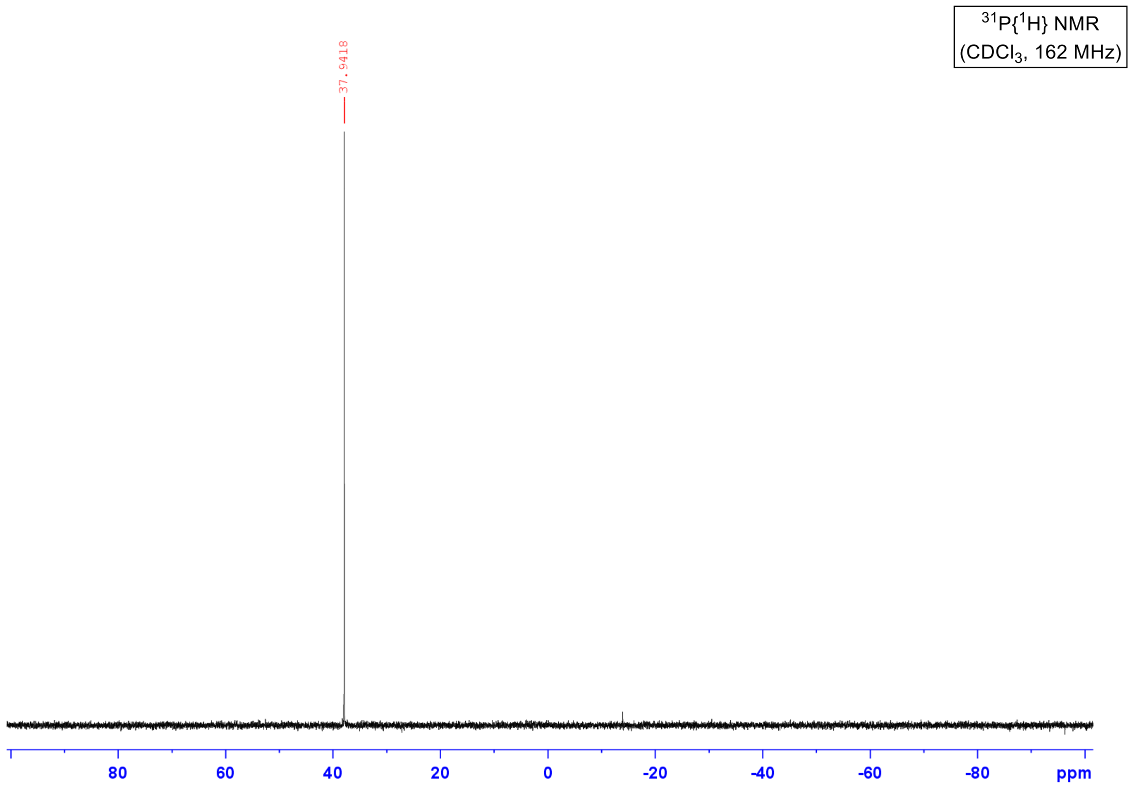

$^{31}P\{^1H\}$ NMR
($CDCl_3$, 162 MHz)
37.9418
80
60
40
20
0
-20
-40
-60
-80
ppm

[$^{1}H$, $^{13}C\{^{1}H\}$, $^{19}F\{^{1}H\}$, and $^{31}P\{^{1}H\}$ NMR Spectra of **4fa**]

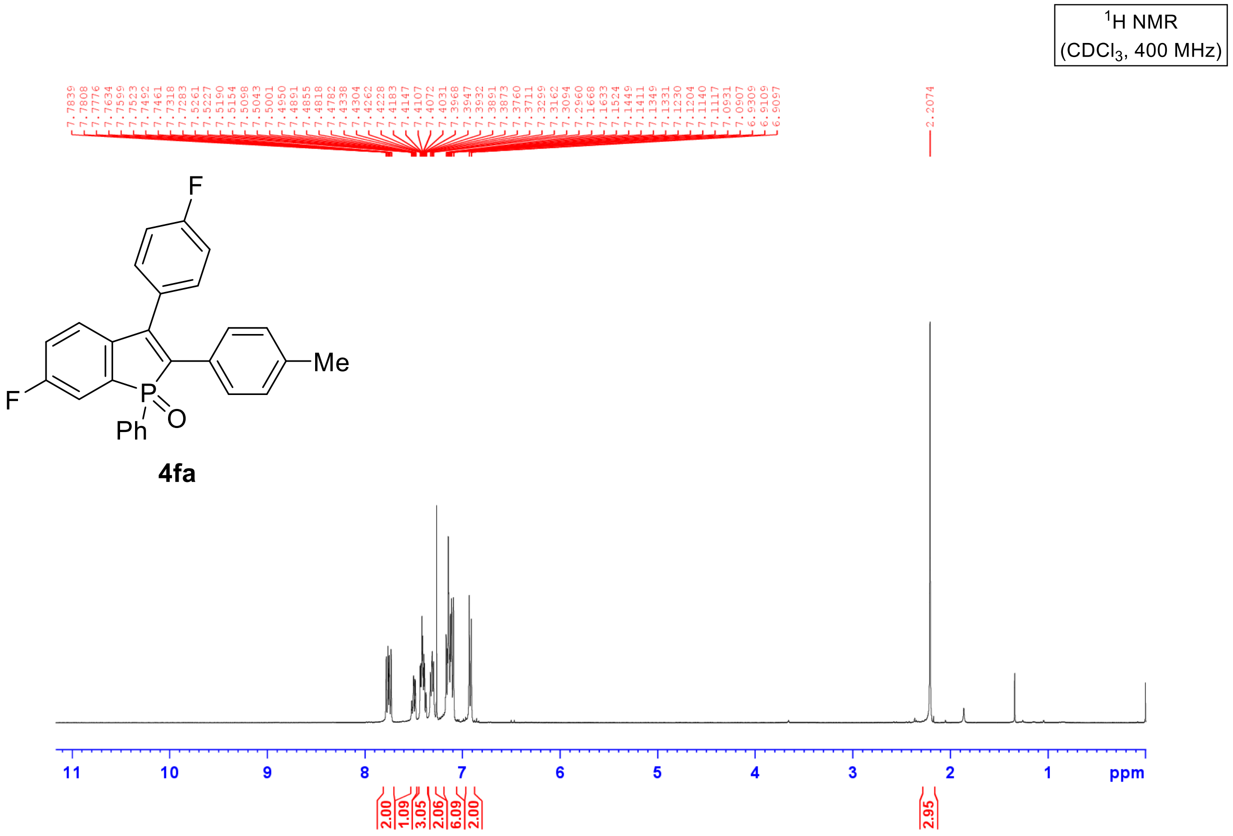


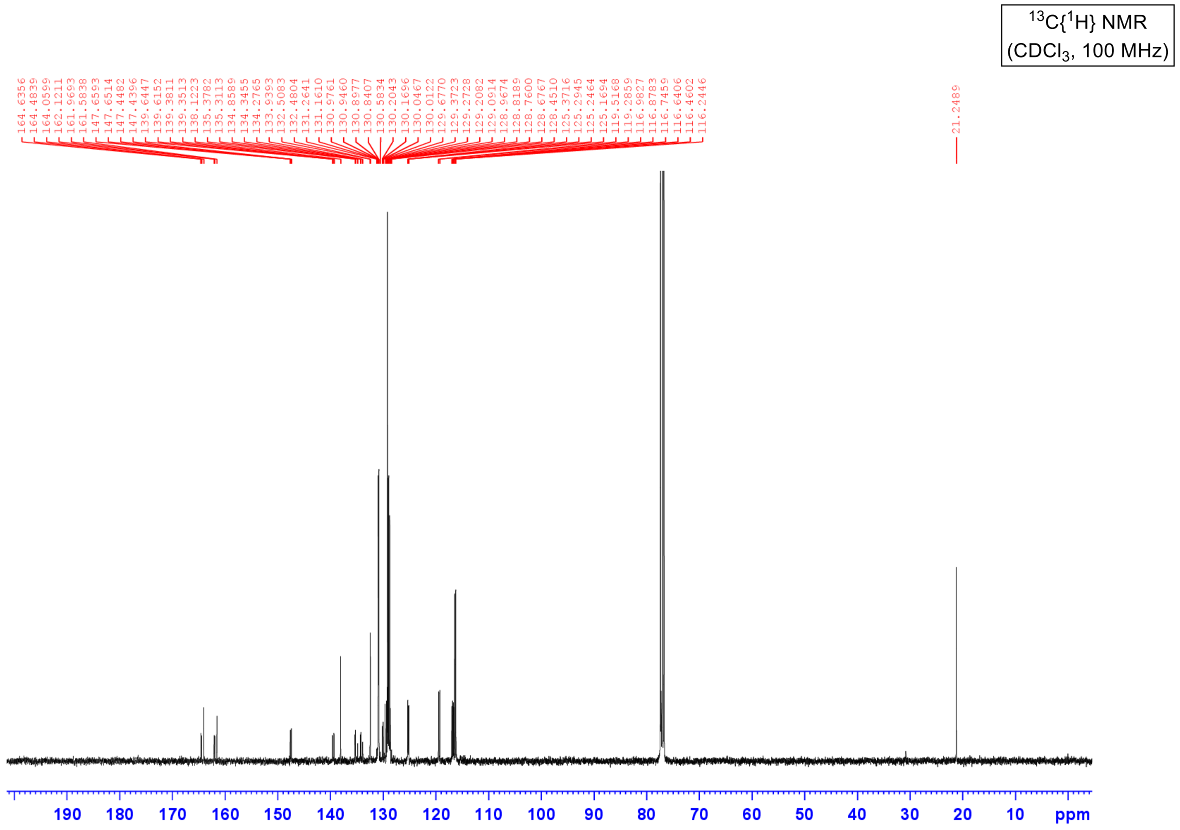

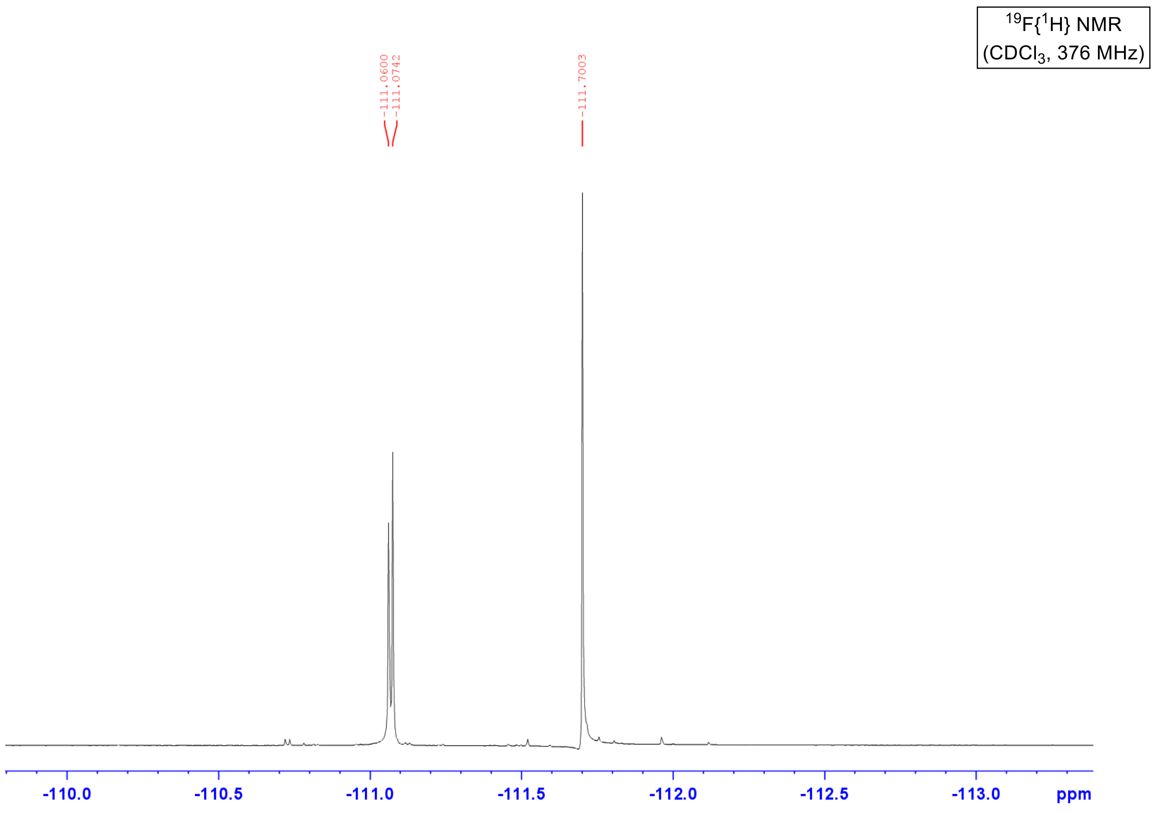
$^{19}F\{^1H\}$ NMR
($CDCl_3$, 376 MHz)
-111.0600
-111.0742
-111.7003
-110.0
-110.5
-111.0
-111.5
-112.0
-112.5
-113.0
ppm

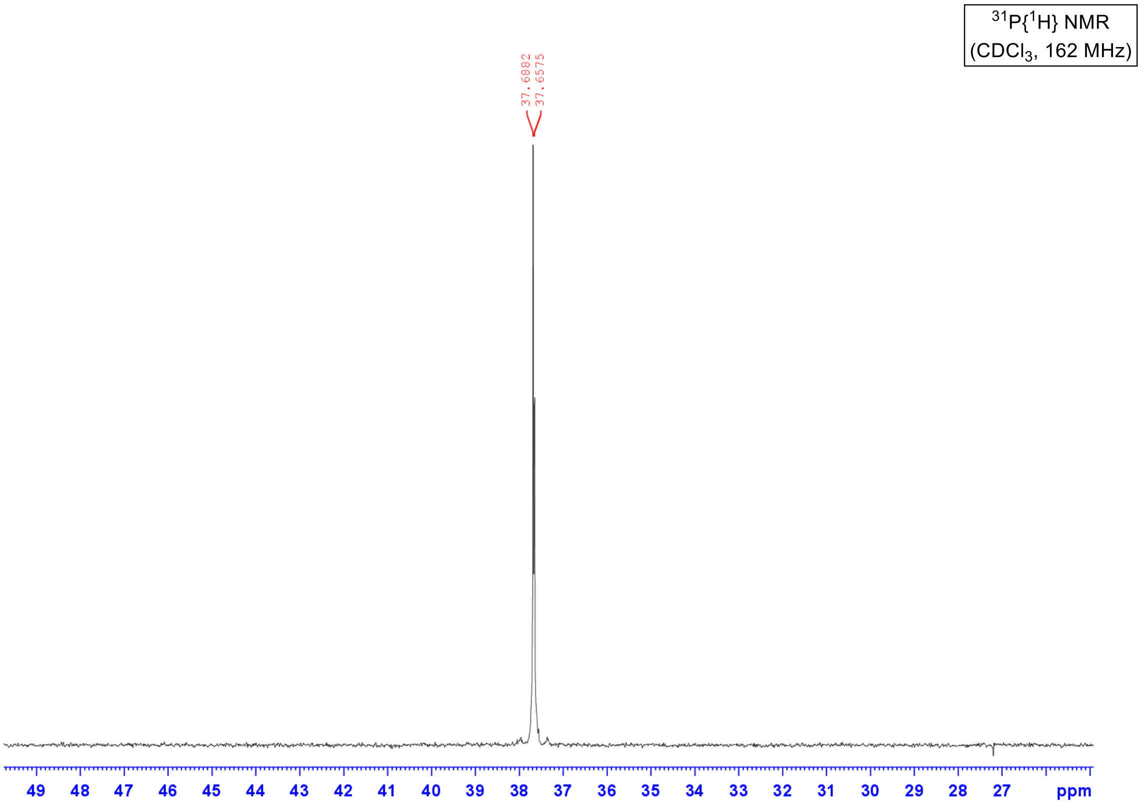
$^{31}P\{^1H\}$ NMR
($CDCl_3$, 162 MHz)
37.6882
37.6575
49
48
47
46
45
44
43
42
41
40
39
38
37
36
35
34
33
32
31
30
29
28
27
ppm

[$^{1}$H, $^{13}$C{$^{1}$H}, and $^{31}$P{$^{1}$H} NMR Spectra of **4ga**]

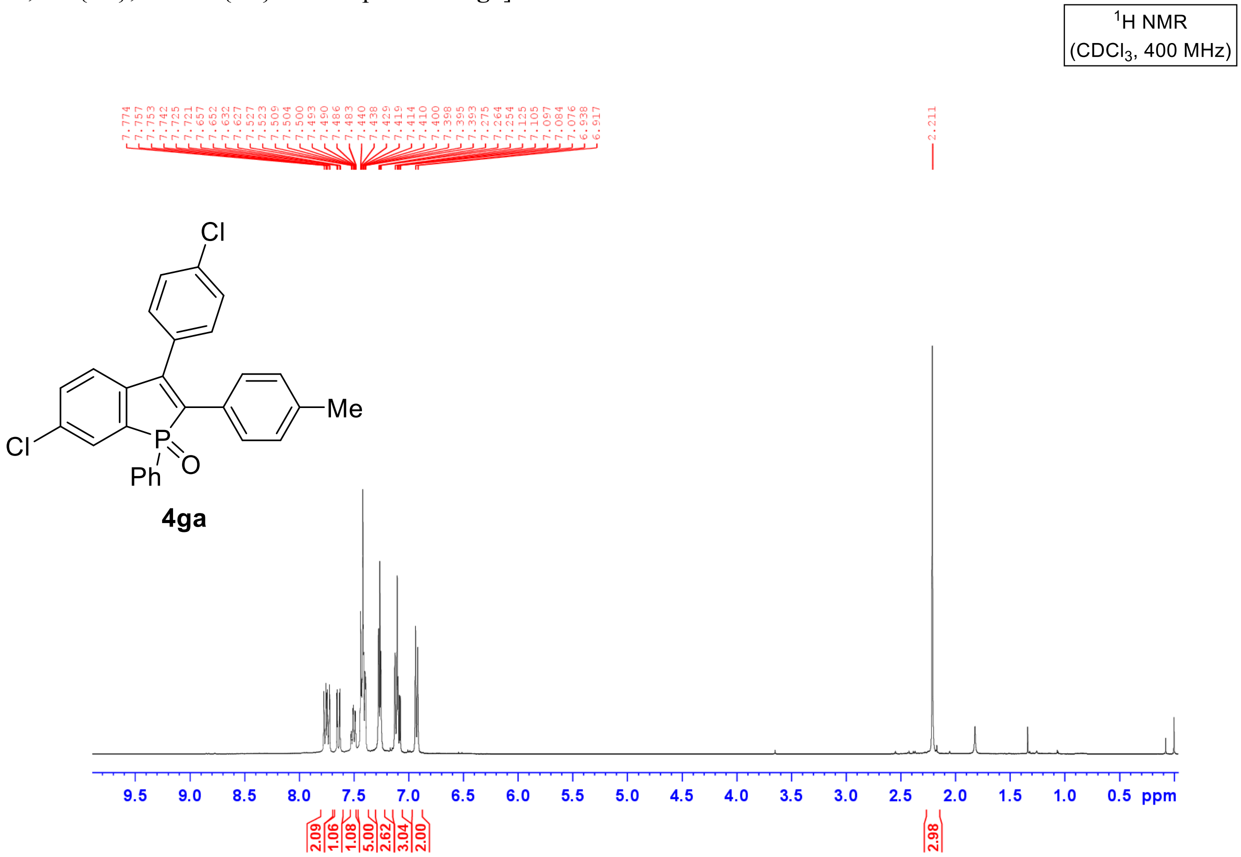


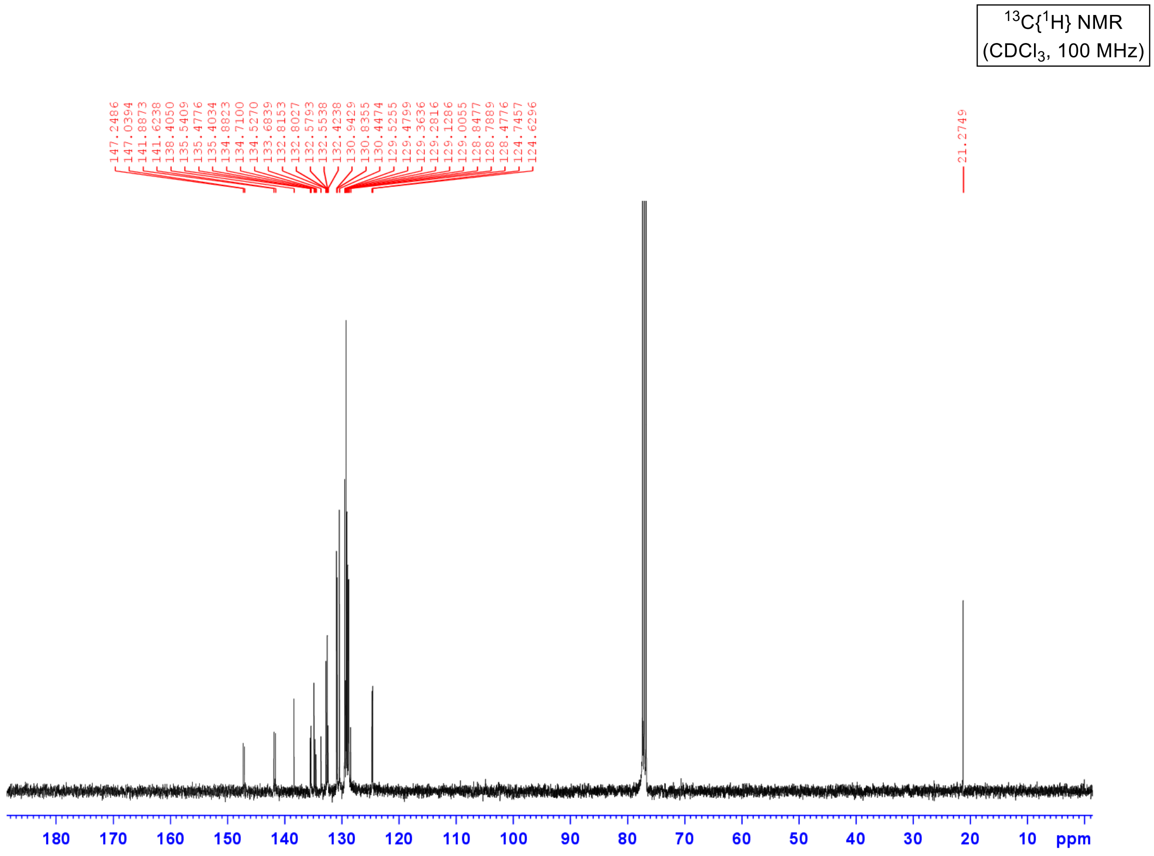

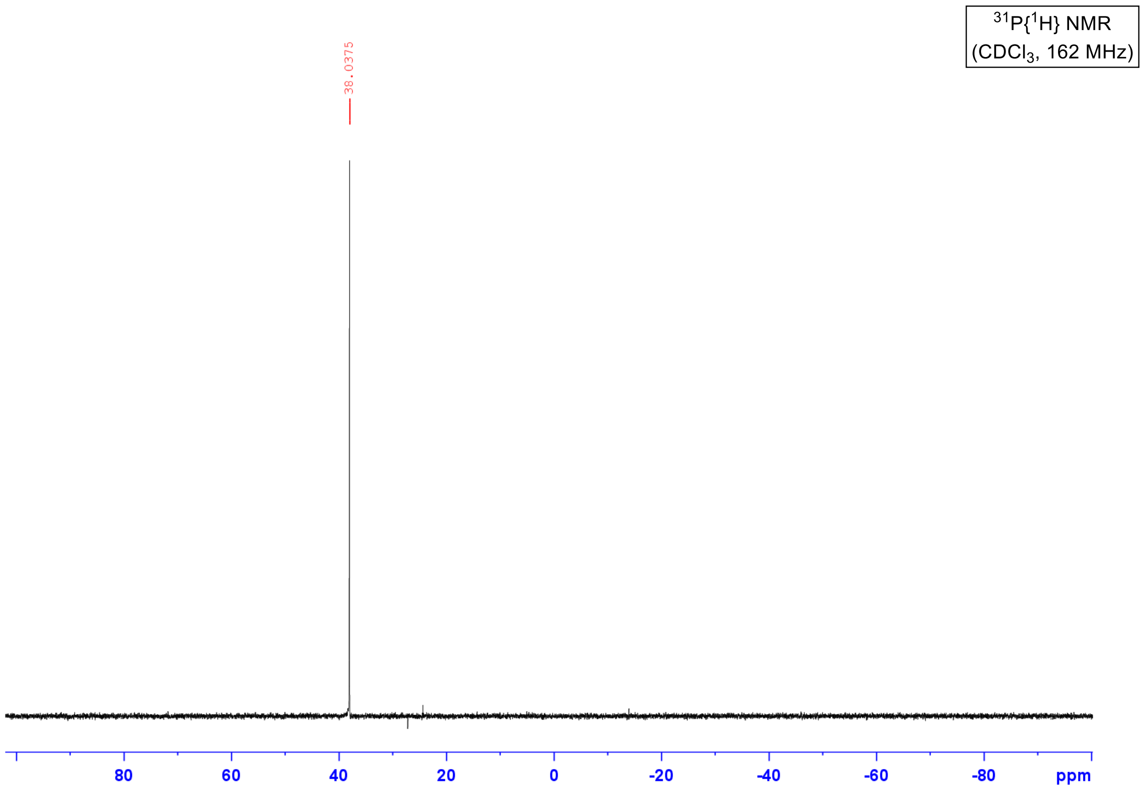
$^{31}P\{^1H\}$ NMR
($CDCl_3$, 162 MHz)
38.0375
80
60
40
20
0
-20
-40
-60
-80
ppm

[$^{1}H$, $^{13}C\{^{1}H\}$, and $^{31}P\{^{1}H\}$ NMR Spectra of **4ha**]

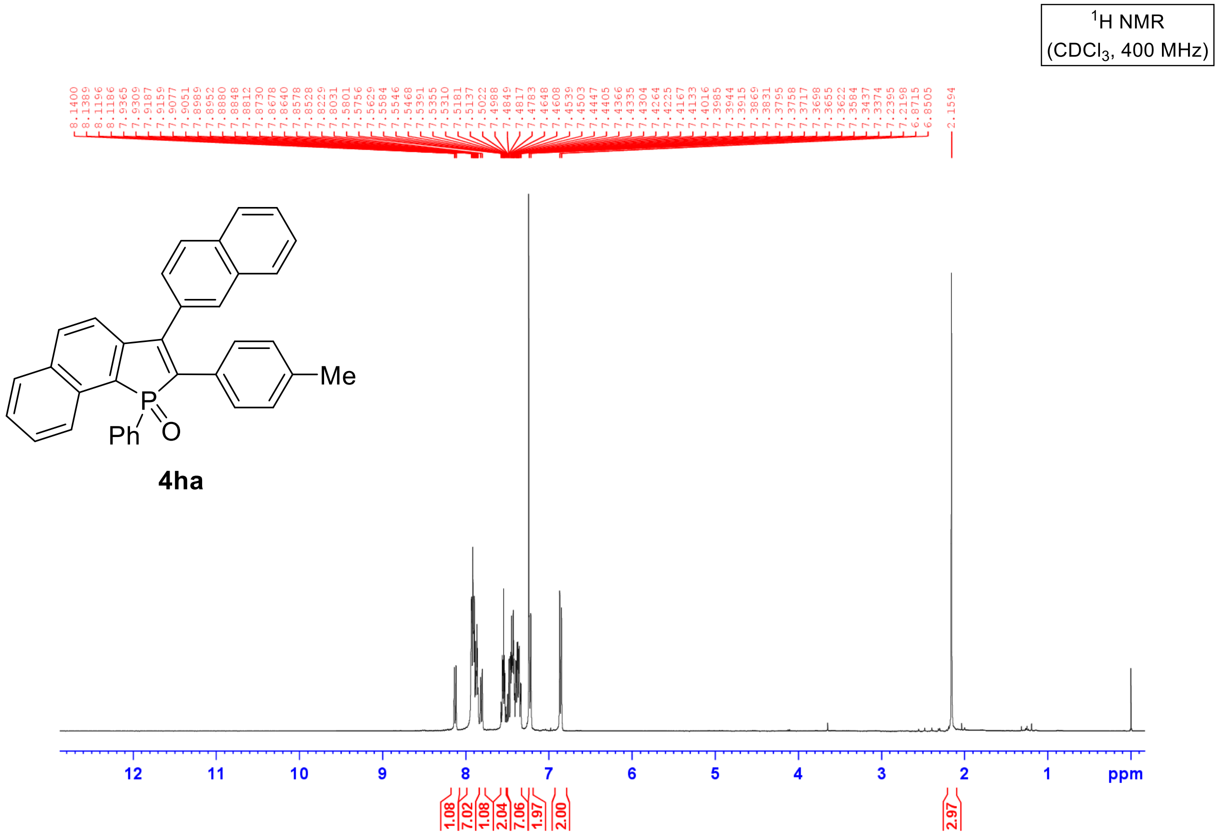


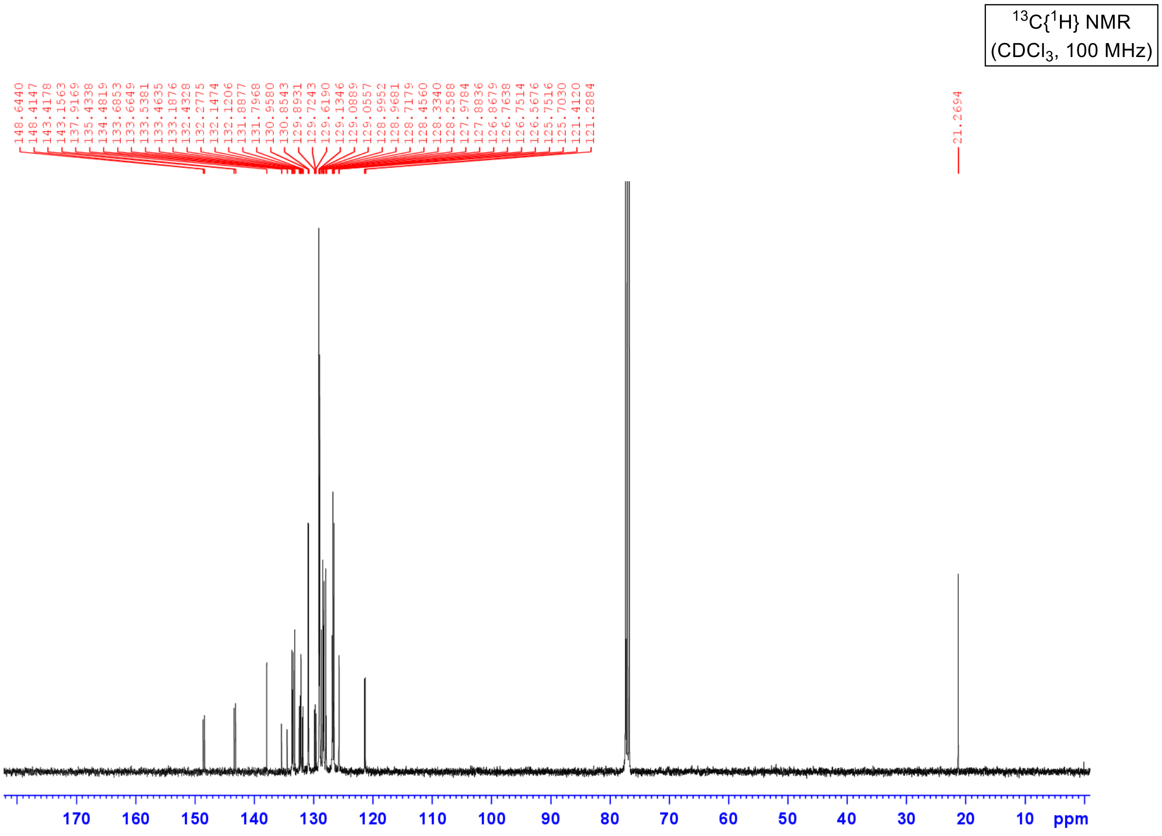

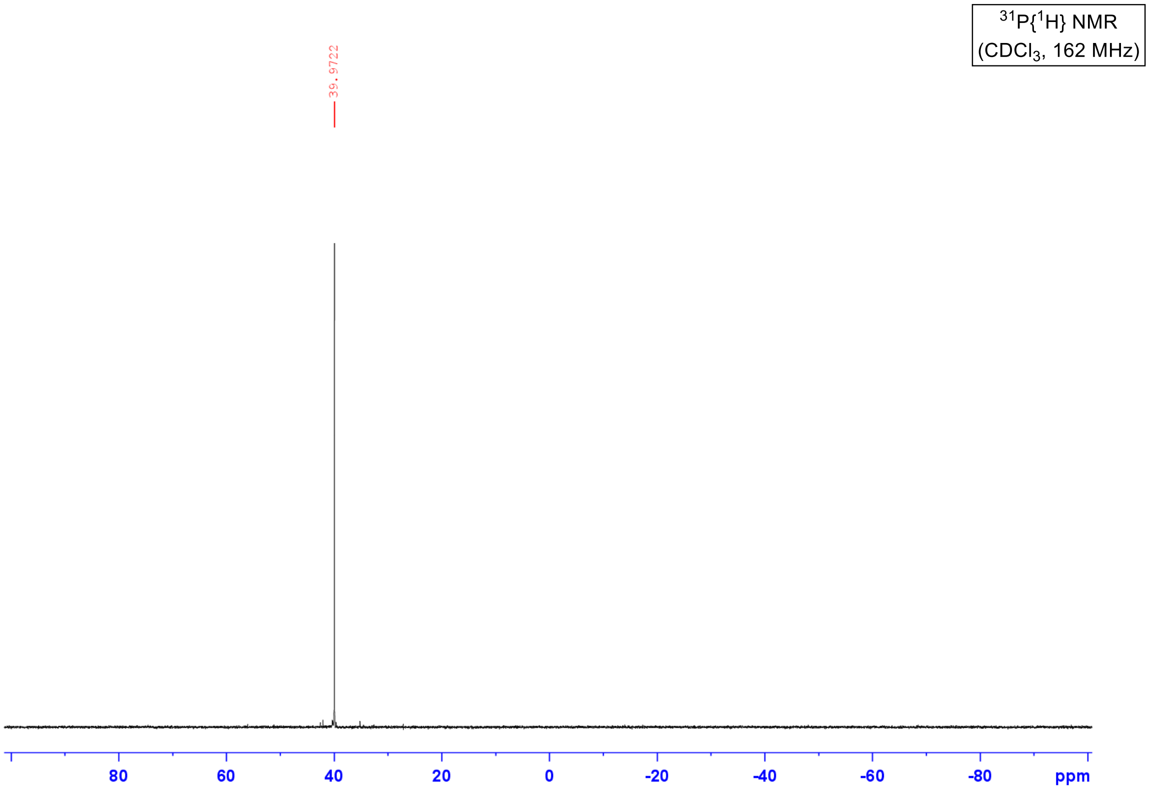
$^{31}P\{^1H\}$ NMR
($CDCl_3$, 162 MHz)
39.9722
80
60
40
20
0
-20
-40
-60
-80
ppm

[$^{1}H$, $^{13}C\{^{1}H\}$, and $^{31}P\{^{1}H\}$ NMR Spectra of **4ia**]

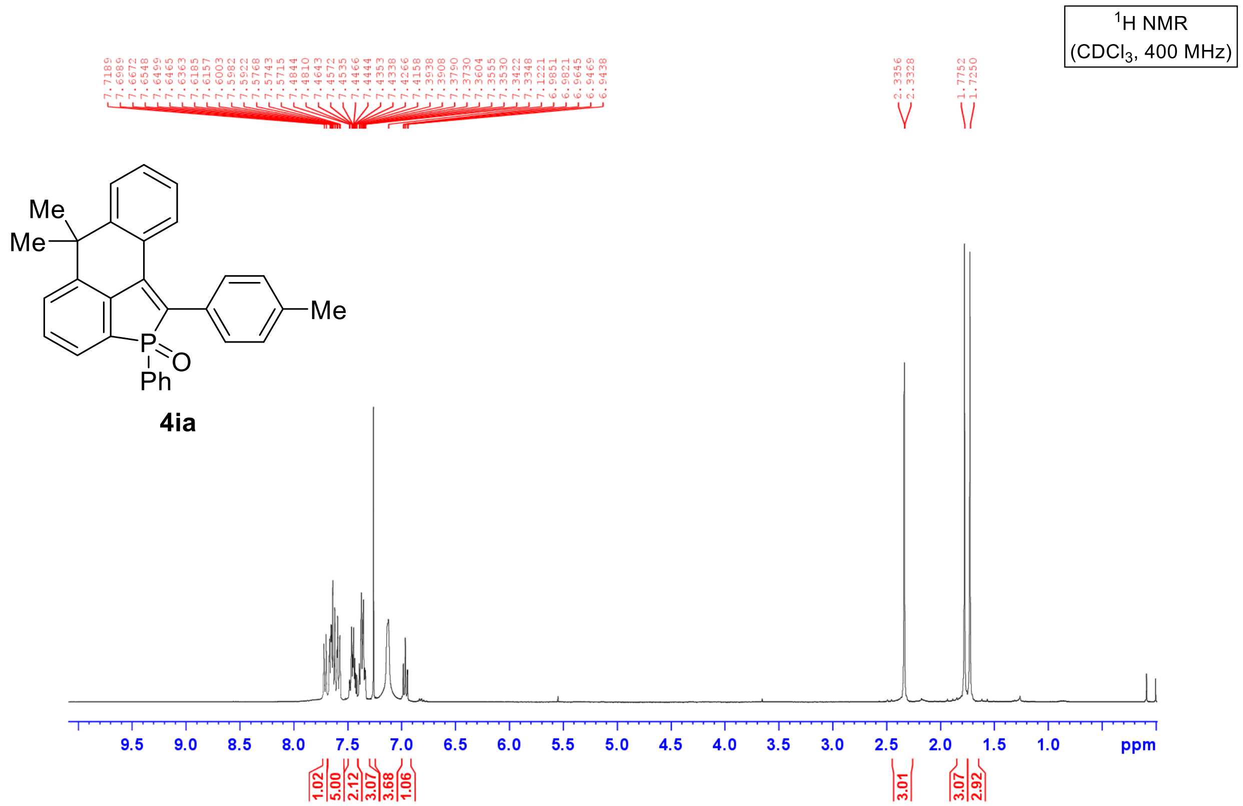

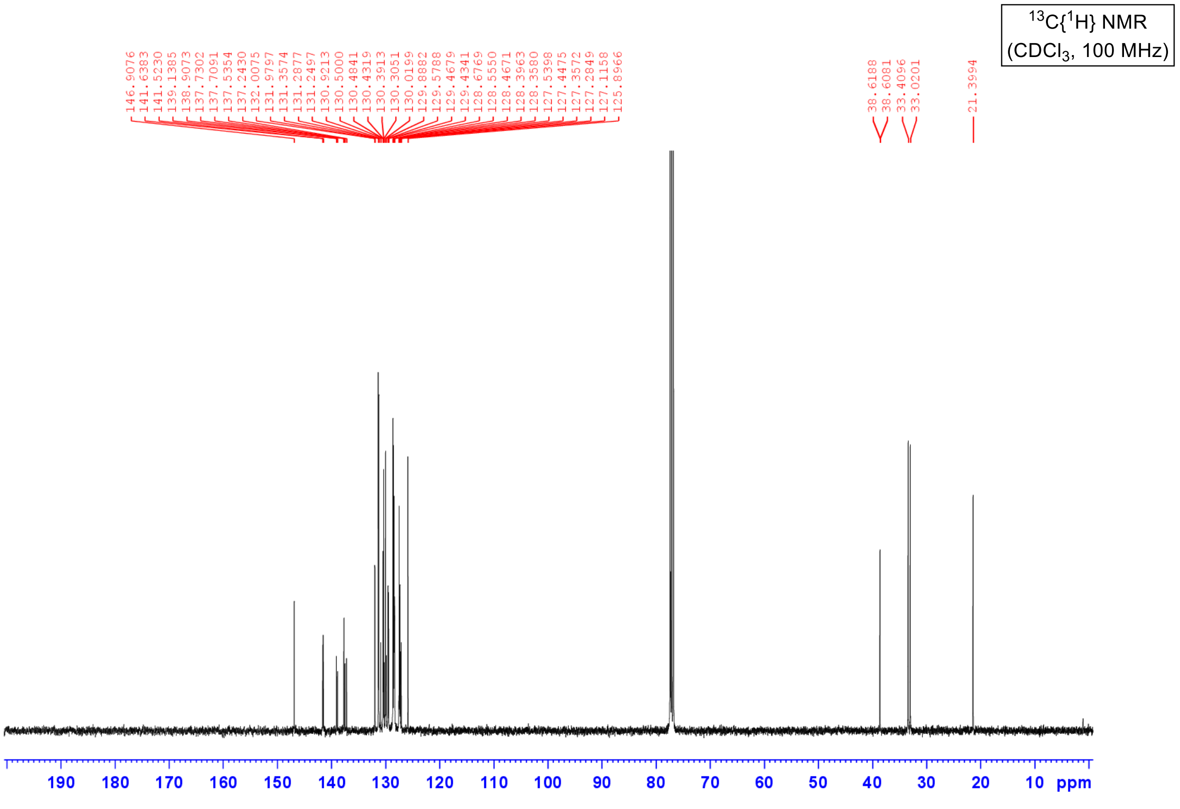

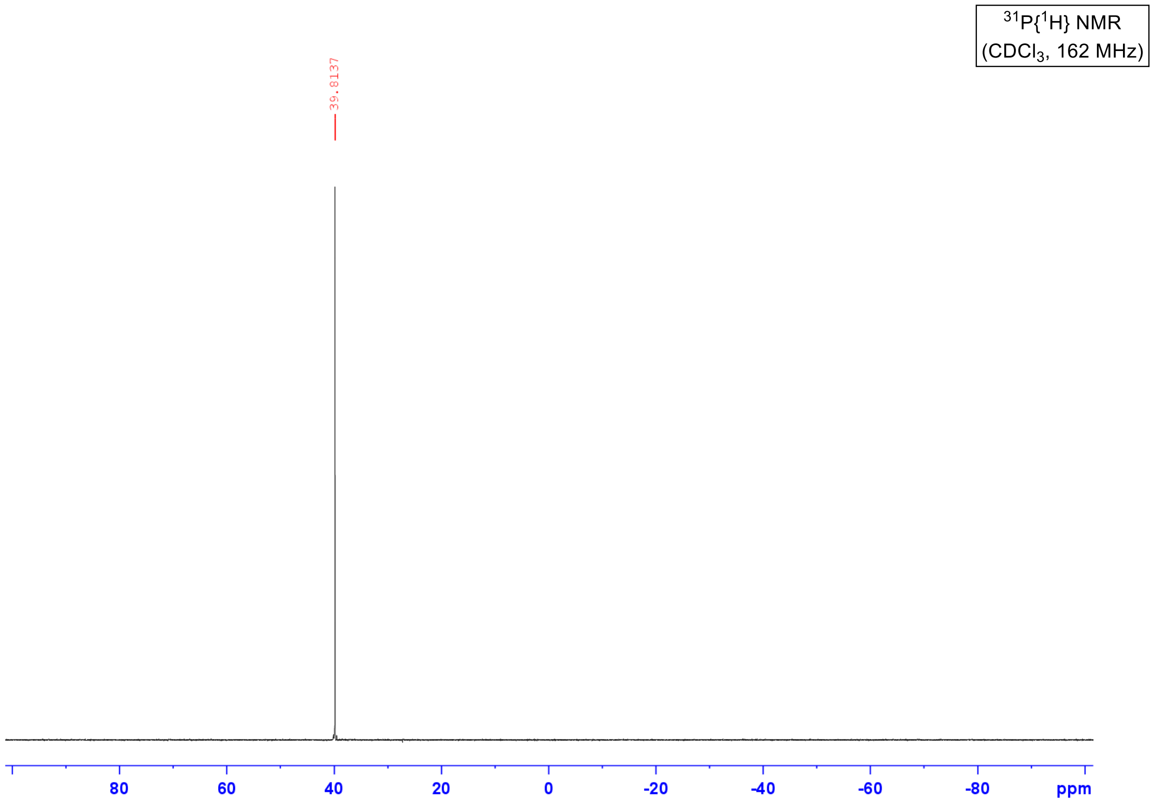

$^{31}P\{^{1}H\}$ NMR
($CDCl_3$, 162 MHz)
39.8137
80
60
40
20
0
-20
-40
-60
-80
ppm

[$^{1}H$, $^{13}C\{^{1}H\}$, and $^{31}P\{^{1}H\}$ NMR Spectra of **4ja**]

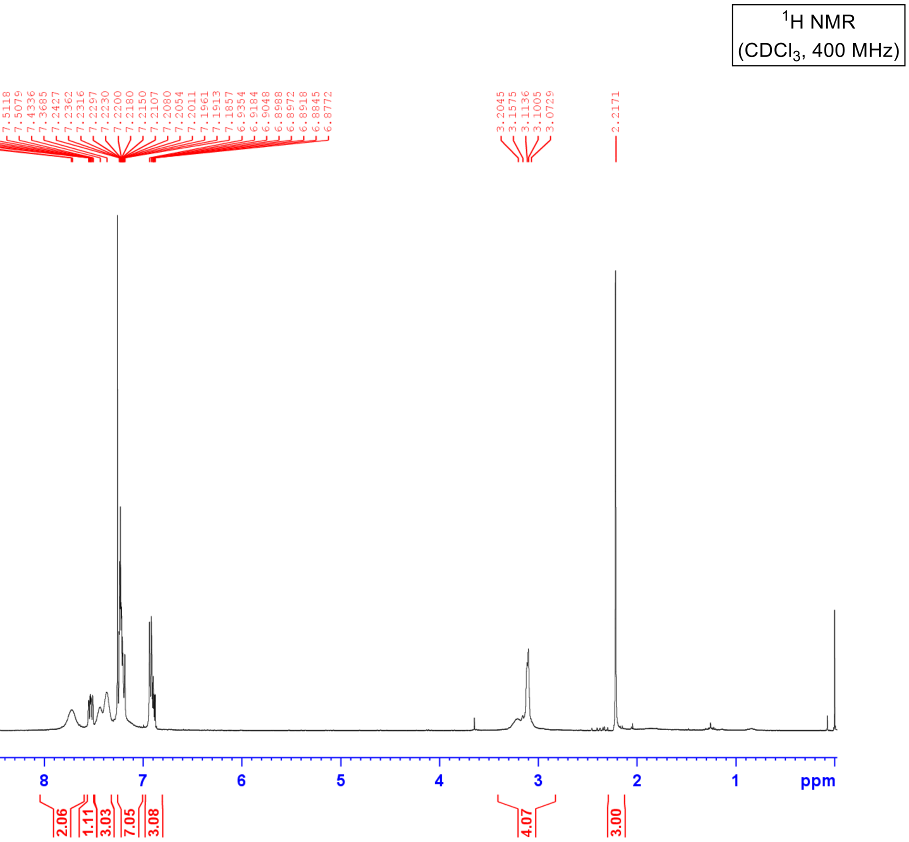


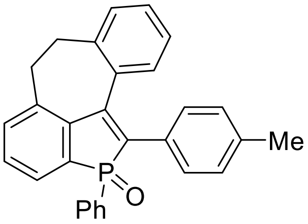


**4ja**

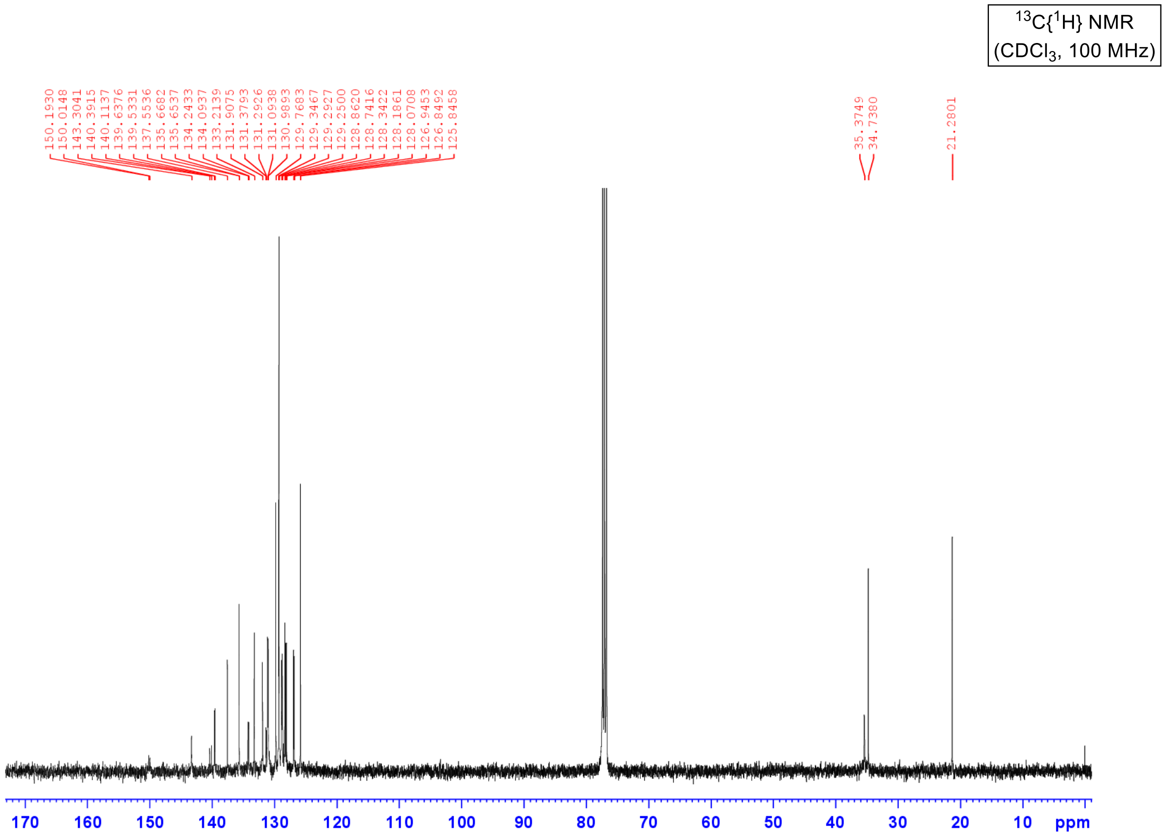

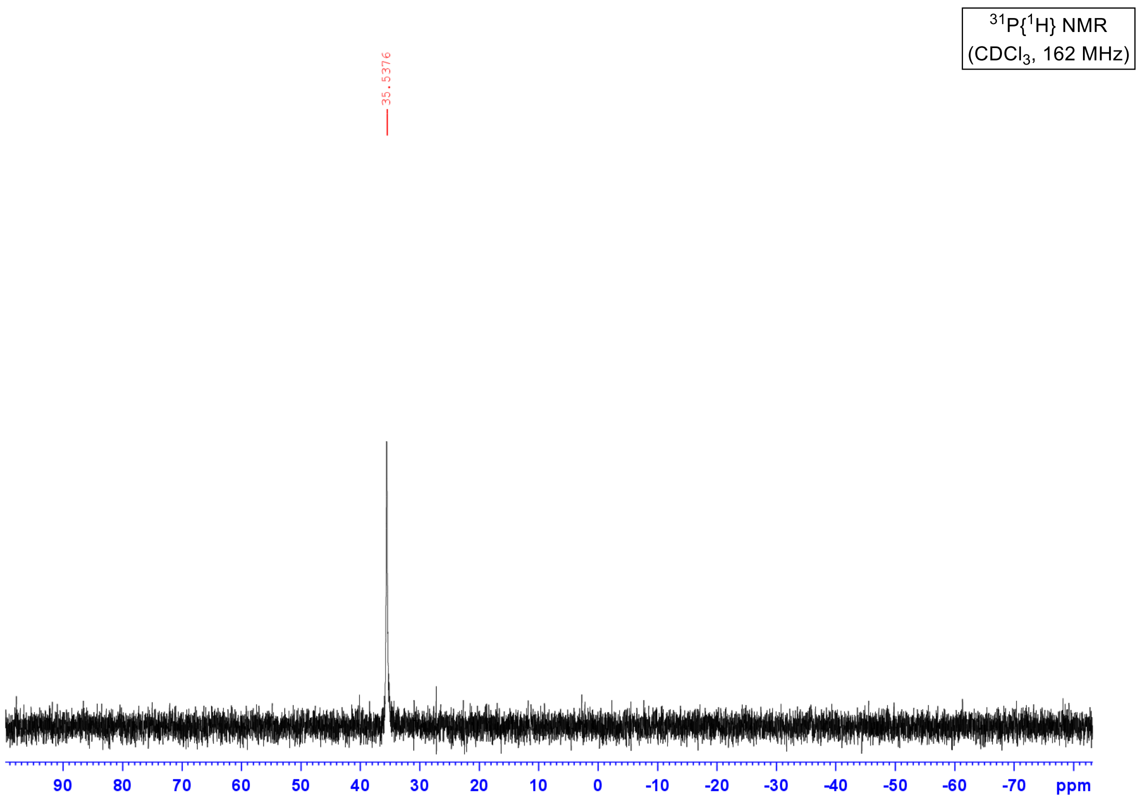
$^{31}P\{^1H\}$ NMR
($CDCl_3$, 162 MHz)
35.5376
90
80
70
60
50
40
30
20
10
0
-10
-20
-30
-40
-50
-60
-70
ppm

[$^{1}$H, $^{13}$C{$^{1}$H}, and $^{31}$P{$^{1}$H} NMR Spectra of **5aa**]

$^{1}$H NMR
(CDCl$_3$, 400 MHz)

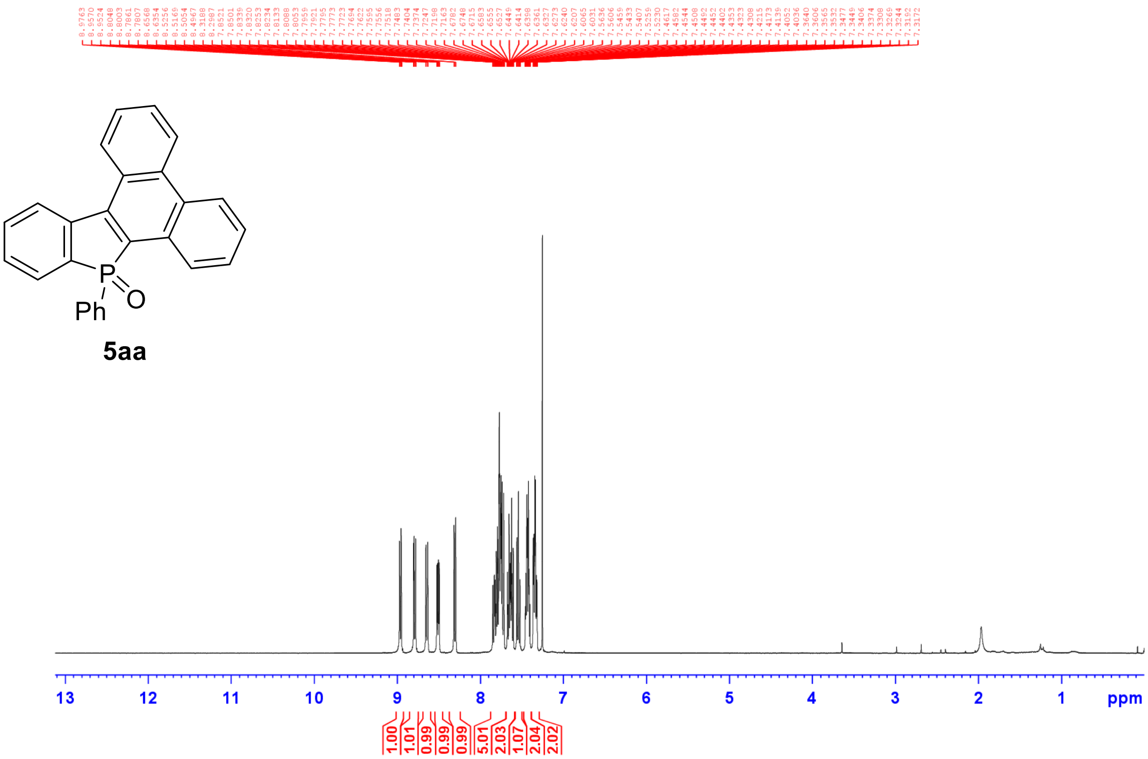


$^{13}$C{$^{1}$H} NMR
(CDCl$_3$, 100 MHz)

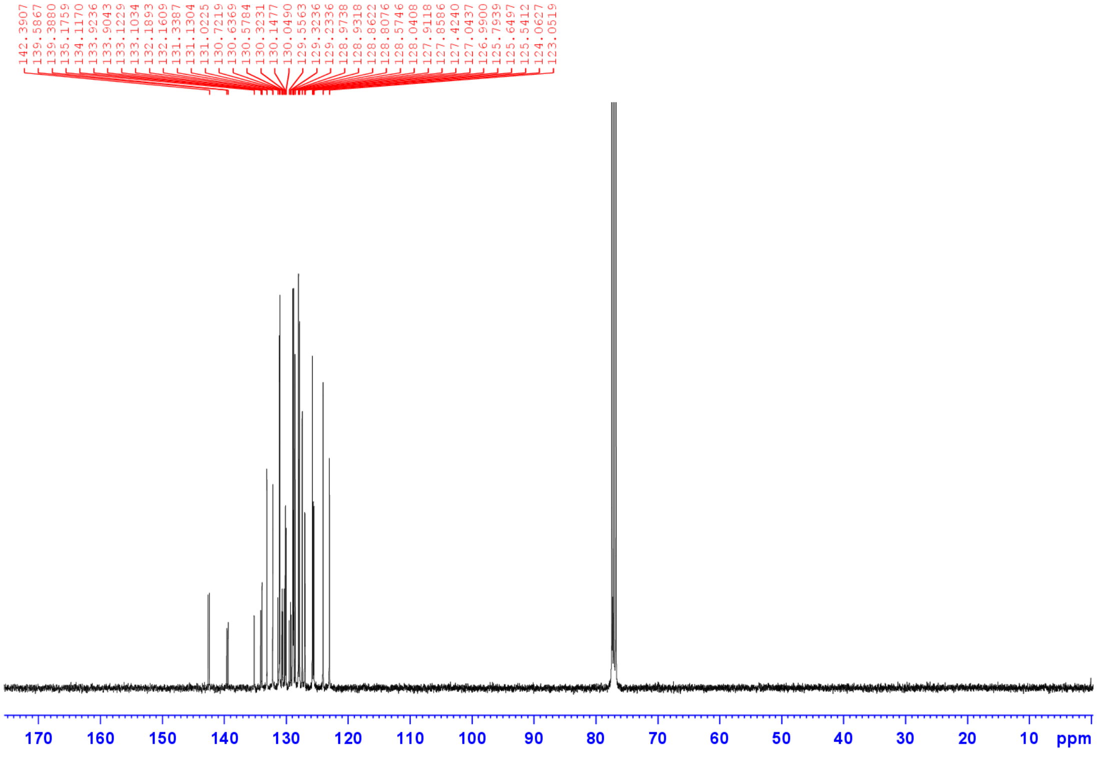

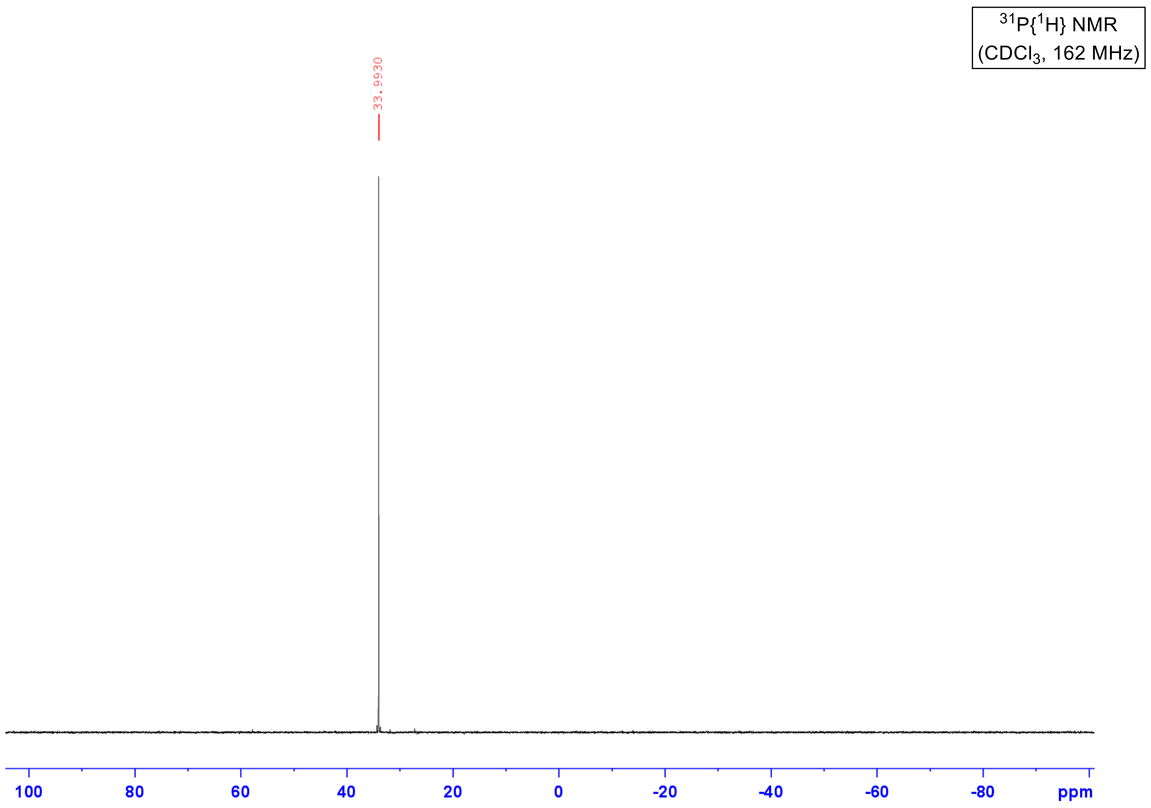
$^{31}P\{^{1}H\}$ NMR
($CDCl_3$, 162 MHz)
33.9930
100
80
60
40
20
0
-20
-40
-60
-80
ppm

[$^{1}$H, $^{13}$C{$^{1}$H}, and $^{31}$P{$^{1}$H} NMR Spectra of **5ab**]

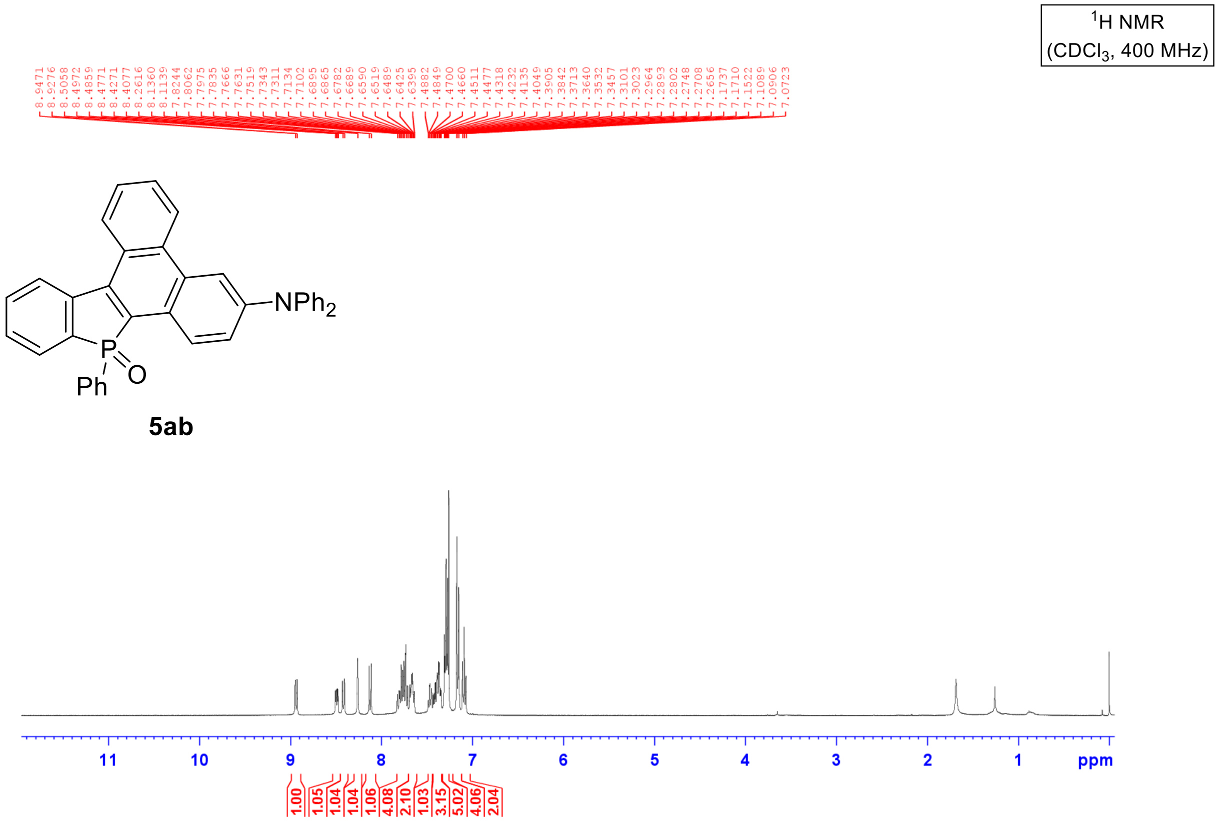


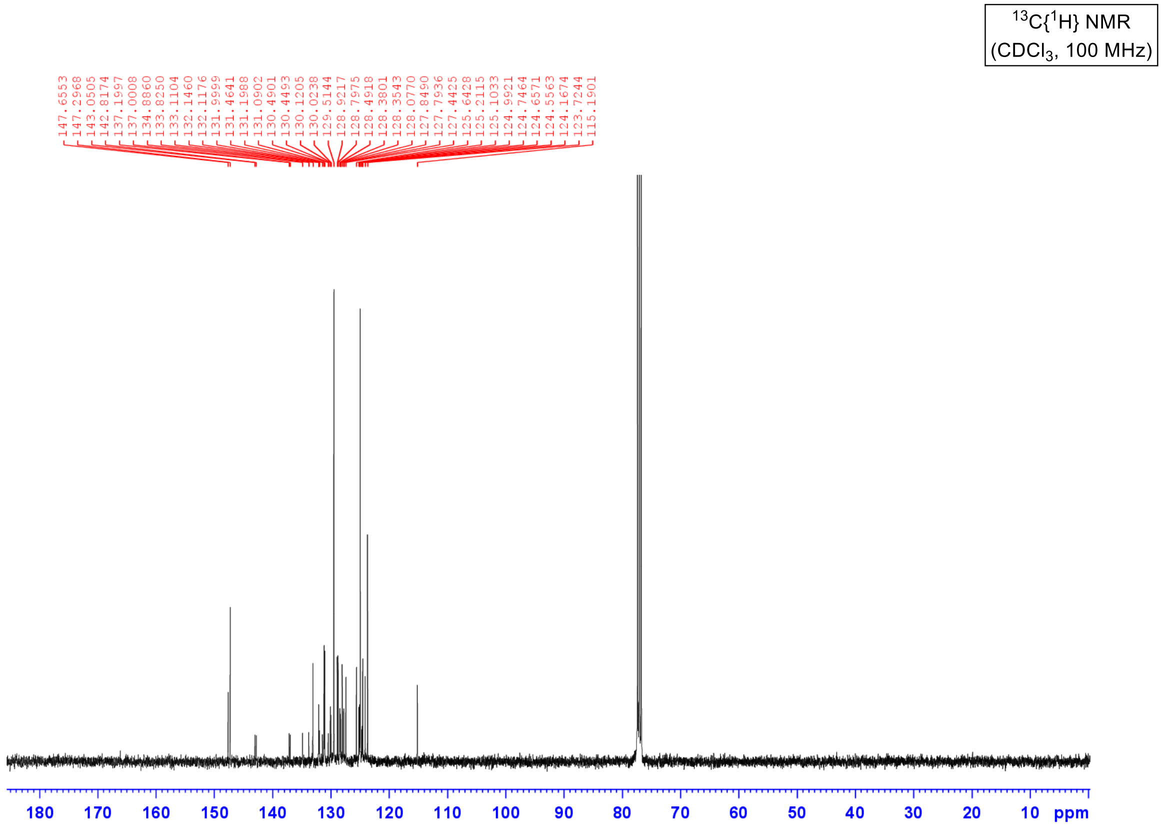

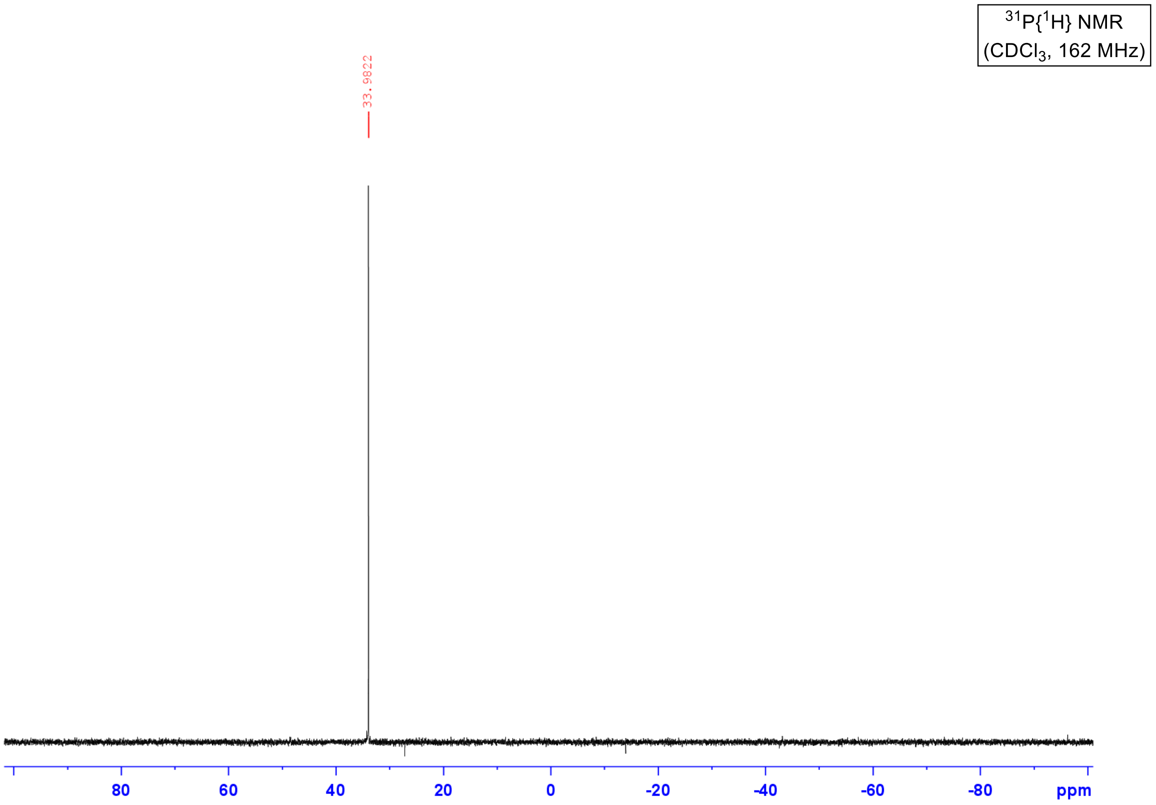

$^{31}P\{^1H\}$ NMR
($CDCl_3$, 162 MHz)
33.9822
80
60
40
20
0
-20
-40
-60
-80
ppm

[$^{1}H$, $^{13}C\{^{1}H\}$, and $^{31}P\{^{1}H\}$ NMR Spectra of **5ac**]

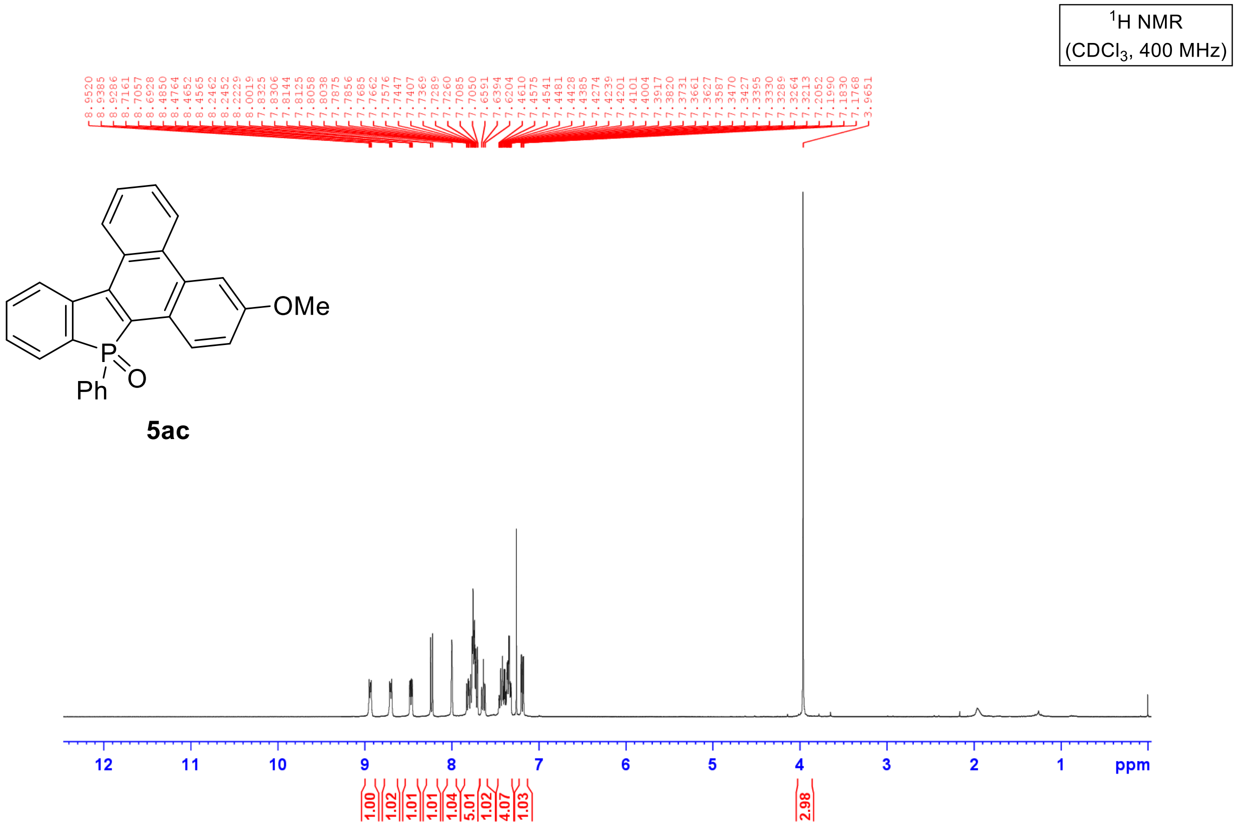


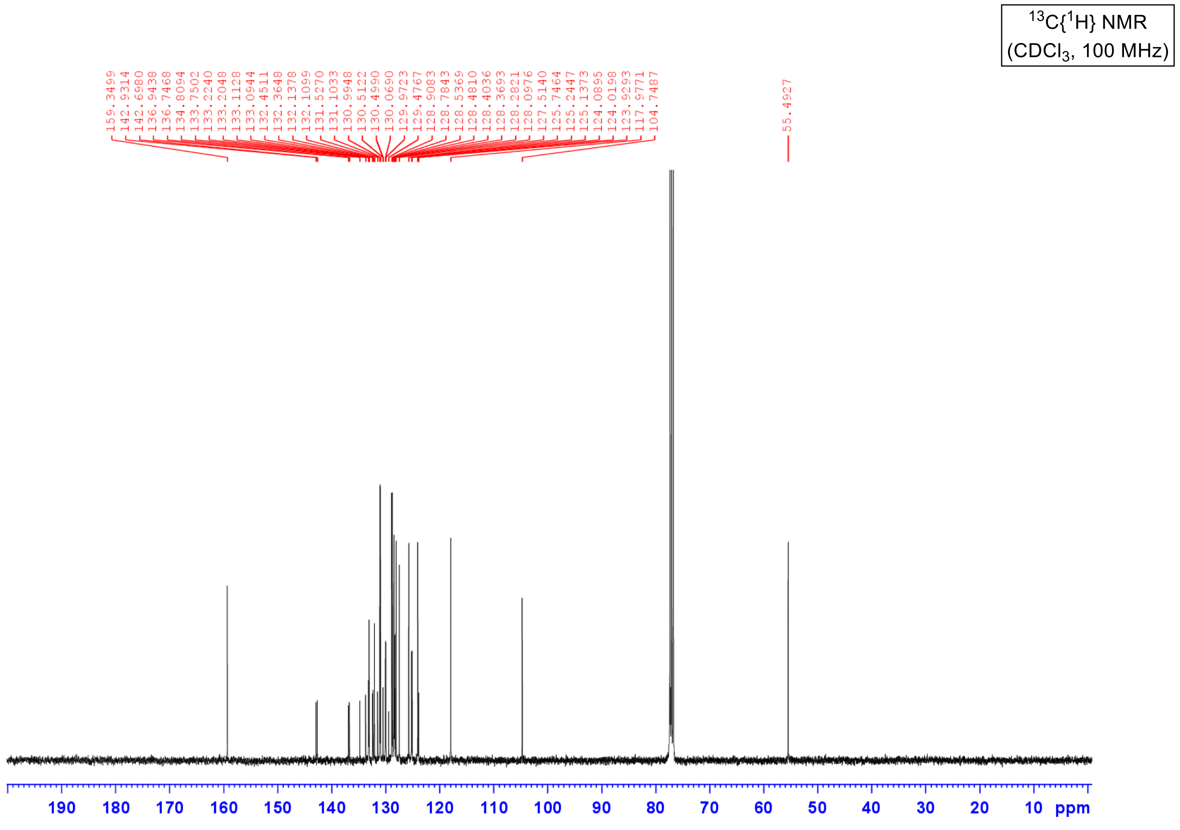

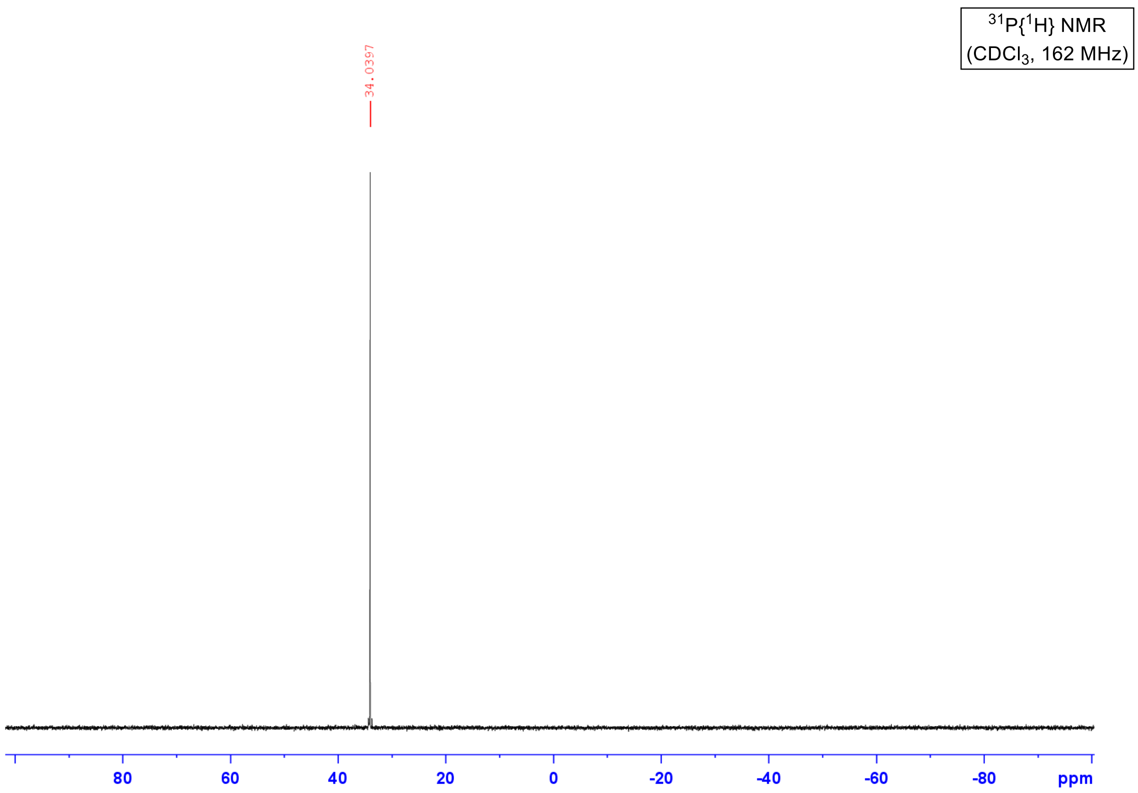
34.0397
$^{31}P\{^1H\}$ NMR
($CDCl_3$, 162 MHz)
80
60
40
20
0
-20
-40
-60
-80
ppm

**References**

(S1) T. H.H. Hsieh and V. M. Dong, *Tetrahedron,* 2009, **65**, 3062–3068.

(S2) Y. Kanazawa, T. Yokota, H. Ogasa, H. Watanabe, T. Hanakawa, S. Soga and M. Kawatsura, *Tetrahedron,* 2015, **71**, 1395–1402.

(S3) K. Nishimura, K. Hirano and M. Miura, *Org. Lett.,* 2020, **22**, 3185–3189.
